# Regulating lattice evolution via an ordered two-dimensional intermediate phase for resilient perovskite solar cells

**DOI:** 10.1038/s41467-026-76425-3

**Published:** 2026-08-11

**Authors:** Xiaoting Ma, Tianyu Sun, Junyi Huang, Dongyang Zhang, Quanming Geng, Haixuan Yu, Zhirong Liu, Ying Xu, Lei Wang, Bing Hu, Han Pan, Yan Shen, Mingkui Wang

**Affiliations:** 1 School of Physics, Huazhong University of Science and Technology, 1037 Luoyu Road, Wuhan, Hubei P R China; 2 Wuhan National Laboratory for Optoelectronics, School of Optical and Electronic Information, Huazhong University of Science and Technology, Wuhan, Hubei P R China; 3 Wuhan Hero Optoelectronics Technology Co., LTD, 6 Huanglongshan North Road, East Lake High-Tech Development Zone, Wuhan, Hubei Province P R China; 4 School of Physics and Optoelectronic Engineering, Yangtze University, Jingzhou, Hubei P R China

**Keywords:** Materials for energy and catalysis, Devices for energy harvesting

## Abstract

The dynamic expansion and contraction of metal halide hybrid perovskite lattices during day-night cycling generate severe mechanical strain, accelerating trap accumulation and device degradation. Here, we show a strain-regulation strategy that employs a highly oriented two-dimensional (2D) perovskite, 1-(*p*-fluorophenyl)biguanide lead iodide, as an ordered intermediate phase to template the vertically oriented growth of the (100) crystal plane of the bulk perovskite layer. During the subsequent thermal annealing process, this 2D intermediate decomposes to release PbI_2_, which directly participates in the crystallization of the three-dimensional (3D) bulk layer. This spatial enhancement of lattice orientation suppresses photo- and thermal-induced lattice expansion and minimizes lattice distortion from 0.22% to 0.09%, thereby mitigating structural deterioration during dynamic operational cycles. Consequently, planar n-i-p Cs_0.05_MA_0.05_FA_0.9_PbI_3_ perovskite solar cells achieve outstanding power conversion efficiencies of 26.32 % for small-area devices (0.06 cm^2^) and 22.25 % for large-area modules (16.8 cm^2^) under one sun illumination. Furthermore, unencapsulated devices retain over 92% of their initial efficiency after 1,800 hours of continuous maximum power point tracking in an N_2_ atmosphere. Notably, the devices exhibit robust diurnal stability, preserving over 89% of their initial efficiency after 40 rigorous light-dark cycles.

## Abstract (web summary)

Solar cells made from metal halide perovskites break down because of daily temperature and light cycles strain their structure and create defects. Ma et al. fixed this by using a specialized 2D template to guide crystal growth. This reduces structural distortion, making the cells efficient and stable.

## Introduction

Metal halide perovskite solar cells (PSCs) have achieved certified power conversion efficiencies (PCEs) exceeding 27%^1–3^, yet their operational stability under real-world conditions remains the critical bottleneck for commercialization^4–6^. This durability gap originates from unconstrained crystallization kinetics during film formation, which yield polycrystalline matrices characterized by severe crystallographic heterogeneity, deep-level defects, and persistent lattice strain^7–9^. Because distinct perovskite facets exhibit disparate optoelectronic properties, uncontrolled grain orientation impairs charge transport and accelerates non-radiative recombination^10–12^. Specifically, cubic perovskite crystals with a preferred (100) orientation deliver superior carrier mobility and lower trap densities than their (110)- or (111)-oriented counterparts^12–14^. Conversely, grain-to-grain misorientation introduces localized lattice mismatch and residual tensile stress, triggering structural dislocations^15^. These localized strain fields perturb the electronic band structure by distorting Pb-X bond lengths, altering Pb-X-Pb bond angles, and diminishing orbital overlap^16^. Consequently, this pervasive strain heterogeneity creates severe transport bottlenecks and drives chronic long-term device degradation^4,17^.

This static disorder is compounded by the intrinsically soft, dynamic nature of organic-inorganic hybrid perovskite lattices^18,19^, which readily deform under external stimuli such as light, heat and electric fields^20–22^. For example, an isotropic lattice expansion of ~0.63% occurs in FA_0.7_MA_0.25_Cs_0.05_PbI_3_ thin films under one sun illumination for 180 minutes^23^. Under realistic day-night operation, periodic illumination and temperature fluctuations impose cyclic expansion and contraction upon the defect-rich matrix^24,25^. This dynamic lattice strain thermodynamically favors defect generation by lowering their formation energies, while kinetically accelerating ion migration via reduced activation barriers^26^. Cumulatively, these processes trigger mechanical failure through microcrack propagation^27^, underscoring the critical necessity of simultaneously suppressing crystallographic disorder and mitigating dynamic lattice deformation to ensure long-term stability.

Precise crystallization control offers a viable pathway to reduce lattice disorder, promote preferential orientation, and relieve residual stress^8^. Consequently, strategies spanning nucleation suppression^28^, high-binding-energy additives^29^, and intermediate-phase engineering^30,31^ have been developed to modulate perovskite crystallization. In particular, two-dimensional (2D) perovskites have emerged as versatile interfacial modifiers^32^, buried 2D/3D heterostructures^33^, and crystallization-directing templates^34–38^. Despite these advances, permanently retained 2D passivation layers suffer from intrinsic limitations^39^. Their low electronic conductivity and propensity to adopt horizontally stacked quantum-well configurations severely impede vertical charge extraction^40^. Furthermore, residual, uncontrolled mixed-dimensional phases within the bulk matrix can introduce parasitic charge-trapping sites^41,42^. More fundamentally, conventional 2D strategies primarily target post-crystallization interfacial architectures or localized heterojunctions^43^, rather than comprehensively directing the early-stage crystallization pathway to enforce orientation uniformity throughout the entire film thickness. Emerging evidence also indicates that initially phase-pure 2D/3D stacks can dynamically evolve during the aging process^44^, meaning the structural fate of the 2D phase intimately dictates long-term device stability^45^. Consequently, whether a transient, non-persistent 2D intermediate can successfully regulate the crystallization pathway and imprint full-space orientational uniformity onto the final 3D framework remains an open and critical question.

Here, we demonstrate that a thermally unstable 2D perovskite, FPGC_2_PbI_4_ (1-(*p*-fluorophenyl)biguanide lead iodide), acts as a transient, ordered intermediate phase to direct the crystallization pathway of Cs_0.05_MA_0.05_FA_0.9_PbI_3_. This oriented 2D phase forms preferentially during the initial stages of thermal annealing, effectively suppressing competing solvate intermediates and templating the growth of a highly aligned 3D framework. Upon further heating, the 2D phase decomposes and directly integrates into the evolving 3D matrix, imprinting full-space orientational uniformity and suppressing cyclic lattice deformation. Mechanistic investigation reveals that this enhanced crystalline arrangement effectively mitigates photo- and thermal-induced lattice expansion, minimizing lattice distortion from 0.22% to 0.09%. Consequently, a regular planar PSC device based on this strategy achieves a PCE of 26.32% for small-area devices (0.06 cm^2^) and 22.25% for large-area modules (16.8 cm^2^) under simulated AM 1.5 G illumination. Furthermore, unencapsulated devices retain over 92% of their initial PCE after 1,800 hours of continuous maximum power point (MPP) tracking at 50 °C under one-sun illumination in an N_2_ atmosphere. Remarkably, under rigorous simulated diurnal cycling (~50 °C under illumination and ~25 °C in the dark), these intermediate-templated PSCs sustain over 89% of their initial PCE after 40 day-night cycles. These findings demonstrate that regulating crystallization via a transient 2D intermediate offers a robust strategy to overcome the intrinsic structural fragility of metal halide perovskites, enabling durable operation under realistic conditions.

## Results

**Fig. 1:**
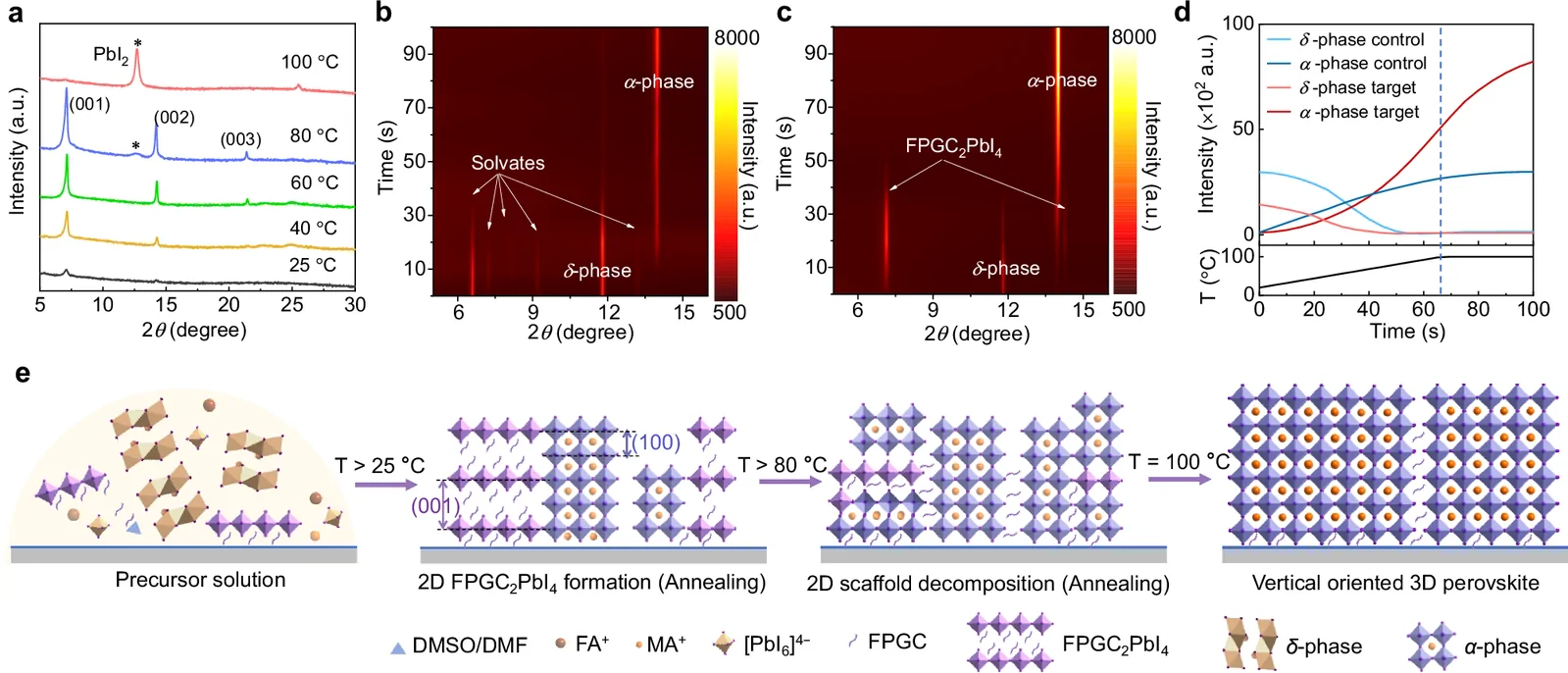
Formation and crystallization kinetics of the three-dimensional (3D) perovskite matrix mediated by the two-dimensional (2D) FPGC_2_PbI_4_ intermediate phase. **a** Temperature-dependent X-ray diffraction (XRD) patterns of an isolated FPGC_2_PbI_4_ thin film annealed at various temperatures. In situ XRD evolution profiles recorded over time during the initial thermal annealing stage for (**b**) the control and (**c**) the target perovskite films. **d** Time-resolved integrated peak intensity corresponding to the (100) *δ*-phase and (100) *α*-phase reflections for the control (blue) and target (red) films. **e** Schematic illustration depicting the ordered intermediate growth strategy and the templated crystallization pathway during the thermal annealing process.

Controlling crystallographic orientation is essential for mitigating lattice disorder and its associated strain^8,10,27^. Here, we introduce a 2D FPGC_2_PbI_4_ perovskite intermediate phase that actively templates the formation of a highly textured 3D perovskite matrix. The molecular structure of the 1-(*p*-fluorophenyl)biguanide hydrochloride (FPGC) precursor is shown in Supplementary Fig. 1. To track the thermal evolution of this intermediate phase, we performed temperature-dependent X-ray diffraction (XRD) analyses on FPGC_2_PbI_4_ thin films (Fig. 1a). Distinct diffraction peaks emerge at 2*θ* = 7.1° and 14.2°, which are assigned to the (001) and (002) reflections of FPGC_2_PbI_4_, respectively. These low-angle reflections demonstrate that the 2D perovskite phase crystallizes preferentially at low temperatures prior to 3D phase nucleation (Fig. 1a and Supplementary Fig. 2). Further XRD profiling reveals that FPGC_2_PbI_4_ reaches optimal crystallinity at ~60 °C, yielding a film with a strongly preferred out-of-plane (00 *l*) orientation.

Upon elevating the temperature above 80 °C, the layered 2D structure collapses, as evidenced by the disappearance of the low-angle reflection and the concomitant emergence of the PbI_2_ characteristic peak at 2*θ* = 12.7°. Thermogravimetric analysis (TGA) confirms that the isolated FPGC molecule remains stable up to 217 °C (Supplementary Fig. 3); thus, the dissolution of the 2D XRD features above 80 °C does not stem from molecular decomposition, but rather from a loss of long-range crystalline order within the FPGC_2_PbI_4_ framework. Grazing-incidence wide-angle X-ray scattering (GIWAXS) patterns exhibit sharp, discrete Bragg spots (Supplementary Fig. 4), verifying the formation of highly ordered, vertically oriented 2D perovskite domains. Based on the Bragg equation, the interplanar spacing of the (002) planes in the 2D FPGC_2_PbI_4_ lattice is calculated to be 6.24 Å. This value yields an exceptional out-of-plane lattice match of 98.4% with the target 3D perovskite phase (Supplementary Table1), highlighting its geometric suitability as a structural template.

Dynamic light scattering (DLS) profiles reveal that the FPGC-containing precursor solution develops an additional population of large colloids ( > 1 μm) alongside a narrower, more concentrated distribution of smaller species in the 30-90 nm range (Supplementary Fig. 5). This behavior points to a profound reconstruction of the precursor colloidal structure, which is consistent with the formation of seed-like aggregates driven by a strong, preferential association between the FPGC cation and PbI_2_^46,47^.

To track the subsequent nucleation and phase-transformation kinetics, we performed in situ XRD characterization while ramp-heating the wet precursor films from 20 to 100 °C within 100 s. For the control films (fabricated without FPGC), multiple diffraction peaks emerge at 2*θ* = 6.6°, 7.2°, 8.1° 9.2° and 13.2° during the initial 0˗40 s interval (Fig. 1b). These reflections signify a disordered mixture of crystalline solvate intermediates, such as ((MA)_2_Pb_3_I_8_·2DMSO or (MA)_2_Pb_3_I_8_·2DMF)^48^. Concurrently, a peak at 2*θ* = 11.8° appears during this 0˗40 s window, corresponding to the hexagonal 2H perovskite polymorph^49^. In stark contrast, the target films incorporating FPGC clearly suppress these parasitic solvate complexes during the first 50 s of thermal processing. Instead, the in situ profiles display only the 2H phase (2*θ* = 11.8°) alongside well-defined reflections from the 2D FPGC_2_PbI_4_ intermediate phase at 2*θ* = 7.1° and 14.2° (Fig. 1c). Notably, the diffraction intensities of these 2D FPGC_2_PbI_4_ reflections increase monotonically over the first 30 s, and then completely vanish after 50 s. This transient behavior matches the thermal stability thresholds established in Fig. 1a, validating the 2D intermediate crystallizes preferentially at lower temperatures but becomes unstable above 80 °C. To quantify these transformation pathways, we extracted and plotted the time-resolved integrated peak intensities of the (100) *δ*-phase and (100) *α*-phase reflections (Fig. 1d). In the control films, the *α*-phase peak intensity rises rapidly between 10 s and 70 s to reach its maximum, accompanied by a synchronous decay of the competing (100) *δ*-phase reflection. For the target films, however, the *α*-phase evolution is noticeably retarded, reaching its maximum plateau only after 90 s. This delay indicates that the transient 2D intermediate establishes a higher kinetic barrier, effectively slowing down bulk crystallization.

This modified crystallization pathway was further substantiated by in situ photoluminescence (PL) measurements conducted during spin-coating and early-stage annealing (Supplementary Fig. 6). Unlike the control film, which exhibits a plateau-like PL signature characteristic of disordered intermediate species during spin-coating, the target film completely bypasses this phase. During the subsequent annealing stage, the PL intensity of the target film takes a longer time to reach its peak value. Collectively, these structural and optical findings demonstrate that the FPGC additive successfully arrests the formation of disordered solvate intermediates at the nucleation onset by introducing a highly ordered, transient 2D template.

The schematic in Fig. 1e illustrates the templated formation mechanism of the 3D perovskite thin films using the FPGC-modified precursor solution. During the initial thermal staging at relatively low temperature (~25 °C), a 2D FPGC_2_PbI_4_ perovskite phase crystallizes preferentially at the film/substrate interface. This localized nucleation effectively suppresses the formation of competing, disordered PbI_2_-solvent crystalline intermediate complexes, such as (MA)_2_Pb_3_I_8_·2DMSO and (MA)_2_Pb_3_I_8_·2DMF^48^, which are otherwise ubiquitous in the control films (Supplementary Fig. 7). Benefiting from a robust (00 *l*) out-of-plane texture and an exceptional lattice-matched heterointerface with the target cubic phase, this transient 2D perovskite FPGC_2_PbI_4_ template guides the ordered assembly of the incoming 3D perovskite framework, effectively imprinting a highly aligned out-of-plane (100) crystal orientation. As the annealing temperature approaches ~80 °C, the metastable 2D FPGC_2_PbI_4_ structure collapses, releasing PbI_2_ and the organic cations. This gradual, thermally regulated dissolution of the 2D lattice reduces the instantaneous chemical supersaturation of the film matrix. By enforcing supply-limited crystallization kinetics for the growing α‑phase, this transient intermediate successfully modulates the growth pathway to yield a highly textured 3D perovskite film with a strongly preferred (100) orientation at 100 °C.

To evaluate the thermodynamic driving forces underlying this mechanism, density functional theory (DFT) calculations were performed (Supplementary Fig. 8 and Supplementary Table 2). The results reveal that the binding energy of the FPGC–PbI_2_ interaction ( − 1.26 eV) is substantially stronger than that of the PbI_2_–DMSO ( − 0.83 eV), PbI_2_˗DMF (−0.70 eV), or PbI_2_˗FA^+^ ( − 1.10 eV). This energetic hierarchy demonstrates that the formation of the 2D FPGC_2_PbI_4_ framework is thermodynamically favored over both the standard solvate adducts and direct *α*-phase nucleation, successfully blocking disordered crystallization pathways at the nucleation onset. Liquid-state proton nuclear magnetic resonance (^1^H NMR) and Fourier transform infrared (FTIR) spectroscopies further validate this precursor-state reconstruction (Supplementary Fig. 9). Upon the introduction of PbI_2_, pronounced chemical shift and vibrational frequency variations emerge within the N˗H and biguanide-framework spectral regions, confirming a robust chemical coordination between FPGC and the lead iodide octahedra in the solution phase.

Complementary spatial characterizations elucidate the distribution and structural evolution of these phases during processing. Dual-side excitation PL spectroscopy profiles exhibit distinctive spectral disparities that confirm the preferential segregation of FPGC to form the 2D perovskite intermediate layer at the buried film/substrate interface during initial deposition (Supplementary Fig. 10). Following full thermal annealing, full-survey X-ray photoelectron spectroscopy (XPS) and time-of-flight secondary ion mass spectrometry (ToF-SIMS) verify that FPGC-derived chemical species are completely retained within the final thin film. Crucially, cross-sectional and two-dimensional ToF-SIMS mapping reveals a localized chemical enrichment at the bulk grain boundaries (Supplementary Figs. 11–13). These observations collectively demonstrate that the transient, interfacial 2D intermediate undergoes spatial redistribution during the high-temperature structural evolution, rather than remaining trapped exclusively at the buried interface.

**Fig. 2:**
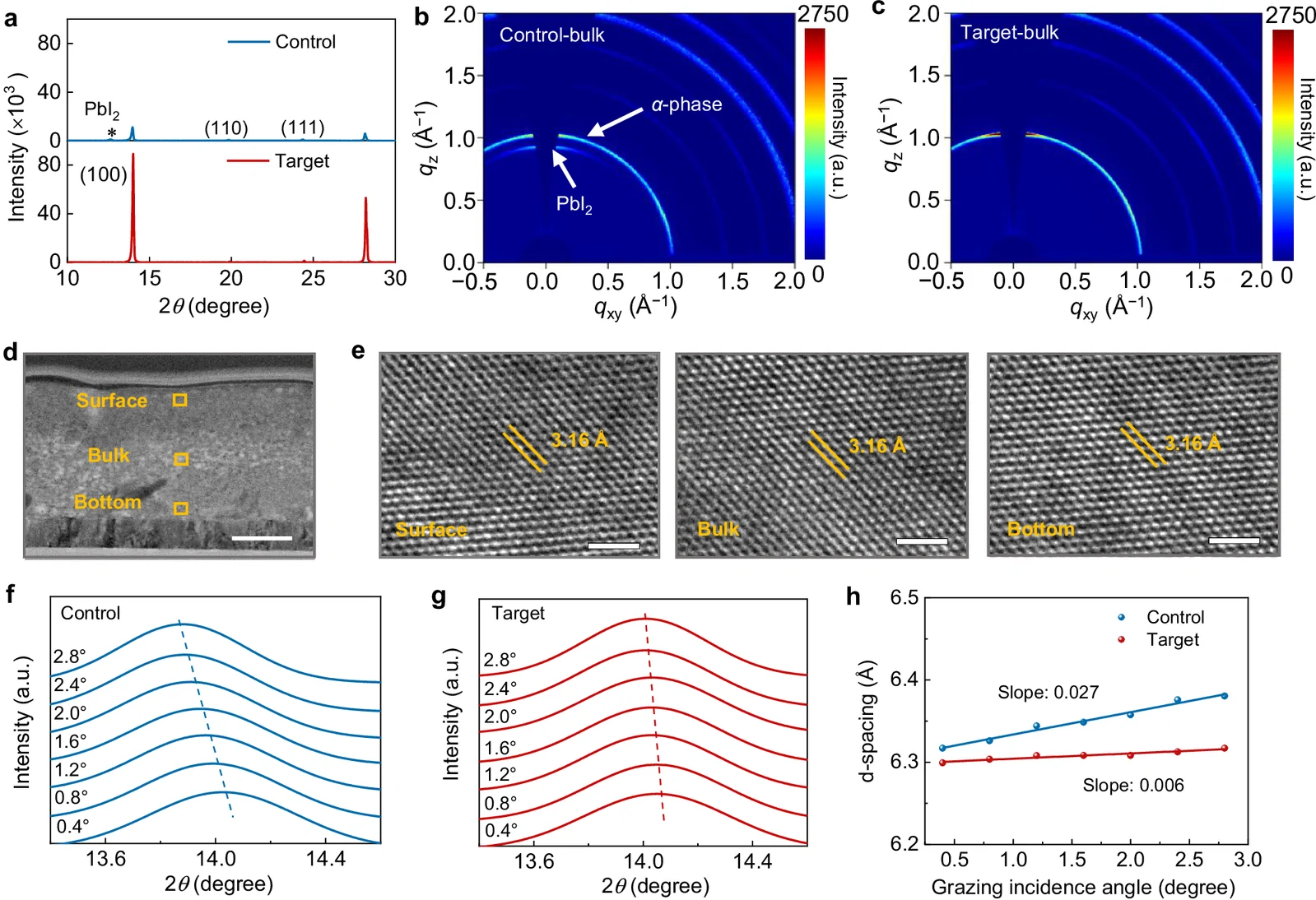
Crystallographic orientation and strain regulation. **a** Comparison of the XRD patterns of the control and target films. Two-dimensional grazing-incidence wide-angle X-ray scattering (2D GIWAXS) patterns of the (**b**) control and (**c**) target films recorded at an incidence angle of 1.0°. **d** Cross-sectional scanning electron microscopy (SEM) image of the target perovskite film. Scale bar, 200 nm. **e** High-resolution lattice fringe images collected from the surface, bulk, and bottom regions indicated in (**d**). Scale bar, 2 nm. Grazing-incidence X-ray diffraction (GIXRD) patterns of the (**f**) control and (**g**) target films at various incident angles. **h** Interplanar spacing of the (100) planes derived from GIXRD measurements plotted as a function of the incidence angle.

The control film exhibits a relatively random crystallographic orientation, characterized by a (100):(110):(111) peak intensity ratio of ~16.7:1:1.5 (Fig. 2a). In the target film, this ratio shifts drastically to ~140:1:2.1, indicating a pronounced texturing of the (100) crystal plane. The integrated peak-area ratio of the (100):(110):(111) reflections similarly increases from 14.1:1:1.3 in the control film to 141:1:1.4 in the target film. Furthermore, the characteristic PbI_2_ diffraction peak is notably absent in the target film, suggesting a more complete precursor conversion. Generally, structural disorder, lattice distortion, and microstrain manifest as peak broadening in XRD patterns^50^. To quantify this effect, the intensity of the (100) reflection was normalized and its full width at half maximum (FWHM) was analyzed. The target film exhibits a significant decrease in FWHM (0.094°) relative to the control (0.117°) (Supplementary Fig. 14), verifying an enhanced long-range structural order. Crucially, the complete absence of low-angle diffraction features in the fully annealed target film confirms that the FPGC_2_PbI_4_ phase functions strictly as a transient intermediate and is not permanently retained as a distinct 2D crystalline phase.

To probe the vertical structural homogeneity across the film thickness, we performed depth-dependent grazing-incidence wide-angle X-ray scattering (GIWAXS) characterization by varying the angle of incidence (*α*_i_) between 0.1° (surface-sensitive) and 1.0° (bulk-penetrating)^51^. The control film exhibits a weak, isotropic (100) diffraction ring with scattering intensity widely dispersed across multiple azimuthal angles (Fig. 2b and Supplementary Fig. 15a)^52^. In stark contrast, the target film displays a highly oriented (100) *α*-phase diffraction arc (*q* ≈ 10 nm^−1^) featuring uniform continuity from the surface through the entire bulk matrix (Fig. 2c and Supplementary Fig. 15b). Azimuthal integration along the diffraction arc at *q* ≈ 10 nm^−1^ reveals a sharp, intense maximum centered at an azimuth angle of ϕ *=* 90° (Supplementary Fig. 16), confirming a highly preferred out-of-plane alignment of the (100) planes. These results demonstrate that the target film possesses superior vertical structural homogeneity and significantly enhanced crystallographic texture relative to the control film^10^. We attribute this behavior to the transient FPGC_2_PbI_4_ intermediate, whose robust (00 *l*) orientation and excellent out-of-plane lattice match with the 3D perovskite matrix effectively template the ordered assembly and out-of-plane (100) growth during bulk crystallization^35^.

Cross-sectional scanning electron microscopy (SEM) imaging details the morphology of the target film (Fig. 2d). To evaluate phase heterogeneity, the interplanar spacing of the lattice was measured across three distinct regions of the perovskite films (*i.e*., the surface, bulk, and bottom). As shown in the corresponding high-resolution transmission electron microscopy (HRTEM) images in Fig. 2e, the target film exhibits a constant interplanar spacing of 3.16 Å across all regions, confirming a structurally uniform 3D perovskite framework throughout the film thickness. In contrast, the control film displays a gradual increase in interplanar spacing from the surface (3.17 Å) to the bottom (3.19 Å) (Supplementary Fig. 17), suggesting depth-dependent lattice distortion. These results indicate that the FPGC-assisted, intermediate-mediated crystallization strategy promotes more uniform crystallization across the entire spatial matrix. Furthermore, control experiments using methylammonium chloride (MACl)-free, MACl-matched, and 1-(3-fluorophenyl)biguanide hydrochloride (3-FPGC)-containing precursors demonstrate that the orientation effect cannot be ascribed to chloride addition alone; instead, it is intrinsically linked to the specific molecular structure of FPGC and its unique capability to form a transient ordered intermediate (Supplementary Figs.18, 19 and Supplementary Note1).

To systematically quantify the depth-dependent residual strain distribution, we conducted depth-resolved grazing-incidence X-ray diffraction (GIXRD) measurements. For the control films, the (100) diffraction peak systematically shifts to lower angles as the incidence angle (*α*_i_) increases from 0.4° to 2.8° (Fig. 2f). According to Bragg’s law, this shift corresponds to an expansion of the (100) interplanar spacing (*d*_100_) from 6.318 Å to 6.383 Å, indicating a pronounced tensile strain gradient propagating toward the buried substrate interface. Conversely, the (100) peak position for the target film remains virtually invariant with increasing *α*_i_ (Fig. 2g). Linear regression fitting of the *d*_100_ versus *α*_i_ profiles yields a slope of just 0.006 Å deg^−1^ for the target film, which is significantly lower than the 0.027 Å deg^−1^ for the control film (Fig. 2h). This finding indicates a markedly suppressed vertical strain gradient in the target film, establishing a structural reinforcement that directly correlates with the observed robust cyclic stability.

**Fig. 3:**
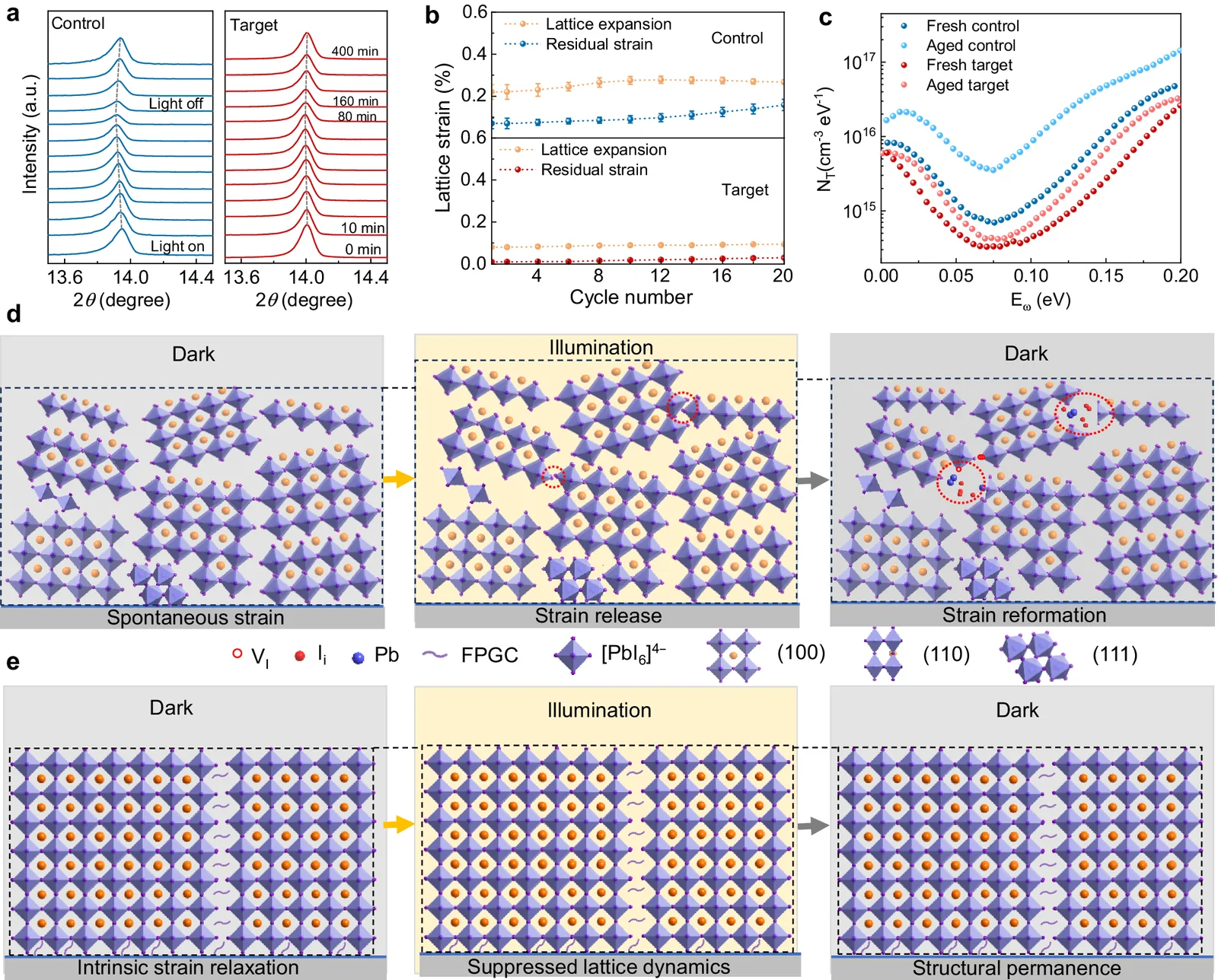
Lattice dynamics and defect evolution under cyclic illumination. **a** In-situ XRD patterns of the control and target films recorded under continuous one-sun illumination (100 mW cm^−2^) and following dark storage. **b** Variation in the (100) interplanar spacing extracted from the XRD analysis over 20 light-dark cycles. Error bars represent the standard deviation derived from fifteen independent film measurements. **c** Trap-state density of the control and target devices before and after aging for 20 simulated day-night cycles. Schematic illustrations showing the structural lattice variation and phase evolution of the (**d**) control and (**e**) target perovskites under cyclic illumination. Dashed red circles highlight defect formation and structural degradation within the perovskite matrix.

We performed in-situ XRD characterization to track the real-time evolution of the perovskite lattice under one‑sun illumination at ~55 °C. During the initial irradiation period (0–70 min), the (100) diffraction peak of the Cs_0.05_MA_0.05_FA_0.9_PbI_3_ control film gradually shifted toward lower angles (Fig. 3a). Quantitative evaluation of the lattice constant for the (100) plane shows a light- and thermal-induced lattice expansion (Δ*d*/*d*) of 0.22% (Fig. 3b). Following a 5 h dark interval paired with cooling to room temperature, the diffraction profile and the (100) peak position nearly reverted to their pristine states. Over the course of 10 cycles, the crystal structure continued a quasi-reversible behavior. However, the residual strain accumulated to ~0.16% in the dark phase after 20 cycles, indicating that an irreversible accumulation of structural deformation. In contrast, the (100) peak of the target perovskite film remained highly symmetric and stationary, indicating negligible volumetric variance (Fig. 3a). Even after 20 light-dark cycles, the target film exhibited a markedly suppressed light- and thermal-state dilation of only ~0.09% alongside a negligible residual dark-state strain of ~0.03% (Fig. 3b). Depth-dependent GIXRD further confirmed that light- and thermal- soaking induces a pronounced vertical lattice deformation profile in the control film, whereas this light- and heat-induced strain heterogeneity is substantially suppressed in the target film (Supplementary Fig. 20).

Angular-frequency-dependent capacitance measurements were conducted to quantify the defect trap-state density (*N*_T_) and its energy distribution during diurnal light-dark cycling. As shown in Fig. 3c, the *N*_T_ of the control device at an energy depth of E_ω_ ≈ 0.07 eV increases from 6.96 × 10^14^ to 3.52 × 10^15 ^cm^−3^ eV^−1^ after 20 cycles, where E_ω_ = *E*_T_ − *E*_V_ (*E*_T_ and *E*_V_ denote the trap energy level and the valence band maximum of the perovskite film, respectively)^53^. In contrast, the target device exhibits only a minor increase in *N*_T_ from 3.30 × 10^14^ to 4.17 × 10^14 ^cm^−3^ eV^−1^ under identical conditions (Fig. 3c). These findings demonstrate that cyclic mechanical strain generated during light-dark operations is a primary driver of defect proliferation. In the control film, the structurally disordered lattice experiences large strain fluctuations during light-dark cycling, which accelerates trap accumulation and irreversible device degradation (Fig. 3d). Conversely, the ordered intermediate-mediated crystallization strategy achieves thorough vertical and lateral spatial uniformity in crystallization and orientation, effectively restricting lattice dynamics to preserve structural integrity (Fig. 3e).

To correlate this preferred crystallographic orientation with film quality, we performed a suite of morphological and photophysical characterizations. Top-view SEM and atomic force microscopy (AFM) images reveal that the target film exhibits significantly larger and more uniform grains with the average diameter increasing from ~600 nm to ~1000 nm, alongside a distinct reduction in secondary PbI_2_ residues compared to the control film (Supplementary Figs. 21 and S22). This structural improvement extends to the buried substrate interface, indicating uniform film growth throughout the entire thickness (Supplementary Fig. 23). Consistently, XRD characterization targeted at the buried interface exhibits the same preferential (100) texture observed at the top surface, confirming through-thickness crystallographic uniformity (Supplementary Fig. 24). Local electronic properties were probed via Kelvin probe force microscopy (KPFM). The target film exhibits a more negative average contact potential difference ( − 231 mV) than the control film (116 mV), accompanied by a much narrower spatial distribution (Supplementary Fig. 25). Conductive AFM (c-AFM) mapping under identical conditions shows that local current collection at the grain boundaries is suppressed relative to the grain interiors in both films. Notably, the target film exhibits a substantially higher global current signal across the scanned area (Supplementary Fig. 26), corroborating enhanced charge transport and extraction dynamics^54^. Carrier recombination dynamics were further evaluated using steady-state photoluminescence (PL), time-resolved PL (TRPL), and transient absorption spectroscopy (TAS). The steady-state PL intensity of the target film is approximately sevenfold higher than that of the control (Supplementary Fig. 27), and its TRPL carrier lifetime increases from 468 ns to 1170 ns (Supplementary Fig. 28). Furthermore, TAS characterization reveals a stronger photobleaching signal at 770 nm for the target film, along with an elongated decay lifetime (Supplementary Figs. 29–31). Collectively, these results demonstrate that the orientation-controlled target films successfully suppress non-radiative recombination pathways.

**Fig. 4:**
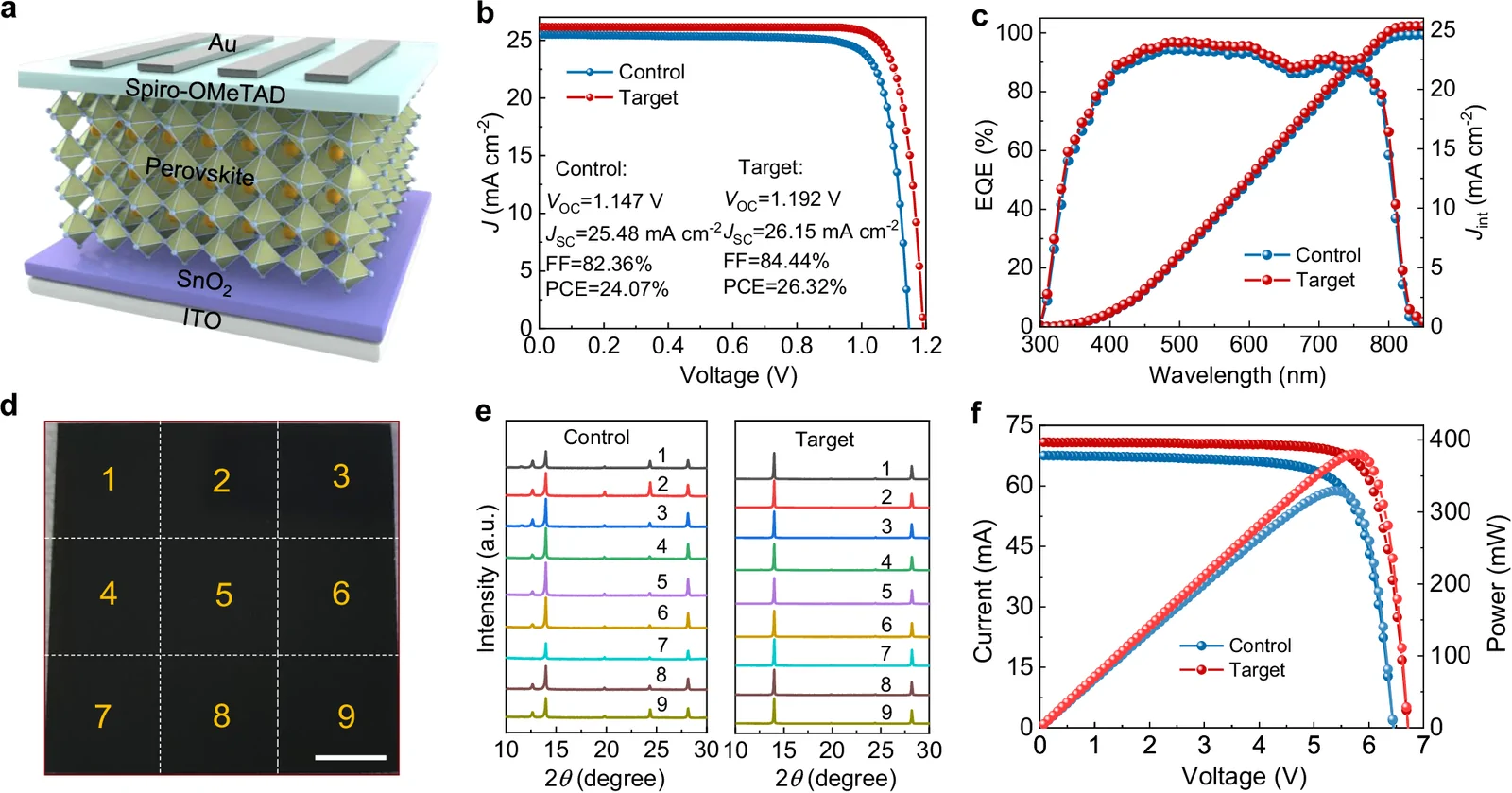
Photovoltaic performance of the small-area solar cells and large-area modules. **a** Schematic architecture of the fabricated perovskite solar cells (PSCs). **b** Current density-voltage (*J*-*V*) curves of the control and the target devices (active area: 0.06 cm^2^). **c** External quantum efficiency (EQE) spectra and corresponding integrated short-circuit current density (*J*_SC_) curves of the control and the target PSCs. **d** Photograph of perovskite film on 5 × 5 cm^2^ substrates. Scale bar, 1 cm. **e** XRD patterns of nine individual segments obtained by cutting a 5 × 5 cm^2^ film for both the control and target samples. **f** *J*-*V* curves of the control and target large-area PSC module (active area: 16.8 cm^2^).

We fabricated planar PSCs with an ITO/SnO_2_/Cs_0.05_MA_0.05_FA_0.9_PbI_3_/spiro-OMeTAD/Au configuration to evaluate their performance and stability (Fig. 4a). Optimization of the FPGC concentration yielded a peak PCE at 0.6 mg mL^−1^ (Supplementary Fig. 32). Figure 4b displays the current density-voltage (*J*-*V*) curves of the control and the target PSCs (active area 0.06 cm^2^) under simulated AM 1.5 G illumination. The champion control device exhibited a PCE of 24.07%, with an open-circuit voltage (*V*_OC_) of 1.147 V, a short-circuit current density (*J*_SC_) of 25.48 mA cm^−2^, and a fill factor (FF) of 82.36%. In contrast, the target device achieved a superior PCE of 26.32%, with a *V*_OC_ of 1.192 V, a *J*_SC_ of 26.15 mA cm^−2^, and an FF of 84.44%. The integrated current densities derived from external quantum efficiency (EQE) spectra (Fig. 4c) are 24.61 and 25.36 mA cm^−2^ for the control and target devices, respectively, showing excellent agreement with the *J*-*V* measurements. Transient photovoltage and transient photocurrent decay profiles (Supplementary Fig. 33) confirm suppressed carrier recombination and enhanced charge collection in the target devices, with the calculated diffusion length increasing from 2275 nm to 5901 nm under one‑sun illumination (Supplementary Fig. 34). To elucidate the physical origin of the enhanced *V*_OC_, we evaluated the photoluminescence quantum yield (PLQY) of the neat perovskite films and the corresponding multilayer device stacks (Supplementary Fig. 35a). The thermodynamic metrics demonstrate that the *V*_OC_ enhancement is primarily dominated by the mitigation of bulk defects. Consistently, the light-intensity-dependent *V*_OC_ ideality factor slope decreases from 1.80 to 1.29 *k*_B_*T*/*q*, further confirming the effective suppression of trap-assisted recombination pathways (Supplementary Fig. 35b).

To assess the in-plane uniformity, we employed a one-step coating process to deposit large-area perovskite films (5 × 5 cm^2^). A representative optical photograph of the macroscale film is displayed in Fig. 4d, highlighting nine distinct spatial coordinates (labeled 1-9) selected for localized structural verification. The preferred crystallographic orientation and peak position of the (100) crystal plane in the target film remain highly consistent across the entire substrate area (Fig. 4e). Consistently, the extracted (110)/(100) XRD intensity ratio across the nine regions decreases sharply from a broad distribution of 0.057–0.110 for the control film to a narrow, minimized range of 0.008–0.014 for the target film (Supplementary Fig. 36), confirming excellent large-area homogeneity. We further fabricated mini-modules on 5 × 5 cm^2^ substrates to evaluate the compatibility of this strategy with technological scale-up. The statistical distributions of the four key photovoltaic parameters collected from 30 individual devices are presented in Supplementary Fig. 37. The target perovskite module achieved an average PCE of 22.07% and a champion value of 22.25% (Fig. 4F), demonstrating the superior upscaling potential of the ordered intermediate-mediated crystallization strategy. In comparison, the control module (aperture area 16.8 cm^2^) exhibited an average PCE of only 18.93% and a champion value of 19.59%. Detailed photovoltaic parameters for these modules are summarized in Supplementary Table 3.

**Fig. 5:**
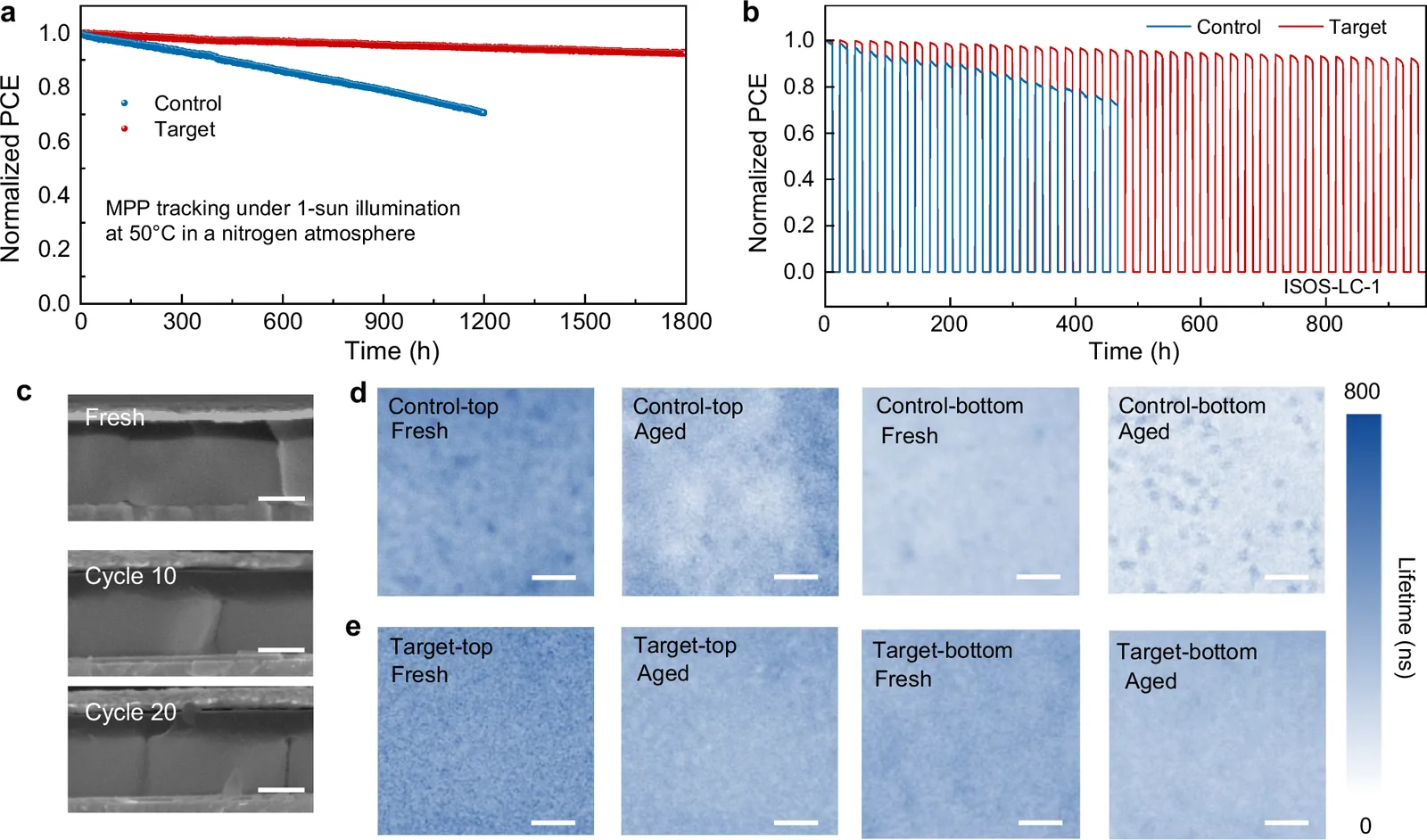
Operational stability under continuous and cyclic stress. **a** Maximum power point (MPP) tracking stability profiles of the control and target devices recorded under continuous one-sun illumination (100 mW cm^−2^) at 50 °C in a nitrogen atmosphere. **b** Normalized power conversion efficiency (PCE) evolution of the control and target PSCs under simulated diurnal day-night cycling (12 h light/12 h dark) in nitrogen. **c** Cross-sectional SEM images of the target devices before and after 20 operational cycles. Scale bar, 300 nm. Photoluminescence lifetime imaging maps of the (**d**) control and (**e**) target perovskite films before and after 20 operational cycles. Scale bar, 10 μm.

Under continuous operational testing (MPP tracking under one-sun illumination at 50 °C in a nitrogen atmosphere), the unencapsulated target device retained over 92% of its initial PCE after 1800 h (Fig. 5a). In contrast, the control device retained only ~70% of its initial efficiency after a shorter duration of 1200 h. To examine whether the spatial distribution of the FPGC-derived species evolves during operation, we performed ToF-SIMS depth profiling on devices after 100 h of continuous operation (Supplementary Fig. 38). The vertical fluorine (F^–^) signal distribution shows no appreciable displacement relative to that of the pristine device, indicating that the FPGC-derived species remain configurationally stable during operational stress. Under realistic diurnal day-night cycling (following the ISOS-LC-1 protocol, ~50 °C during illumination and ~25 °C in the dark), the control device retained only ~71% of its initial PCE after 20 cycles (Fig. 5b). Conversely, the target device exhibited reversible performance profiles, preserving over 89% of its initial PCE even after 40 complete cycles. The partial performance recovery observed during the dark intervals is consistent with the reversible day-night behavior widely reported for PSCs, which is generally associated with the thermal relaxation of light-induced metastable states in the dark^55–58^. In our system, the larger recovery amplitude of the control device suggests a more severe structural perturbation during illumination, which correlates with its larger light- and thermal-induced lattice expansion and greater residual strain accumulation. By contrast, the target device displays minimized day-night efficiency fluctuations, confirming that such structural perturbations are effectively suppressed. Under accelerated thermal cycling at 85 °C, the target device modified with a poly(3-hexylthiophene) hole-transport layer retained 86% of its initial efficiency after 20 day-night cycles, whereas the control device dropped to only ~35% after 15 cycles (Supplementary Figs. 39 and S40). The corresponding initial PCEs of the control and target devices used for this thermal-cycling evaluation were 22.75% and 24.59%, respectively (Supplementary Fig. 41).

To evaluate the thermal dependence of day-night cycling degradation, we performed accelerated aging tests at 45, 65, 80, and 95 °C under repeated light-dark operations (Supplementary Fig. 42a). The degradation rates extracted across this temperature range were fitted using an Arrhenius model (Supplementary Fig. 42b). The fitted regression yields a degradation activation energy (*E*_a_) of 0.224 eV (Supplementary Fig. 42c). Using 55 °C as the reference operational temperature and 95 °C as the accelerated ageing temperature, the corresponding acceleration factor (AF) was calculated to be 2.37^59,60^. On this basis, the projected T_80_ lifetime (defined as the time required for the efficiency to decay to 80% of its initial value) at 55 °C was estimated to be 78.2 cycles. These results indicate that the device degradation under day-night cycling is a thermally activated process and can be quantitatively accelerated under elevated temperature, providing a predictive framework for evaluating long-term operational durability under practical diurnal conditions.

Cross-sectional SEM imaging reveals that the target film exhibits uniform, columnar grains extending across the entire thickness, consistent with the intended vertical orientation control (Fig. 5c). Following 20 simulated diurnal cycles, the target film completely maintains its dense, crack-free morphology (Fig. 5c). Fluorescence lifetime imaging microscopy (FLIM) directly visualizes the spatial divergence in carrier dynamics induced by operational cycling (Fig. 5d, e). In pristine samples, the target film exhibits markedly longer and spatially homogeneous lifetimes from both the top and bottom surfaces (Fig. 5e). Upon aging, the control film suffers a catastrophic collapse in lifetime accompanied by severe spatial heterogeneity, particularly localized at the buried substrate interface where physical macrocracks form (Fig. 5d). In contrast, the aged target film preserves its prolonged, uniform lifetime profiles throughout the entire film thickness (Fig. 5e). By mitigating the root cause of cyclic strain, this ordered intermediate-mediated crystallization strategy effectively suppresses both physical and electronic degradation pathways, enabling exceptionally durable PSCs.

## Discussion

In summary, we have demonstrated a strain-regulation strategy that employs a highly oriented 2D FPGC_2_PbI_4_ perovskite as an ordered intermediate phase to template the crystallization of 3D bulk perovskites. Benefiting from a low formation energy and limited thermal stability, this 2D intermediate acts as a transient, vertically aligned, and lattice-matched platform that effectively moderates the overall crystallization kinetics. This process results in Cs_0.05_MA_0.05_FA_0.9_PbI_3_ films characterized by a highly preferred vertical (100) crystallographic texture and large, uniform grains throughout the entire spatial matrix. Consequently, the resulting planar PSCs achieve an outstanding PCE of 26.32% alongside exceptional operational durability. Under continuous one-sun illumination for 1,800 h in a nitrogen atmosphere, unencapsulated target devices preserve over 92% of their initial efficiency. Crucially, the enhanced through-thickness lattice orientation suppresses light- and thermal-induced lattice expansion and contraction under dynamic operating conditions. Under rigorous diurnal day-night cycling (cycling between 25 to 50 °C), the target devices retain over 89% of their initial PCE after 40 cycles. This work underscores the vital importance of vertical crystallographic uniformity in mitigating cyclic lattice strain, validating the ordered intermediate-mediated crystallization strategy as a robust and scalable route to overcome operational stability challenges for future commercial perovskite photovoltaics.

## Methods

### Materials

Etched indium tin oxide (ITO) glass sheets (*R*_S_ ≈ 8 Ω/sq), formamidinium iodide (FAI, 99.9%) and 2, 2′′, 7, 7′′-tetrakis [N, N-di(4-methoxyphenyl) amino]-9, 9′′-Spirobifluorene (Spiro-OMeTAD, 99.9%) were purchased from Advanced Election Technology CO., Ltd (Yingkou, China). Lead Iodide (PbI_2_, >99%) were acquired from TCI (Japan). Butylammonium Iodide (BAI, 99.5%), Methylammonium Iodide (MAI, 99.5%), methylammonium chloride (MACl, 99.9%), Cesium chloride, (CsCl, 99.9%), 1-(*p*-fluorophenyl)biguanide hydrochloride (FPGC), 1-(3-fluorophenyl)biguanide hydrochloride (3-FPGC), 4-test-butyl pyridine (4-tBP, 96%) and bis (trifluoromethyl sulfonyl)-imide lithium salt (Li-TFSI) were purchased from Xi’an Yuri Solar Co., Ltd. SnO_2_ (Tin (IV) oxide, 15% in H_2_O colloidal dispersion) was purchased from Alfa Aesar. Dimethyl sulfoxide (DMSO, 99.9%), N, N-dimethylformamide (DMF, 99.9%), chlorobenzene (CB, 99.9%), and isopropanol (IPA, 99.9%) were purchased from Sigma-Aldrich. All chemicals were used as received without further purification.

### Preparation of solutions

The SnO_2_-KCl solution was prepared by mixing 15% SnO_2_ aqueous colloidal dispersion with a 10 mg·mL^−1^ KCl solution at a 1:1 volume ratio. The mixed solution was stirred for 2 h, then filtered through a 0.22 µm syringe filter before use. For the precursor solution, a Cs_0.05_MA_0.05_FA_0.9_PbI_3_ solution was prepared by dissolving PbI_2_ (645.4 mg), FAI (216.7 mg), MAI (11.1 mg), CsCl (11.8 mg), and MACl (12.5 mg) in 1 mL of a DMSO and DMF co-solvent mixture (1:4 volume ratio). For the FPGC-modified precursor solutions, varying amounts of FPGC was added directly to the perovskite precursor to achieve final concentrations ranging from 0 to 0.8 mg mL^−1^. The hole transport layer solution was prepared by dissolving 72.3 mg of Spiro-OMeTAD in 1 mL of chlorobenzene. After stirring for 20 min, the solution was doped with 17.5 μL of a Li-TFSI stock solution (520 mg of Li-TFSI dissolved in 1 mL of acetonitrile) and 28.5 μL of tBP. The Spiro-OMeTAD solution was stirred at room temperature for 12 h before use.

### Small-sized n-i-p Solar Cell Fabrication

ITO glass substrates were sequentially cleaned via ultrasonication in a detergent solution, deionized water, acetone, and anhydrous ethanol for 30 min each, followed by drying under a continuous nitrogen flow. Prior to deposition of the electron transport layer, the cleaned ITO glass substrates were treated with UV-ozone for 30 min. The prepared SnO_2_-KCl solution was spin-coated onto the ITO substrate at 4000 rpm for 20 s and subsequently annealed at 150 °C for 20 min in air. After cooling down to room temperature, the ITO glass/SnO_2_ substrates were transferred into a nitrogen-filled glovebox. The prepared Cs_0.05_MA_0.05_FA_0.9_PbI_3_ or the modified FPGC-doped perovskite precursor solutions were spin-coated onto the substrates using a two-step program: 1000 rpm for 5 s and 4000 rpm for 30 s. During the second step, 200 µL of chlorobenzene antisolvent was dropped onto the rotating film 15 s before the end of the spin-coating cycle. The resulting perovskite films were then annealed at 100 °C for 60 min, followed by 150 °C for 10 min. Upon cooling to room temperature, a BAI solution (5 mg mL^−1^ in IPA) was spin-coated onto the perovskite surface at 6000 rpm for 20 s, and the films were annealed at 100 °C for 10 min. The hole transport layer was subsequently deposited by spin-coating the prepared Spiro-OMeTAD solution at 5000 rpm for 20 s. Finally, a ~100-nm-thick gold (Au) top electrode was deposited via thermal evaporation under a high vacuum.

### Perovskite solar minimodule fabrication

Perovskite solar minimodules consisting of six series-connected sub-cells were fabricated on 5 × 5 cm^2^ ITO glass substrates. The series interconnection was realized by monolithic P1, P2, and P3 lines patterned using a laser scribing system (wavelength: 1064 nm, maximum output power: 20 W). The ITO substrate was pre-patterned to form P1 lines (width: 80 μm) using 30% laser power at a scribing speed of 300 mm s^−1^ with a frequency of 65 kHz. The subsequent thin-film deposition steps prior to Au electrode evaporation followed by the identical prodecures described for the small-area devices. The P2 lines (width: 600 μm) were patterned through the perovskite/HTL stack immediately before the Au evaporation step, utilizing 15% laser power at a speed of 300 mm s^−1^ and frequency of 65 kHz. The lateral distance between the P1 and P2 lines was maintained at ~60 μm. Subsequently, a ~100-nm-thick Au back contact was deposited via vacuum thermal evaporation. Finally, the P3 (width: 100 μm) was scribed through the complete film stack under the same laser conditions used for the P2 lines, with a spacing of ~60 μm between the P2 and P3 lines.

### Characterization and measurements

Photocurent-voltage (*J*-*V*) characteristics of the small-area (0.06 cm^2^) and large-area (16.8 cm^2^) solar cells were measured using a Keithley 2400 digital sources meter under simulated AM 1.5 G Solar illumination (100 mW cm^−2^) provided by a 450 W xenon light source solar simulator (Oriel, model 9119) equipped with an AM 1.5 G filter (Oriel, model 91192). Incident photon-to-current efficiency (IPCE) spectra for bias light-free devices were recorded in the wavelength range of 300 to 900 nm using an Oriel IQE200B system. Photoluminescence quantum yield (PLQY) was evaluated utilizing an integrating sphere setup (Horiba Jobin Yvon, Fluorolog) coupled with an Andor Kymera 193i spectrograph and a 600 nm continuous-wave laser (Coherent, OBIS) operating at a 1-sun equivalent photon flux. Surface morphology was examined using field-emission scanning electron microscopy (FE-SEM, Nova NanoSEM 450). Elemental distributions were evaluated via transmission electron microscopy (TEM) operating at an energy resolution of 136 eV. Time-of-flight secondary-ion mass spectrometry (ToF-SIMS) depth profiling was conducted using a 25 keV pulsed Bi^+^ primary ion beam across an analysis area of 100 × 100 μm^2^.

X-ray diffraction (XRD) patterns were recorded on a Shimadzu XRD-6100 diffractometer using Cu Kα radiation. In situ XRD experiments were performed on a Rigaku SmartLab 9 kW diffractometer equipped with a planar detector and a high-temperature stage for precise thermal profiling. Grazing-incidence wide-angle X-ray scattering (GIWAXS) measurements were collected at the Shanghai Synchrotron Radiation Facility (SSRF); all GIWAXS film samples were processed from the corresponding perovskite precursor solutions on quartz substrates. Fourier-transform infrared (FTIR) spectra were captured using a Thermo Scientific Nicolet iS50R spectrometer equipped with a diamond attenuated total reflection (ATR) crystal. ^1^H nuclear magnetic resonance (^1^H NMR) spectra were recorded on a Bruker 600 MHz spectrometer in DMSO-*d*_6_ at a well-controlled, constant temperature of 294 ± 0.2 K.

Steady-state photoluminescence (PL) measurements were carried out with a Horiba LabRAM HR800 fluorescence spectrophotometer under 532 nm laser excitation. In situ PL spectra during film formation were monitored using a commercial Dynamic & Static Photoluminescent Spectrometer (PR-Opt-DSP1 from PURI Materials, China) with the excitation wavelength of 450 nm. Time-resolved photoluminescence (TRPL) kinetics were monitored using a Horiba Scientific DeltaFlex fluorescence lifetime system using a 478 nm pulsed light source. Fluorescence lifetime imaging microscopy (FLIM) was performed with a FLIM300 system (Dalian Chuangrui Spectral Technology Co., Ltd.) using film samples uniformly processed on glass substrates following the exact manufacturing procedures of the device layers. X-ray photoelectron spectroscopy (XPS) was conducted using a Kratos AXIS-ULTRA DLD-600W spectrometer (Shimadzu). Transient photovoltage (TPV) and transient photocurrent (TPC) decay characterizations were carried out using a specialized transient setup (Hua Ming, model III). Background white-light bias generation was established using a diode array, and a pulsed green laser (pulse width: 10 *ns*, repetition rate: 10 Hz) served as the small-signal perturbation source. The transient output parameters were captured directly from the solar cells via a high-speed digital oscilloscope.

### Computational method

First-principles computations were carried out using the Vienna Ab initio Simulation Package (VASP) based on density functional theory (DFT). The exchange-correlation interaction was described using the generalized gradient approximation (GGA) parameterized by the Perdew-Burke-Ernzerhof (PBE) functional. Projector-augmented wave (PAW) pseudopotentials were adopted to describe ion–electron interactions with a plane-wave cutoff energy of 500 eV. The electronic energy and ionic forces were converged to within 10^−5^ eV and 0.03 eV Å^−1^, respectively. A vacuum spacing of 15 Å was applied perpendicular to the slab to avoid unphysical periodic interlayer interactions. For each PbI_2_–ligand system (including DMF, DMSO, FA^+^, and FPGC^+^), multiple initial binding configurations featuring distinct coordination sites and molecular orientations were constructed and fully relaxed. The configuration yielding the lowest total energy was selected for binding-energy comparison.

The binding energy (${\Delta E}_{{{{\rm{b}}}}{{{\rm{ind}}}}}$) was calculated using the following equation:

1 $${\Delta E}_{{{{\rm{b}}}}{{{\rm{ind}}}}}={E}_{{{{\rm{c}}}}{{{\rm{omplex}}}}}-{E}_{{{{{\rm{PbI}}}}}_{2}}-{E}_{{{{\rm{l}}}}{{{\rm{igand}}}}}$$

where$\,{E}_{{{{\rm{c}}}}{{{\rm{omplex}}}}}$, ${E}_{{{{{\rm{PbI}}}}}_{2}}$ and ${E}_{{{{\rm{l}}}}{{{\rm{igand}}}}}$ represent the total energies of the optimized PbI_2_–ligand complex, the isolated PbI_2_ slab, and the isolated ligand, respectively. The PbI_2_–DMF, PbI_2_–DMSO, PbI_3_^−^–FA^+^, and PbI_3_^−^–FPGC^+^ systems were modeled as net-neutral configurations. For the charged species (FA⁺, FPGC⁺, PbI_2_–FA⁺, and PbI_2_–FPGC⁺), the net charge of the simulation cell was set to +1 by reducing the total electron account accordingly.

The three-dimensional charge density difference ($\Delta \rho (r)$) was calculated as:

2 $$\Delta \rho (r)={\rho }_{{{{\rm{c}}}}{{{\rm{omplex}}}}}-{\rho }_{{{{{\rm{PbI}}}}}_{2}}-{\rho }_{{{{\rm{l}}}}{{{\rm{igand}}}}}$$

where ${\rho }_{{{{\rm{c}}}}{{{\rm{omplex}}}}}$, ${\rho }_{{{{{\rm{PbI}}}}}_{2}}$ and ${\rho }_{{{{\rm{l}}}}{{{\rm{igand}}}}}$ are electronic charge densities of the optimized complex and its constituent isolated fragments, respectively, with the fragments maintaining the exact spatial coordinates of the fully relaxed complex geometry.

### Reporting summary

Further information on research design is available in the Nature Research Reporting Summary linked to this article.

## Supplementary information

Supplementary Information

Solar cell Reporting Summary

Transparent Peer Review file

## Source data

Source Data

## Data Availability

The data generated in this study are provided in the Supplementary Information/Source Data file. Source data are provided with this paper.
