# Supplementary material for: Regulating lattice evolution via an ordered two-dimensional intermediate phase for resilient perovskite solar cells
Source: Nat Commun. 2026 Aug 11;17:9624. doi: 10.1038/s41467-026-76425-3 (PMC13554244; doi:10.1038/s41467-026-76425-3)
Supplement: Supplementary file 1 [file 41467_2026_76425_MOESM1_ESM.pdf]

Supporting Information

Regulating lattice evolution *via* an ordered two-dimensional intermediate phase for resilient perovskite solar cells

Xiaoting Ma [1], Tianyu Sun [2], Junyi Huang [2], Dongyang Zhang [2], Quanming Geng [2], Haixuan Yu [2], Zhirong Liu [2], Ying Xu [2], Lei Wang [3], Bing Hu [2,3], Han Pan [4,*], Yan Shen [2,*], and Mingkui Wang [2,*]

[1] School of Physics, Huazhong University of Science and Technology, 1037 Luoyu Road, Wuhan 430074, Hubei, P.R. China

[2] Wuhan National Laboratory for Optoelectronics, School of Optical and Electronic Information, Huazhong University of Science and Technology, Wuhan, Hubei, 430074, P.R China

[3] Wuhan Hero Optoelectronics Technology Co., LTD, 6 Huanglongshan North Road, East Lake High-Tech Development Zone, Wuhan, Hubei Province, P.R China

[4] School of Physics and Optoelectronic Engineering, Yangtze University, Jingzhou 434023, Hubei, P.R China

* Corresponding authors

E-mail: hanpanph@163.com, ciac_sheny@mail.hust.eud.cn, mingkui.wang@mail.hust.edu.cn

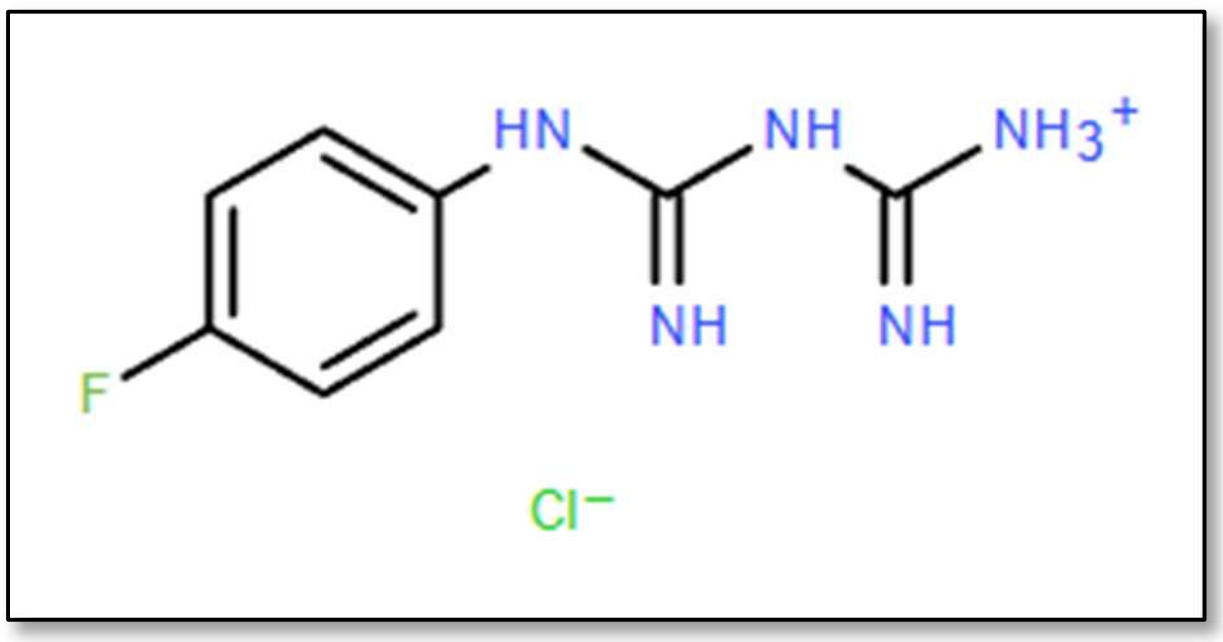

Supplementary Fig. 1 Molecular structure of 1-(*p*-fluorophenyl)biguanide hydrochloride.

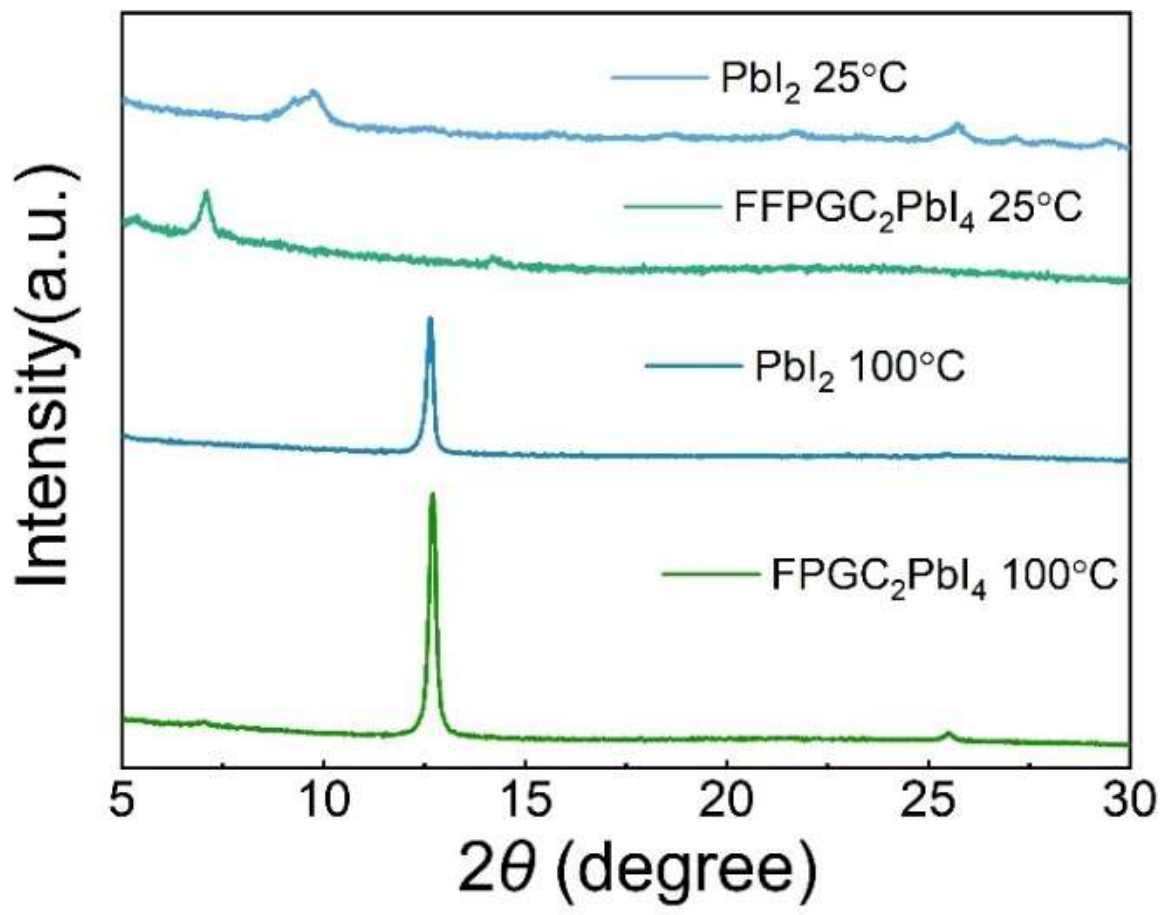

Supplementary Fig. 2 XRD patterns of $FPGC_2PbI_4$ and PbI_2 films obtained by spin-coating the corresponding precursor solutions, both as-deposited (without annealing) and after thermal annealing at 100 °C.

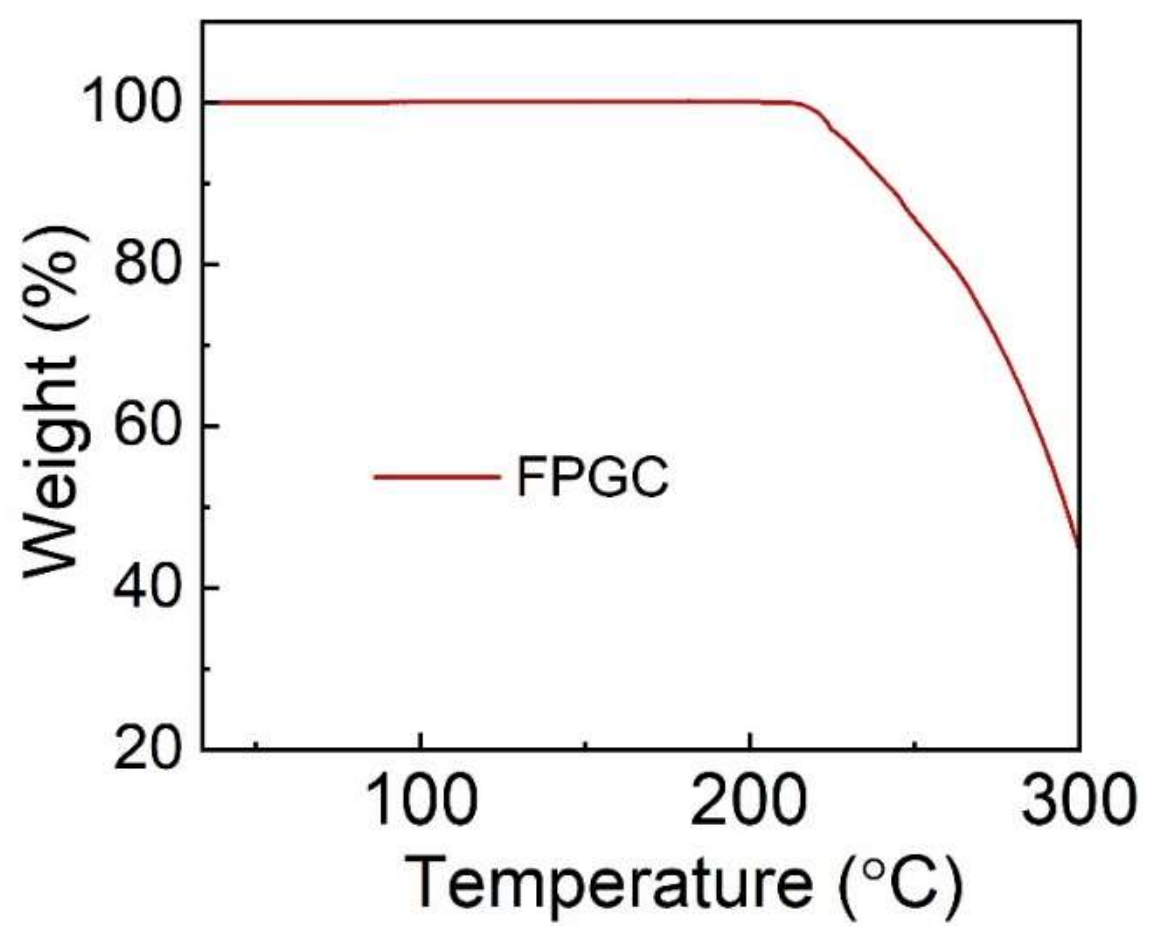

Supplementary Fig. 3 Thermogravimetric analysis profile of the neat FPGC molecular powder.

Thermogravimetric analysis (TGA) demonstrated that the FPGC ligand decomposes at 217 °C, a temperature substantially higher than that at which the low-angle X-ray diffraction (XRD) reflections of the $FPGC_2PbI_4$ phase disappear (~80 °C). Consequently, the loss of this characteristic layered diffraction feature cannot be attributed to the chemical degradation of FPGC. Instead, it signifies an irreversible structural conversion of the long-range-ordered layered phase upon thermal treatment. In layered hybrid halide perovskites, thermally induced disorder within the organic sublattice can couple strongly to the inorganic framework, thereby driving structural phase transitions well below the decomposition temperature of the isolated organic component [1–3]. The concurrent emergence of PbI_2 reflections further indicates that this transformation exceeds a simple, reversible order-disorder transition, involving the complete collapse of the layered hybrid framework [3,4]. This behavior aligns with recent literature demonstrating that a two-dimensional (2D) perovskite phase formed during solution processing can act as a transient intermediate, which subsequently disappears in the final annealed film after successfully guiding oriented three-dimensional (3D) crystallization [5].

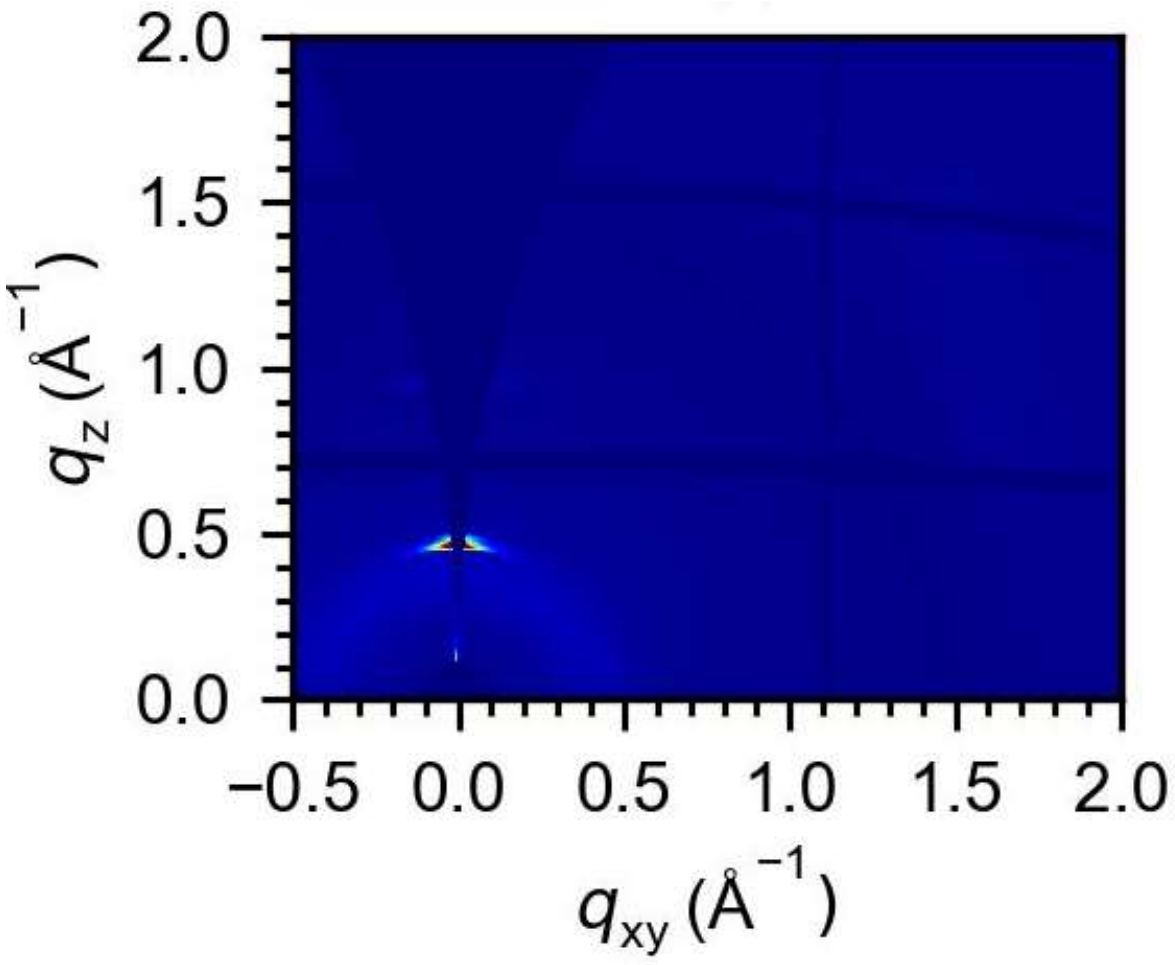

Supplementary Fig. 4 2D GIWAXS patterns of the $FPGC_2PbI_4$ perovskite films.

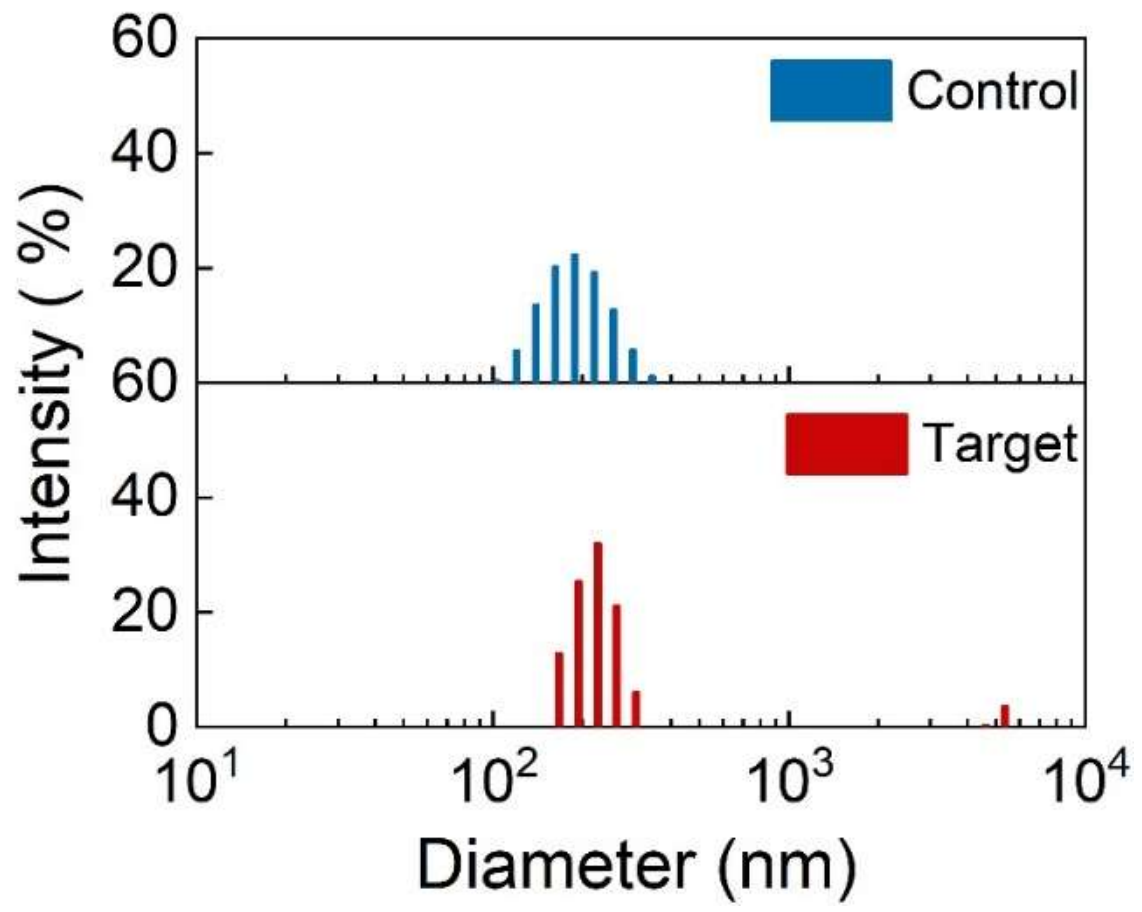

Supplementary Fig. 5 Dynamic light scattering spectra of control and target precursor solutions.

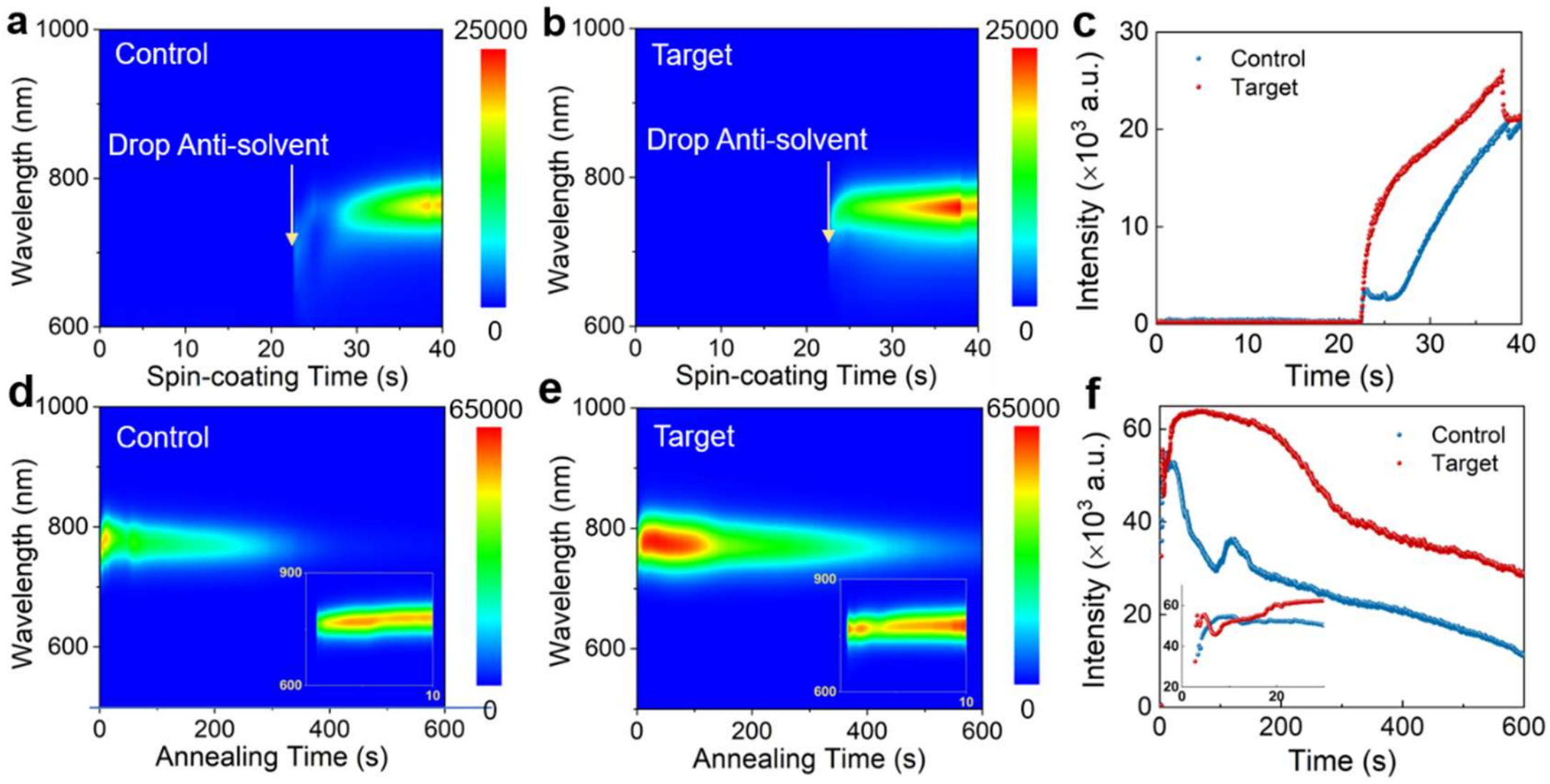

Supplementary Fig. 6 Evaluation of perovskite film growth kinetics via *in situ* photo-luminescence. In situ PL profiles recorded during the spin-coating process of the a) control and b) target perovskite films. c) Comparative analysis of the PL intensity evolution extracted from the spin-coating profiles of the control and target films. In situ PL profiles captured during the initial annealing stage of the d) control and e) target perovskite films (insets: magnified views of the 0–10 s interval). f) Comparative analysis of the PL intensity evolution during the initial annealing stage of the control and target films (inset: magnified view of the 0–30 s interval).

To further elucidate the crystallization kinetics and the specific role of the FPGC ligand during film formation, *in situ* photoluminescence (PL) spectroscopy was conducted during both the spin-coating and initial thermal annealing stages. The control and target films exhibited markedly different PL evolution profiles, indicating distinct crystallization pathways [6] (Supplementary Fig. 6a, b). Following the dripping of the chlorobenzene antisolvent at 22.5 s, the PL intensity of the control film increased gradually, reflecting accelerated solvent extraction [7]. A plateau-like region was subsequently observed from 23.0 to 26.6 s (Supplementary Fig. 6c), during which the PL intensity remained nearly constant while the emission spectral profile was broadly distributed over the 650–800 nm range. This behavior suggests the presence of multiple inhomogeneous intermediate species, which can be attributed to a disordered phase

evolution involving methylammonium (MA)-rich nanocrystalline nuclei [8]. Beyond 26.6 s, the PL intensity rose sharply, and the emission spectra became dominated by a single peak at 757 nm after 28.0 s, signifying the formation and progressive growth of formamidinium (FA)-rich nuclei as solvent evaporation proceeded.

In contrast, the target film displayed a much more rapid increase in PL intensity immediately after antisolvent deposition, featuring a virtually suppressed plateau region and no discernible MA-rich emission characteristics. This result indicates that the formation of disordered intermediate species is strongly inhibited by the presence of FPGC. After 38.0 s, both film systems exhibited characteristic PL quenching, marking the final stage of wet-film conversion. At this juncture, the majority of the solvent had evaporated, exposing the perovskite crystallites to adjacent domains and the local environment, which triggered grain-boundary formation and rapid non-radiative recombination [9].

The PL evolution tracked during thermal annealing over a longer timescale (0–600 s) further revealed distinct kinetic behaviors between the two systems (Supplementary Fig. 6d, e). For the control film, the PL intensity increased rapidly during the initial 0–7.8 s, reaching a maximum as small crystallites continuously grew and coalesced into larger grains. Conversely, the incorporation of FPGC within the target film induced a distinct, intermediate-stabilized pre-growth stage during the first 0–7.0 s. Consequently, the overall crystallization rate was substantially retarded; the PL intensity reached its peak at approximately 21.6 s and remained elevated for a prolonged duration (Supplementary Fig. 6f). These findings collectively demonstrate that FPGC regulates the crystallization pathway by introducing a stable, transient intermediate phase. This action effectively suppresses disordered early-stage phase segregation and enables a slower, well-controlled crystal growth process. This kinetic modulation directly correlates with the observed formation of larger grains, improved crystallinity, and a more preferentially oriented final perovskite film.

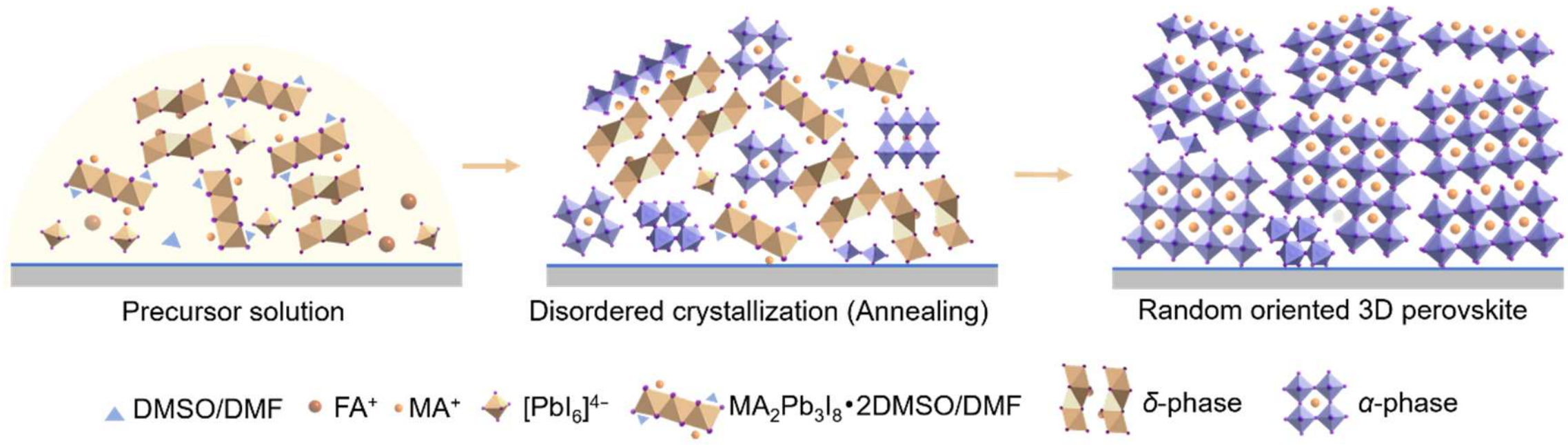

Supplementary Fig. 7 Schematic illustration of the possible phase evolution in the control perovskite film during annealing.

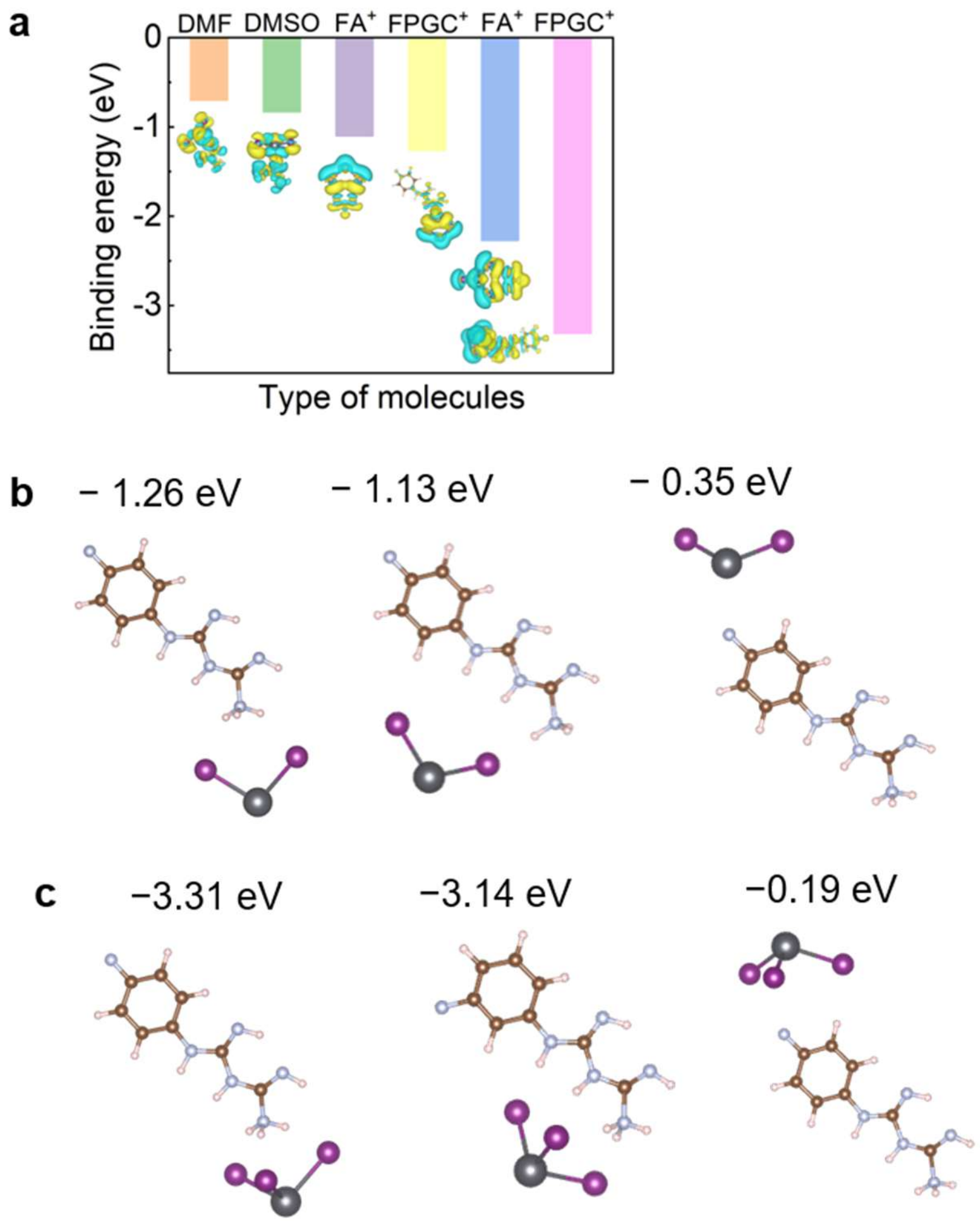

Supplementary Fig. 8 Density functional theory calculations of ligand-perovskite interactions. a) Calculated binding energies and three-dimensional charge density difference profiles for PbI_2 interacting with DMF, DMSO, FA^+ and $FPGC^+$ ligands, along with the binding energies and electrostatic potential maps of the FA^+–PbI_3^- and $FPGC^+$–PbI_3^- ion-pair systems. The yellow and cyan isosurfaces denote electron accumulation and depletion regions, respectively, evaluated at a constant isosurface value of 0.0004 e Å^{-3}. b) Distinct fully relaxed binding configurations optimized for the PbI_2–$FPGC^+$ coordination complex. c) Distinct fully relaxed binding configurations optimized for the PbI_3^- and $FPGC^+$ ion pair.

To evaluate the molecular interaction strengths governing the precursor chemistry, density functional theory (DFT) calculations were carried out to determine the binding energies between PbI_2 and various species, including DMF, DMSO, FA^+, and $FPGC^+$ (Supplementary Fig. 8). To ensure a rigorous comparison, multiple initial coordination

geometries were examined for the FA^+–PbI_3^- and $FPGC^+$–PbI_3^- configurations, from which the lowest-energy states were selected for binding-energy calculations and three-dimensional charge density difference analyses. The resulting charge density difference profiles revealed pronounced charge redistribution localized at the PbI_2–$FPGC^+$ interface, indicating strong electronic coupling between the $FPGC^+$ cation and the PbI_2 framework. The calculated binding energy between $FPGC^+$ and PbI_2 was found to be −1.26 eV, which is significantly stronger than those calculated for PbI_2–DMF (−0.70 eV), PbI_2–DMSO (−0.83 eV), and PbI_2–FA^+ (−1.10 eV). Although absolute binding energies can exhibit sensitivity to the specific computational framework, exchange-correlation functional, plane-wave cutoff, dispersion corrections, and implicit solvation treatments, the relative trend established here is in excellent agreement with established literature on PbI_2–solvent interactions [9-14], validating the physical reliability of our models.

Given that PbI_2 readily coordinates with iodide ions to form iodoplumbate coordinates (such as PbI_3^-) within the complex precursor solution environment, the binding energies of the FA^+–PbI_3^- and $FPGC^+$–PbI_3^- ion-pair systems were further investigated. The binding energy of the $FPGC^+$–PbI_3^- assembly was determined to be −3.31 eV, which substantially exceeds that of the FA^+–PbI_3^- counterpart (−2.27 eV). This disparity confirms the superior thermodynamic affinity of the $FPGC^+$ cation toward the iodoplumbate framework. Collectively, these computational findings demonstrate that $FPGC^+$ can more effectively coordinate with plumbate–iodide components to guide the assembly of the transient $FPGC_2PbI_4$ intermediate phase, thereby successfully suppressing uncontrolled, amorphous phase segregation during the initial stage of perovskite crystallization.

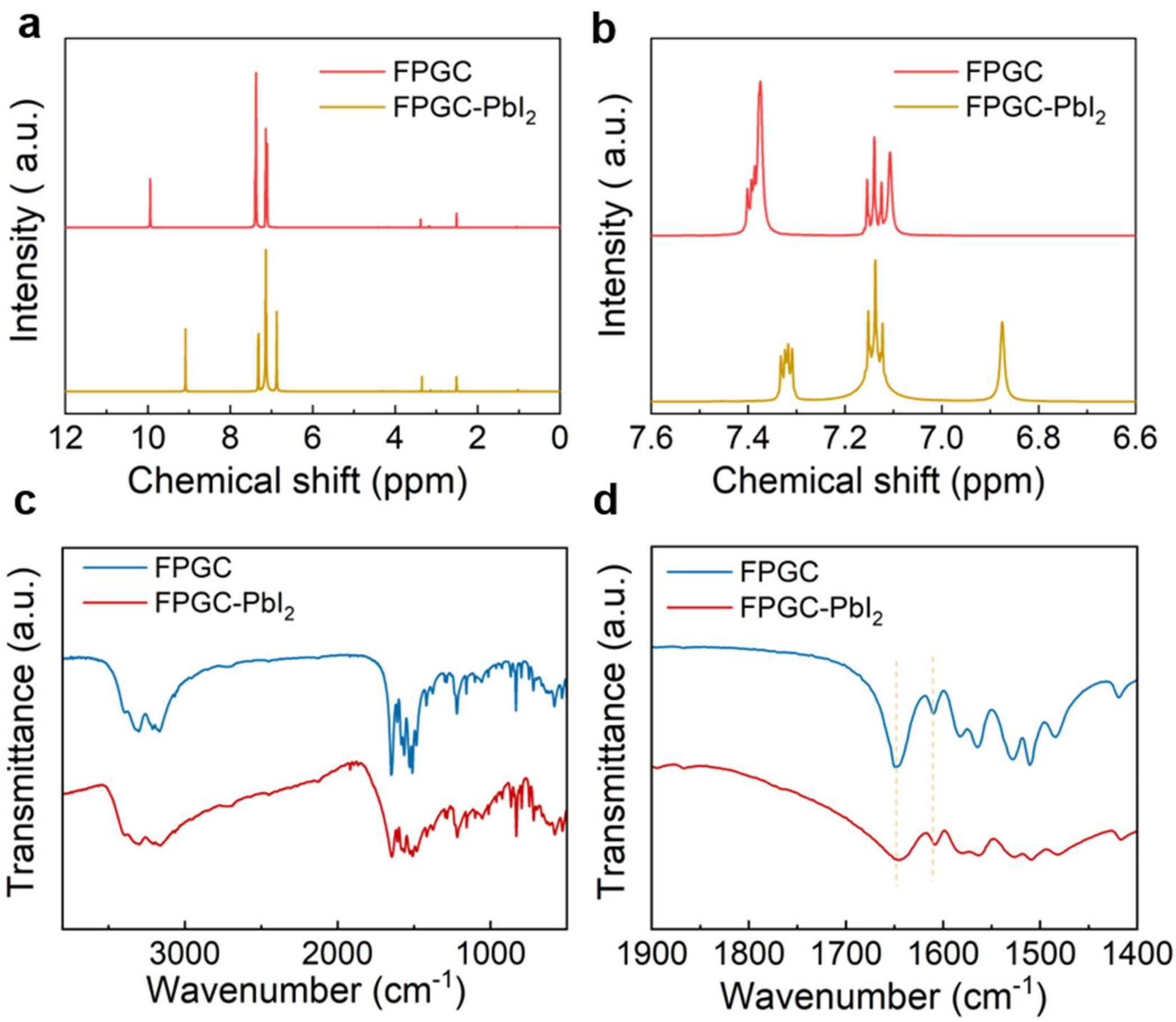

Supplementary Fig. 9 FTIR and ¹H NMR analysis of FPGC-PbI$_2$ molecular interactions. a) ^{1}H NMR spectra of FPGC and FPGC+PbI$_2$ in DMSO-*d*6. b) Magnified view of the highlighted region in a). c) FTIR spectra of the FPGC and FPGC-PbI$_2$. d) Magnified view of the highlighted region in c).

To experimentally probe the intermolecular interactions within the precursor solution, ^{1}H nuclear magnetic resonance (^{1}H NMR) spectroscopy was performed. As illustrated in the ^{1}H NMR spectra (Supplementary Fig. 9a, b), the introduction of PbI$_2$ into the FPGC solution induced a significant upfield shift of the resonance peak corresponding to the protonated biguanide amine $-NH_3^+$ group, moving from 9.94 to 9.08 ppm. Concurrently, the multi-peak resonance assigned to the secondary N-H groups of the biguanide backbone shifted downfield from 7.41–7.33 ppm to 7.35–7.29 ppm. These coordinate variations demonstrate a pronounced change in local electronic shielding, confirming strong chemical interactions between the FPGC cation and the PbI$_2$ framework.

To further elucidate these coordinate frameworks, Fourier-transform infrared (FTIR) spectroscopy was conducted on the corresponding thin films (Supplementary Fig. 9c, d). Upon coordination with PbI_2, the characteristic N-H stretching vibrational band of pristine FPGC at 3297 cm^{-1} exhibited clear broadening, signifying a substantial alteration in the local hydrogen-bonding network surrounding the biguanide moiety. Similarly, the N-H bending mode (1609 cm^{-1}) and the C=N stretching mode (1649 cm^{-1}) of the FPGC framework both shifted to lower wavenumbers, appearing at 1607 and 1643 cm^{-1}, respectively. These vibrational modifications provide definitive evidence of specific chemical coupling, most notably through N···Pb coordination bonds alongside secondary N–H···I hydrogen-bonding networks. Collectively, these precursor-state and wet-film spectroscopic observations substantiate the pre-organization of PbI_2–ligand complex species, establishing a chemical foundation that directly aligns with the subsequent formation of the transient FPGC-derived intermediate phase during film crystallization.

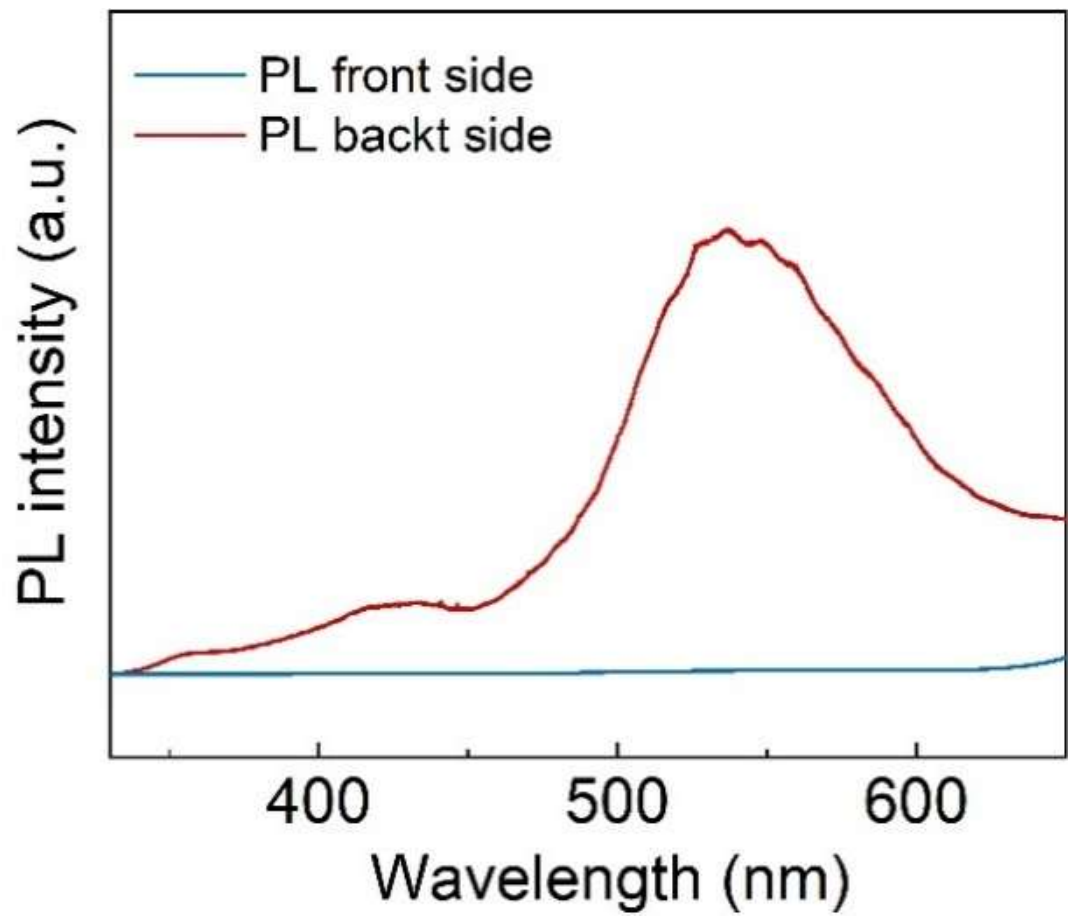

Supplementary Fig. 10 PL spectra of perovskite films with different incident directions.

Dual-side excitation photoluminescence (PL) spectroscopy was conducted to determine the final structural state of the FPGC ligand within the perovskite films, specifically distinguishing between isolated, dispersed cations and assembled two-dimensional (2D) networks. Following a low-temperature thermal treatment at 60 °C for 10 min, the resulting films exhibited distinct spatial variations in their optical profiles. Upon excitation from the front surface (the film/air interface), the sample displayed negligible emission features. Conversely, excitation from the rear surface (the film/substrate interface) revealed multiple distinct, well-resolved emission peaks located at 357, 428, and 530 nm, which provide unambiguous evidence of localized 2D perovskite phases. This pronounced asymmetric spectral response demonstrates that the FPGC ligand preferentially segregates at the buried film/substrate interface to form a structured 2D perovskite bottom layer.

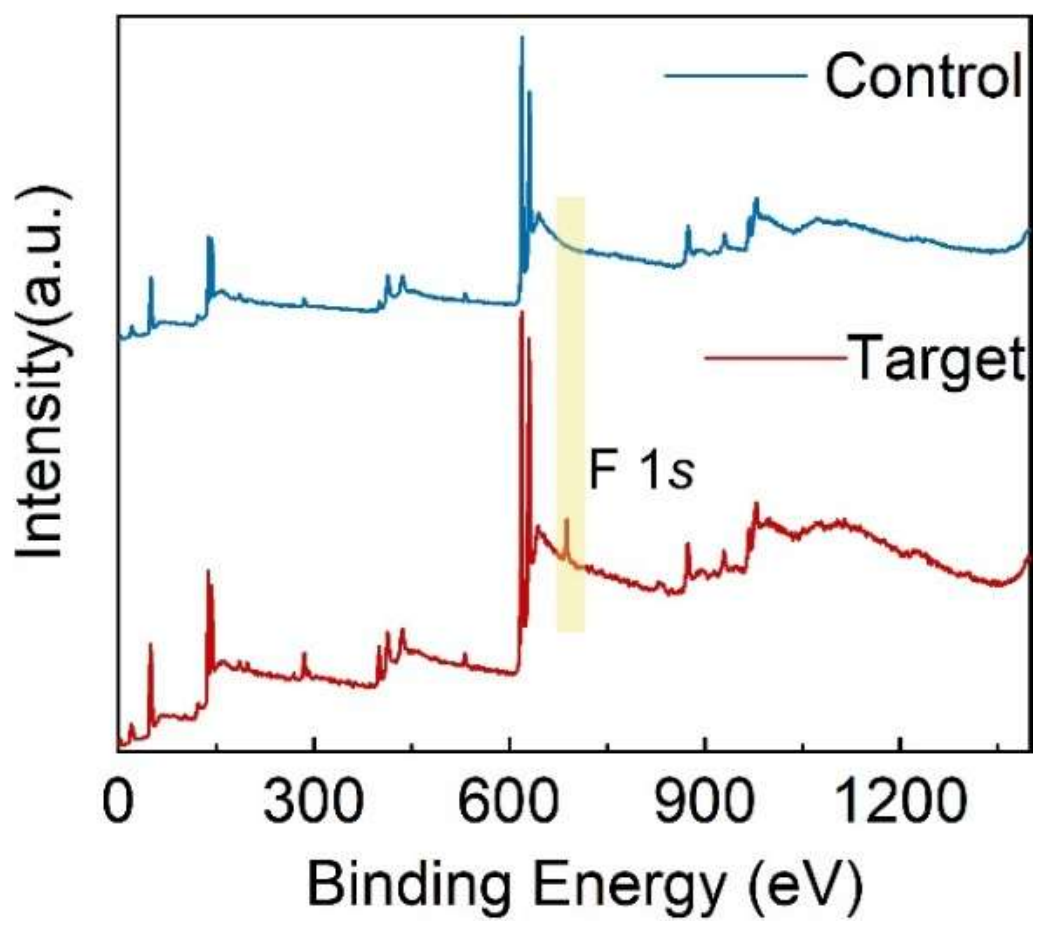

Supplementary Fig. 11 Full-survey XPS spectra of control and target perovskite film.

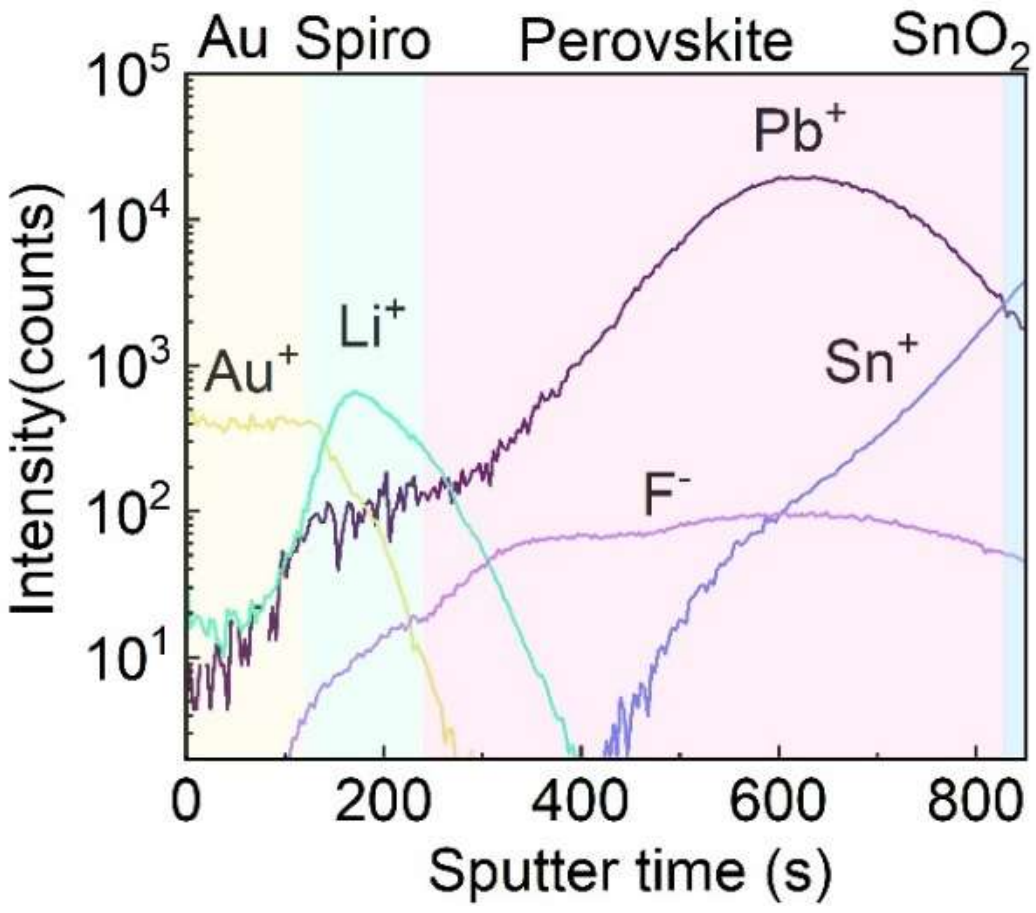

Supplementary Fig. 12 TOF-SIMS spectra of the target perovskite devices.

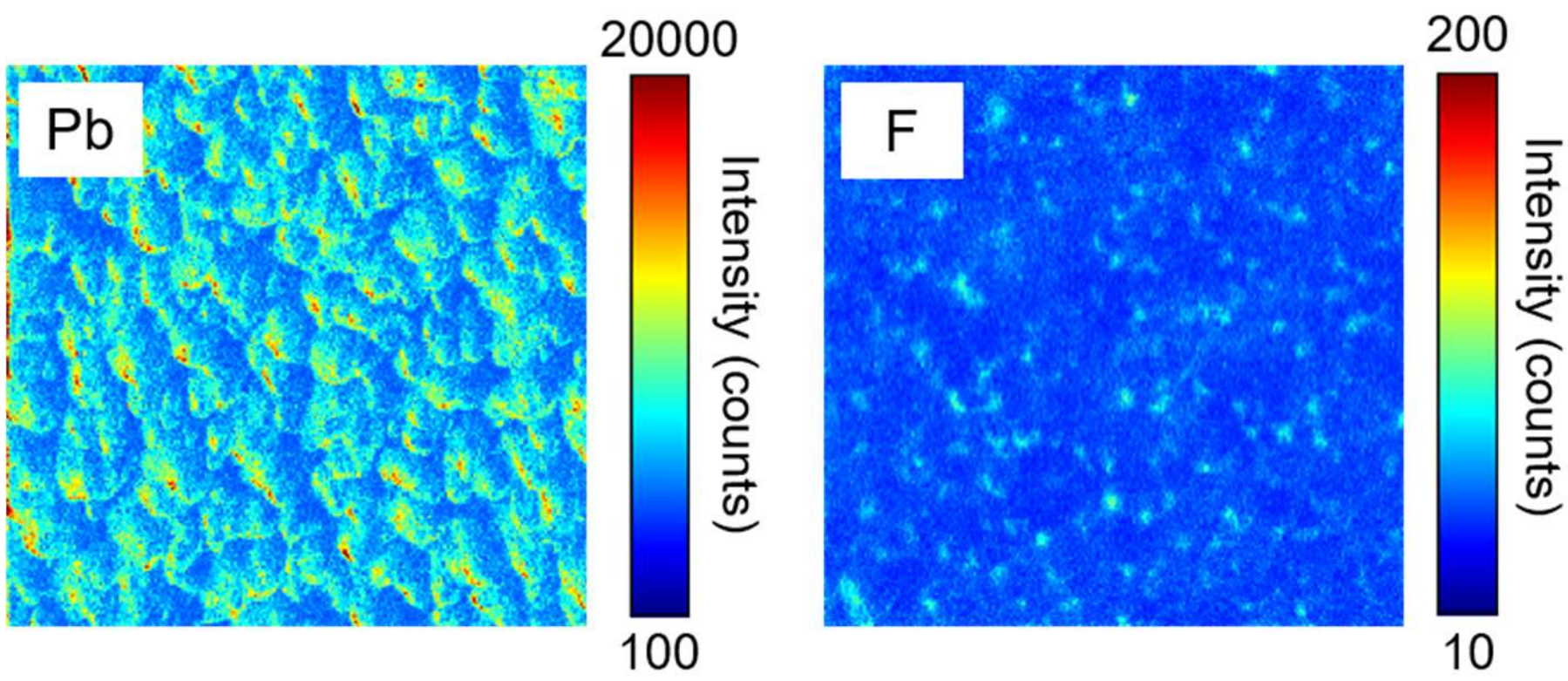

Supplementary Fig. 13 ToF-SIMS 2D imaging map of Pb and F elemental distribution on the FPGC-contained perovskite layer.

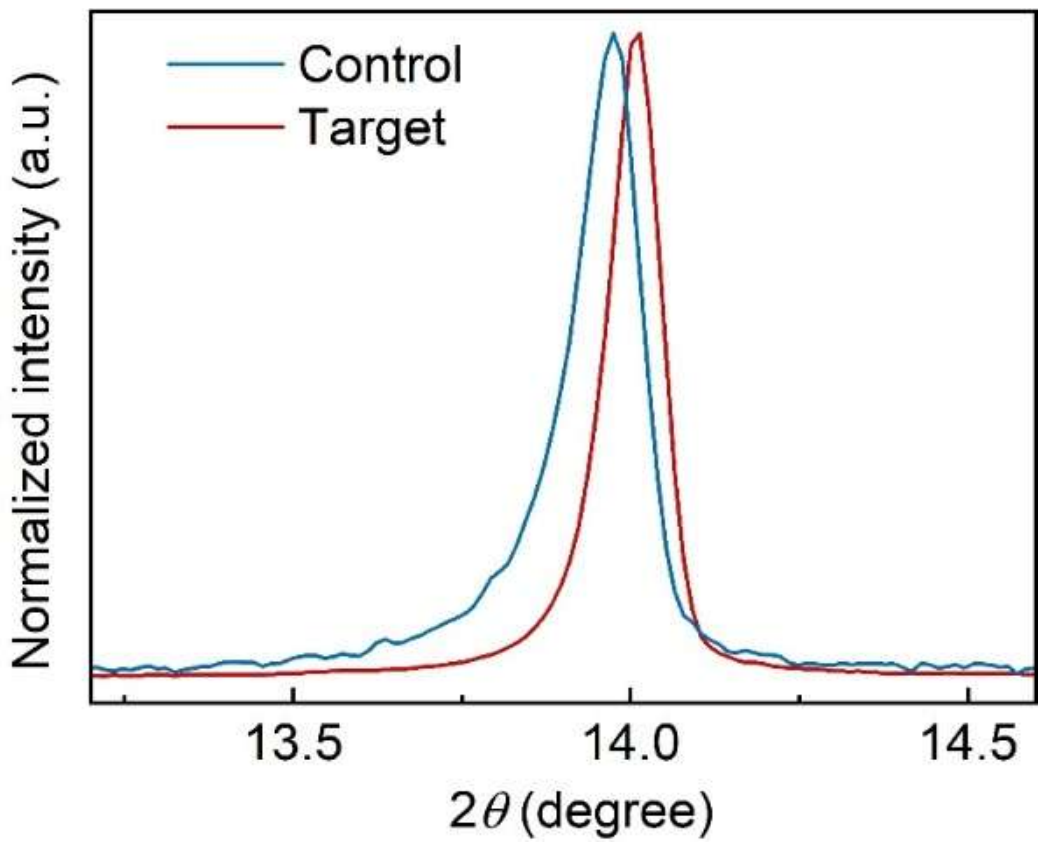

Supplementary Fig. 14 XRD patterns of the control and target thin films.

As illustrated in Supplementary Fig. 14, the primary (110) diffraction peak of the perovskite film undergoes a distinct shift toward a higher scattering angle, moving from 13.975° for the control film to 14.007° for the target film. According to Bragg's law, this displacement signifies a reduction in the corresponding interplanar *d*-spacing, confirming a slight lattice contraction within the target film framework. This structural modulation is attributed to the altered crystallization pathway mediated by the FPGC ligand, which guides the assembly of a more highly packed and compact final lattice state relative to the control matrix. Such a pre-contracted lattice framework is expected to play a critical role in mitigating subsequent light- and thermally induced lattice expansion during operation, thereby contributing to the enhanced long-term structural robustness of the device [15].

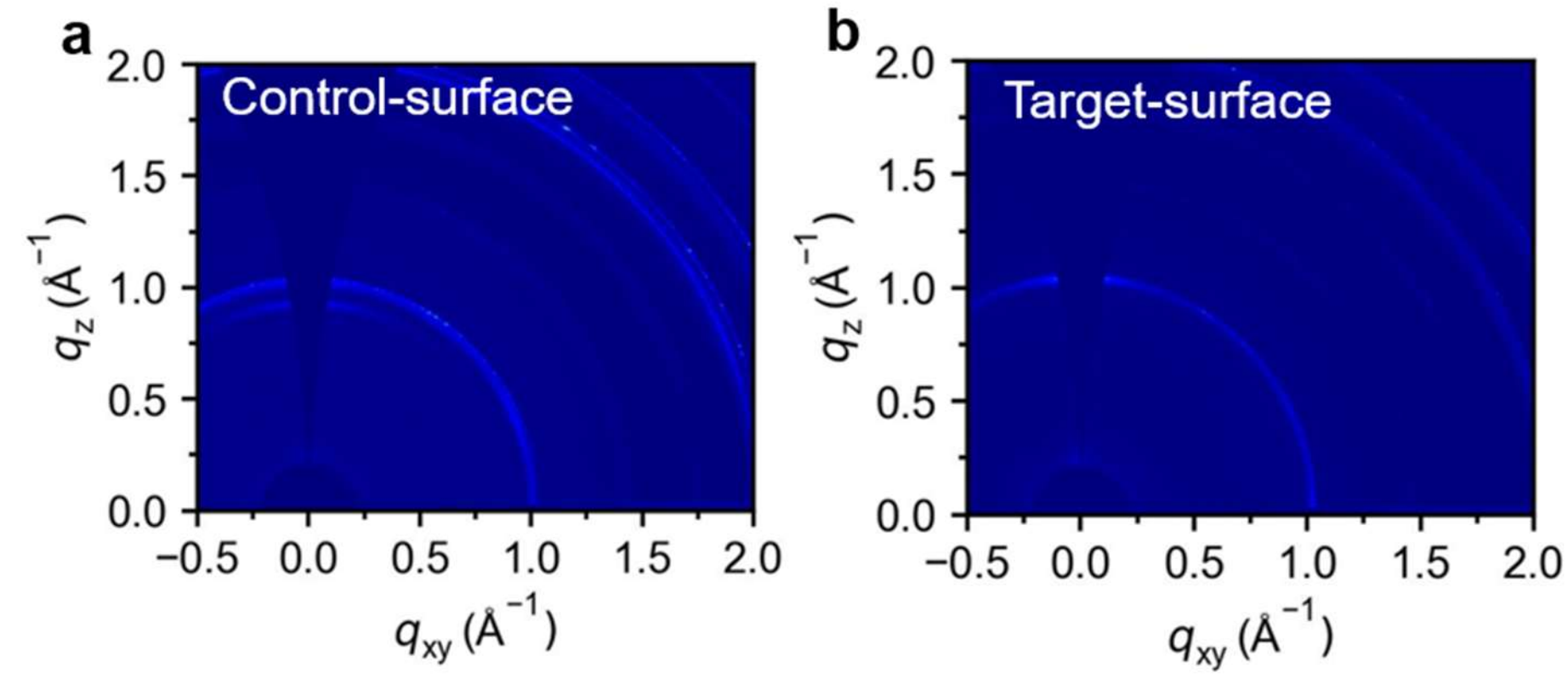

Supplementary Fig. 15 2D GIWAXS patterns of the a) control and b) target perovskite films with incident angle of 0.1°, respectively.

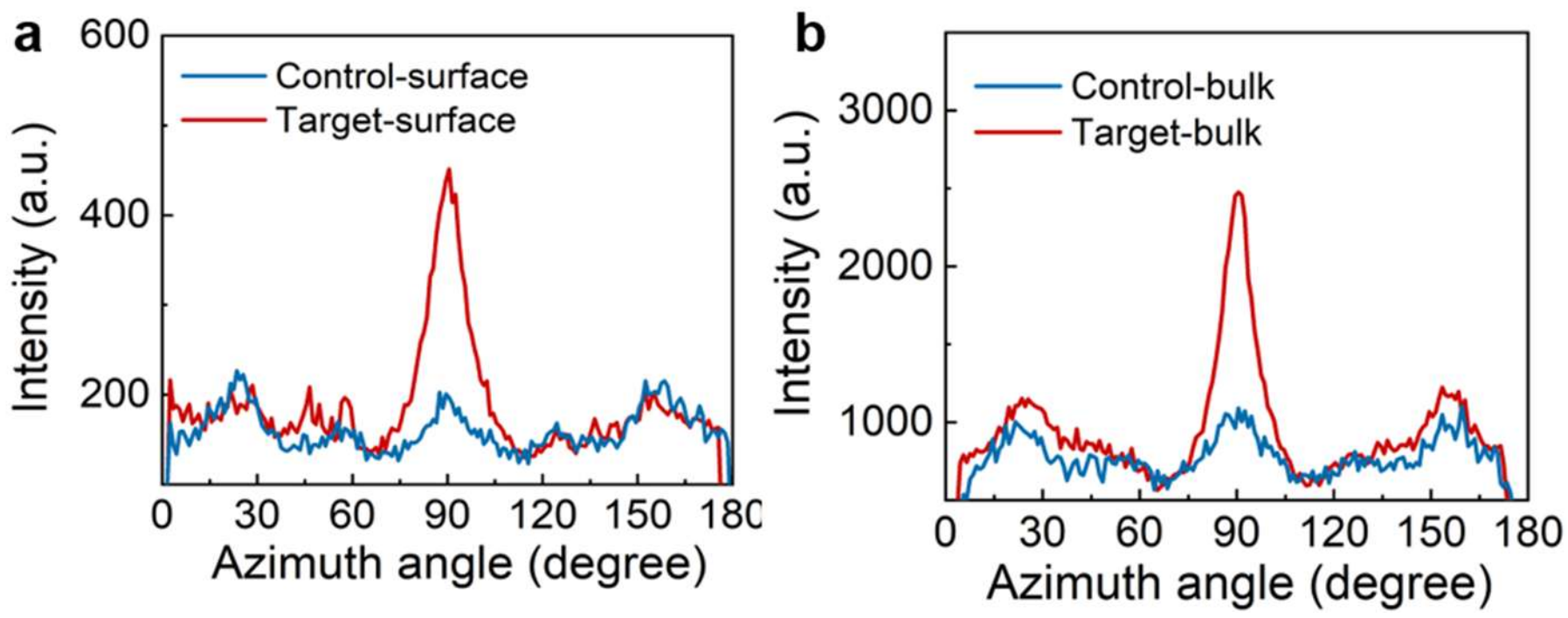

Supplementary Fig. 16 Integrated intensity plots azimuthally along the ring at q_{xy} = 10 nm^{-1}.

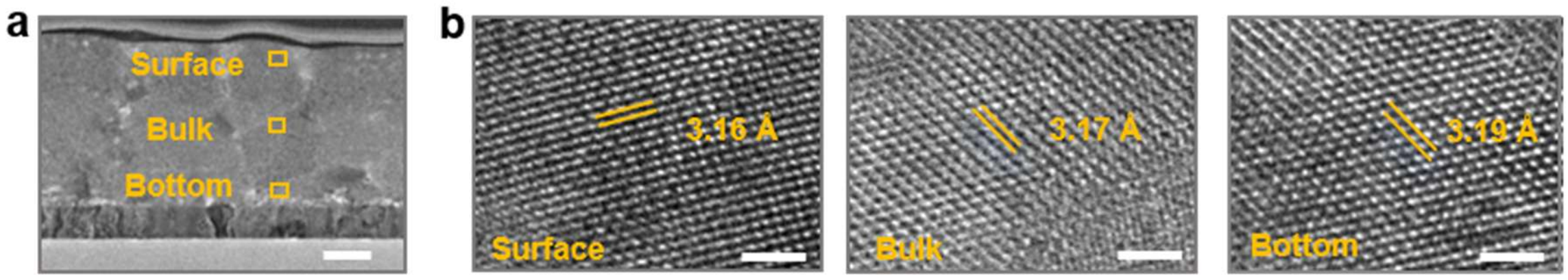

Supplementary Fig. 17 a) Cross-sectional SEM image of the control perovskite film. Scar bar, 200 nm. b) Lattice fringe images collected from the surface, the bulk, and the bottom regions indicated in a). Scar bar, 2 nm.

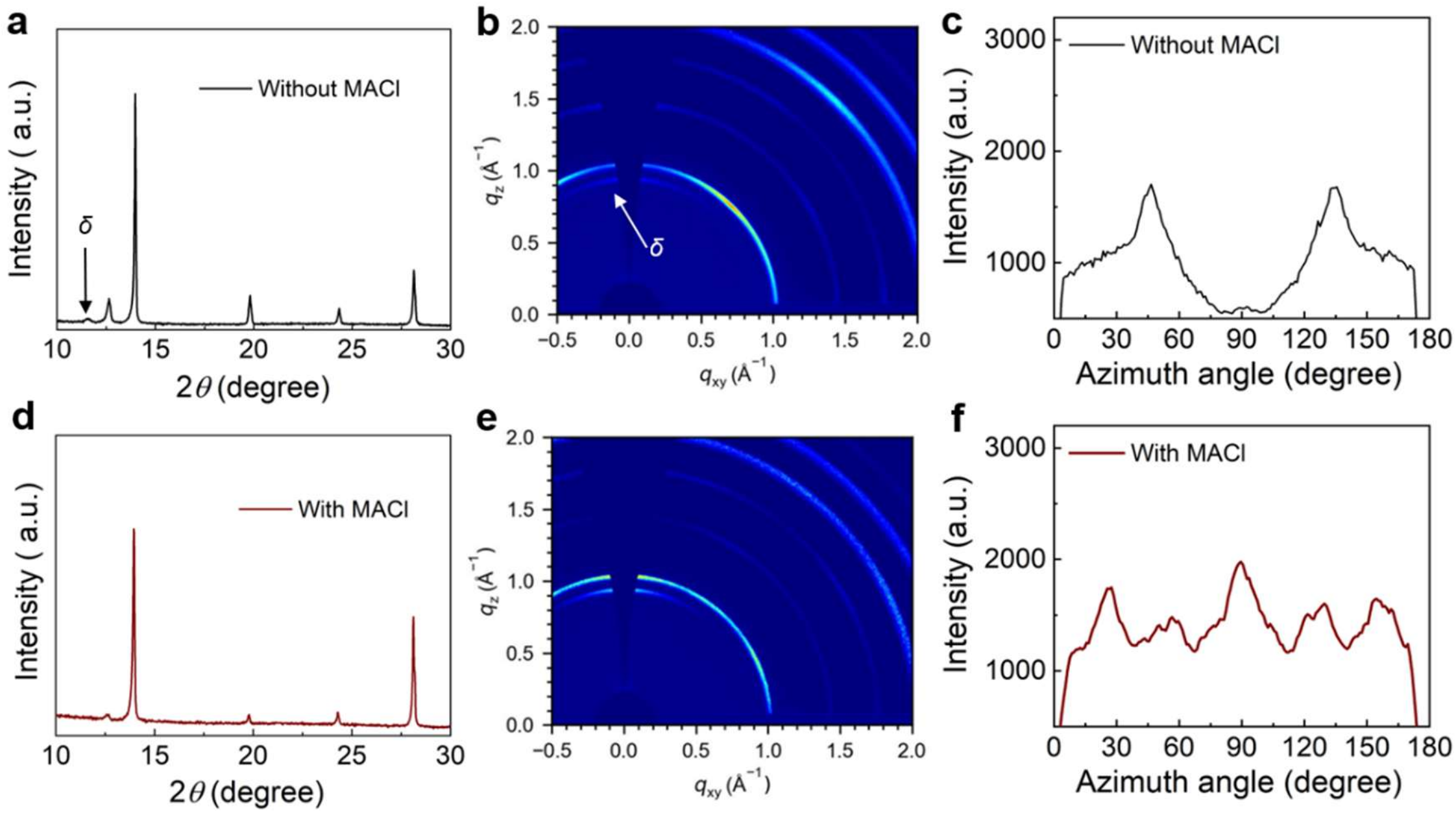

Supplementary Fig. 18 Structural characterization of perovskite films with/without MACl. a) XRD patterns of perovskite films prepared without MACl. b) GIWAXS patterns of perovskite films without MACl. c) Azimuthally integrated intensity profiles along the ring at q_{xy} = 10 nm^{-1} of perovskite films prepared without MACl. d) XRD patterns of perovskite films prepared with MACl added at an amount equivalent to that introduced by FPGC. e) GIWAXS patterns of perovskite films prepared with MACl added at an amount equivalent to that introduced by FPGC. f) Integrated intensity plots azimuthally along the ring at q_{xy} = 10 nm^{-1} of perovskite films prepared with MACl added at an amount equivalent to that introduced by FPGC.

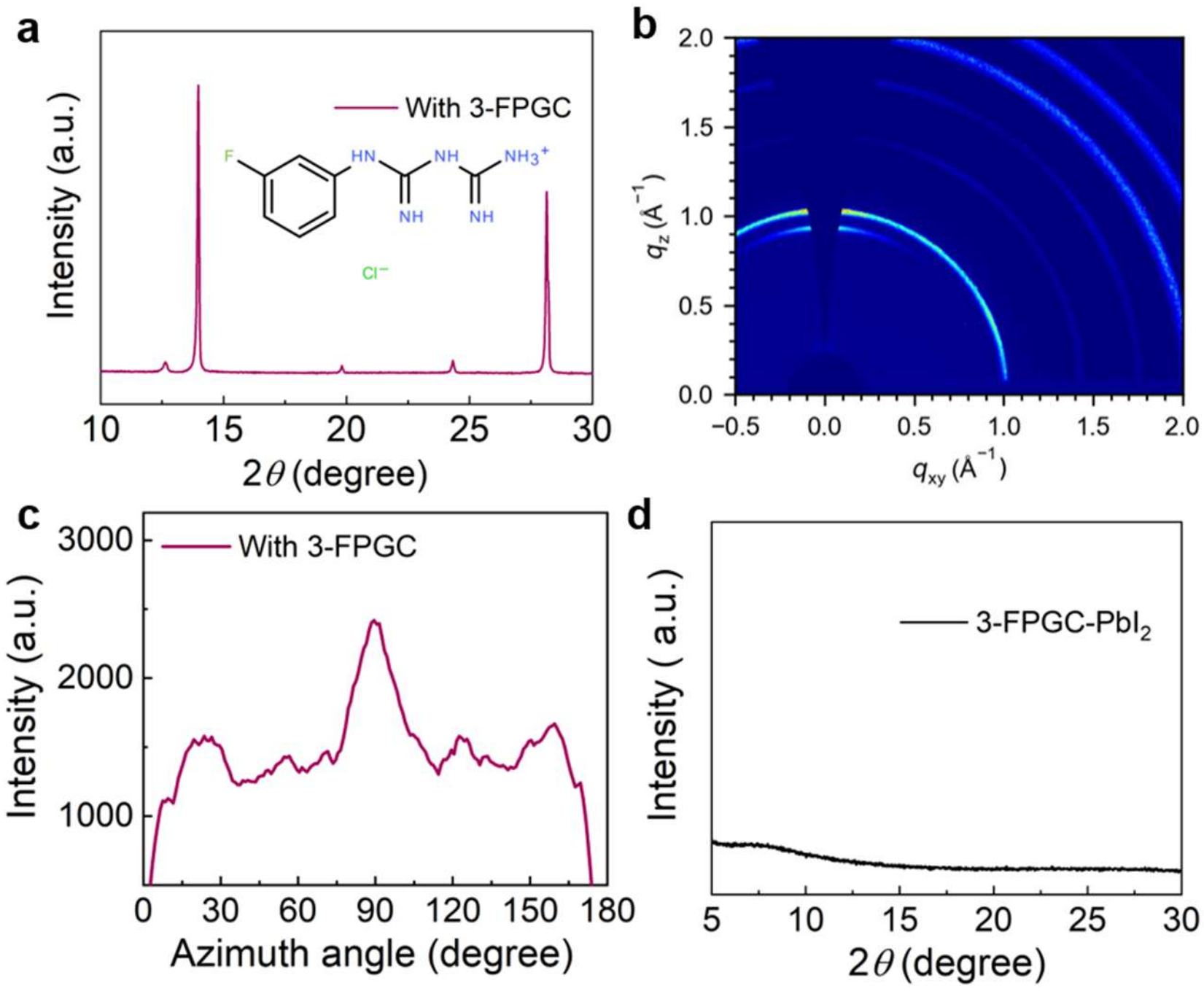

Supplementary Fig. 19 Structural-analogue control using 1-(3-fluorophenyl)biguanide hydrochloride (3-FPGC). a) XRD patterns of perovskite films prepared with the 3-FPGC. The inset shows the molecular structure of 3-FPGC. b) GIWAXS patterns of perovskite films prepared with the 3-FPGC. c) Integrated intensity plots azimuthally along the ring at $q_{xy} = 10\ nm^{-1}$. d) XRD pattern of the film prepared by spin-coating the 3-FPGC with PbI_2.

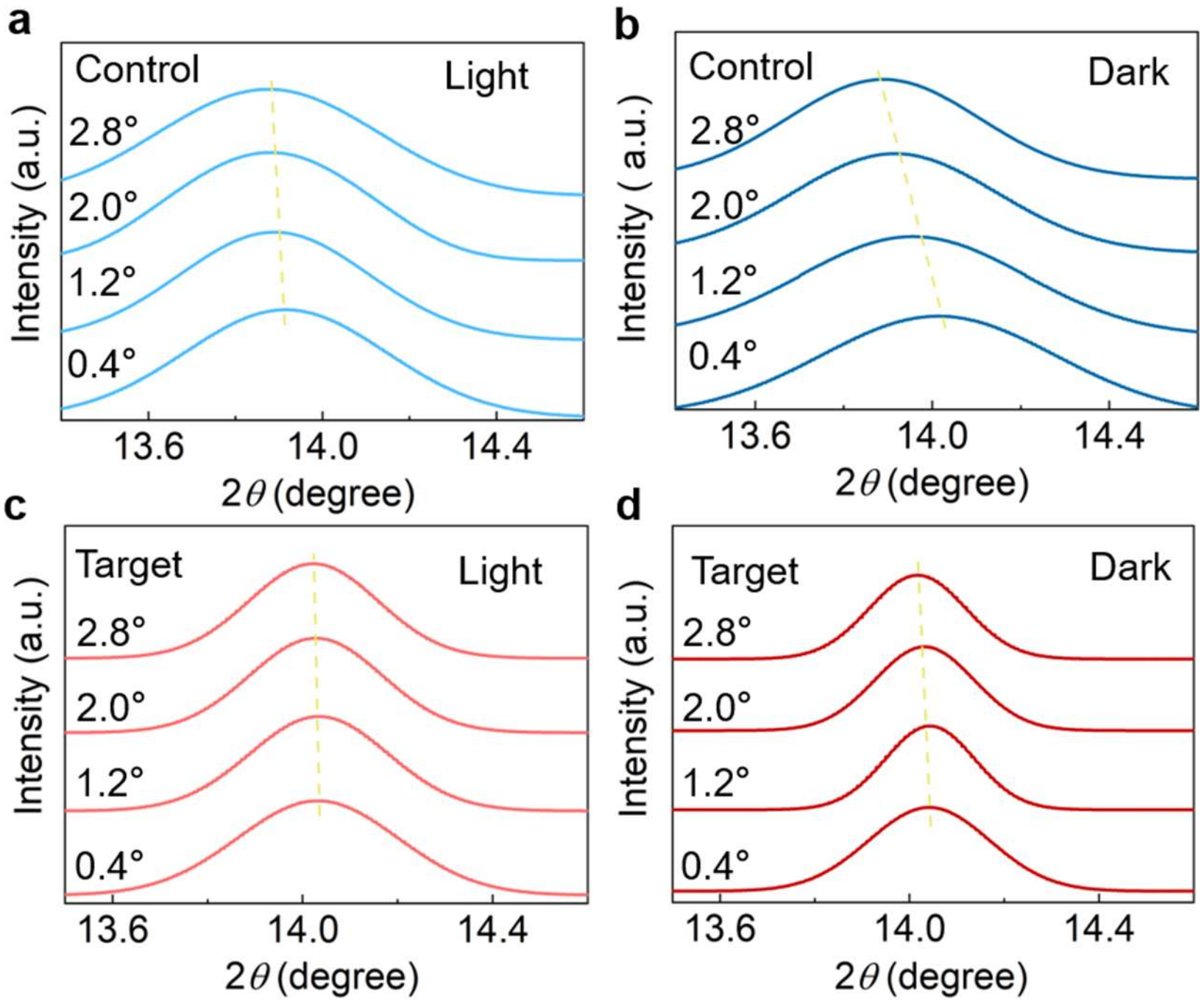

Supplementary Fig. 20 GIXRD of perovskite films under different states. a) GIXRD patterns of the control film first exposure to light for 70 minutes at ~ 55 °C. b) GIXRD patterns of the control film after transfer to dark conditions. c) GIXRD patterns of the target film first exposure to light for 70 minutes at ~ 55 °C. d) GIXRD patterns of the target film after transfer to dark conditions.

To investigate the spatial variations in structural dynamics, depth-dependent grazing-incidence X-ray diffraction (GIXRD) measurements were conducted to evaluate the vertical lattice response of the control and target films during light/dark cycling. The films were continuously illuminated for 70 min under simulated 1-sun intensity (100 mW cm^{-2}) at ~ 55 °C and subsequently stored in the dark for 5 h paired with cooling to room temperature. For the control film, continuous illumination with temperature increase induced pronounced diffraction peak shifts and localized line broadening across various incident angles, providing clear evidence of an inhomogeneous, depth-dependent lattice deformation and increased strain heterogeneity. The corresponding interplanar spacing was calculated using Bragg's law. The depth-dependent variation in *d*-spacing (Δd) evolved from 0.0635 Å in the pristine state to 0.0182 Å after light exposure, and partially recovered to 0.0566 Å after dark storage. This cyclical evolution demonstrates that the control film undergoes significant light- and heat-induced

structural distortion followed by only partial lattice relaxation in the dark. In sharp contrast, the target film exhibited negligible variations in both peak positions and Δd values under identical cycling protocols. This structural stability demonstrates that the enhanced, uniform crystallographic orientation across the entire film depth effectively suppresses light- and heat-induced lattice perturbations and eliminates vertical strain redistribution.

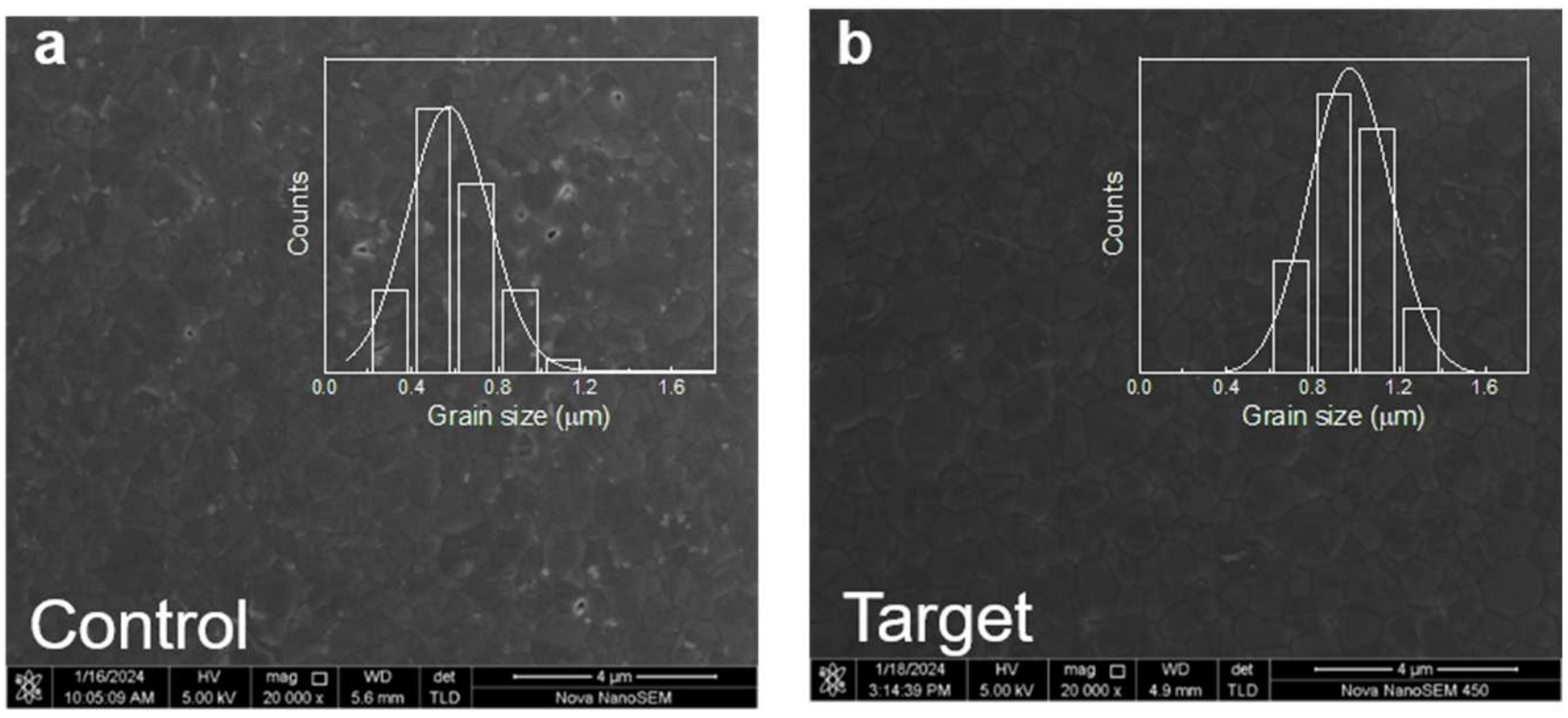

Supplementary Fig. 21 Top-view SEM images of a) the control perovskite film and b) the target perovskite film surface.

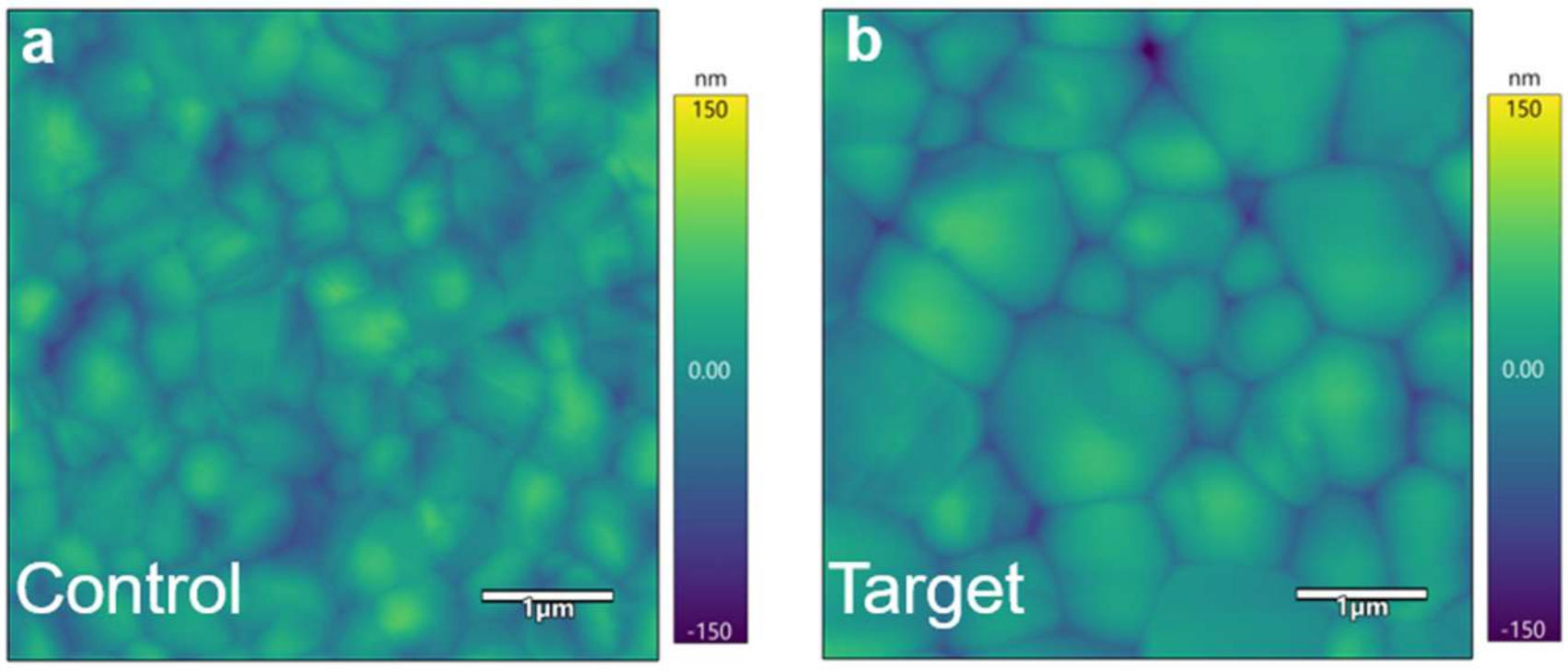

Supplementary Fig. 22 AFM images of top surface the a) control and b) target perovskite film.

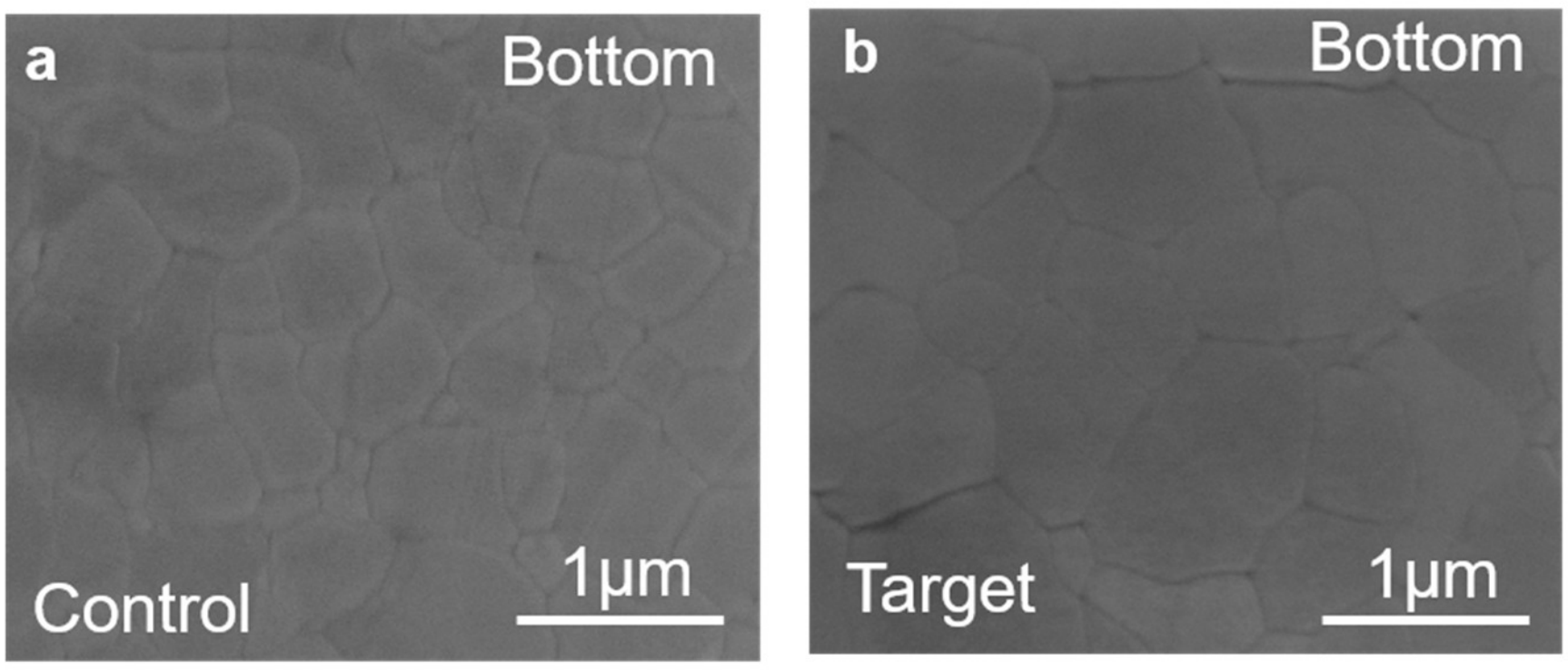

Supplementary Fig. 23 SEM images of bottom surface of the a) control and b) target perovskite film.

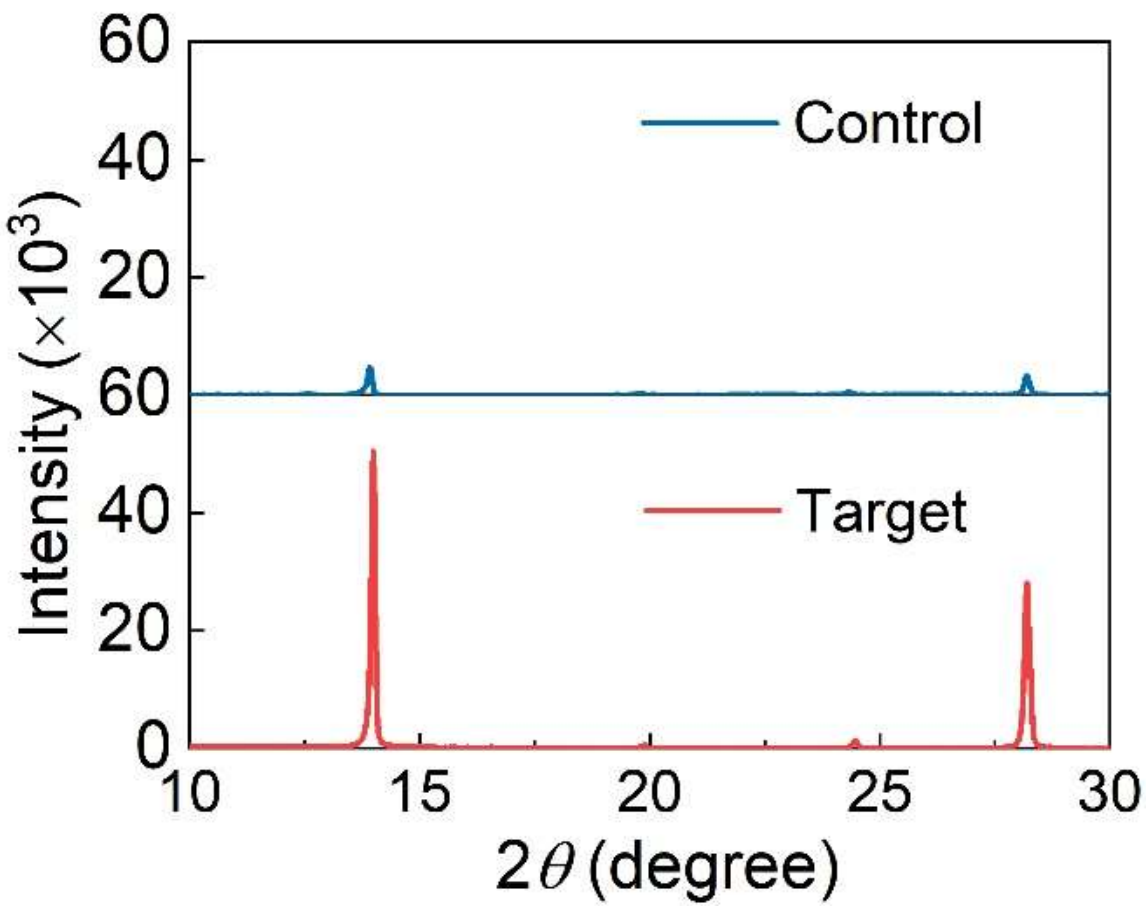

Supplementary Fig. 24 Comparison of XRD patterns of the control and target films from bottom surface.

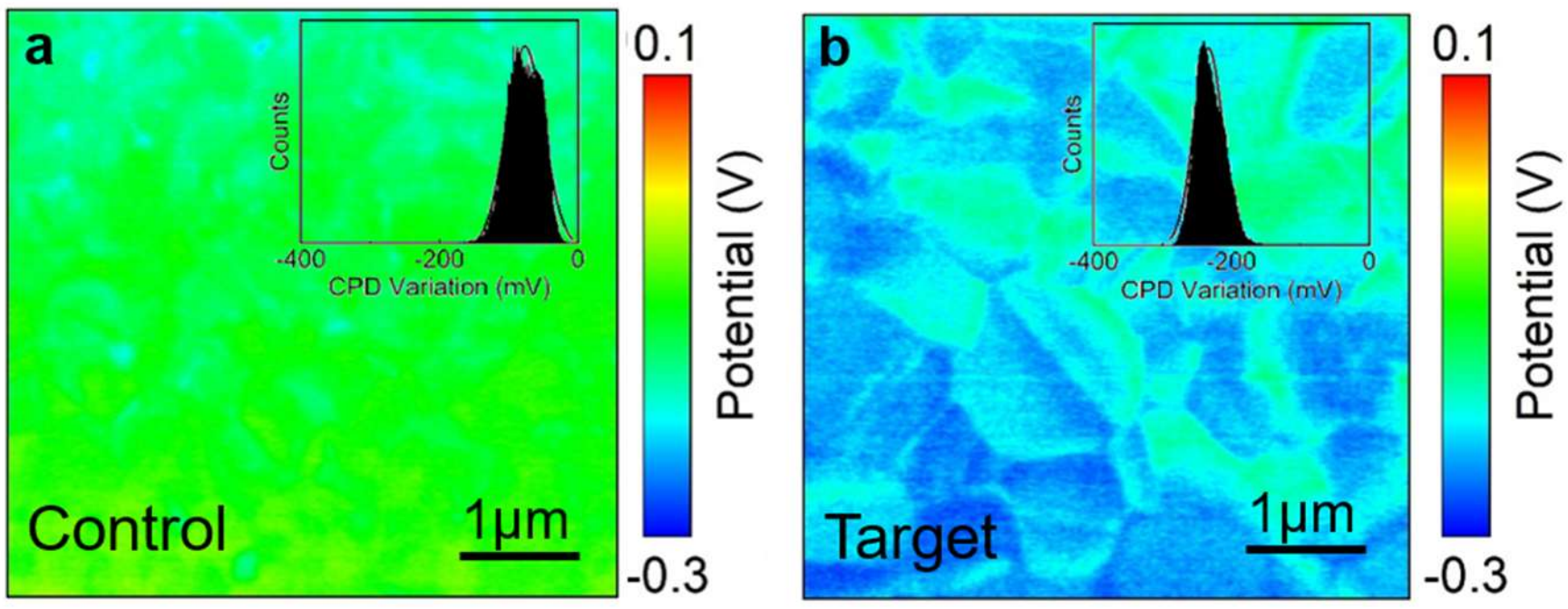

Supplementary Fig. 25 KPFM patterns of the a) control and b) target perovskite film.

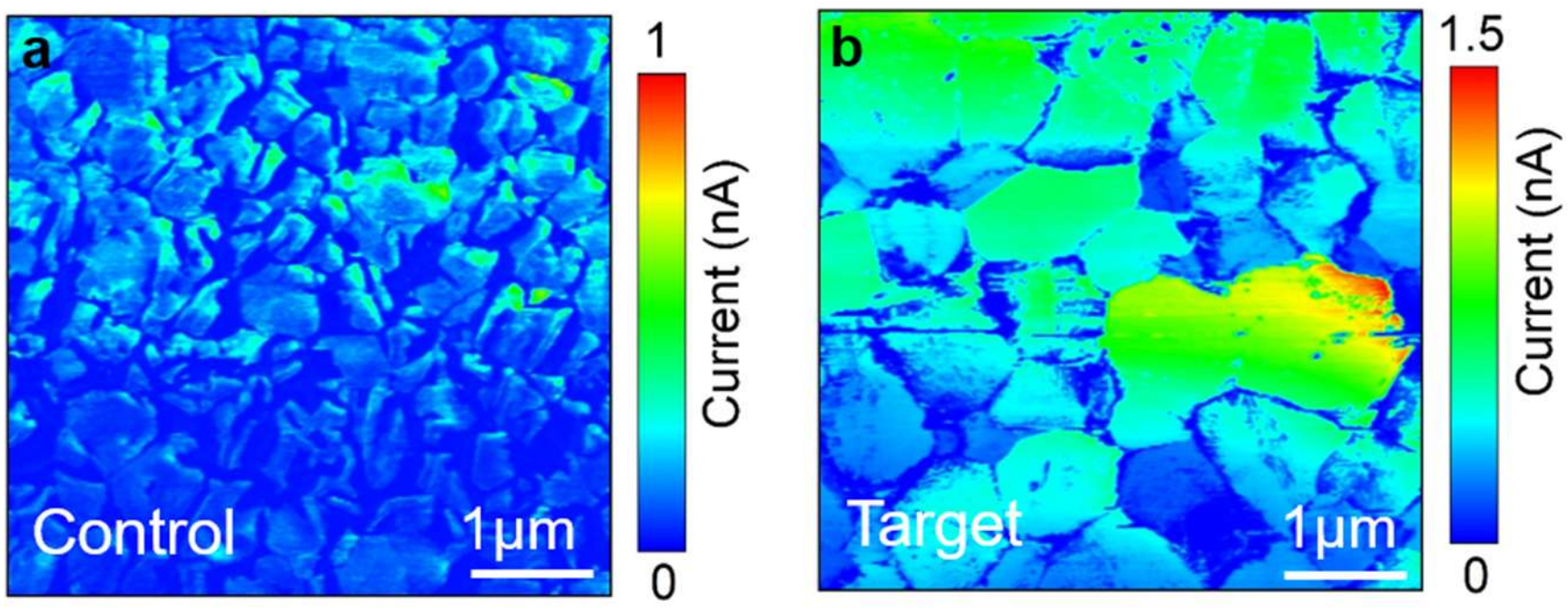

Supplementary Fig. 26 CAFM mapping for the a) control and b) target perovskite film.

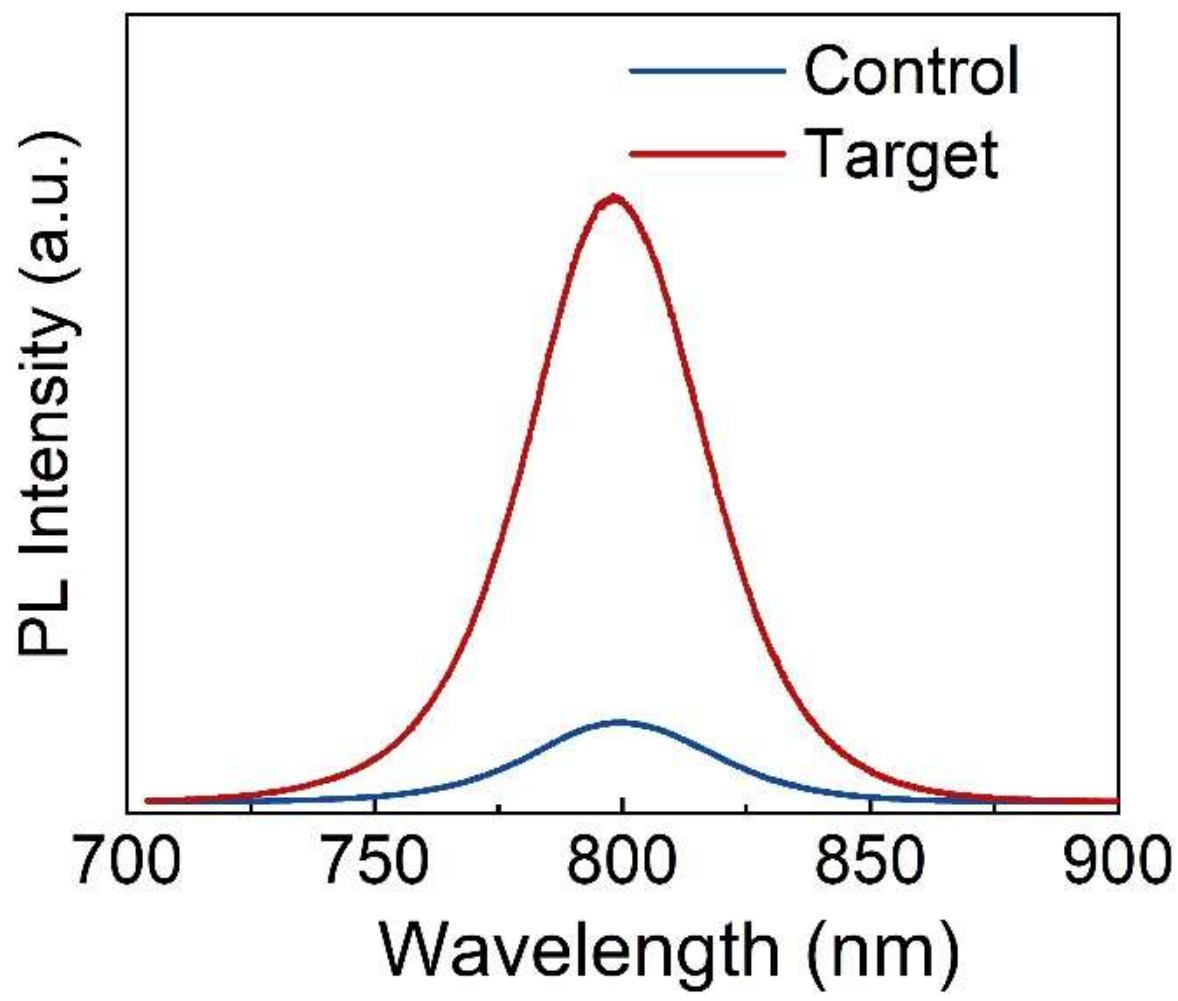

Supplementary Fig. 27 Steady-state photoluminescence (PL) spectra of perovskite films deposited on glass substrates.

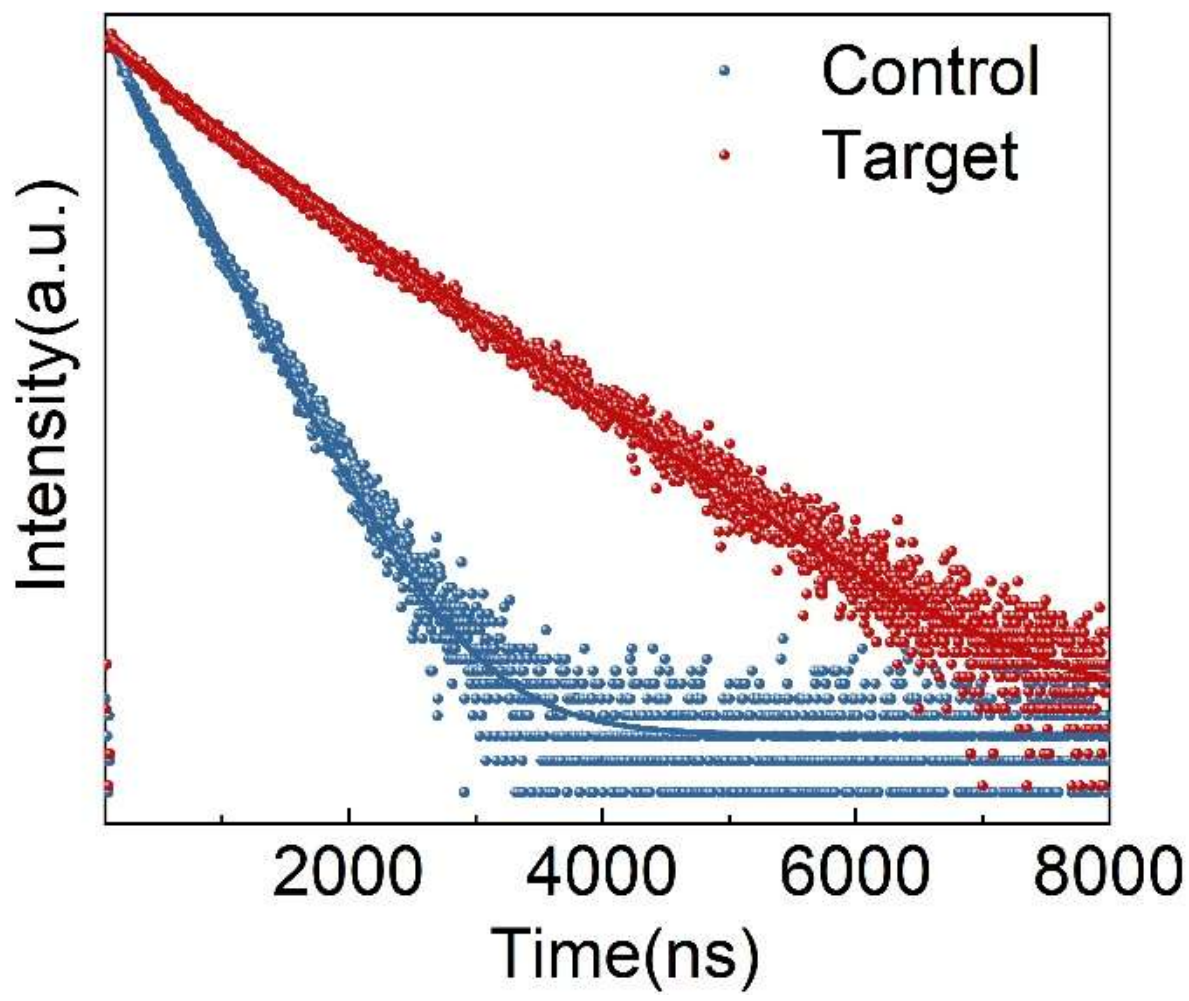

Supplementary Fig. 28 Time-resolved photoluminescence (TRPL) decays of perovskite films deposited on glass substrates. The carrier lifetime was extracted by fitting the decays with a single-exponential equation.

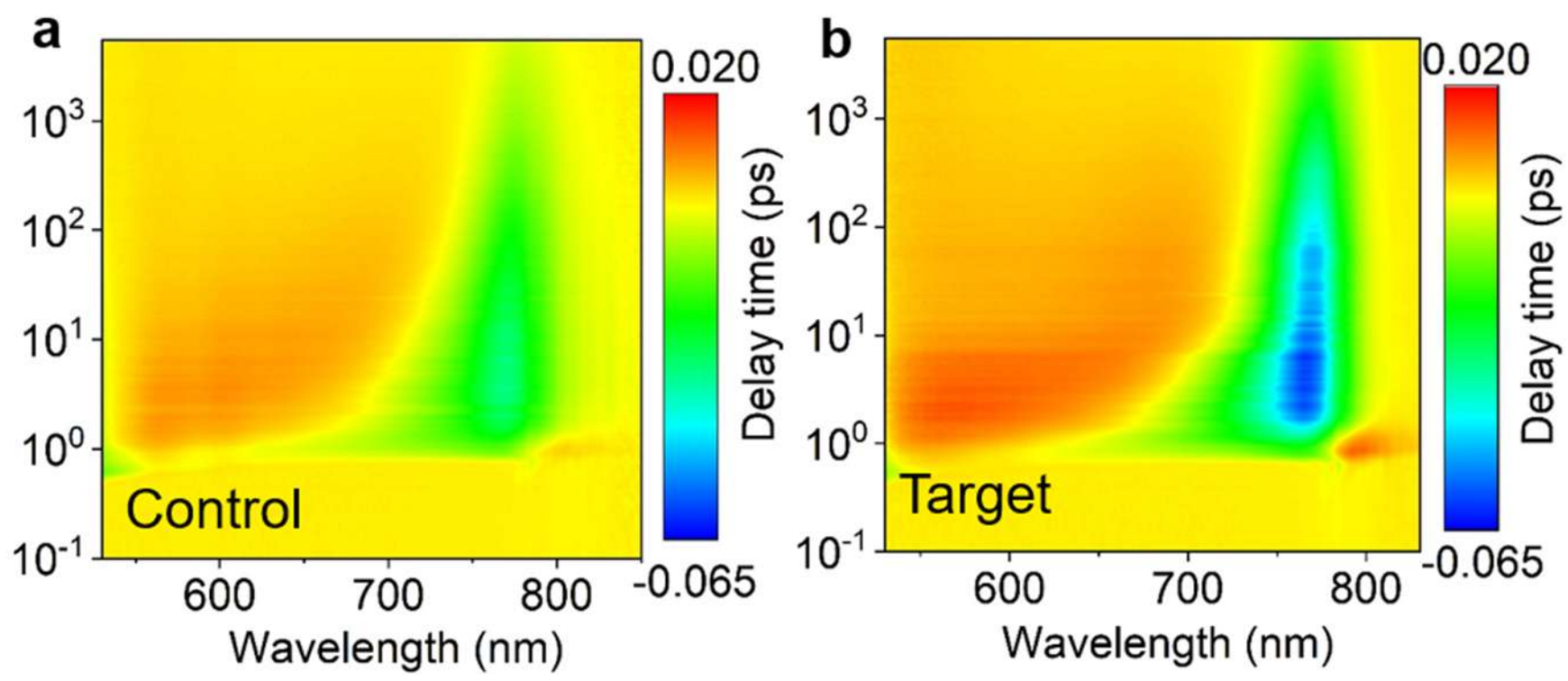

Supplementary Fig. 29 Transient absorption spectroscopy (TAS) images of the a) control and b) target perovskite film.

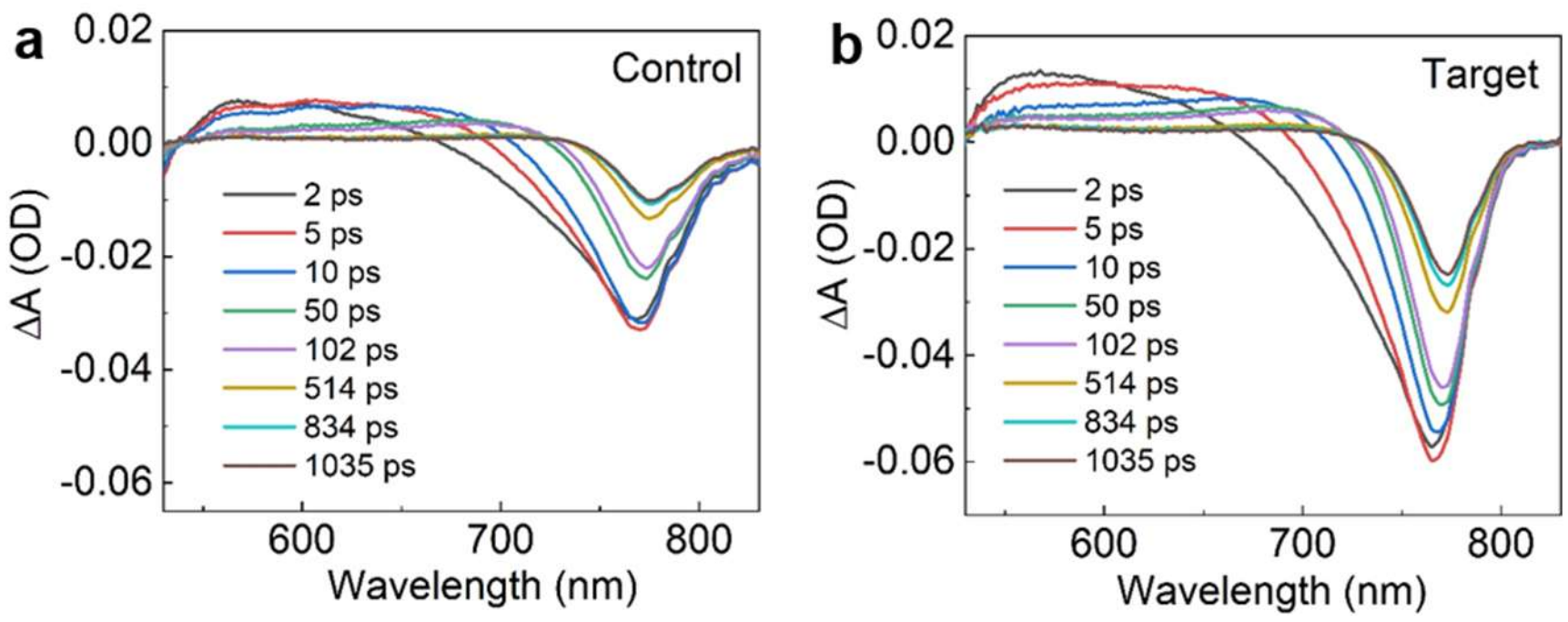

Supplementary Fig. 30 Transient absorption spectroscopy (TAS) at selected probe times of the perovskite films for a) control and b) target perovskite film.

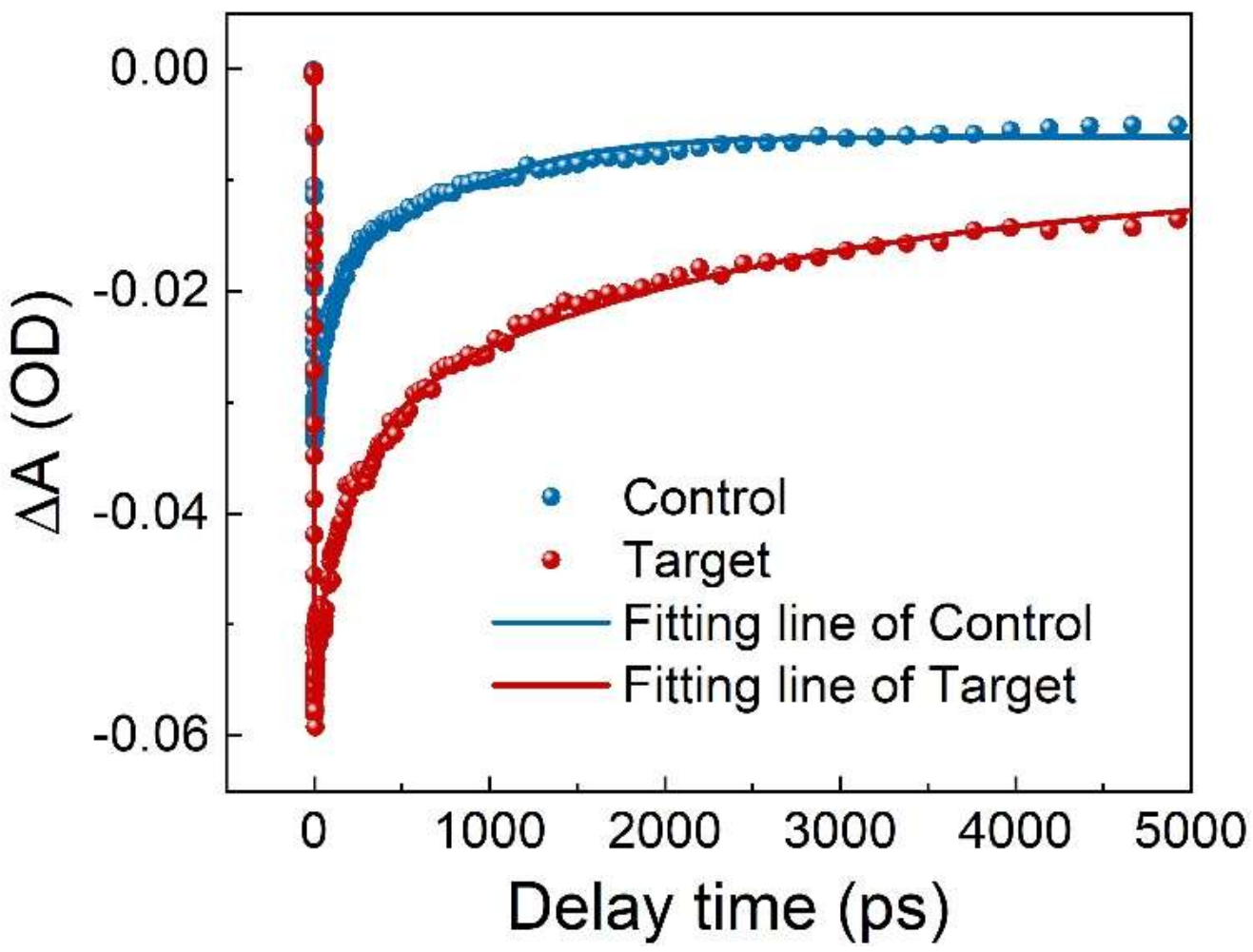

Supplementary Fig. 31 The bleach recovery kinetics from transient absorption spectroscopy characterization for the corresponding perovskite films following the excitation at probe wavelength.

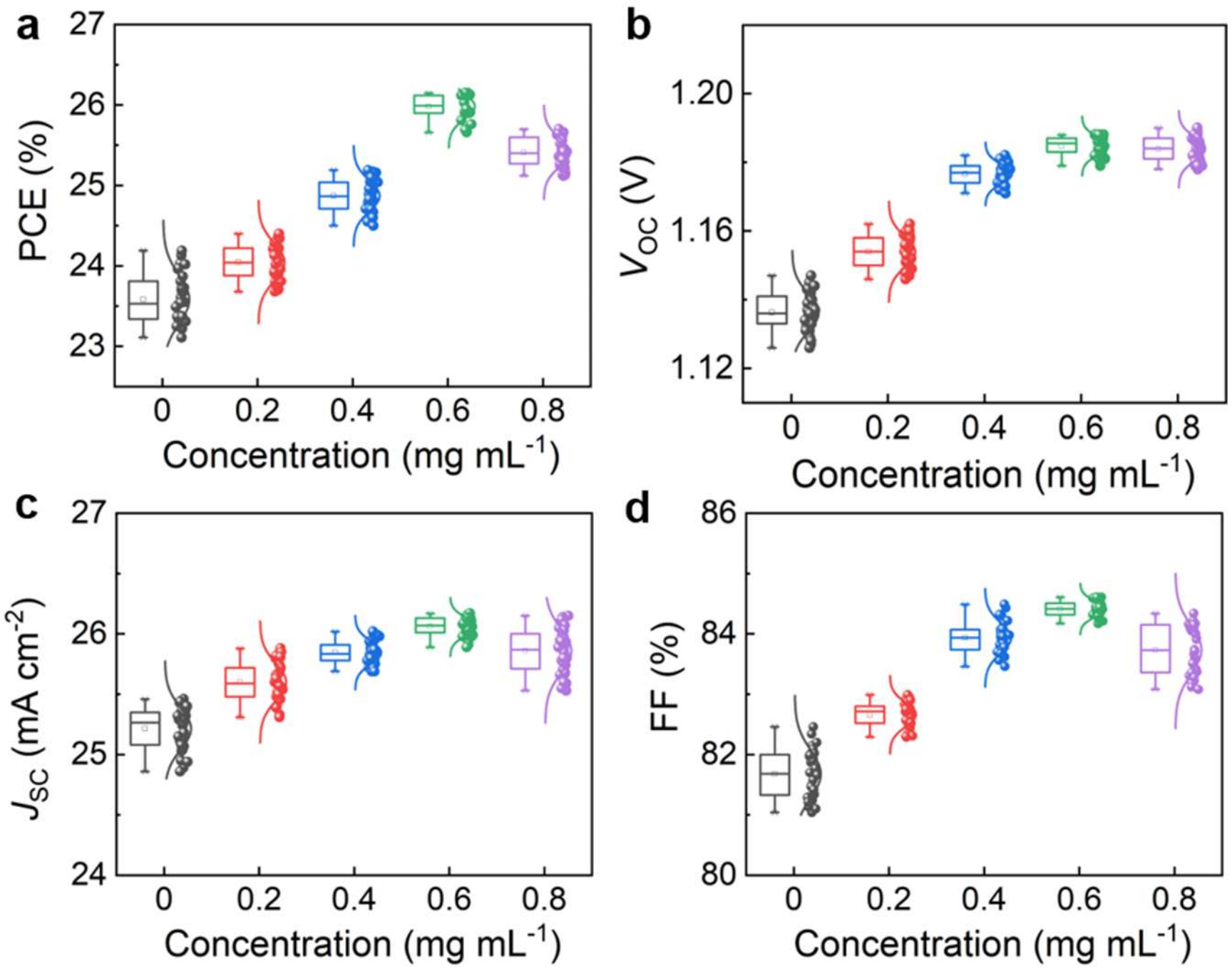

Supplementary Fig. 32 Photovoltaic parameters statistics of a) PCE, b) V_{OC}, c) J_{SC}, and d) FF based on 30 devices with different concentration of FPGC. The central line of the box plot represents the median, the box length indicates data dispersion and the whiskers denote the maximum and minimum values.

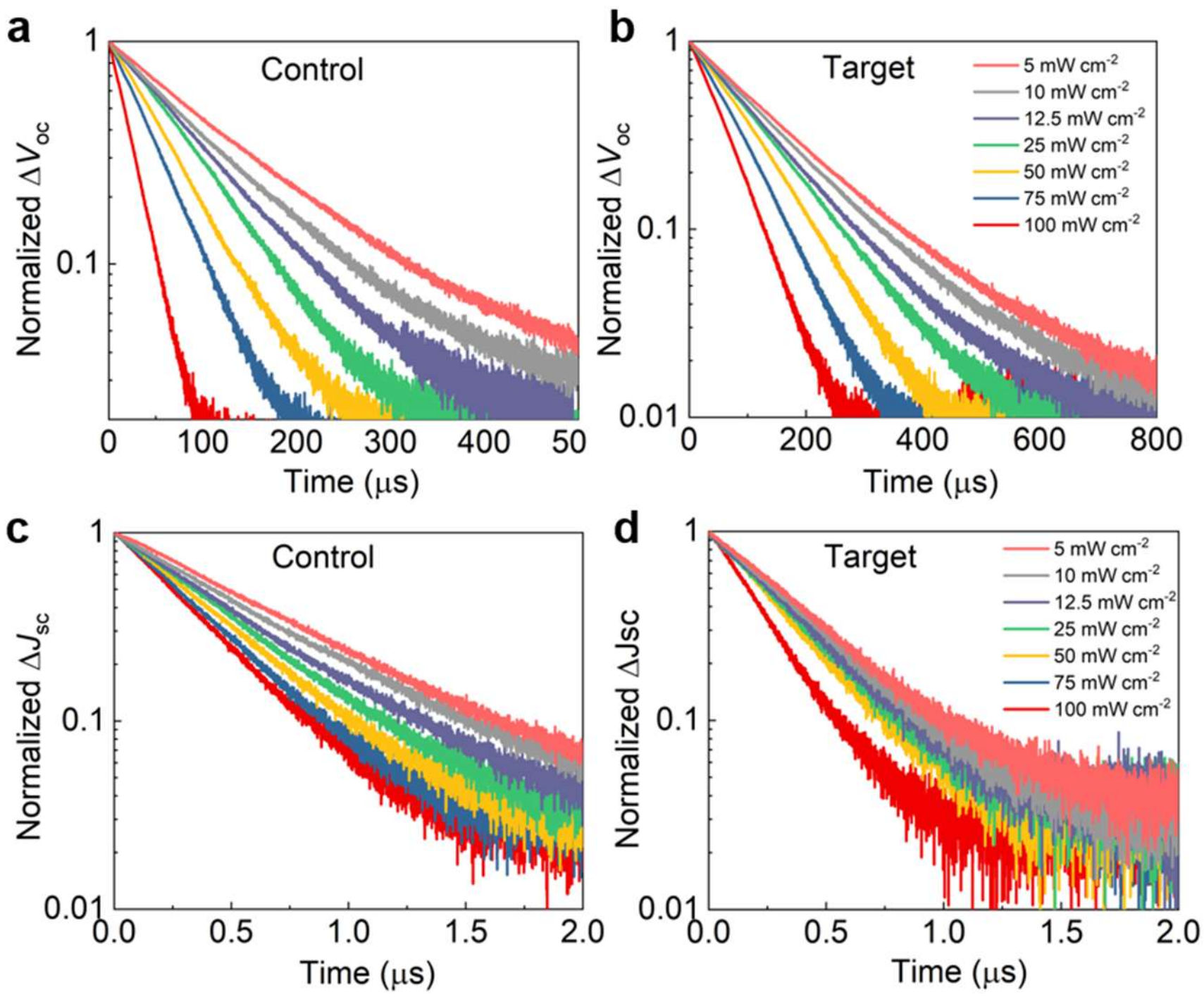

Supplementary Fig. 33 a), b) Transient photovoltage decay, and c), d) transient photocurrent decay curves of the control and target devices under varying light intensities.

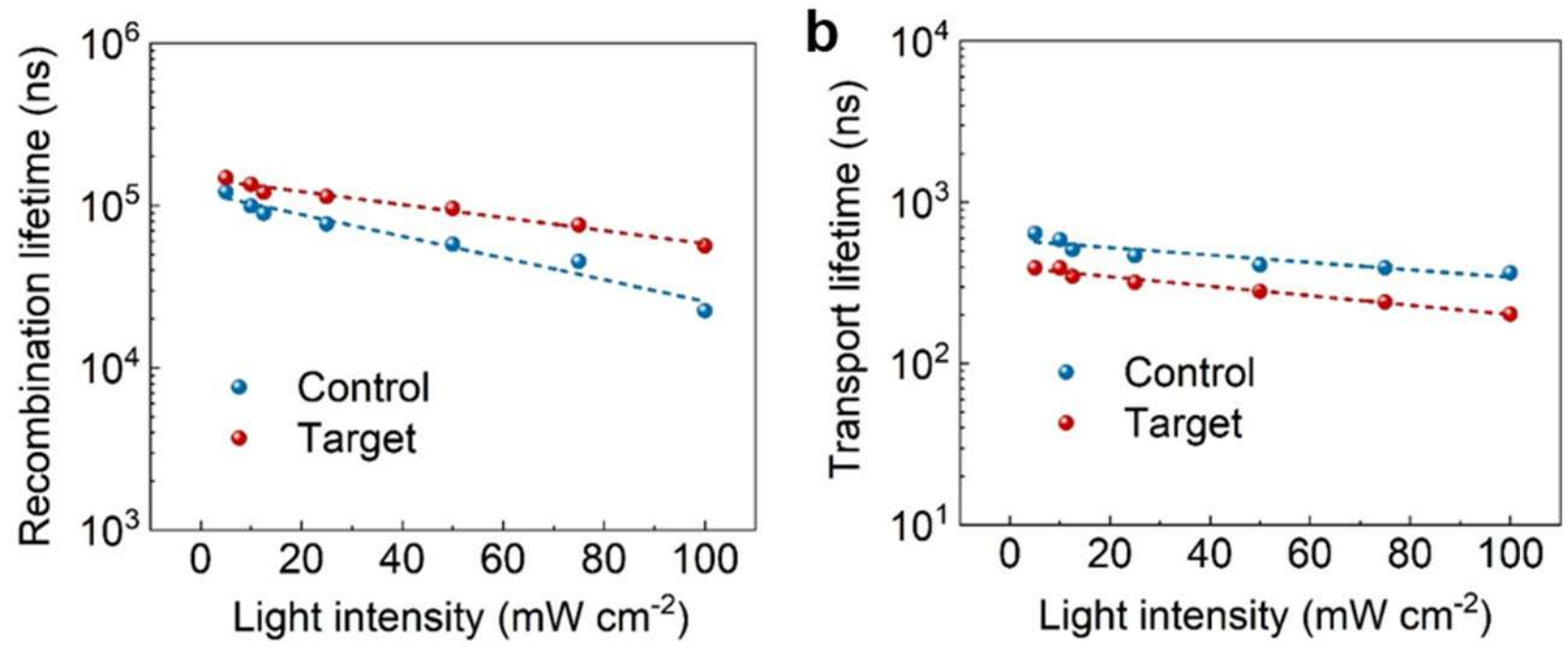

Supplementary Fig. 34 a) Charge recombination lifetime and b) transport lifetime of the control and target device.

Transient photocurrent (TPC) and transient photovoltage (TPV) decay measurements were conducted under varying light intensities to elucidate charge carrier transport and recombination dynamics within the photovoltaic devices. By fitting the TPV profiles recorded under open-circuit conditions (Supplementary Fig. 33a, b) and the TPC profiles recorded under short-circuit conditions (Supplementary Fig. 33c, d), the carrier recombination lifetime (τ_{re}) and transport lifetime (τ_{tr}) were extracted as functions of the incident light intensity for both the control and target devices (Supplementary Fig. 34). Across all evaluated light intensities, τ_{re} for the target device consistently exceeded that of the reference cell, whereas τ_{tr} for the control device remained systematically higher than that of the target device. This behavior demonstrates that the target devices possess a significantly suppressed charge recombination probability coupled with accelerated charge extraction.

The effective carrier diffusion coefficient (*D*) was evaluated using the following equation:

$$D = d^2/(c \times \tau_{tr}) \qquad (1)$$

where *d* is the active perovskite absorber thickness and <u>*c*</u> is a geometric proportionality constant established as 2.37. Under simulated 1-sun illumination, *D* increased from 3.42 × 10^{-3} cm^2 s^{-1} for the control film to 6.17 × 10^{-3} cm^2 s^{-1} for the target layer. Consequently, the calculated carrier diffusion length (LD, derived via $L_D = \sqrt{D \times \tau_{re}}$) increased substantially from 2275 nm to 5901 nm. These findings confirm that this

intermediate-mediated crystallization strategy effectively optimizes charge carrier collection efficiency within the perovskite solar cells.

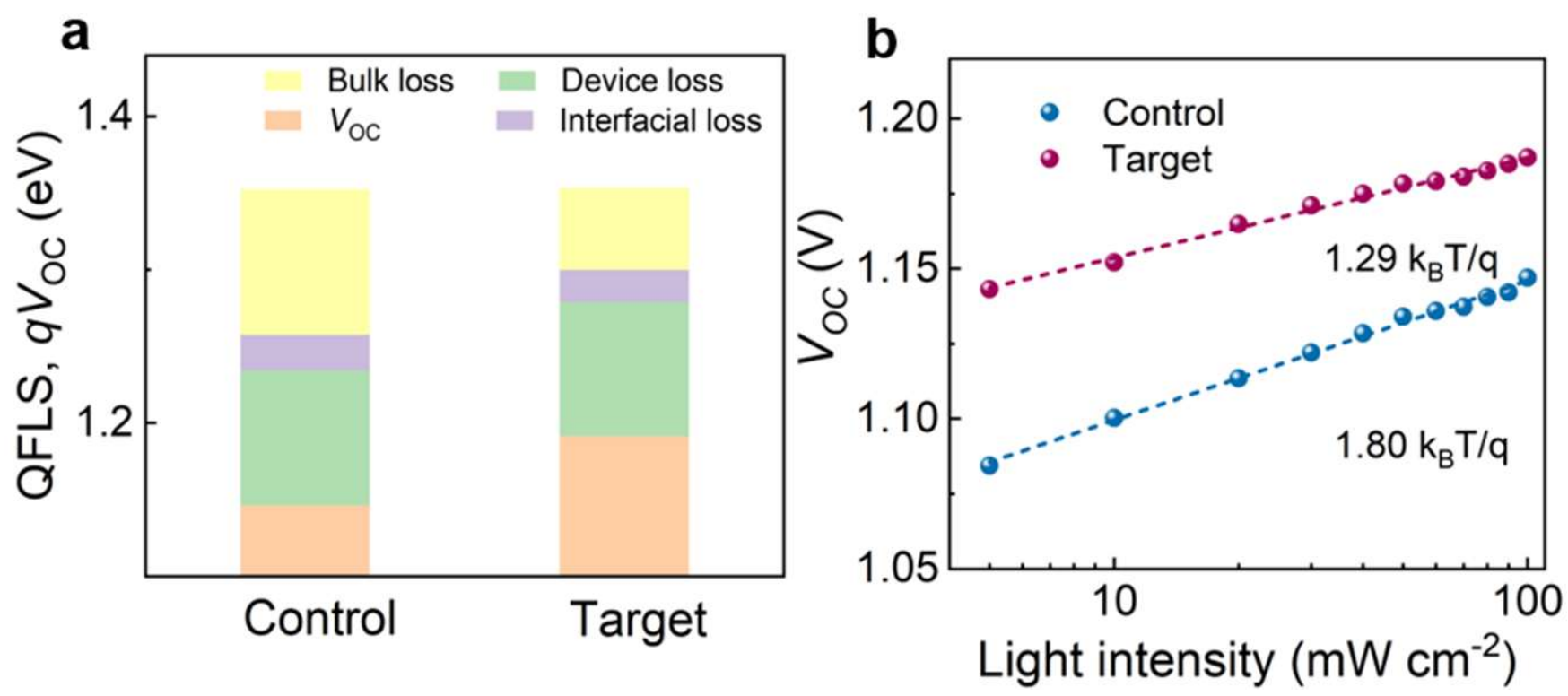

Supplementary Fig. 35 Mechanistic origin of the enhanced open-circuit voltage (V_{OC}) in the target device. a) Photoluminescence quantum yield (PLQY)-derived quasi-Fermi level splitting (ΔE_F or QFLS) values for the neat perovskite films and multi-layer device architectures. b) Light-intensity-dependent V_{OC} characteristics of the control and target devices.

To quantitatively elucidate the mechanism underlying the open-circuit voltage (V_{OC}) enhancement, photoluminescence quantum yield (PLQY) measurements were performed on the neat perovskite films and multi-layer device stacks under 1-sun-equivalent photoexcitation. These values were subsequently used to evaluate the quasi-Fermi level splitting (QFLS). To extract the QFLS values from the PLQY data, the dark radiative recombination current density ($J_{0,rad}$) of the device configuration must be established. The baseline flux of blackbody photons (ϕ_{BB}) was calculated according to the Planck radiation law:

$$\phi_{BB} = \frac{1}{4\pi^2\hbar^3c^2}\frac{E^2}{\exp\left(\frac{E}{k_BT}\right)-1} \quad (2)$$

where $\hbar$ is the reduced Planck constant, c is the speed of light in vacuum, E represents photon energy, k_B is the Boltzmann constant, and T denotes the absolute temperature. The value of $J_{0,rad}$ was then determined by integrating the blackbody photon flux with the experimental external quantum efficiency (EQE) spectrum:

$$J_{0,rad} = q\int EQE(E)\phi_{BB}(E)dE \quad (3)$$

where q represents the elementary electron charge. The thermodynamic radiative limit of the quasi-Fermi level splitting ($QFLS_{rad}$) was evaluated using the following relation:

$$QFLS_{rad} = k_B T \ln\left(\frac{J_G}{J_{0,rad}}\right) \quad (4)$$

where J_G represents the generation current density, which is approximated by the short-circuit current density (J_{SC}). Subsequently, the actual, non-ideal QFLS value under open-circuit conditions was calculated via:

$$\mathrm{QFLS} = QFLS_{rad} + k_B T \ln(PLQY) \quad (5)$$

The non-radiative voltage loss originating from the bulk perovskite film ($\Delta E_{nr,bulk}$) is defined as [5]:

$$\Delta E_{nr,bulk} = QFLS_{rad} - QFLS_{fi} \quad = -k_B T \ln\left(PLQY_{film}\right) \quad (6)$$

The non-radiative voltage loss associated with interfacial recombination (ΔE_{int}) across the contact boundaries is given by:

$$\Delta E_{int} = QFLS_{film} - QFLS_{stack} \quad (7)$$

Finally, the remaining non-radiative voltage loss occurring within the operational device layout (ΔE_{device}), often associated with non-ideality factors and resistive extraction boundaries, is defined as:

$$\Delta E_{devic} \quad = QFLS_{stack} - qV_{OC} \quad (8)$$

To elucidate the physical origin of the enhanced V_{OC}, we evaluated the photoluminescence quantum yield (PLQY) of the neat perovskite films and the corresponding multilayer device stacks (Supplementary Fig. 35a). The PLQY of the neat target film increases markedly from 2.63% (control) to 13.21%, corresponding to a substantial expansion in quasi-Fermi level splitting (QFLS) from ~ 1.258 to ~ 1.301 eV. This substantial enhancement confirms a profound suppression of bulk non-radiative recombination within the target matrix. For the complete multilayer device stacks, the PLQY similarly rises from 1.06% to 5.82%, with the corresponding QFLS increasing from ~ 1.235 to ~ 1.280 eV. Detailed energetic loss analysis reveals that the bulk non-radiative loss decreases drastically from 94.0 to 52.5 meV, whereas the interfacial recombination loss decreases only marginally from 23.2 to 21.0 meV. These thermodynamic metrics demonstrate that the V_{OC} enhancement is primarily dominated by the mitigation of bulk defects. Consistently, the light-intensity-dependent V_{OC}

ideality factor slope decreases from 1.80 to 1.29 k_BT/q, further confirming the effective suppression of trap-assisted recombination pathways (Supplementary Fig. 35b). These results explain the improved V_{OC} and confirm the target strategy establishes a structurally coherent perovskite framework with a minimized trap density.

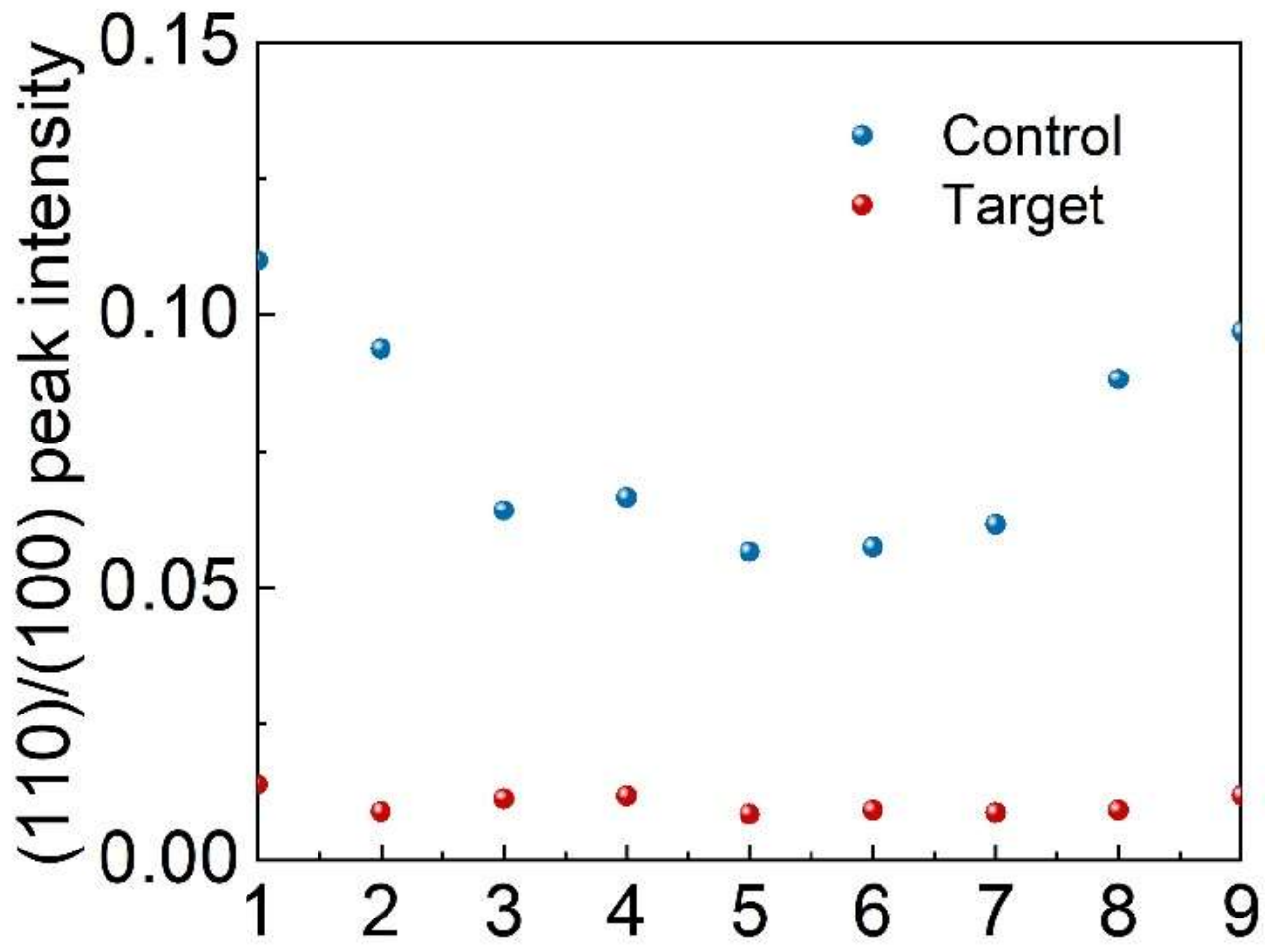

Supplementary Fig. 36 The peak intensity ratio of (100) and (110) facet of nine samples obtained by segmenting a 5 × 5 cm^2 control and target perovskite film.

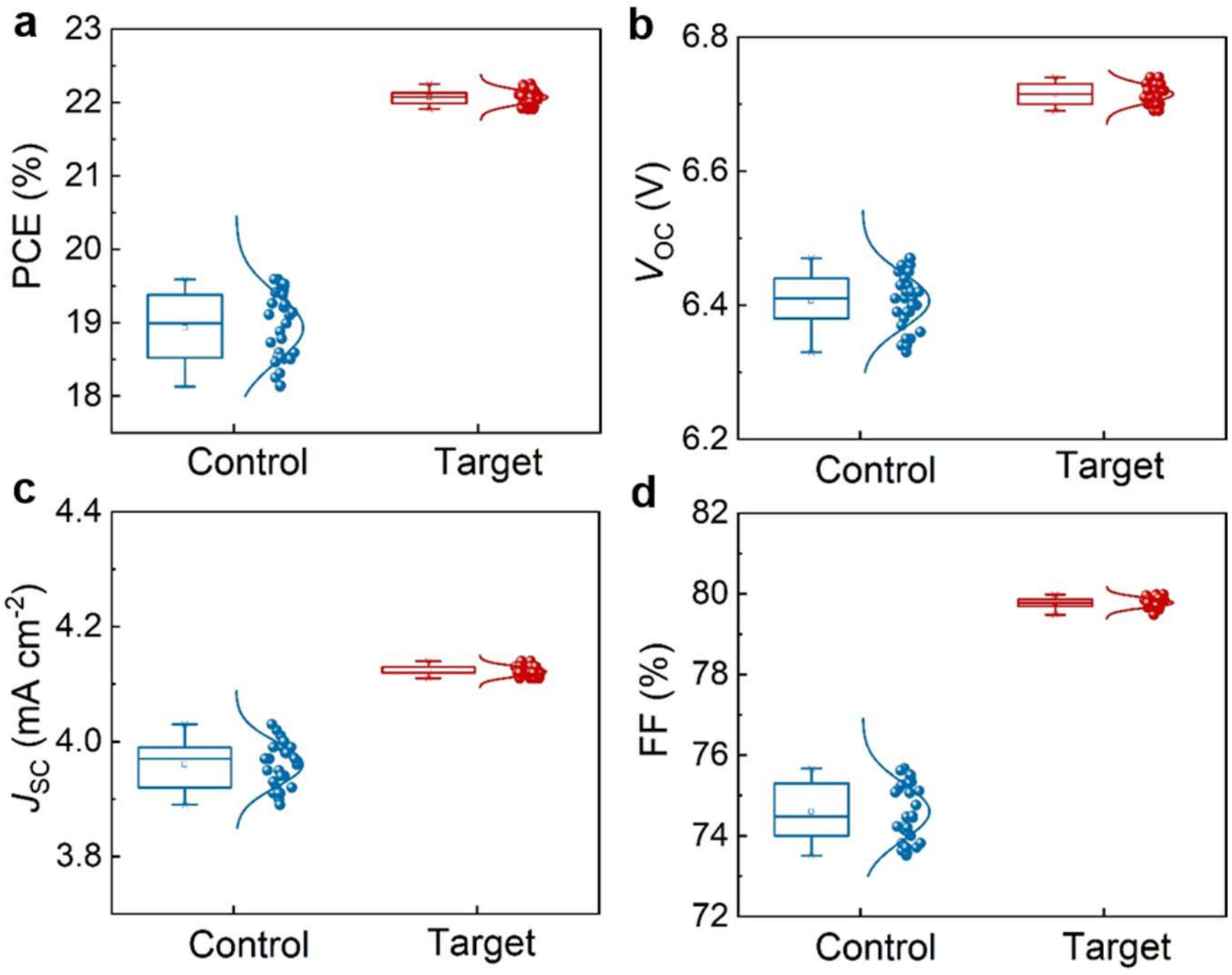

Supplementary Fig. 37 Photovoltaic parameters statistics of a) PCE, b) V_{OC}, c) J_{SC}, and d) FF based on 30 perovskite modules. The central line of the box plot represents the median, the box length indicates data dispersion and the whiskers denote the maximum and minimum values.

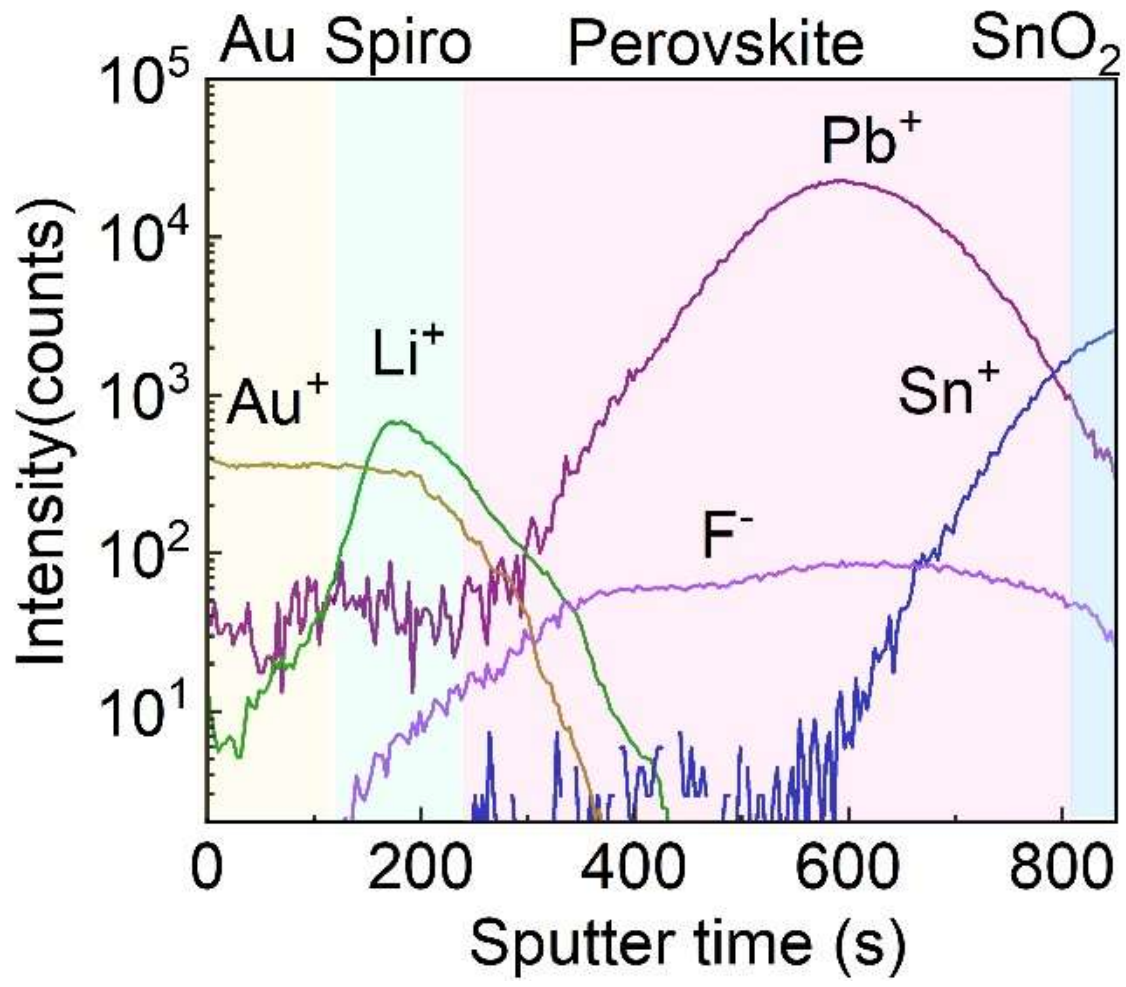

Supplementary Fig. 38 TOF-SIMS spectra of the target perovskite devices after 100 h of operation.

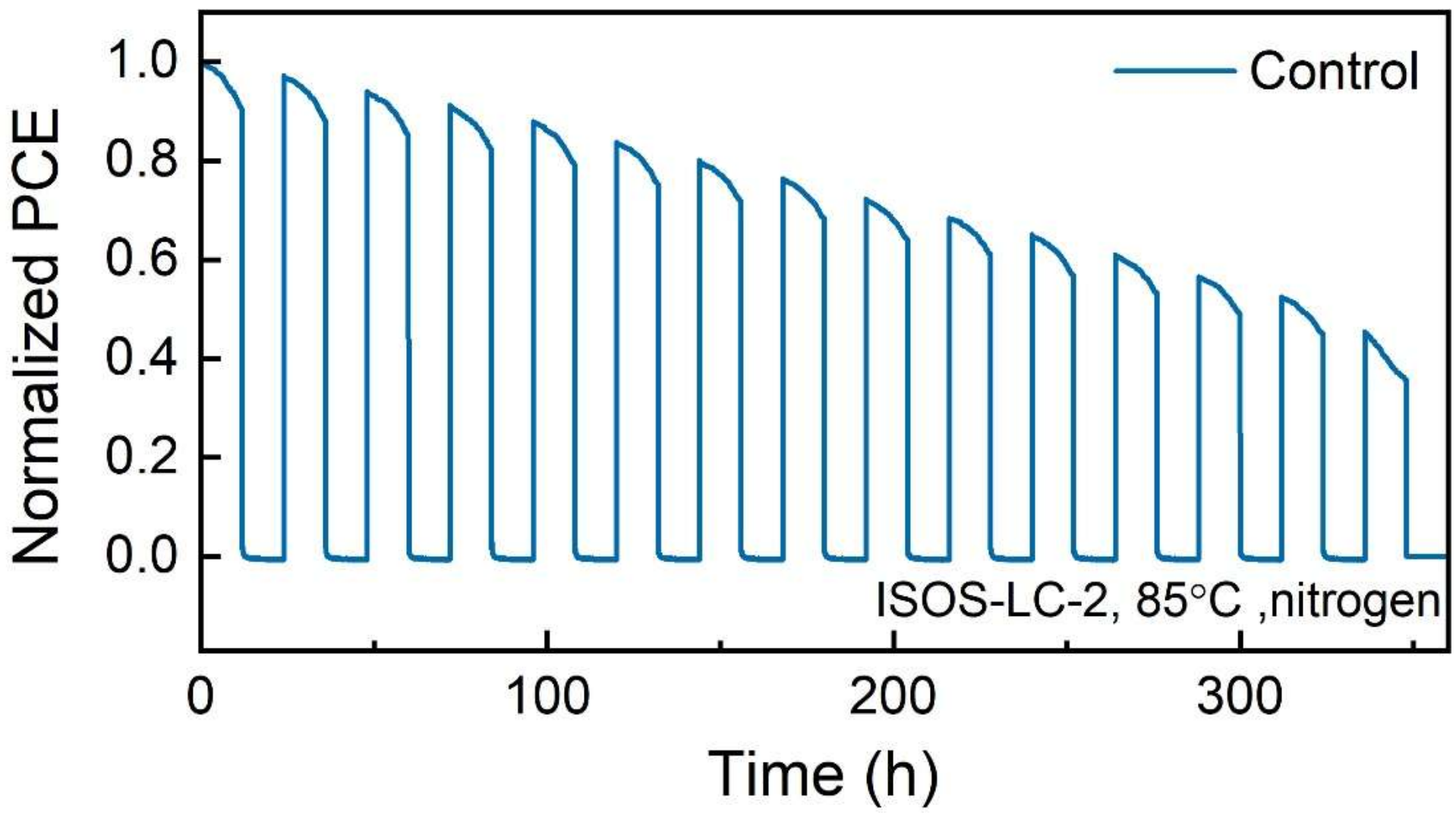

Supplementary Fig. 39 Normalized PCE of the pristine devices operating under 12-hour light-dark cycling at 85°C in nitrogen.

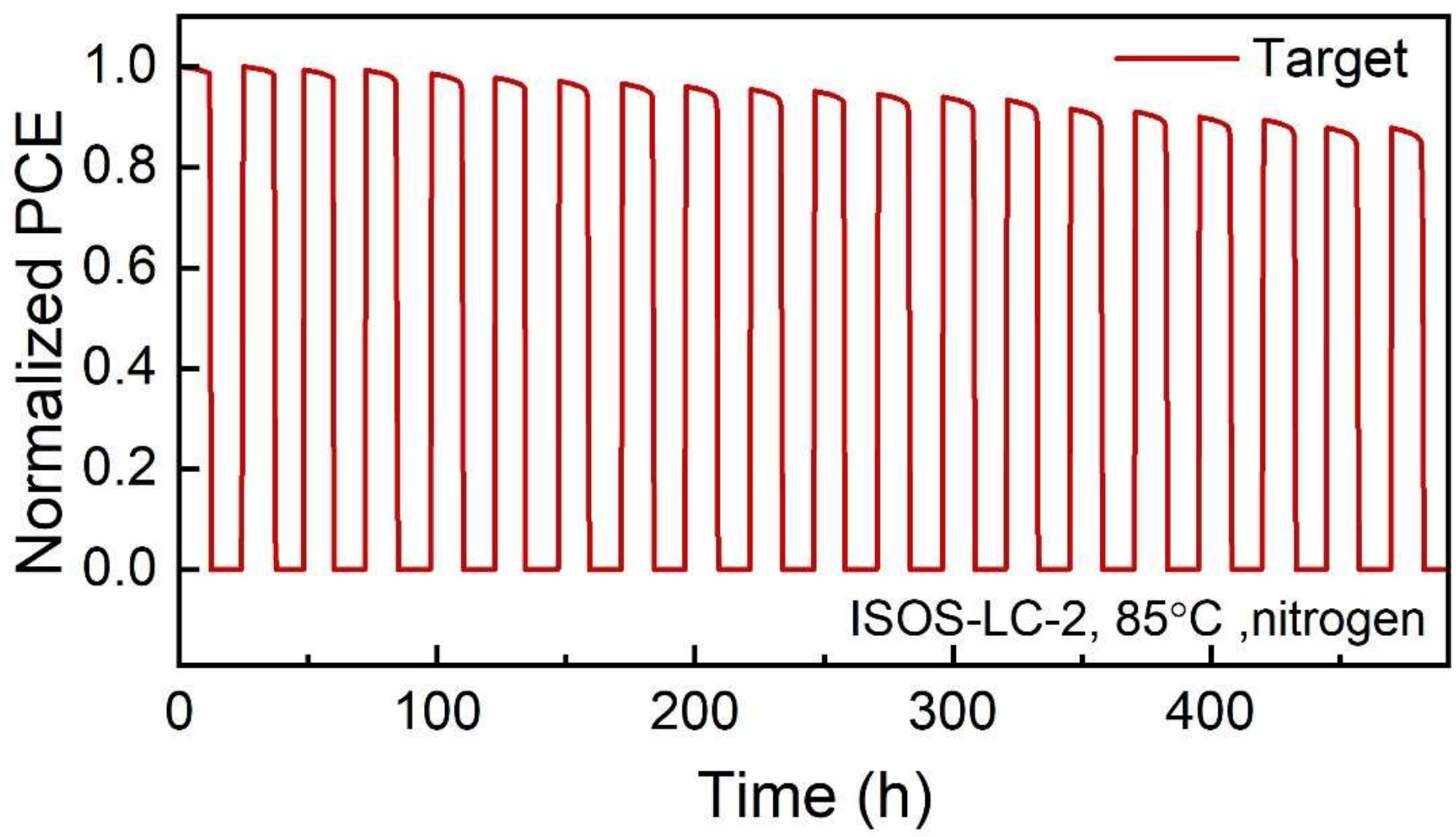

Supplementary Fig. 40 Normalized PCE of the target devices operating under 12-hour light-dark cycling at 85°C in nitrogen.

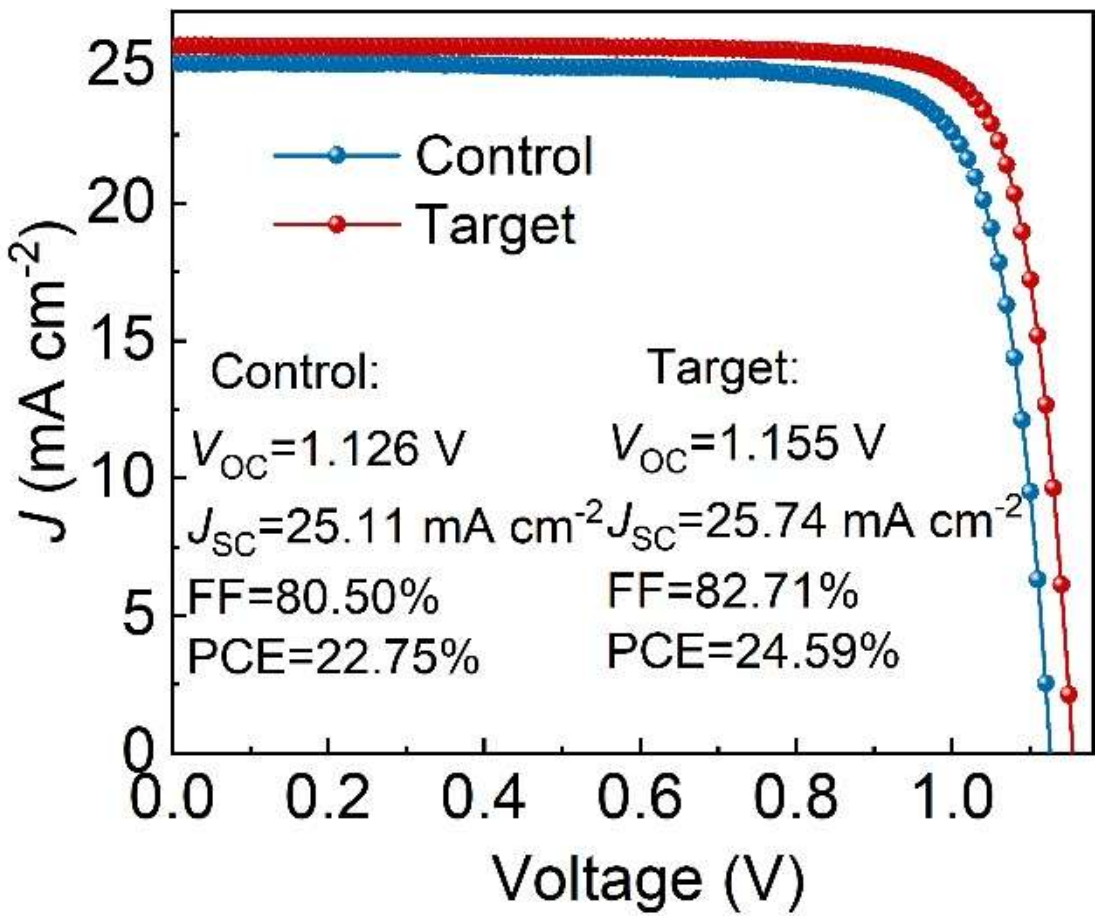

Supplementary Fig. 41 Initial *J*-*V* curve of the PSCs with P3HT as the hole transport layer.

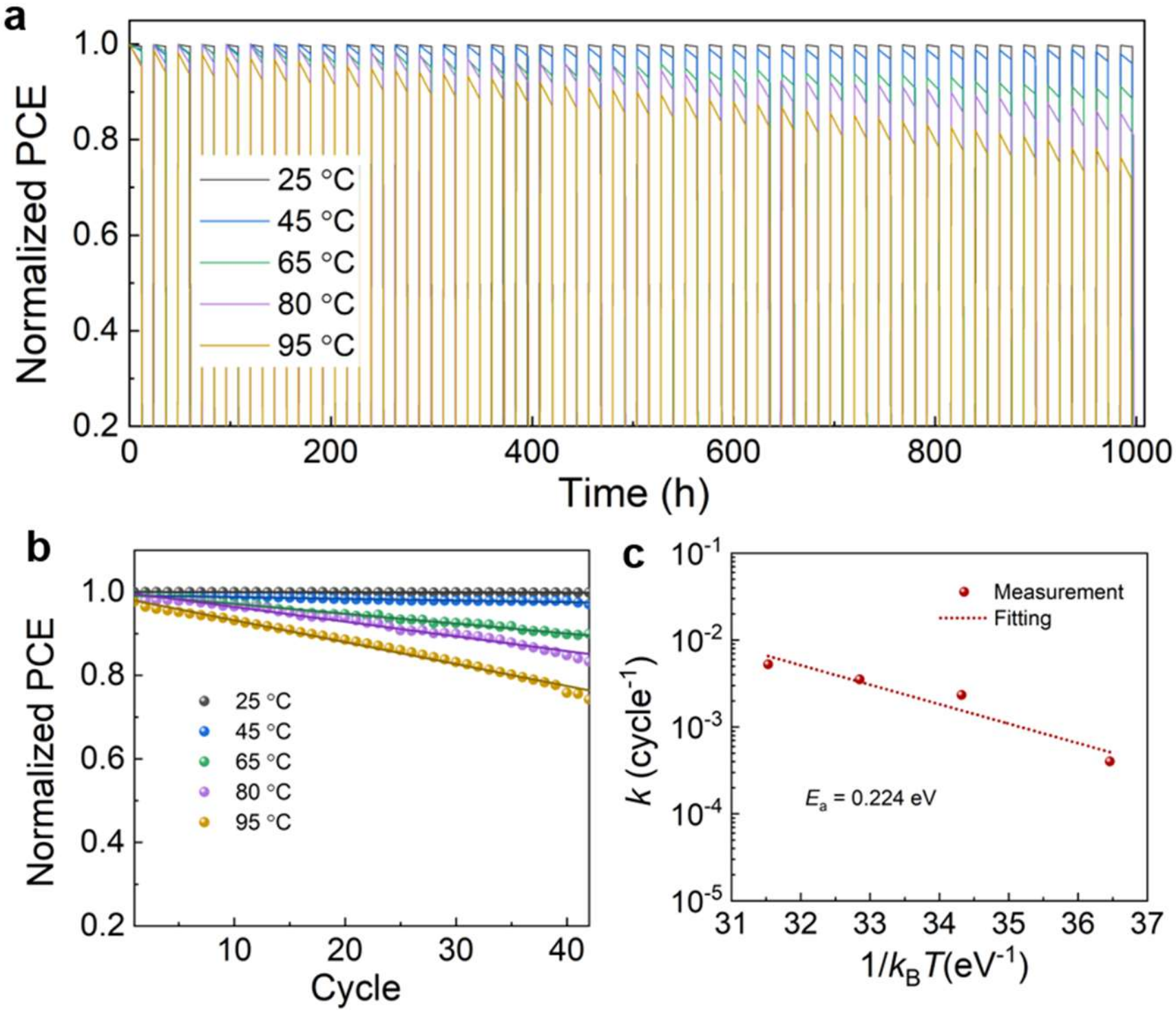

Supplementary Fig. 42 Accelerated ageing analysis under day-night cycling. a) Normalized PCE evolution of the devices under repeated light-dark cycling at 25, 45, 65, 80 and 95 °C. The degradation rate increases with temperature, whereas negligible degradation is observed at 25 °C within the measured time window. b) Normalized extracted PCE for each cycle operating under 12-hour light-dark cycling at temperatures of 25, 45, 65, 80 and 95 °C, respectively. (c) Arrhenius plot of the degradation rates extracted from the cycling data at 45-95 °C.

To quantify the thermal acceleration of device degradation under repeated light–dark transitions, accelerated aging tests were performed across a temperature series of 25, 45, 65, 80, and 95 °C. The normalized power conversion efficiency (PCE) decay profiles at each temperature step were fitted using a linear degradation function:

$$PCE(t) = 1 - kt \qquad (9)$$

where k represents the characteristic degradation rate coefficient and t denotes the cumulative cycle number.

Because negligible degradation occurred at 25 °C within the operational measurement window, only the degradation rates extracted between 45 and 95 °C were utilized for the Arrhenius analysis. The apparent degradation activation energy (E_a) was determined

from the temperature dependence of the degradation rate according to the Arrhenius relation:

$$E_a = -\frac{\partial In\left(k(T)\right)}{\partial\left(\frac{1}{k_B T}\right)} \quad (10)$$

where $k(T)$ is the temperature-dependent degradation rate, and k_B is the Boltzmann constant. Linear regression of the experimental data yielded an apparent degradation activation energy of 0.224 eV.

To accurately project device lifetime from the accelerated testing regimes to standard reference operating conditions, the thermal acceleration factor (AF) was evaluated as follows:

$$AF = \frac{k_{ace}}{k_{ref}} = exp\left[\frac{E_a}{k_B}\left(\frac{1}{T_{ref}} - \frac{1}{T_{ace}}\right)\right] \quad (11)$$

where T_{ace} and T_{ref} represent the absolute temperatures under the accelerated aging and reference operating conditions, respectively. In this evaluation, T_{ref} was defined as 55 °C (328.15 K) and T_{ref} was set to 95 °C (368.15 K).

Supplementary Table 1. The *d*-spacing value obtained from the XRD parameters.

	2θ (degree)	d-spacing (Å)
PbI_2	12.67	6.98
δ-$FAPbI_3$	11.81	7.49
α-$FAPbI_3$	13.95	6.34
$FPGC_2PbI_4$	14.18	6.24

Supplementary Table 2. Literature comparison of binding energies for ligand–perovskite interactions.

Complexes	Binding energy (eV)	Ref
DMF–PbI_2	-0.79	9
FA^+–PbI_3^-	-1.00	9
DMSO–PbI_2	-0.906	10
DMSO–PbI_2	-1.01	11
DMSO–PbI_2	-0.68	12
DMSO–PbI_2	-0.2	13
FA^+–PbI_2	-6.1	13
FA^+–PbI_3^-	-6.6	14

Supplementary Table 3. Photovoltaic parameters of the 16.8 cm^2 sized perovskite module.

Device	V_{OC} [V]	J_{SC} [mA cm^{-2}]	FF [%]	PCE [%]
Control	6.44	4.02	75.67	19.59
Target	6.72	4.14	79.98	22.25

Supplementary Note1: Synergistic roles of MACl and the $FPGC_2PbI_4$ transient intermediate in crystallization and orientation control

Recent studies have demonstrated that chloride-based additives are highly effective in regulating crystallization kinetics and enhancing perovskite thin-film quality [17]. Among these, methylammonium chloride (MACl) is widely utilized to lower the formation energy of the target perovskite phase, stabilize the photoactive black polymorph, and promote preferential crystallographic growth [18]. However, MACl incorporation alone is often insufficient to suppress all unfavorable intermediate pathways; furthermore, inhomogeneous chloride distribution or additive aggregation can induce severe compositional heterogeneity, defect formation, and vertical strain accumulation [19]. To address these limitations, multi-component additive engineering strategies have been explored. For instance, Ding et al. demonstrated that combining MACl with a Lewis-basic ionic liquid effectively suppresses MACl aggregation, thereby enhancing phase homogeneity, boosting crystallinity, and reducing defect density [20]. Similarly, Xiong et al. reported that the incorporation of oxalate-based alkali metal compounds can successfully homogenize the chlorine distribution within MACl-containing systems [21].

In this work, we introduced a two-dimensional (2D) perovskite intermediate into a methylammonium chloride (MACl)-containing precursor system. The resulting improvement in full-space crystallization homogeneity and the preferential out-of-plane growth along the (100) crystallographic planes arose from the synergistic effects of baseline MACl doping and FPGC incorporation. To elucidate whether these modifications originated simply from chloride-assisted crystallization or instead from the specific molecular role of FPGC, three distinct control experiments were conducted.

First, a baseline control was prepared using the standard precursor formulation without MACl or FPGC, while maintaining all other conditions identical to those established for the control films in the main text. X-ray diffraction (XRD) analysis revealed poor crystallinity for the α-phase along with residual δ-phase impurities (Supplementary Fig. 18a-c). This outcome confirms the essential role of MACl in promoting the δ-to-α phase

transition, which aligns with its established impact on precursor coordination and phase-transition kinetics [17].

Second, to test whether the function of FPGC could be replicated simply by increasing the total chloride concentration, an additional-chloride control was fabricated. The MACl content was increased such that the extra chloride molarity was comparable to that introduced by the FPGC ligand (Supplementary Fig. 18d-f). Although this control preserved the chloride-assisted chemistry inherent to the original precursor formulation, it failed to reproduce the pronounced preferential out-of-plane (100) orientation observed in the FPGC-containing films, nor did it induce discernible structural variations in the final thin film. This finding indicates that a minor increase in chloride content alone is insufficient to account for the orientation-control effect.

Third, a structural analogue, 1-(3-fluorophenyl)biguanide hydrochloride (3-FPGC), was examined. This molecule preserves the chloride-containing biguanide framework of FPGC but differs strictly in the positional isomerism of the fluorine substituent on the phenyl ring. When incorporated at an identical concentration to FPGC, 3-FPGC enhanced the intensity of the (100) diffraction peak, indicating some degree of influence on bulk crystallization (Supplementary Fig. 19a-c). However, grazing-incidence wide-angle X-ray scattering (GIWAXS) profiling did not show the pronounced out-of-plane (100) diffraction features characteristic of the FPGC-modified films, demonstrating that enhanced crystallinity alone is inadequate to generate the highly oriented vertical framework.

Furthermore, when 3-FPGC was mixed with PbI_2 and spin-coated under the identical processing conditions used for the FPGC reference system (1 M, 60 °C, 10 min), no distinct diffraction peaks were detected (Supplementary Fig. 19d). This lack of signal indicates that 3-FPGC does not form a crystalline intermediate phase analogous to $FPGC_2PbI_4$. In sharp contrast, the FPGC-containing system assembled a detectable, long-range-ordered crystalline intermediate that closely correlates with the subsequent development of the strongly oriented three-dimensional (3D) perovskite matrix. Taken

together, these control evaluations demonstrate that the observed orientation-control mechanism does not arise simply from baseline MACl or supplemental chloride, but is closely coupled with the specific molecular structure of FPGC and its unique capacity to form a transient crystalline intermediate that templated preferentially oriented 3D crystal growth.

Supplementary References

1. Dahod NS, Paritmongkol W, Stollmann A, Settens C, Zheng S-L, Tisdale WA. Melting transitions of the organic subphase in layered two-dimensional halide perovskites. *J. Phys. Chem. Lett.* **10**, 2924-2930 (2019).

2. Menahem M*, et al.* Strongly anharmonic octahedral tilting in two-dimensional hybrid halide perovskites. *ACS Nano* **15**, 10153-10162 (2021).

3. Sutanto AA*, et al.* In situ analysis reveals the role of 2D perovskite in preventing thermal-induced degradation in 2D/3D perovskite interfaces. *Nano Lett.* **20**, 3992-3998 (2020).

4. Perini CAR*, et al.* Interface reconstruction from Ruddlesden-Popper structures impacts stability in lead halide perovskite solar cells. *Adv. Mater.* **34**, 2204726 (2022).

5. Liu Z*, et al.* All-perovskite tandem solar cells achieving >29% efficiency with improved (100) orientation in wide-bandgap perovskites. *Nat. Mater.* **24**, 252-259 (2025).

6. Yang H, Guo H, Wang Y, Chen R, Tsang S-W, Cheng Y. In-situ photoluminescence for perovskite crystallization: bridging mechanistic insights and device engineering control. *Adv. Mater.*, e18643 (2026).

7. Zhou N*, et al.* Homologous molecule treatment in perovskite solar cells: synergistic management of holistic defect and charge transfer. *Adv. Funct. Mater.* **35**, 2418798 (2025).

8. He Q*, et al.* A self-assembled molecule directs ordered α-$FAPbI_3$ for n-i-p perovskite solar cells. *Nat. Commun.* **17**, 1479 (2026).

9. Li F*, et al.* Hydrogen-bond-bridged intermediate for perovskite solar cells with enhanced efficiency and stability. *Nat. Photonics* **17**, 478-484 (2023).

10. Liu J, Li M, Ji J, Zhao Z, Li M. Mild coordination enabled by steric hindrance facilitates fabrication of large-area perovskite solar modules. *Adv. Mater.* **38**, e21181 (2026).

11. Chen R*, et al.* Reduction of bulk and surface defects in inverted methylammonium- and bromide-free formamidinium perovskite solar cells. *Nat. Energy* **8**, 839-849 (2023).

12. Zhao X, *et al.* Regulation of buried interface through the rapid removal of $PbI_2 \cdot DMSO$ complex for enhancing light stability of perovskite solar cells. *ACS Energy Lett.* **9**, 2659-2669 (2024).
13. Pan T, *et al.* Surface-energy-regulated growth of α-phase $Cs_{0.03}FA_{0.97}PbI_3$ for highly efficient and stable inverted perovskite solar cells. *Adv. Mater.* **35**, 2208522 (2023).
14. Tian L, *et al.* Divalent cation replacement strategy stabilizes wide-bandgap perovskite for $Cu(In,Ga)Se_2$ tandem solar cells. *Nat. Photonics* **19**, 479-485 (2025).
15. Zhang J, *et al.* In situ dynamic regulation of strain at the buried interface of stable perovskite solar cells. *Nat. Photonics* **20**, 119-127 (2026).
16. Zhang S, *et al.* The role of bulk and interface recombination in high-efficiency low-dimensional perovskite solar cells. *Adv. Mater.* **31**, 1901090 (2019).
17. Liu X, Guo Y, Cheng Y, Lu S, Li R, Chen J. Advances in chloride additives for high-efficiency perovskite solar cells: multiple points of view. *Chem. Commun.* **59**, 13394-13405 (2023).
18. Kim M, *et al.* Methylammonium chloride induces intermediate phase stabilization for efficient perovskite solar cells. *Joule* **3**, 2179-2192 (2019).
19. Wang J, *et al.* Homogenized optoelectronic properties in perovskites: Achieving high-efficiency solar cells with common chloride additives. *J. Am. Chem. Soc.* **148**, 6229-6237 (2026).
20. Ding B, *et al.* Dopant-additive synergism enhances perovskite solar modules. *Nature* **628**, 299-305 (2024).
21. Xiong Z, *et al.* Homogenized chlorine distribution for >27% power conversion efficiency in perovskite solar cells. *Science* **390**, 638-642 (2025).
