# Supplementary material for: Regulating lattice evolution via an ordered two-dimensional intermediate phase for resilient perovskite solar cells
Source: Nat Commun. 2026 Aug 11;17:9624. doi: 10.1038/s41467-026-76425-3 (PMC13554244; doi:10.1038/s41467-026-76425-3)
Supplement: Supplementary file 2 [file 41467_2026_76425_MOESM2_ESM.pdf]

nature portfolio

Corresponding author(s): Han Pan, Yan Shen, Mingkui Wang

Solar Cells Reporting Summary

Nature Portfolio wishes to improve the reproducibility of the work that we publish. This form is intended for publication with all accepted papers reporting the characterization of photovoltaic devices and provides structure for consistency and transparency in reporting. Some list items might not apply to an individual manuscript, but all fields must be completed for clarity.

For further information on Nature Research policies, including our data availability policy, see Authors & Referees.

Experimental design

Please check the following details are reported in the manuscript, and provide a brief description or explanation where applicable.

1. Dimensions

Area of the tested solar cells — ☒ Yes ☐ No

0.06 cm2

Explain why this information is not reported/not relevant.

Method used to determine the device area — ☒ Yes ☐ No

Measured through a shadow mask with 0.06 cm-2 area. This information can be found in Supporting Information

Explain why this information is not reported/not relevant.

2. Current-voltage characterization

Current density-voltage (J-V) plots in both forward and backward direction — ☒ Yes ☐ No

Figure 4b

Voltage scan conditions — ☒ Yes ☐ No

Scan direction: forward and backward

Explain why this information is not reported/not relevant.

Test environment — ☒ Yes ☐ No

AM1.5G; constant load; 25 oC; without encapsulation; air; RH:30%

Explain why this information is not reported/not relevant.

Protocol for preconditioning of the device before its characterization — ☒ Yes ☐ No

Store in a drying cabinet for 48 hours.

Explain why this information is not reported/not relevant.

Stability of the J-V characteristic — ☒ Yes ☐ No

Maximum Power Point Tracking

Explain why this information is not reported/not relevant.

3. Hysteresis or any other unusual behaviour

Description of the unusual behaviour observed during the characterization — ☐ Yes ☒ No

Provide a description of hysteresis or any other unusual behaviour observed during the characterization.

Explain why this information is not reported/not relevant.

Related experimental data — ☐ Yes ☒ No

Provide a description of the related experimental data.

Explain why this information is not reported/not relevant.

4. Efficiency

External quantum efficiency (EQE) or incident photons to current efficiency (IPCE) — ☒ Yes ☐ No

IPCE values for bias light-free devices were measured in the wavelength range of 300 to 900 nm using IQE200B, Oriel.

Explain why this information is not reported/not relevant.

A comparison between the integrated response under the standard reference spectrum and the response measure under the simulator — ☒ Yes ☐ No

discrepancies below 3% (26.11mA cm-2 vs. 25.36mA cm-2)

Explain why this information is not reported/not relevant.

For tandem solar cells, the bias illumination and bias voltage used for each subcell

☐ Yes
☒ No

Provide a description of the measurement conditions.

Explain why this information is not reported/not relevant.

5. Calibration

Light source and reference cell or sensor used for the characterization

☒ Yes
☐ No

Simulated sunlight at 100 mW cm-2 (Oriel, model 91192). The intensity of the simulated light was precisely calibrated using an NREL-certified KG5-filtered Si photodiode detector.

Explain why this information is not reported/not relevant.

Confirmation that the reference cell was calibrated and certified

☐ Yes
☒ No

Identify the independent certification laboratory.

Explain why this information is not reported/not relevant.

Calculation of spectral mismatch between the reference cell and the devices under test

☒ Yes
☐ No

0.2% mismatch

Explain why this information is not reported/not relevant.

6. Mask/aperture

Size of the mask/aperture used during testing

☒ Yes
☐ No

0.06 cm2

Explain why this information is not reported/not relevant.

Variation of the measured short-circuit current density with the mask/aperture area

☐ Yes
☒ No

Report the difference in the short-circuit current density values measured with the mask and aperture area.

Explain why this information is not reported/not relevant.

7. Performance certification

Identity of the independent certification laboratory that confirmed the photovoltaic performance

☒ Yes
☒ No

Identify the independent certification laboratory.

Explain why this information is not reported/not relevant.

A copy of any certificate(s)

☐ Yes
☒ No

Certificate copies should be provided in the Supplementary information. Please state the supplementary item number.

Explain why this information is not reported/not relevant.

8. Statistics

Number of solar cells tested

☒ Yes
☐ No

Approximately 150 solar cells have been measured, with 30 substrates. There are five sub-cells on each substrate.

Explain why this information is not reported/not relevant.

Statistical analysis of the device performance

☒ Yes
☐ No

This information can be found in Figure S32

Explain why this information is not reported/not relevant.

9. Long-term stability analysis

Type of analysis, bias conditions and environmental conditions

☒ Yes
☐ No

Stability tests of the unencapsulated PSCs at MPPT model under continuous simulated illumination (100 mW cm–2) in a temperature range of 50–55 °C in N2 atmosphere. Stability tests of the unencapsulated PSCs under day/night cycling (50 ° C in light and 25 °C in dark) in N2 atmosphere.

Explain why this information is not reported/not relevant.
