# Supplementary material for: Regulating lattice evolution via an ordered two-dimensional intermediate phase for resilient perovskite solar cells
Source: Nat Commun. 2026 Aug 11;17:9624. doi: 10.1038/s41467-026-76425-3 (PMC13554244; doi:10.1038/s41467-026-76425-3)
Supplement: Supplementary file 3 [file 41467_2026_76425_MOESM3_ESM.pdf]

nature portfolio

Peer Review File

Regulating lattice evolution via an ordered two-dimensional intermediate phase for resilient perovskite solar cells

Corresponding Author: Professor Mingkui Wang

This file contains all reviewer reports in order by version, followed by all author rebuttals in order by version.

Version 0:

Reviewer comments:

Reviewer #1

(Remarks to the Author)
This manuscript introduces a strategy utilizing a 2D perovskite (FPGC2PbI4) as a "sacrificial template" to regulate the crystallographic orientation of 3D perovskite films. The authors demonstrate that this template can induce vertical growth of the (100) crystal plane, thereby effectively suppressing light-induced lattice expansion and reducing lattice distortion from 0.22% to 0.09%. Consequently, the authors report that small-area devices achieved a power conversion efficiency (PCE) of 26.15% and retained over 89% of their initial efficiency after 40 light/dark cycles.
However, the core concept of the "sacrificial template" is essentially indistinguishable from existing strategies that utilize buried 2D/3D heterojunctions to assist crystallization, improve crystal quality, and induce vertical orientation growth to release strain. These strategies have been widely reported in the literature over the past few years (e.g., DOI: 10.1038/s41560-026-01980-4; 10.1038/s41566-025-01797-9; 10.1039/D5EE00156K; etc.). Furthermore, the argumentation regarding the core mechanism of the "sacrificial template" is questionable. To prove that FPGC2PbI4 exists below 80°C and "sacrifices" itself above 80°C to promote the vertical orientation growth of the perovskite (100) plane, the authors rely solely on XRD signals, lacking other equally important evidence (such as NMR, FTIR, etc.) to verify this point.
To assist the authors in refining the manuscript for subsequent revision, detailed queries and suggestions regarding specific experimental designs and data interpretations are listed:
1. The XRD results for the control group in Figure 2A show a 100:011:111 ratio of 3:1:1.2; however, the proportions displayed in the figure are clearly inconsistent with this, please verify.

2. The data in Table S1 indicates that the lattice matching between the (002) plane of FPGC2PbI4 and the 3D perovskite reaches 98.4%, yet the two are shown as completely mismatched in the schematic of Figure 1E.

3. In Figure S5, (i) the authors only display one configuration for the binding of PbI2 with DMF, DMSO, FA+, and FPCG+, respectively; is this an arbitrarily selected configuration for the DFT calculations, or is it the most probable configuration obtained after a conformational search? (ii) The manuscript lacks computational details. What specific method was used to calculate the binding energies? When calculating the binding energies and orbital distributions of FA+ and FPCG+ (and Figure S5 fails to specify which orbitals are shown), was the charge specified? It appears to me that the system charge was not specified when calculating the binding energy between FA+ and FPCG+, otherwise the binding energy should be larger than the current result. These two points directly affect the reliability of this result. Additionally, have the authors compared the binding energies of PbI2 with DMF, DMSO, and FA+ against other studies?

4. Given that the binding strength between FPCG+ and PbI2 far exceeds that of FA+ and PbI2, why does FPGC2PbI4 decompose at around 80°C? This temperature is significantly lower than the thermal decomposition temperature of 3D perovskites, presenting a logical contradiction that directly affects the core conclusion of this work.

5. The manuscript states that FPGC2PbI4 decomposes into PbI2 and organic species at 80°C; do the bulky organic species remain in the lattice or are they expelled to the surface? Why do TOF-SIMS results show a stronger F signal in the bulk and bottom regions, while HRTEM shows identical lattice spacing at the surface, bulk, and bottom? Additionally, the HRTEM images of the control group are not shown. Does the non-uniform distribution of organic species in the bulk affect the long-term stability of the device?

6. Figure S9 shows a significant shift in XRD peak positions between the control and experimental groups; please explain

this.

7. Are the XRD signals evolving with light soaking time in Figure 3A based on ex-situ or in-situ/quasi-in-situ measurements? If they are ex-situ signals, the reliability of this result is significantly compromised.

8. The surface GIWAXS signal intensity in Figure S10 is notably higher than the bulk GIWAXS signal in Figure 2, which is evidently caused by the inconsistent color bar ranges used, and this is misleading to readers.

9. The reported efficiency difference between the target and control devices is excessively large, suggesting that may the efficiency of the control device may have been artificially lowered. Furthermore, the target device shows a significant improvement in VOC compared to the control, yet the authors have offered no explanation for this.

10. Additionally, there are several non-scientific issues: (i) in many places, the manuscript merely describes experimental phenomena without providing further conclusions or insights; (ii) many schematics depict the traditional 3D perovskite crystal structure as excessively defective.

Reviewer #2

(Remarks to the Author)
In this work, the authors propose a sacrificial template strategy for the regulation of the lattice orientation of perovskite films, with a view to improving device stability. Specifically, a two-dimensional perovskite 1-(p-fluorophenyl)biguanide lead iodide, with strong (00l) texture and high lattice-matched heterointerface to the three dimensional perovskite, serves as a template. These findings contribute to the fundamental understanding and technical improvement of perovskite solar energy conversion technologies. Therefore, the present manuscript is suitable for publication in Nature Communications after minor revision.
1. The authors claim that the uniform crystallization is achieved across the entire space through the sacrificial template strategy. In order to provide substantiating evidence for this claim, further characterization of the bottom interface should be performed. Such further characterization may include XRD or SEM analysis, which would serve to improve clarity.
2. The interaction between PbI2 and DMF or other components in the precursor solution, amongst other factors, has been identified as a pivotal parameter in determining the quality of the film. The authors are recommended to provide information for estimating the binding energies in Figure S5. The necessary description should be given in the experimental methods.
3. In order to substantiate the enhancements in in-plane uniformity (Figure 4E), the proportions or intensity ratios of different crystal planes across the large-area film should be provided.
4. As shown in Figure S25, the stability test entailed the replacement of the hole transport material Spiro-OMeTAD with alternative materials. Consequently, it is imperative to elucidate the initial performance of all devices subjected to stability tests. A commentary on the phenomena observed in the stability test is highly warranted in this study.
5. There are some typographical errors and inconsistencies in the use of terminology throughout the manuscript. It is recommended that a thorough proofreading is carried out in order to correct these issues and unify the terminology used across the main text and supplementary information.
6. The study concluded that the template under investigation facilitates the vertical orientation growth of the (100) crystal plane of the bulk perovskite layer. The enhanced lattice orientation that is observed throughout the entire space effectively restricts photo-induced lattice expansion and decreases the lattice distortion, thereby contributing to improved operational stability. In order to present this result, it is necessary to undertake preparatory work, including the impact of these crystal planes and their orientations on perovskite solar cells, such as power conversion efficiency and stability. In addition, it is imperative to ascertain efficacious methodologies for the regulation of these crystal planes.

Reviewer #3

(Remarks to the Author)
In this article, Ma et al. demonstrate a highly oriented two-dimensional (2D) perovskite that acts as a sacrificial intermediate layer for the templated growth of three-dimensional (3D). The photovoltaic devices fabricated with this technique reveal power conversion efficiencies of 26.15 % for small area and 22.25 % for large area. Additionally, the device exhibits excellent day/night cycling stability under realistic conditions (ISOS-LC-1 protocol, ~50 °C in light and ~25 °C in dark). Although the work has some merit, I have major concerns that need to be addressed before this manuscript can be published in Nature Communications.
1. The authors claim that the 2D FPGC2PbI4 perovskite template directs 3D crystallization via lattice-matched, quasi-epitaxial growth (page 5, line 109). For epitaxial growth and lattice templating, the in-plane lattice parameter of 2D perovskite should be matched with the 3D perovskite. The out-of-plane (002) lattice spacing does not contribute to epitaxial templating. Therefore, the claim of lattice templating is not justified.
2. Is there any existence of 2D crystal seeds found in the precursor solution? DLS or similar investigations could be performed to confirm that.
3. For the in-situ formation of 2D, FPGC molecule has been utilized in the perovskite precursor solution. The role of “Cl” is not explained at all in the manuscript. The observed orientation effect probably originates form enhanced crystallization facilitated by the 2D component and Cl incorporation. The role of 2D and Cl should be investigated systematically.
4. TOF-SIMS and XPS analyses demonstrate that the FPGC molecule is incorporated in the final film, while the XRD analysis demonstrates the 2D decomposes above 80 ˚C-Why? Additionally, if the molecule is readily extinguished at high

temperature, that could lead to degradation of perovskite in harsh conditions.
5. Why do GIXRD or XRD analyses reveal no low-angle peak for 2D perovskite in the final film?
6. The cross-section FESEM in Figure 2D is not clear. Please provide high resolution FESEM image.
7. The authors have observed a performance recovery for both control and target devices after the dark phase of the day/night cycle. What is the reason for this observation, and why is it more pronounced for the control sample. Also, it will be interesting to co-relate the acceleration factor for the day/night cycling stability with film analyses.
8. Minor Comment: The box plot for Jsc, Voc, and FF of the devices should be provided in the Supporting Information to better illustrate the device trend.

Version 1:

Reviewer comments:

Reviewer #1

(Remarks to the Author)
The authors have addressed most of my concerns and now the manuscript could be considered for publication.

Reviewer #2

(Remarks to the Author)
I have carefully reviewed the revised manuscript and the authors' responses to my previous comments. The authors have addressed all issues thoroughly and appropriately. The revised version is significantly improved in terms of both scientific rigor and presentation clarity.
I have no further reservations and recommend acceptance of this paper without additional revision.

Reviewer #3

(Remarks to the Author)
The authors have successfully addressed the concerns raised by the reviewers, and the revised manuscript has been substantially improved. The newly included stability data are compelling and further strengthen the overall impact of the work. In its current form, I consider the manuscript suitable for publication in Nature Communications.

Open Access This Peer Review File is licensed under a Creative Commons Attribution 4.0 International License, which permits use, sharing, adaptation, distribution and reproduction in any medium or format, as long as you give appropriate credit to the original author(s) and the source, provide a link to the Creative Commons license, and indicate if changes were made.
In cases where reviewers are anonymous, credit should be given to 'Anonymous Referee' and the source.
The images or other third party material in this Peer Review File are included in the article’s Creative Commons license, unless indicated otherwise in a credit line to the material. If material is not included in the article’s Creative Commons license and your intended use is not permitted by statutory regulation or exceeds the permitted use, you will need to obtain

permission directly from the copyright holder.
To view a copy of this license, visit https://creativecommons.org/licenses/by/4.0/

Reviewers' comments:

Reviewer #1 (Remarks to the Author):

C1.1 This manuscript introduces a strategy utilizing a 2D perovskite ($FPGC_2PbI_4$) as a "sacrificial template" to regulate the crystallographic orientation of 3D perovskite films. The authors demonstrate that this template can induce vertical growth of the (100) crystal plane, thereby effectively suppressing light-induced lattice expansion and reducing lattice distortion from 0.22% to 0.09%. Consequently, the authors report that small-area devices achieved a power conversion efficiency (PCE) of 26.15% and retained over 89% of their initial efficiency after 40 light/dark cycles.

However, the core concept of the "sacrificial template" is essentially indistinguishable from existing strategies that utilize buried 2D/3D heterojunctions to assist crystallization, improve crystal quality, and induce vertical orientation growth to release strain. These strategies have been widely reported in the literature over the past few years (e.g., DOI: 10.1038/ s41560-026-01980-4; 10.1038/s41566-025-01797-9; 10.1039/D5EE00156K; etc.).

Authors Response:

We thank the reviewer for this insightful comment. We agree that the original Introduction did not sufficiently distinguish our strategy from the rapidly growing literature on buried 2D/3D heterojunctions and 2D-assisted crystallization. In response, we have substantially revised the Introduction to better position our work relative to these prior studies and to clarify our specific conceptual focus.

We agree that our study shares a general objective with previous reports: utilizing a low-dimensional perovskite-related phase to regulate crystallization and improve film quality. However, the central feature of our work is not the construction of a stable buried 2D/3D heterojunction as a final functional architecture. Instead, our study focuses on a transient $FPGC_2PbI_4$ intermediate that forms during the early stage of annealing and restructures the crystallization pathway before being consumed during

conversion to the 3D perovskite.

We now emphasize three key distinctions in the revised manuscript: 1) The layered phase in our system is transient rather than deliberately retained as a stable buried interlayer. 2) Its primary role is to regulate the crystallization pathway by suppressing competing intermediate species and promoting orientationally coherent 3D growth, rather than merely optimizing the final interfacial structure. 3) The resulting structural effect is full-space orientational uniformity with reduced cyclic lattice deformation, rather than localized interfacial passivation or a buried heterojunction effect.

Furthermore, to avoid overstating our novelty or implying that our approach constitutes an entirely separate category from previous 2D-related strategies, we have revised our terminology throughout the manuscript. Specifically, we have removed the stand-alone conceptual label "sacrificial template". Instead, we adopt more precise descriptions where appropriate, such as "2D perovskite intermediate phase" and "ordered intermediate phase".

As suggested, the following sentences were revised on page 4, lines 1-20 in the revised manuscript:

"Precise crystallization control offers a viable pathway to reduce lattice disorder, promote preferential orientation, and relieve residual stress[8]. Consequently, strategies spanning nucleation suppression[28], high-binding-energy additives[29], and intermediate-phase engineering[30,31] have been developed to modulate perovskite crystallization. In particular, two-dimensional (2D) perovskites have emerged as versatile interfacial modifiers[32], buried 2D/3D heterostructures[33], and crystallization-directing templates[34-38]. Despite these advances, permanently retained 2D passivation layers suffer from intrinsic limitations[39]. Their low electronic conductivity and propensity to adopt horizontally stacked quantum-well configurations severely impede vertical charge extraction[40]. Furthermore, residual, uncontrolled mixed-dimensional phases within the bulk matrix can introduce parasitic charge-trapping sites[41,42]. More fundamentally,

conventional 2D strategies primarily target post-crystallization interfacial architectures or localized heterojunctions[43], rather than comprehensively directing the early-stage crystallization pathway to enforce orientation uniformity throughout the entire film thickness. Emerging evidence also indicates that initially phase-pure 2D/3D stacks can dynamically evolve during the aging process[44], meaning the structural fate of the 2D phase intimately dictates long-term device stability[45]. Consequently, whether a transient, non-persistent 2D intermediate can successfully regulate the crystallization pathway and imprint full-space orientational uniformity onto the final 3D framework remains an open and critical question."

New references are added in the revised manuscript:

28. Park J, *et al.* Controlled growth of perovskite layers with volatile alkylammonium chlorides. *Nature* **616**, 724-730 (2023).

29. Yang Y, *et al.* A thermotropic liquid crystal enables efficient and stable perovskite solar modules. *Nat. Energy* **9**, 316-323 (2024).

30. Correa-Baena J-P, *et al.* Homogenized halides and alkali cation segregation in alloyed organic-inorganic perovskites. *Science* **363**, 627-631 (2019).

31. Li F, *et al.* Hydrogen-bond-bridged intermediate for perovskite solar cells with enhanced efficiency and stability. *Nat. Photonics* **17**, 478-484 (2023).

32. Song Z, *et al.* Buried and bulk synergistic engineering enables high-performance inverted 2D/3D perovskite solar cells. *Energy Environ. Sci.* **18**, 3740-3749 (2025).

33. Li H, *et al.* 2D/3D heterojunction engineering at the buried interface towards high-performance inverted methylammonium-free perovskite solar cells. *Nat. Energy* **8**, 946-955 (2023).

34. Chen J, *et al.* Surface template realizing oriented perovskites for highly efficient solar cells. *Adv. Mater.* **37**, 2417054 (2025).

35. Wang Y, *et al.* Highly oriented $FAPbI_3$ *via* 2D ruddlesden popper perovskite template growth. *Adv. Energy Mater.* **14**, 2401721 (2024).

36. Sidhik S, *et al.* Two-dimensional perovskite templates for durable, efficient formamidinium perovskite solar cells. *Science* **384**, 1227-1235 (2024).

37. Gao Y*, et al.* Spontaneous 2D perovskite formation at the buried interface of perovskite solar cells enhances crystallization uniformity and defect passivation. *Nat. Photonics* **20**, 178-185 (2026).
38. Zhao Q*, et al.* Buried 2D/3D heterojunction in n-i-p perovskite solar cells through solid-state ligand-exchange reaction. *Nat. Energy* **11**, 581-592 (2026).
39. Teale S, Degani M, Chen B, Sargent EH, Grancini G. Molecular cation and low-dimensional perovskite surface passivation in perovskite solar cells. *Nat. Energy* **9**, 779-792 (2024).
40. Liang C*, et al.* Two-dimensional Ruddlesden-Popper layered perovskite solar cells based on phase-pure thin films. *Nat. Energy* **6**, 38-45 (2021).
41. Shih M-C*, et al.* A 2D/3D heterostructure perovskite solar cell with a phase-pure and pristine 2D Layer. *Adv. Mater.* **37**, 2416672 (2025).
42. Gu H, Xia J, Liang C, Chen Y, Huang W, Xing G. Phase-pure two-dimensional layered perovskite thin films. *Nat. Rev. Mater.* **8**, 533-551 (2023).
43. Li J*, et al.* Homogeneous coverage of the low-dimensional perovskite passivation layer for formamidinium-caesium perovskite solar modules. *Nat. Energy* **9**, 1540-1550 (2024).
44. Tan S*, et al.* Spontaneous formation of robust two-dimensional perovskite phases. *Science* **388**, 639-645 (2025).
45. Sutanto AA*, et al.* Dynamical evolution of the 2D/3D interface: a hidden driver behind perovskite solar cell instability. *J. Mater. Chem. A* **8**, 2343-2348 (2020).

C1.2 Furthermore, the argumentation regarding the core mechanism of the "sacrificial template" is questionable. To prove that $FPGC_2PbI_4$ exists below 80°C and "sacrifices" itself above 80°C to promote the vertical orientation growth of the perovskite (100) plane, the authors rely solely on XRD signals, lacking other equally important evidence (such as NMR, FTIR, etc.) to verify this point.

Authors Response:

We validate the reviewer's concern and agree that relying solely on XRD data is

insufficient to establish the proposed crystallization pathway. Accordingly, we have reinforced our mechanism by incorporating a comprehensive array of complementary characterizations. To prove precursor-state interactions, we added ^{1}H NMR and FTIR measurements, both of which show distinct spectral shifting that indicates strong chemical coordination between FPGC and PbI_2 rather than a physical mixture. This is further corroborated by DLS data, which show a unique colloidal population forming in the modified precursor solution. To capture the phase transition kinetics, *in situ* PL was deployed during film formation and thermal annealing. The control film tracks through a disordered intermediate plateau, whereas the target film suppresses this phase heterogeneity, showing an intermediate-mediated pre-growth signature and a regulated, slower crystallization rate. Lastly, TGA confirms that FPGC does not decompose at 80 °C, confirming that the disappearance of the 2D XRD peaks signals structural consumption during 3D framework conversion rather than thermal degradation. These multi-technique results successfully substantiate our revised, regulated crystallization model.

As suggested, the following sentences were revised on page 6, lines 1-4 in the revised manuscript: "<u>Thermogravimetric analysis (TGA) confirms that the isolated FPGC molecule remains stable up to 217 °C (Figure S3); thus, the dissolution of the 2D XRD features above 80 °C does not stem from molecular decomposition, but rather from a loss of long-range crystalline order within the $FPGC_2PbI_4$ framework.</u>"

On page 7, lines 1-5 in the revised manuscript: "<u>Dynamic light scattering (DLS) profiles reveal that the FPGC-containing precursor solution develops an additional population of large colloids (>1 μm) alongside a narrower, more concentrated distribution of smaller species in the 30-90 nm range (Figure S5). This behavior points to a profound reconstruction of the precursor colloidal structure, which is consistent with the formation of seed-like aggregates driven by a strong, preferential association between the FPGC cation and PbI_2[46, 47].</u>"

On page 8, lines 1-9 in the revised manuscript: "<u>This modified crystallization pathway</u>

was further substantiated by in situ photoluminescence (PL) measurements conducted during spin-coating and early-stage annealing (Figure S6). Unlike the control film, which exhibits a plateau-like PL signature characteristic of disordered intermediate species during spin-coating, the target film completely bypasses this phase. During the subsequent annealing stage, the PL intensity of the target film takes longer time to reach its peak value. Collectively, these structural and optical findings demonstrate that the FPGC additive successfully arrests the formation of disordered solvate intermediates at the nucleation onset by introducing a highly ordered, transient 2D template."

On page 9, lines 6-11 in the revised manuscript: "Liquid-state proton nuclear magnetic resonance (^{1}H NMR) and Fourier transform infrared (FTIR) spectroscopies further validate this precursor-state reconstruction (Figure S9). Upon the introduction of PbI_2, pronounced chemical shift and vibrational frequency variations emerge within the N-H and biguanide-framework spectral regions, confirming a robust chemical coordination between FPGC and the lead iodide octahedra in the solution phase."

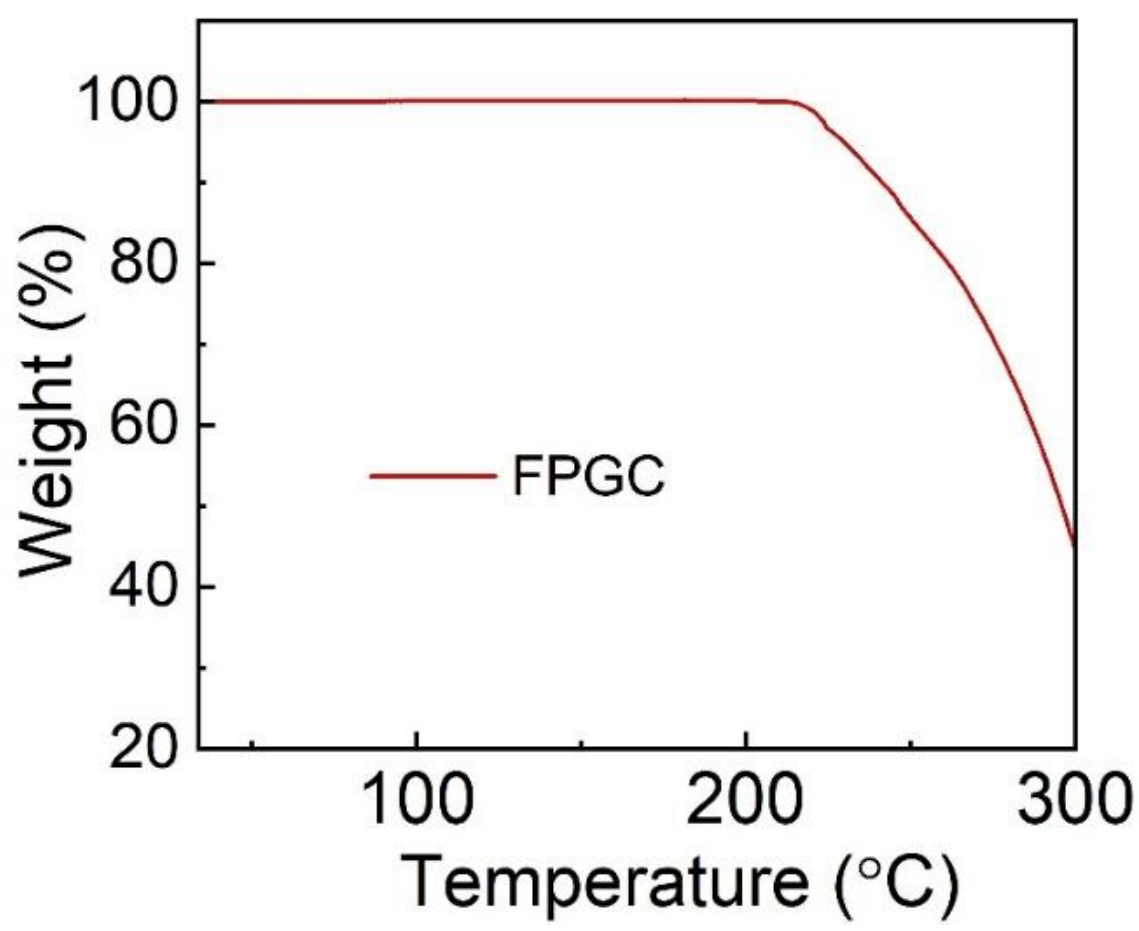

Figure S3. Thermogravimetric analysis profile of the neat FPGC molecular powder.

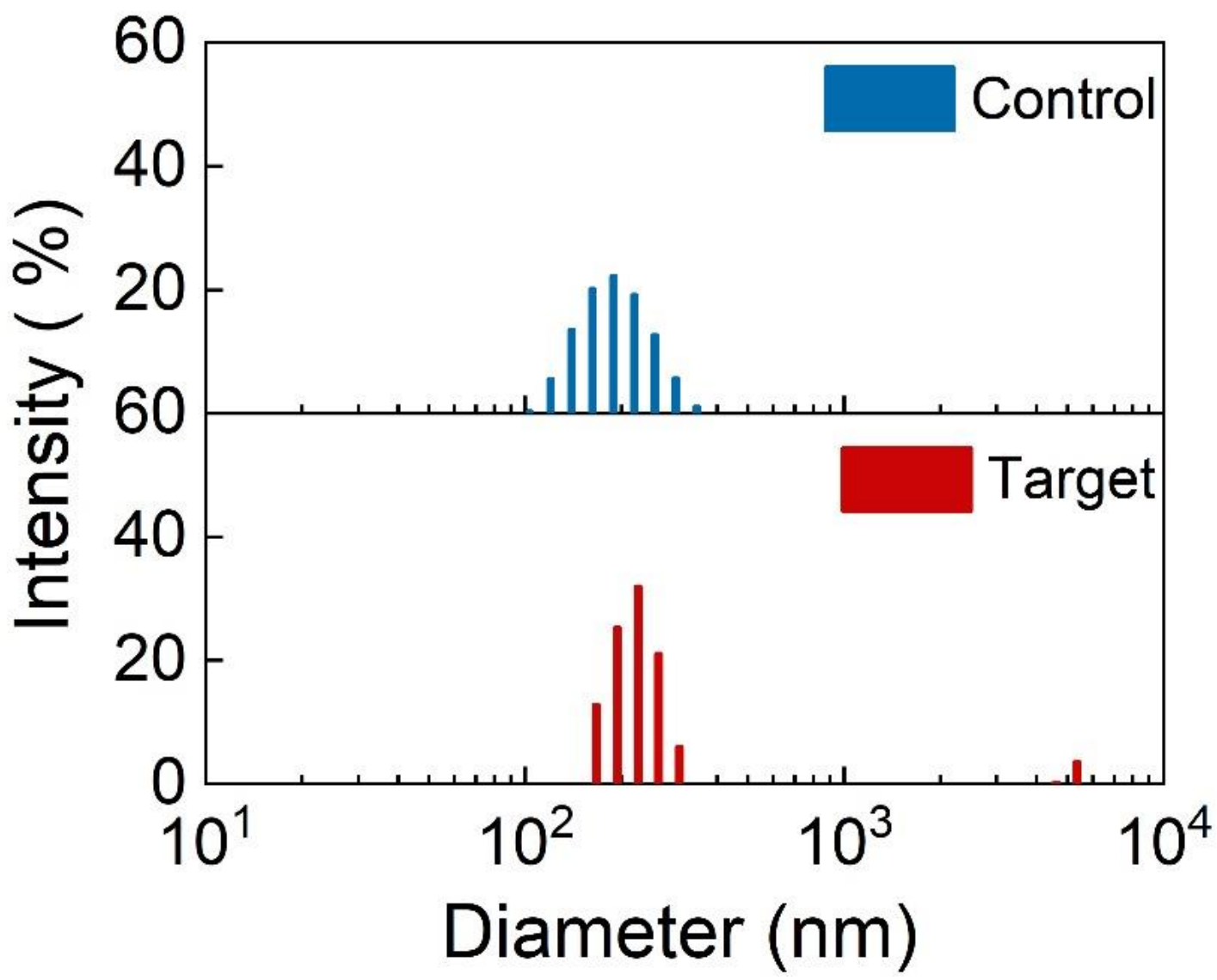

Figure S5. Dynamic light scattering spectra of control and target precursor solutions.

As suggested, the following sentences were added on pages 12-13 in the Supporting information: "To further elucidate the crystallization kinetics and the specific role of the FPGC ligand during film formation, *in situ* photoluminescence (PL) spectroscopy was conducted during both the spin-coating and initial thermal annealing stages. The control and target films exhibited markedly different PL evolution profiles, indicating distinct crystallization pathways [6] (Supplementary Fig. 6a, b). Following the dripping of the chlorobenzene antisolvent at 22.5 s, the PL intensity of the control film increased gradually, reflecting accelerated solvent extraction [7]. A plateau-like region was subsequently observed from 23.0 to 26.6 s (Supplementary Fig. 6c), during which the PL intensity remained nearly constant while the emission spectral profile was broadly distributed over the 650–800 nm range. This behavior suggests the presence of multiple inhomogeneous intermediate species, which can be attributed to a disordered phase evolution involving methylammonium (MA)-rich nanocrystalline nuclei [8]. Beyond 26.6 s, the PL intensity rose sharply, and the emission spectra became dominated by a single peak at 757 nm after 28.0 s, signifying the formation and progressive growth of formamidinium (FA)-rich nuclei as solvent evaporation proceeded.

In contrast, the target film displayed a much more rapid increase in PL intensity immediately after antisolvent deposition, featuring a virtually suppressed plateau region

and no discernible MA-rich emission characteristics. This result indicates that the formation of disordered intermediate species is strongly inhibited by the presence of FPGC. After 38.0 s, both film systems exhibited characteristic PL quenching, marking the final stage of wet-film conversion. At this juncture, the majority of the solvent had evaporated, exposing the perovskite crystallites to adjacent domains and the local environment, which triggered grain-boundary formation and rapid non-radiative recombination [9].

The PL evolution tracked during thermal annealing over a longer timescale (0–600 s) further revealed distinct kinetic behaviors between the two systems (Supplementary Fig. 6d, e). For the control film, the PL intensity increased rapidly during the initial 0–7.8 s, reaching a maximum as small crystallites continuously grew and coalesced into larger grains. Conversely, the incorporation of FPGC within the target film induced a distinct, intermediate-stabilized pre-growth stage during the first 0–7.0 s. Consequently, the overall crystallization rate was substantially retarded; the PL intensity reached its peak at approximately 21.6 s and remained elevated for a prolonged duration (Supplementary Fig. 6f). These findings collectively demonstrate that FPGC regulates the crystallization pathway by introducing a stable, transient intermediate phase. This action effectively suppresses disordered early-stage phase segregation and enables a slower, well-controlled crystal growth process. This kinetic modulation directly correlates with the observed formation of larger grains, improved crystallinity, and a more preferentially oriented final perovskite film."

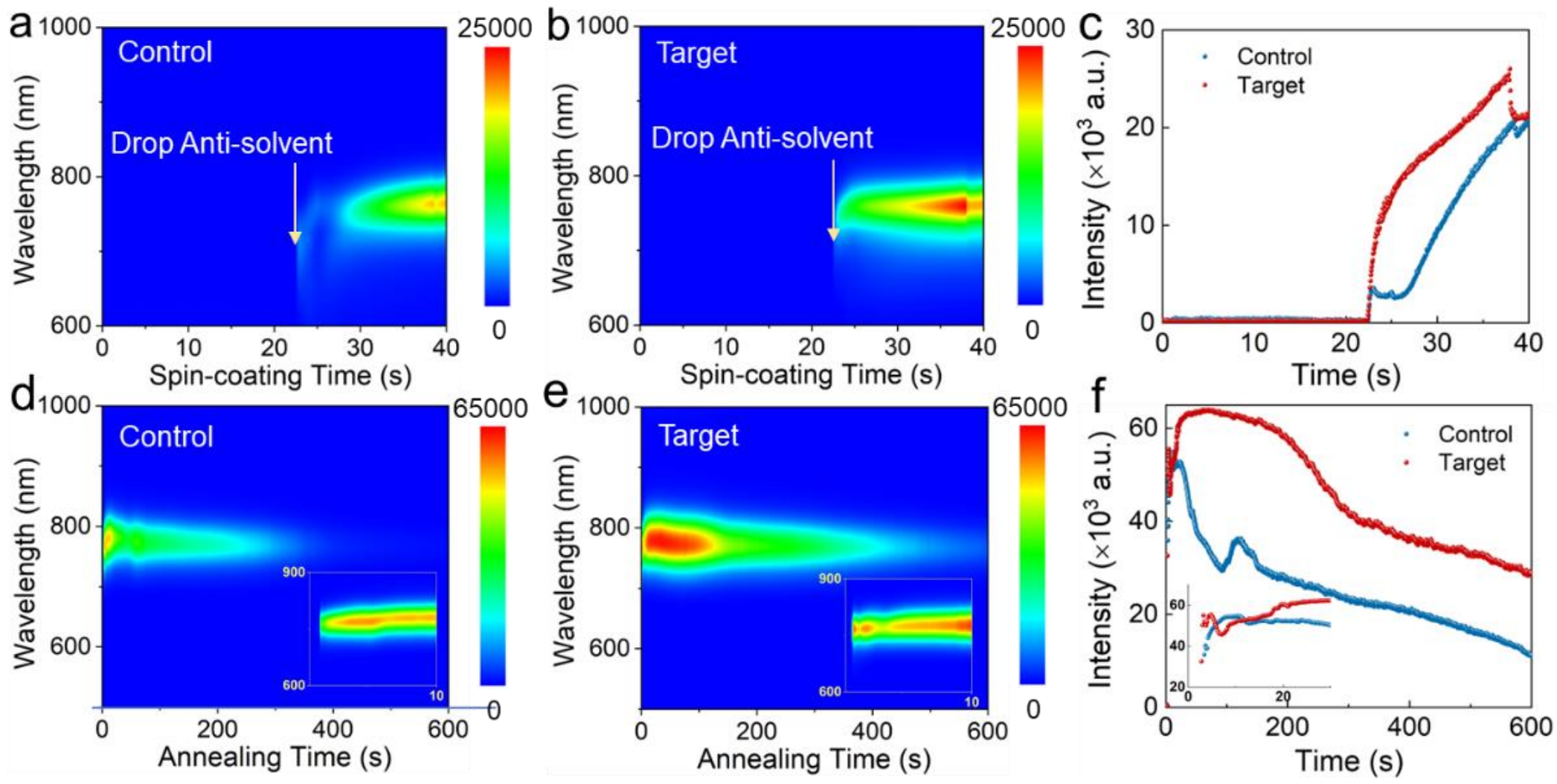

Figure S6. Evaluation of perovskite film growth kinetics via *in situ* photoluminescence. In situ PL profiles recorded during the spin-coating process of the a) control and b) target perovskite films. C) Comparative analysis of the PL intensity evolution extracted from the spin-coating profiles of the control and target films. In situ PL profiles captured during the initial annealing stage of the d) control and e) target perovskite films (insets: magnified views of the 0–10 s interval). F) Comparative analysis of the PL intensity evolution during the initial annealing stage of the control and target films (inset: magnified view of the 0–30 s interval).

As suggested, the following sentences were added on pages 17-18 in the Supporting information: "To experimentally probe the intermolecular interactions within the precursor solution, ^1H nuclear magnetic resonance (^1H NMR) spectroscopy was performed. As illustrated in the ^1H NMR spectra (Supplementary Fig. 9a, b), the introduction of PbI_2 into the FPGC solution induced a significant upfield shift of the resonance peak corresponding to the protonated biguanide amine $-NH_3^+$ group, moving from 9.94 to 9.08 ppm. Concurrently, the multi-peak resonance assigned to the secondary N-H groups of the biguanide backbone shifted downfield from 7.41–7.33 ppm to 7.35–7.29 ppm. These coordinate variations demonstrate a pronounced change in local electronic shielding, confirming strong chemical interactions between the FPGC cation and the PbI_2 framework.

To further elucidate these coordinate frameworks, Fourier-transform infrared (FTIR) spectroscopy was conducted on the corresponding thin films (Supplementary Fig. 9c, d). Upon coordination with PbI_2, the characteristic N-H stretching vibrational band of pristine FPGC at 3297 cm^{-1} exhibited clear broadening, signifying a substantial alteration in the local hydrogen-bonding network surrounding the biguanide moiety. Similarly, the N-H bending mode (1609 cm^{-1}) and the C=N stretching mode (1649 cm^{-1}) of the FPGC framework both shifted to lower wavenumbers, appearing at 1607 and 1643 cm^{-1}, respectively. These vibrational modifications provide definitive evidence of specific chemical coupling, most notably through N···Pb coordination bonds alongside secondary N–H···I hydrogen-bonding networks. Collectively, these precursor-state and wet-film spectroscopic observations substantiate the pre-organization of PbI_2–ligand complex species, establishing a chemical foundation that directly aligns with the subsequent formation of the transient FPGC-derived intermediate phase during film crystallization."

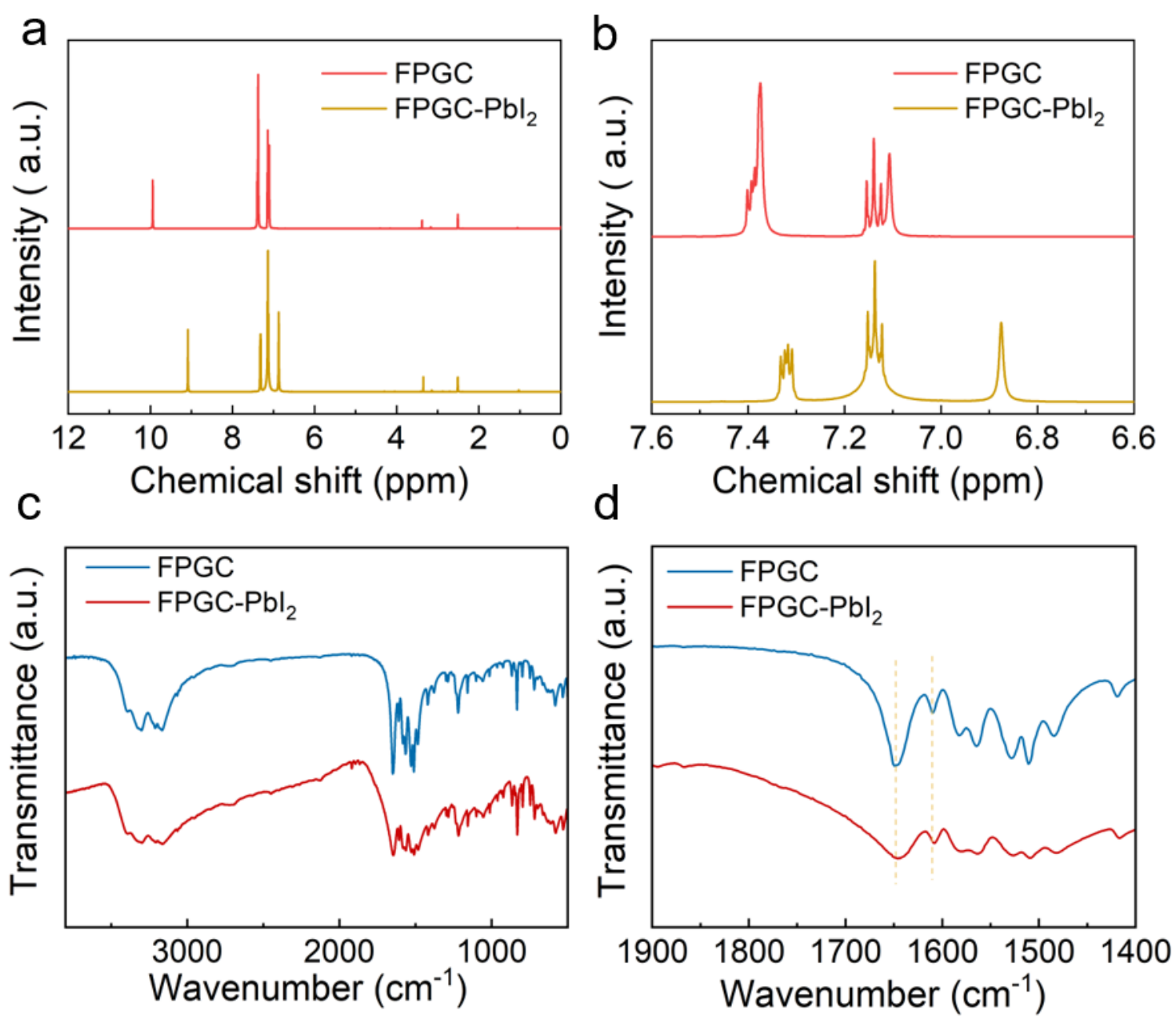

Figure S9. FTIR and ¹H NMR analysis of FPGC-PbI_2 molecular interactions. a) ¹H NMR spectra of FPGC and FPGC+PbI_2 in DMSO-*d6*. b) Magnified view of the highlighted region in a). c) FTIR spectra of the FPGC and FPGC-PbI_2. d) Magnified view of the highlighted region in c).

New references are added in the revised manuscript:

46. Luo C, *et al.* Facet orientation tailoring *via* 2D-seed- induced growth enables highly efficient and stable perovskite solar cells. *Joule* **6**, 240-257 (2022).

47. Chai Y, *et al.* Tracking *α*-phase perovskite crystallization induced by single-crystal seeds for high-performance solar cells. *Chem. Eng. J.* **529**, 173193 (2026).

New references are added in the Supporting information:

6. Yang H, Guo H, Wang Y, Chen R, Tsang S-W, Cheng Y. In-situ photoluminescence for perovskite crystallization: bridging mechanistic insights and device engineering control. *Adv. Mater.*, e18643 (2026).

7. Zhou N, *et al.* Homologous molecule treatment in perovskite solar cells: synergistic management of holistic defect and charge transfer. *Adv. Funct. Mater.* **35**, 2418798 (2025).

8. He Q, *et al.* A self-assembled molecule directs ordered *α*-$FAPbI_3$ for n-i-p perovskite solar cells. *Nat. Commun.* **17**, 1479 (2026).

9. Li F, *et al.* Hydrogen-bond-bridged intermediate for perovskite solar cells with enhanced efficiency and stability. *Nat. Photonics* **17**, 478-484 (2023).

C1.3 To assist the authors in refining the manuscript for subsequent revision, detailed queries and suggestions regarding specific experimental designs and data interpretations are listed:

1. The XRD results for the control group in Figure 2A show a 100:011:111 ratio of 3:1:1.2; however, the proportions displayed in the figure are clearly inconsistent with this, please verify.

Authors Response:

We are highly grateful to the reviewer for this careful and important comment. We agree that the intensity ratio reported in the original manuscript was incorrect, stemming from a transcription error during drafting. Upon rechecking the raw XRD data and peak

profiles, we confirmed that the diffraction intensity ratio of the (100):(110):(111) reflections is 16.7:1:1.5 for the control film and increases markedly to approximately 140:1:2.1 for the target film. To provide a more comprehensive validation, we also calculated the integrated peak-area ratios, which exhibit a parallel trend, increasing from 14.1:1:1.3 (control) to 141:1:1.4 (target). We have corrected these values throughout the revised manuscript. Crucially, this correction reinforces and does not alter our core conclusion that the target film exhibits a significantly enhanced preferential orientation along the (100) crystallographic direction.

As suggested, the following sentences were revised on page 9, lines 24-28 in the revised manuscript: "The control film exhibits a relatively random crystallographic orientation, characterized by a (100):(110):(111) peak intensity ratio of approximately 16.7:1:1.5 (Figure 2A). In the target film, this ratio shifts drastically to ~ 140:1:2.1, indicating a pronounced texturing of the (100) crystal plane. The integrated peak-area ratio of the (100):(110):(111) reflections similarly increases from 14.1:1:1.3 in the control film to 141:1:1.4 in the target film."

The corresponding annotation in Figure 2A has also been corrected accordingly.

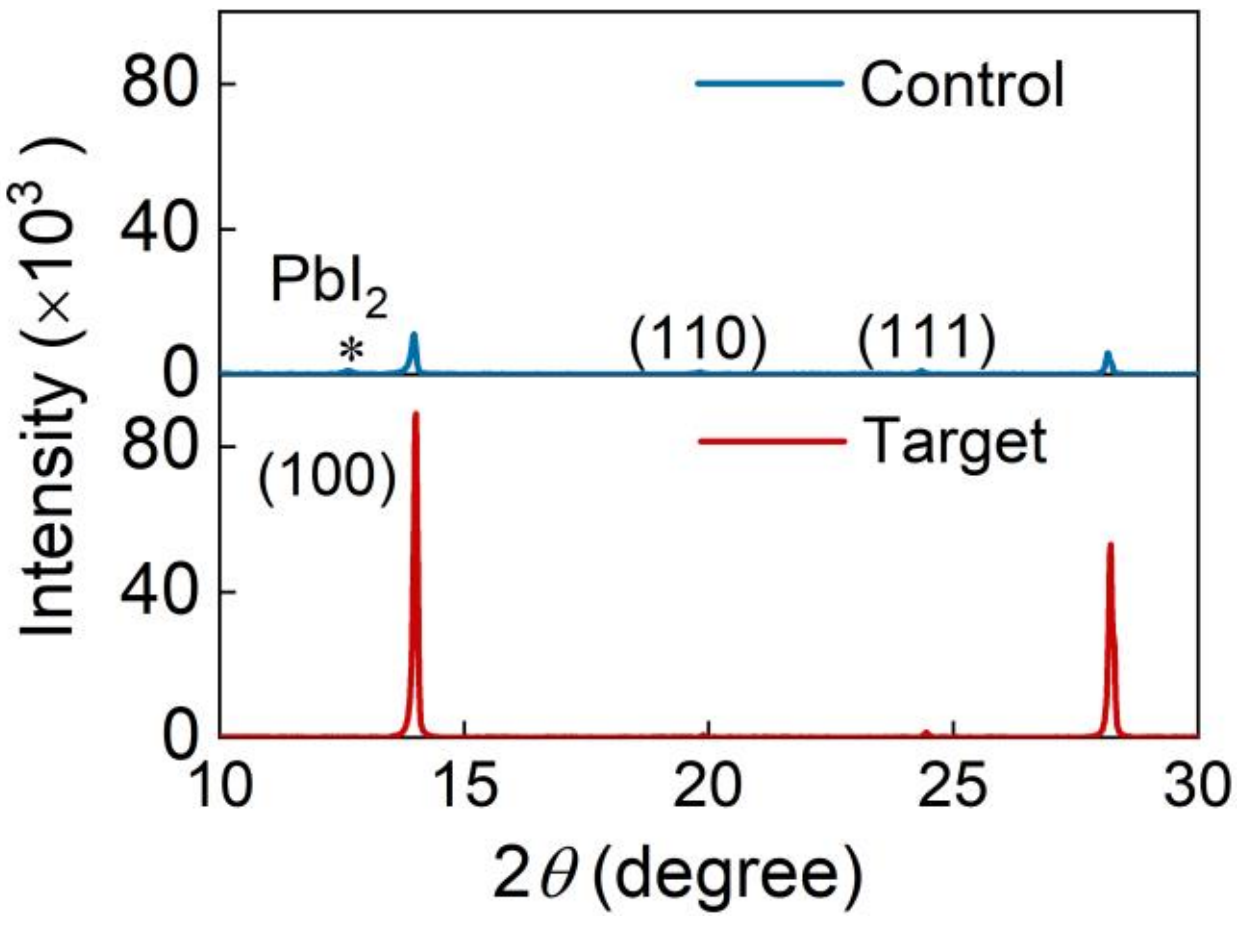

Figure 2. A) Comparison of the XRD patterns of the control and target films.

2. The data in Table S1 indicates that the lattice matching between the (002) plane of $FPGC_2PbI_4$ and the 3D perovskite reaches 98.4%, yet the two are shown as completely mismatched in the schematic of Figure 1E.

Authors Response:

We thank the reviewer for pointing out this inconsistency. We agree that the original schematic in Figure 1E lacked sufficient detail. The 98.4% similarity value in Table S1 evaluates the interplanar spacing correspondence between the two phases. Specifically, the lamellar spacing of the (001) planes of the $FPGC_2PbI_4$ intermediate phase is roughly twice that of the 3D perovskite (100) planes; thus, the (002) reflections of the intermediate closely align with the (100) reflections of the 3D phase. We have modified Figure 1E in the revised manuscript to clearly delineate this structural relationship between the (001) intermediate planes and the (100) 3D perovskite planes.

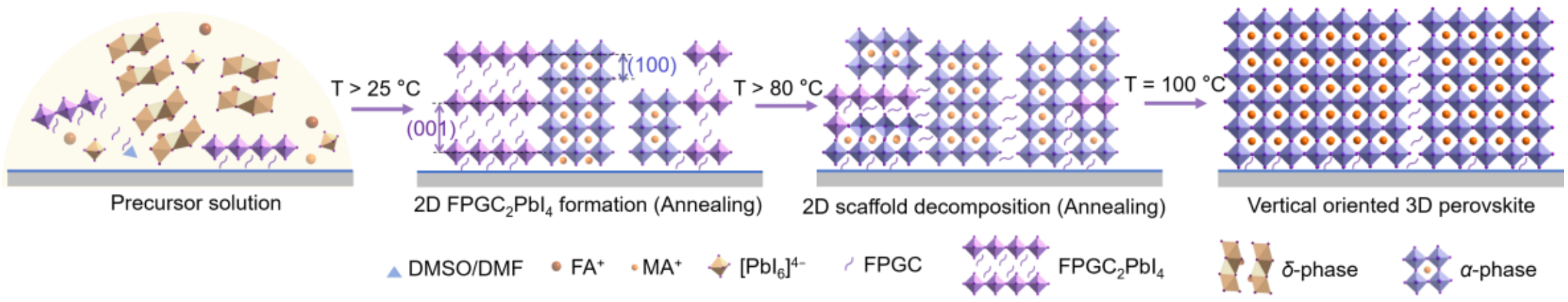

Figure 1. E) Schematic illustration depicting the ordered intermediate growth strategy and the templated crystallization pathway during the thermal annealing process.

3. In Figure S5, (i) the authors only display one configuration for the binding of PbI_2 with DMF, DMSO, FA^+, and $FPCG^+$, respectively; is this an arbitrarily selected configuration for the DFT calculations, or is it the most probable configuration obtained after a conformational search? (ii) The manuscript lacks computational details. What specific method was used to calculate the binding energies? When calculating the binding energies and orbital distributions of FA^+ and $FPCG^+$ (and Figure S5 fails to specify which orbitals are shown), was the charge specified? It appears to me that the system charge was not specified when calculating the binding energy between FA+ and FPCG+, otherwise the binding energy should be larger than the current result. These two points directly affect the reliability of this result. Additionally, have the authors compared the binding energies of PbI_2 with DMF, DMSO, and FA^+ against other studies?

Authors Response:

We sincerely thank the reviewer for this insightful and important comment. We completely agree that both the initial binding configurations and the correct electronic charge states are paramount for obtaining accurate and reliable density functional theory (DFT) results. We have addressed each of these points rigorously, recalculated the entire data set, and updated the manuscript accordingly.

First, following the reviewer's vital observation, we carefully re-examined the electronic charge configurations used in our previous modeling. We identified that the prior calculations involving isolated FA^+ and $FPGC^+$ cations, as well as their corresponding PbI_2–ligand complexes, had inadvertently been treated as net-neutral cells.

To rectify this, we have recalculated the total energies of FA^+, $FPGC^+$, PbI_2–FA^+, and PbI_2–$FPGC^+$ by explicitly setting the total simulation cell charge to +1 (by reducing the total electron count by one). Conversely, the standalone PbI_2 framework, isolated solvents (DMF, DMSO), and their coordinated complexes (PbI_2–DMF, PbI_2–DMSO) were correctly maintained as net-neutral systems.

Furthermore, to provide a more chemically representative model of the interaction between these organic cations and the actual iodoplumbate framework within the precursor solution, we expanded our computational scope to include net-neutral ion-pair systems: PbI_3^-– FA^+ and PbI_3^-– $FPGC^+$. All updated binding energies and three-dimensional charge density difference profiles have been incorporated into the revised Fig. S8a.

Second, to eliminate potential structural bias stemming from a single, arbitrarily chosen initial geometry, we constructed multiple initial binding configurations for every single system evaluated (PbI_2–DMF, PbI_2–DMSO, PbI_2–FA^+, PbI_2–$FPGC^+$, PbI_3^-–FA^+ and PbI_3^-–$FPGC^+$). These configurations systematically accounted for diverse coordination sites and molecular orientations. Each candidate framework was fully relaxed using identical, high-stringency DFT parameters. The most thermodynamically stable (lowest-energy) optimized configurations were selected for the definitive binding

energy calculations and are now displayed in **Fig. S8b, c**.

The binding energy (ΔE_{bind}) was evaluated using the following standard equation:

$$\Delta E_{\text{bind}} = E_{\text{complex}} - E_{\text{PbI}_2} - E_{\text{ligand}}$$

where E_{complex}, E_{PbI_2} and E_{ligand} represent the total energies of the optimized PbI_2–ligand complex, isolated PbI_2 slab, and the isolated ligand (or corresponding ion-pair components), respectively. These explicit computational details have been incorporated into the revised Supplementary Information.

Third, we appreciate the opportunity to clarify that the spatial distributions illustrated in Fig. S8a do not represent single-particle molecular orbital distributions. Rather, they plot the three-dimensional charge density difference ($\Delta\rho(r)$), evaluated as:

$$\Delta\rho(r) = \rho_{\text{complex}} - \rho_{\text{PbI}_2} - \rho_{\text{ligand}}$$

where ρ_{complex}, ρ_{PbI_2} and ρ_{ligand} represent the electronic charge densities of the optimized complex and its constituent isolated fragments, evaluated at the frozen coordinates extracted from the fully relaxed complex geometry. We have revised the caption of Fig. S8 and added clarifying text within the main manuscript to prevent any potential reader misunderstanding.

Finally, we cross-checked our recalculated trends against established literature regarding PbI_2–solvent interactions. Our models show that DMSO binds significantly more strongly to PbI_2 than DMF, which perfectly matches prior reports attributing this behavior to the higher Lewis basicity and superior coordination capacity of the sulfoxide group. While absolute binding energy values are known to shift depending on the choice of exchange-correlation functional, basis set/plane-wave cutoff, dispersion corrections, and implicit solvation treatments, the robust relative trend obtained here confirms that our revised calculations are physically sound and well within a reasonable range. This comparative discussion has been added to the revised manuscript and Supplementary Information.

As suggested, the following sentences were added on pages 5-6 in the Supporting information:

"*Density functional theory calculations.* First-principles computations were carried out using the Vienna Ab initio Simulation Package (VASP) based on density functional theory (DFT). The exchange-correlation interaction was described using the generalized gradient approximation (GGA) parameterized by the Perdew-Burke-Ernzerhof (PBE) functional. Projector-augmented wave (PAW) pseudopotentials were adopted to describe ion–electron interactions with a plane-wave cutoff energy of 500 eV. The electronic energy and ionic forces were converged to within 10^{-5} eV and 0.03 eV $Å^{-1}$, respectively. A vacuum spacing of 15 Å was applied perpendicular to the slab to avoid unphysical periodic interlayer interactions. For each PbI_2–ligand system (including DMF, DMSO, FA^+, and $FPGC^+$), multiple initial binding configurations featuring distinct coordination sites and molecular orientations were constructed and fully relaxed. The configuration yielding the lowest total energy was selected for binding-energy comparison (displayed in Supplementary Fig. 5).

The binding energy (ΔE_{bind}) was calculated using the following equation:

$$\Delta E_{\mathrm{bind}} = E_{\mathrm{complex}} - E_{\mathrm{PbI_2}} - E_{\mathrm{ligand}}$$

where E_{complex}, $E_{\mathrm{PbI_2}}$ and E_{ligand} represent the total energies of the optimized PbI_2-ligand complex, the isolated PbI_2 slab, and the isolated ligand, respectively. The PbI_2-DMF, PbI_2–DMSO, PbI_3^-–FA^+, and PbI_3^-–$FPGC^+$ systems were modeled as net-neutral configurations. For the charged species (FA^+, $FPGC^+$, PbI_2–FA^+, and PbI_2–$FPGC^+$), the net charge of the simulation cell was set to +1 by reducing the total electron account accordingly.

The three-dimensional charge density difference ($\Delta\rho(r)$) was calculated as:

$$\Delta\rho(r) = \rho_{\mathrm{complex}} - \rho_{\mathrm{PbI_2}} - \rho_{\mathrm{ligand}}$$

where $\rho_{complex}$, ρ_{PbI_2} and ρ_{ligand} are electronic charge densities of the optimized complex and its constituent isolated fragments, respectively, with the fragments maintaining the exact spatial coordinates of the fully relaxed complex geometry."

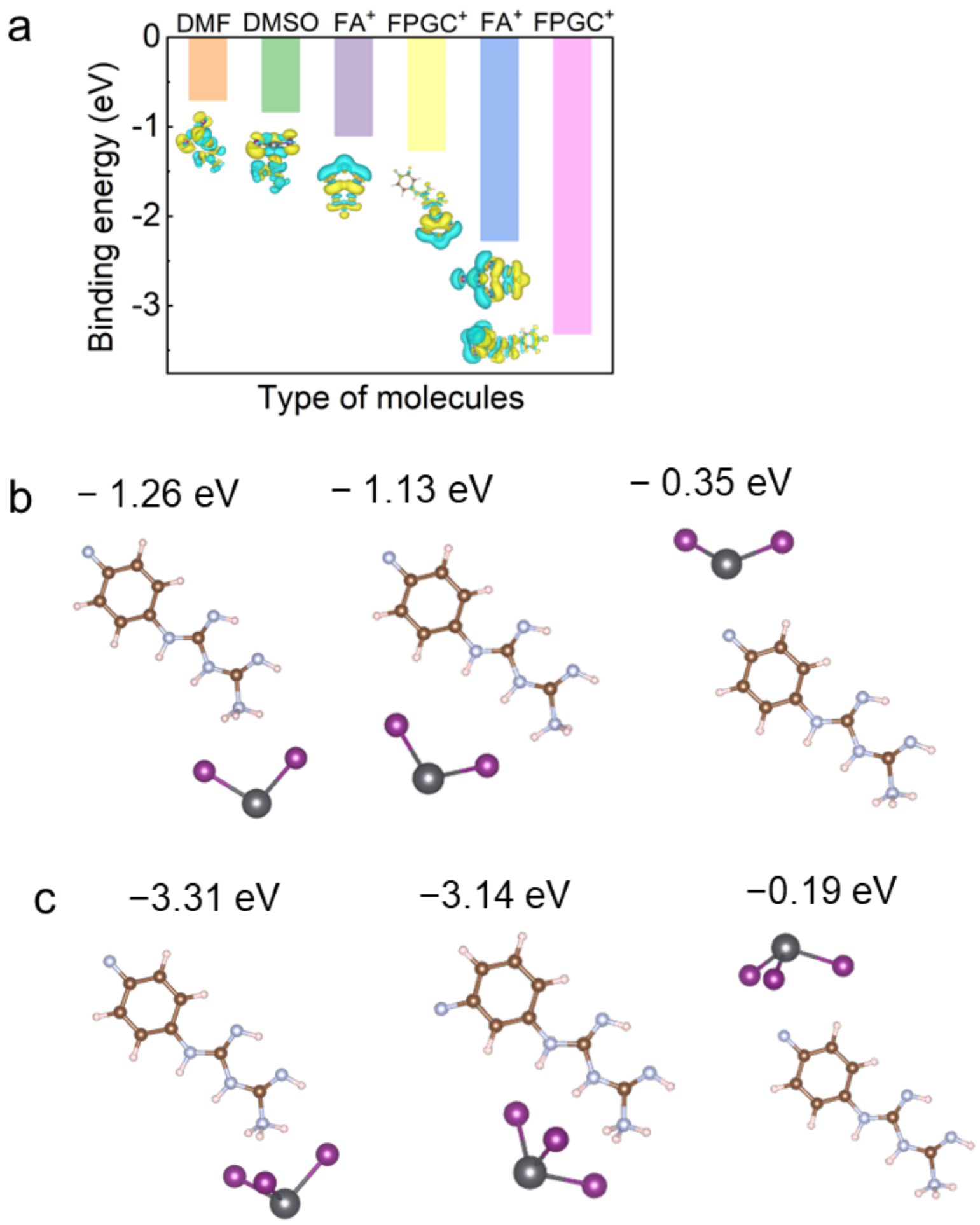

Figure S8. Density functional theory calculations of ligand-perovskite interactions. a) Calculated binding energies and three-dimensional charge density difference profiles for PbI_2 interacting with DMF, DMSO, FA^+ and $FPGC^+$ ligands, along with the binding energies and electrostatic potential maps of the FA^+–PbI_3^- and $FPGC^+$–PbI_3^- ion-pair systems. The yellow and cyan isosurfaces denote electron accumulation and depletion regions, respectively, evaluated at a constant isosurface value of 0.0004 e Å^{-3}. b) Distinct fully relaxed binding configurations optimized for the PbI_2–$FPGC^+$ coordination complex. c) Distinct fully relaxed binding configurations optimized for the PbI_3^- and $FPGC^+$ ion pair.

On pages 15-16 in the revised supporting information: “To evaluate the molecular interaction strengths governing the precursor chemistry, density functional theory (DFT) calculations were carried out to determine the binding energies between PbI_2 and various species, including DMF, DMSO, FA^+, and $FPGC^+$ (Supplementary Fig. 8). To ensure a rigorous comparison, multiple initial coordination geometries were examined for the FA^+–PbI_3^- and $FPGC^+$–PbI_3^- configurations, from which the lowest-energy states were selected for binding-energy calculations and three-dimensional charge density difference analyses. The resulting charge density difference profiles revealed pronounced charge redistribution localized at the PbI_2–$FPGC^+$ interface, indicating strong electronic coupling between the $FPGC^+$ cation and the PbI_2 framework. The calculated binding energy between $FPGC^+$ and PbI_2 was found to be −1.26 eV, which is significantly stronger than those calculated for PbI_2–DMF (−0.70 eV), PbI_2–DMSO (−0.83 eV), and PbI_2–FA^+ (−1.10 eV). Although absolute binding energies can exhibit sensitivity to the specific computational framework, exchange-correlation functional, plane-wave cutoff, dispersion corrections, and implicit solvation treatments, the relative trend established here is in excellent agreement with established literature on PbI_2–solvent interactions, validating the physical reliability of our models.

Given that PbI_2 readily coordinates with iodide ions to form iodoplumbate coordinates (such as PbI_3^-) within the complex precursor solution environment, the binding energies of the FA^+–PbI_3^- and $FPGC^+$–PbI_3^- ion-pair systems were further investigated. The binding energy of the $FPGC^+$–PbI_3^- assembly was determined to be −3.31 eV, which substantially exceeds that of the FA^+–PbI_3^- counterpart (−2.27 eV). This disparity confirms the superior thermodynamic affinity of the $FPGC^+$ cation toward the iodoplumbate framework. Collectively, these computational findings demonstrate that $FPGC^+$ can more effectively coordinate with plumbate–iodide components to guide the assembly of the transient $FPGC_2PbI_4$ intermediate phase, thereby successfully suppressing uncontrolled, amorphous phase segregation during the initial stage of perovskite crystallization.”

Table S2. Literature comparison of binding energies for ligand–perovskite interactions.

Complexes	Binding energy (eV)	Ref
DMF–PbI_2	-0.79	9
FA^+–PbI_3^-	-1.00	9
DMSO–PbI_2	-0.906	10
DMSO–PbI_2	-1.01	11
DMSO–PbI_2	-0.68	12
DMSO–PbI_2	-0.2	13
FA^+–PbI_2	-6.1	13
FA^+–PbI_3^-	-6.6	14

New references are added in the Supporting information:

9. Li F*, et al.* Hydrogen-bond-bridged intermediate for perovskite solar cells with enhanced efficiency and stability. *Nat. Photonics* **17**, 478-484 (2023).

10. Liu J, Li M, Ji J, Zhao Z, Li M. Mild coordination enabled by steric hindrance facilitates fabrication of large-area perovskite solar modules. *Adv. Mater.* **38**, e21181 (2026).

11. Chen R*, et al.* Reduction of bulk and surface defects in inverted methylammonium- and bromide-free formamidinium perovskite solar cells. *Nat. Energy* **8**, 839-849 (2023).

12. Zhao X*, et al.* Regulation of buried interface through the rapid removal of PbI_2·DMSO complex for enhancing light stability of perovskite solar cells. *ACS Energy Lett.* **9**, 2659-2669 (2024).

13. Pan T*, et al.* Surface-energy-regulated growth of *α*-phase $Cs_{0.03}FA_{0.97}PbI_3$ for highly efficient and stable inverted perovskite solar cells. *Adv. Mater.* **35**, 2208522 (2023).

14. Tian L*, et al.* Divalent cation replacement strategy stabilizes wide-bandgap perovskite for Cu(In,Ga)Se_2 tandem solar cells. *Nat. Photonics* **19**, 479-485 (2025).

4. Given that the binding strength between $FPCG^+$ and PbI_2 far exceeds that of FA^+ and PbI_2, why does $FPGC_2PbI_4$ decompose at around 80 °C? This temperature is significantly lower than the thermal decomposition temperature of 3D perovskites, presenting a logical contradiction that directly affects the core conclusion of this work.

Authors Response:

We thank the reviewer for this insightful comment. We completely agree that our original wording lacked sufficient precision regarding the thermal stability of the intermediate phase. A robust binding interaction between the $FPGC^+$ cation and the PbI_2 framework facilitates the initial assembly of the layered intermediate at low temperatures, but it does not imply that this $FPGC_2PbI_4$ phase must remain the thermodynamically preferred or long-range-ordered phase at elevated temperatures.

To definitively clarify the fate of this phase upon thermal treatment, we have performed thermogravimetric analysis (TGA) of the neat FPGC ligand, which reveals a high decomposition temperature of 217 °C (now included as Fig. S3). Because this decomposition threshold is substantially higher than the temperature at which the low-angle X-ray diffraction (XRD) reflections of the $FPGC_2PbI_4$ intermediate vanish (~80 °C), the loss of this characteristic layered feature cannot be attributed to the chemical degradation of the FPGC molecule.

Instead, the disappearance of the low-angle reflections signifies a loss of long-range crystallinity and an irreversible structural conversion of the transient $FPGC_2PbI_4$ matrix during film annealing. This structural evolution is entirely consistent with previous literature demonstrating that low-dimensional or Ruddlesden–Popper-like perovskite phases can undergo thermally driven lattice reconstruction, mixed-phase transformations, or localized amorphization under active heating, rather than remaining

permanently locked as highly ordered layered structures (DOI: 10.1021/acs.nanolett.0c01271; 10.1002/adma.202204726). Furthermore, this behavior perfectly mirrors recent high-impact reports demonstrating that two-dimensional (2D) perovskite phases can function strictly as transient intermediates that structurally collapse and disappear in the final annealed film after successfully template-guiding oriented three-dimensional (3D) crystallization (DOI: 10.1038/s41563-024-02073-x).

Accordingly, we have thoroughly revised the discussion within the manuscript to explicitly clarify that the phase observed below ~80 °C is a transient $FPGC_2PbI_4$ intermediate, and that its disappearance at elevated temperatures reflects a controlled structural phase conversion rather than molecular decomposition.

As suggested, the following sentences were revised on pages 5-6 in the revised manuscript: "Upon elevating the temperature above 80 °C, the layered 2D structure collapses, as evidenced by the disappearance of the low-angle reflection and the concomitant emergence of the PbI_2 characteristic peak at $2\theta = 12.7°$. Thermogravimetric analysis (TGA) confirms that the isolated FPGC molecule remains stable up to 217 °C (Figure S3); thus, the dissolution of the 2D XRD features above 80 °C does not stem from molecular decomposition, but rather from a loss of long-range crystalline order within the $FPGC_2PbI_4$ framework."

As suggested, the following sentences were added on page 9 in the Supporting information: "Thermogravimetric analysis (TGA) demonstrated that the FPGC ligand decomposes at 217 °C, a temperature substantially higher than that at which the low-angle X-ray diffraction (XRD) reflections of the $FPGC_2PbI_4$ phase disappear (~80 °C). Consequently, the loss of this characteristic layered diffraction feature cannot be attributed to the chemical degradation of FPGC. Instead, it signifies an irreversible structural conversion of the long-range-ordered layered phase upon thermal treatment. In layered hybrid halide perovskites, thermally induced disorder within the organic sublattice can couple strongly to the inorganic framework, thereby driving structural phase transitions well below the decomposition temperature of the isolated organic

component [1–3]. The concurrent emergence of PbI_2 reflections further indicates that this transformation exceeds a simple, reversible order–disorder transition, involving the complete collapse of the layered hybrid framework [3,4]. This behavior aligns with recent literature demonstrating that a two-dimensional (2D) perovskite phase formed during solution processing can act as a transient intermediate, which subsequently disappears in the final annealed film after successfully guiding oriented three-dimensional (3D) crystallization [5]."

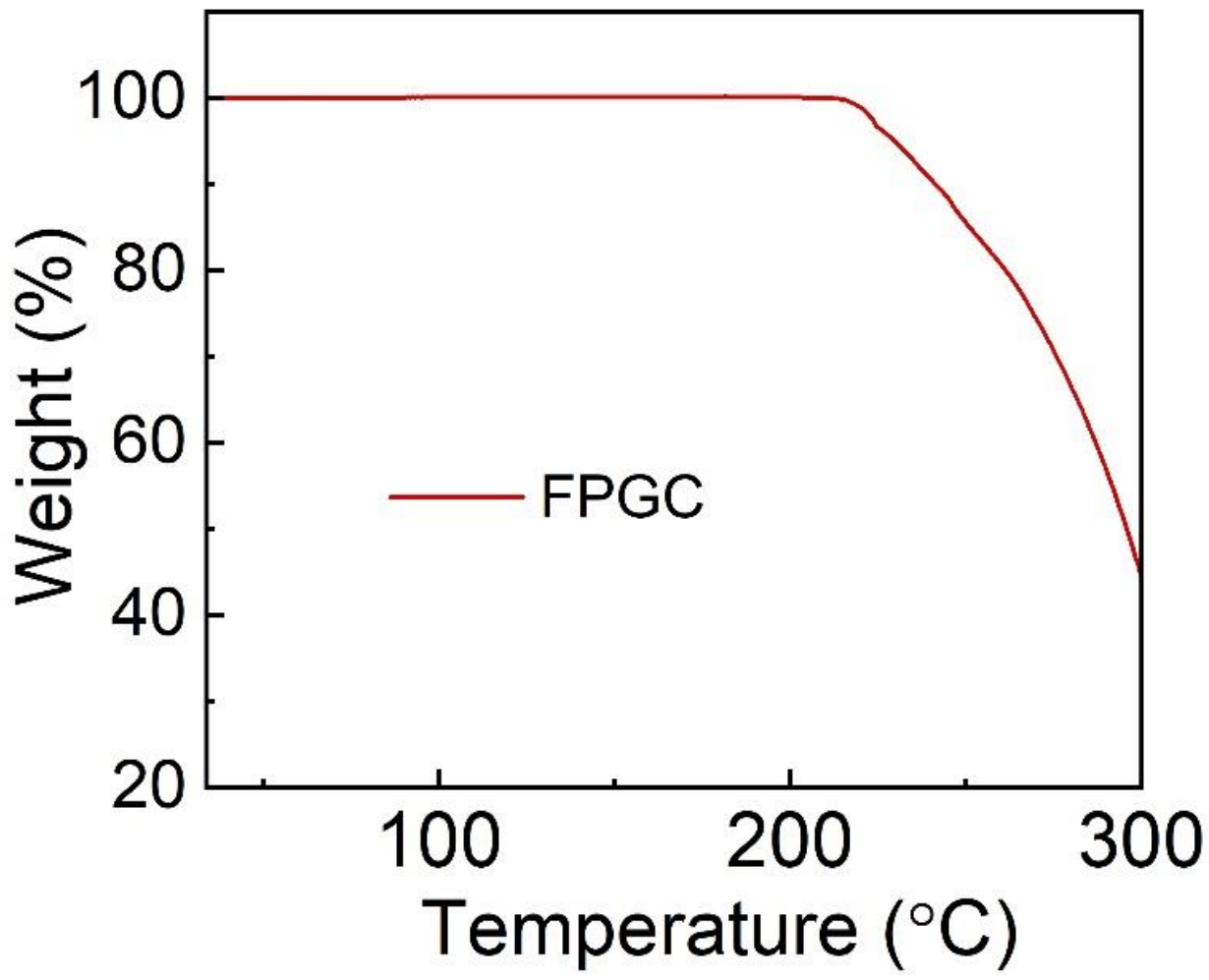

Figure S3. Thermogravimetric analysis profile of the neat FPGC molecular powder.

New references are added in the Supporting information:

1. Dahod NS, Paritmongkol W, Stollmann A, Settens C, Zheng S-L, Tisdale WA. Melting transitions of the organic subphase in layered two-dimensional halide perovskites. *J. Phys. Chem. Lett.* **10**, 2924-2930 (2019).

2. Menahem M*, et al.* Strongly anharmonic octahedral tilting in two-dimensional hybrid halide perovskites. *ACS Nano* **15**, 10153-10162 (2021).

3. Sutanto AA*, et al.* In situ analysis reveals the role of 2D perovskite in preventing thermal-induced degradation in 2D/3D perovskite interfaces. *Nano Lett.* **20**, 3992-3998 (2020).

4. Perini CAR*, et al.* Interface reconstruction from Ruddlesden-Popper structures impacts stability in lead halide perovskite solar cells. *Adv. Mater.* **34**, 2204726 (2022).

5. Liu Z*, et al.* All-perovskite tandem solar cells achieving >29% efficiency with

improved (100) orientation in wide-bandgap perovskites. *Nat. Mater.* **24**, 252-259 (2025).

5. The manuscript states that $FPGC_2PbI_4$ decomposes into PbI_2 and organic species at 80°C; do the bulky organic species remain in the lattice or are they expelled to the surface? Why do TOF-SIMS results show a stronger F signal in the bulk and bottom regions, while HRTEM shows identical lattice spacing at the surface, bulk, and bottom? Additionally, the HRTEM images of the control group are not shown. Does the non-uniform distribution of organic species in the bulk affect the long-term stability of the device?

Authors Response:

We thank the reviewer for this important comment. We agree that the spatial distribution and chemical fate of the FPGC species following the intermediate phase conversion, along with their impact on long-term stability, warrant explicit clarification. We have addressed these points through several additional characterizations:

Time-of-flight secondary-ion mass spectrometry (ToF-SIMS) depth profiles confirm that the FPGC species remain distributed throughout both the surface and bulk regions of the annealed perovskite film. To clarify their local distribution, we have included new two-dimensional (2D) ToF-SIMS chemical maps of fluorine (F^-) and lead (Pb^{2+}) in **Fig. S13**. These maps demonstrate a clear local enrichment of FPGC species at the grain-boundary regions rather than a complete expulsion to the top surface. This localized grain-boundary passivation explains why the macroscopic ToF-SIMS profile records a strong fluorine signature throughout the bulk and bottom regions, while high-resolution transmission electron microscopy (HRTEM) captures the pristine, unperturbed lattice spacing of the core 3D perovskite grains across all film depths.

Following the reviewer's suggestion, we have added comparative cross-sectional HRTEM images of the control film in **Fig. S17**. Unlike the target film—which maintains uniform lattice parameters throughout its entire thickness—the control film

exhibits a gradient of increasing lattice spacing from the top surface to the buried substrate interface. This direct comparison confirms that the target film possesses a more structurally homogeneous 3D framework, successfully mediated by the locally distributed grain-boundary FPGC species.

To address the concern regarding long-term structural and operational stability, we performed post-mortem ToF-SIMS depth profiling on the target devices after 100 h of continuous operation under simulated solar illumination. As illustrated in **Fig. S38**, the vertical fluorine distribution remains unchanged compared to the pristine device state. When combined with the excellent long-term operational stability tracking presented in **Fig. 5**, these data confirm that the localized distribution of the FPGC species remains configurationally stable and does not induce degradation or negatively impact device performance during operation.

As suggested, the following sentences were revised on page 9, lines 16-21 in the revised manuscript: "<u>Following full thermal annealing, full-survey X-ray photoelectron spectroscopy (XPS) and time-of-flight secondary ion mass spectrometry (ToF-SIMS) verify that FPGC-derived chemical species are completely retained within the final thin film. Crucially, cross-sectional and two-dimensional ToF-SIMS mapping reveals a localized chemical enrichment at the bulk grain boundaries (Figures S11-13).</u>"

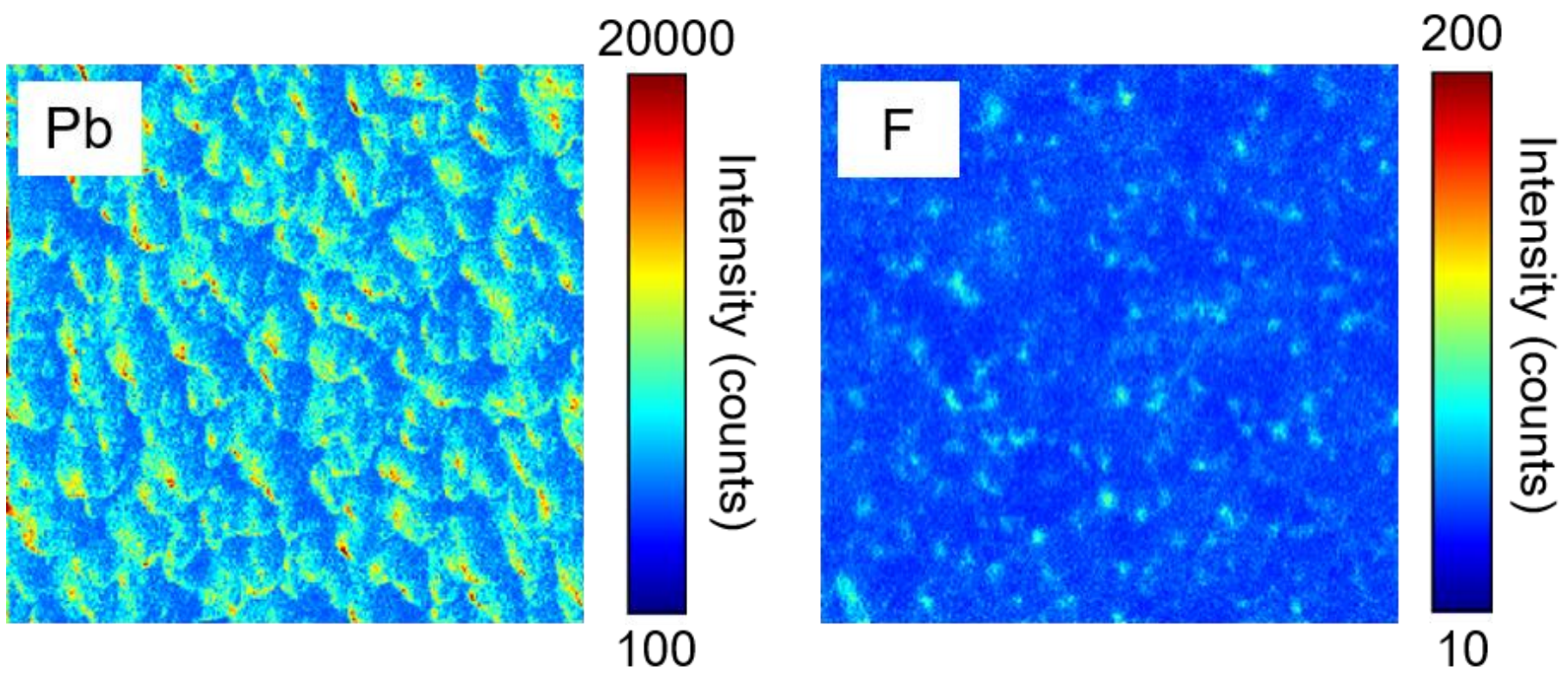

Figure S13. The ToF-SIMS 2D imaging of Pb and F distribution on the FPGC-contained perovskite layer.

On page 12, lines 1-2: "In contrast, the control film displays a gradual increase in interplanar spacing from the surface (3.17 Å) to the bottom (3.19 Å) (Figure S17), suggesting depth-dependent lattice distortion."

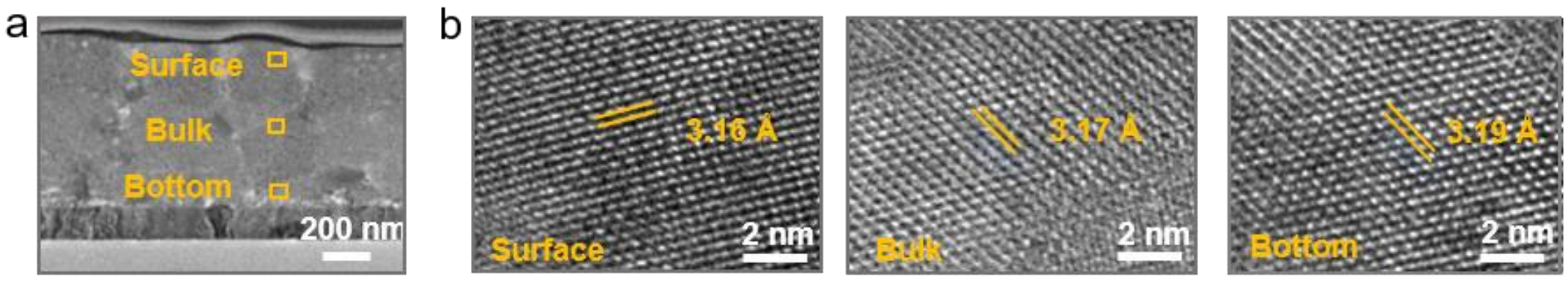

Figure S17. a) The cross-sectional SEM image of the control perovskite film. b) The dextral lattice fringe images collected from the surface, the bulk, and the bottom regions in a).

On pages 17-18: "To examine whether the spatial distribution of the FPGC-derived species evolves during operation, we performed ToF-SIMS depth profiling on devices after 100 h of continuous operation (Figure S38). The vertical fluorine (F^-) signal distribution shows no appreciable displacement relative to that of the pristine device, indicating that the FPGC-derived species remain configurationally stable during operational stress."

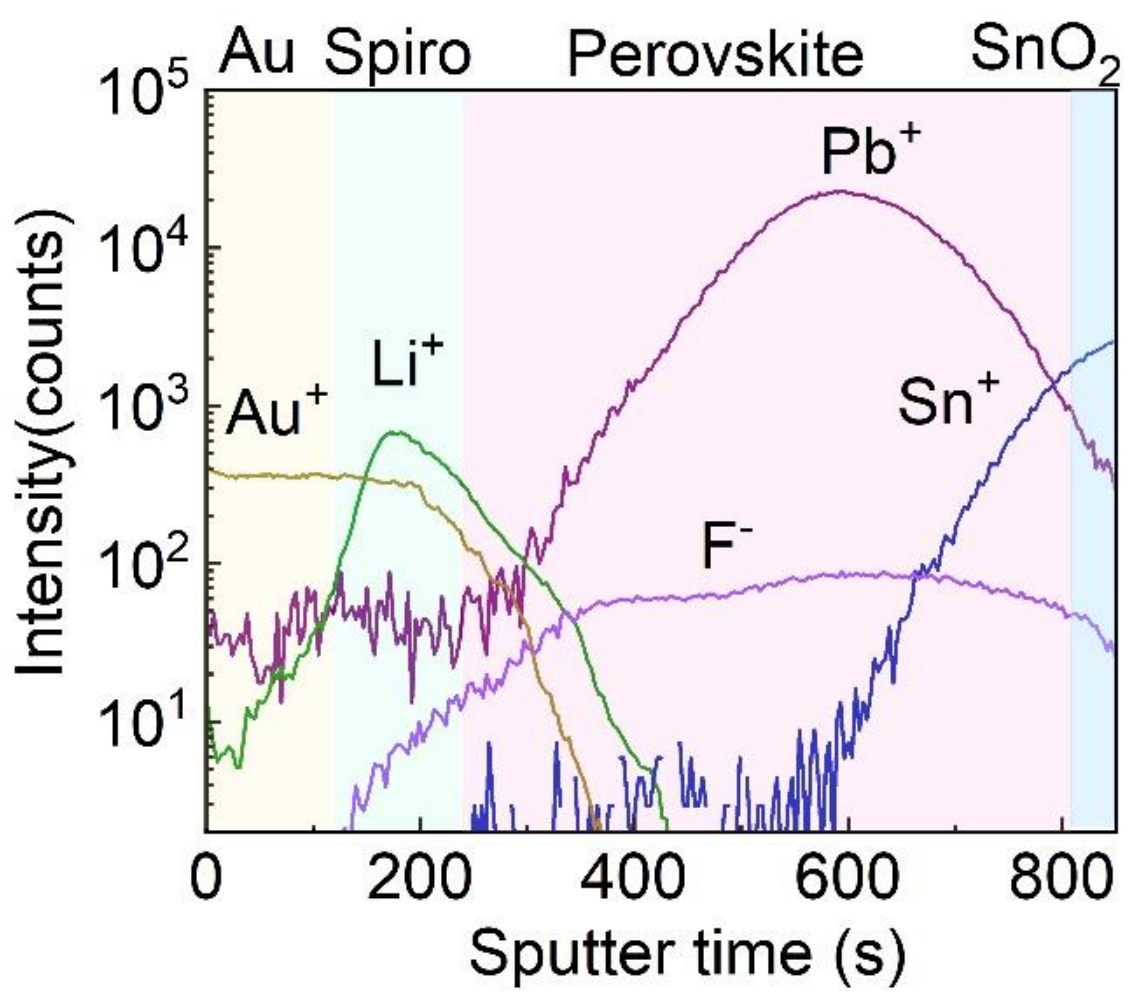

Figure S38. TOF-SIMS spectra of the target devices after 100 h of operation.

6. Figure S9 shows a significant shift in XRD peak positions between the control and

experimental groups; please explain this.

Authors Response:

We thank the reviewer for highlighting this important structural feature. As illustrated in **Fig. S14**, the primary (110) diffraction peak of the target perovskite film undergoes a distinct shift toward a higher scattering angle, moving from 13.975° (control) to 14.007° (target). According to Bragg's law, this displacement signifies a reduction in the interplanar *d*-spacing, confirming a slight lattice contraction within the target film framework.

We attribute this structural modulation to the distinct FPGC-mediated crystallization pathway, which guides the assembly of a more highly packed and compact final lattice state. Such a pre-contracted lattice framework is highly beneficial for mitigating subsequent photo- and thermally induced lattice expansion during operation, thereby enhancing long-term structural and device stability (DOI: 10.1038/s41566-025-01808-9). We have incorporated this explicit discussion into the revised manuscript and Supporting Information.

As suggested, the following sentences were revised on page 23 in the Supporting information: "As illustrated in Supplementary Fig. 14, the primary (110) diffraction peak of the perovskite film undergoes a distinct shift toward a higher scattering angle, moving from 13.975° for the control film to 14.007° for the target film. According to Bragg's law, this displacement signifies a reduction in the corresponding interplanar *d*-spacing, confirming a slight lattice contraction within the target film framework. This structural modulation is attributed to the altered crystallization pathway mediated by the FPGC ligand, which guides the assembly of a more highly packed and compact final lattice state relative to the control matrix. Such a pre-contracted lattice framework is expected to play a critical role in mitigating subsequent light- and thermally induced lattice expansion during operation, thereby contributing to the enhanced long-term structural robustness of the device [15]."

7. Are the XRD signals evolving with light soaking time in Figure 3A based on ex-situ or in-situ/quasi-in-situ measurements? If they are ex-situ signals, the reliability of this result is significantly compromised.

Authors Response:

We thank the reviewer for this important question. We have clarified the experimental setup and provided statistical validation to substantiate the reliability of our findings:

The X-ray diffraction (XRD) profiles presented in **Fig. 3a** were captured via real-time *in situ* light-soaking measurements. The perovskite film was maintained inside the sealed XRD chamber under continuous simulated 1-sun illumination (100 mW cm^{-2}) while diffraction patterns were collected sequentially as a function of exposure time. Therefore, the observed peak displacement directly reflects the dynamic, real-time structural response of the identical film matrix under photoexcitation. This reversible phenomenon is well aligned with recent high-impact reports demonstrating light-induced lattice expansion and subsequent dark recovery in metal halide perovskites (DOI: 10.1038/s41586-024-08161-x; 10.1038/s41566-025-01808-9; 10.1126/science.adu5563; 10.1126/science.adu5563).

To verify the reproducibility of this behavior, we repeated the *in situ* light/dark cycling XRD measurements across five independent samples. All replicates consistently exhibited photoinduced lattice expansion and subsequent structural recovery, with the statistical error bars now presented in **Fig. 3b**.

Furthermore, depth-dependent grazing-incidence XRD (GIXRD) profiling was performed to capture the spatial variations of this response after 70 min of continuous illumination and a subsequent 5 h dark storage period. For the control film, continuous illumination induced pronounced line broadening and peak shifts across different incident angles, signaling vertical strain heterogeneity and an inhomogeneous, depth-dependent lattice deformation. Specifically, the depth-dependent variation in *d*-spacing (Δd) evolved from 0.0635 Å in the pristine state to 0.0182 Å after light exposure, and

partially recovered to 0.0566 Å after dark storage. In sharp contrast, the target film exhibited negligible structural variations across all evaluation states, confirming that the optimized vertical framework successfully suppresses light-induced lattice perturbations.

As suggested, the following sentences were revised on page 13, lines 9-12 in the revised manuscript: "Depth-dependent GIXRD further confirmed that light/thermal soaking induces a pronounced vertical lattice deformation profile in the control film, whereas this light- and heat-induced strain heterogeneity is substantially suppressed in the target film (Figure S20)"

The caption of Figure 3 in the revised manuscript has been revised as follows in the revised manuscript: "**Figure 3. Lattice dynamics and defect evolution under cyclic illumination.** A) In-situ XRD patterns of the control and target films recorded under continuous one-sun illumination (100 mW cm^{-2}) and following dark storage. B) Variation in the (100) interplanar spacing extracted from the XRD analysis over 20 light-dark cycles. Error bars represent the standard deviation derived from five independent film measurements."

As suggested, the following sentences were added on pages 29-30 in the Supporting information: "To investigate the spatial variations in structural dynamics, depth-dependent grazing-incidence X-ray diffraction (GIXRD) measurements were conducted to evaluate the vertical lattice response of the control and target films during light/dark cycling. The films were continuously illuminated for 70 min under simulated 1-sun intensity (100 mW cm^{-2}) at ~ 55 °C and subsequently stored in the dark for 5 h paired with cooling to room temperature. For the control film, continuous illumination with temperature increase induced pronounced diffraction peak shifts and localized line broadening across various incident angles, providing clear evidence of an inhomogeneous, depth-dependent lattice deformation and increased strain heterogeneity. The corresponding interplanar spacing was calculated using Bragg's law. The depth-dependent variation in *d*-spacing (Δd) evolved from 0.0635 Å in the pristine

state to 0.0182 Å after light exposure, and partially recovered to 0.0566 Å after dark storage. This cyclical evolution demonstrates that the control film undergoes significant light- and heat-induced structural distortion followed by only partial lattice relaxation in the dark. In sharp contrast, the target film exhibited negligible variations in both peak positions and Δ*d* values under identical cycling protocols. This structural stability demonstrates that the enhanced, uniform crystallographic orientation across the entire film depth effectively suppresses light- and heat-induced lattice perturbations and eliminates vertical strain redistribution."

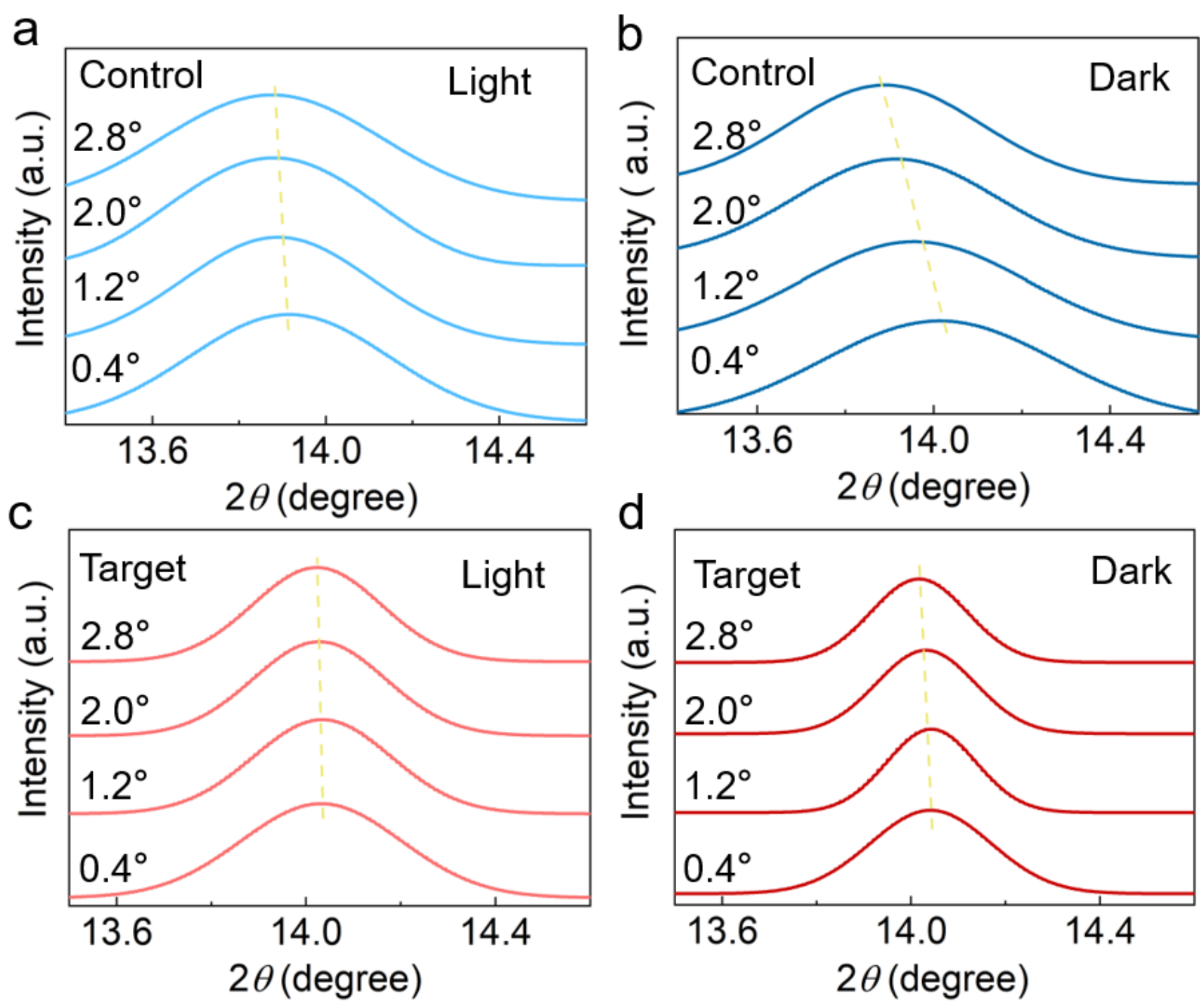

Figure S20. GIXRD of perovskite films under different states. a) GIXRD patterns of the control film first exposure to light for 70 minutes at ~ 55 °C. b) GIXRD patterns of the control film after transfer to dark conditions. c) GIXRD patterns of the target film first exposure to light for 70 minutes at ~ 55 °C. d) GIXRD patterns of the target film after transfer to dark conditions.

8. The surface GIWAXS signal intensity in Figure S10 is notably higher than the bulk GIWAXS signal in Figure 2, which is evidently caused by the inconsistent color bar ranges used, and this is misleading to readers.

Authors Response:

We thank the reviewer for this constructive and important comment. We agree that employing divergent color bar ranges between the figures could inadvertently obscure the true relative signal intensities.

Following the reviewer's excellent recommendation, we have reprocessed all grazing-incidence wide-angle X-ray scattering (GIWAXS) patterns using a fully unified color scale (intensity range: 0–2750) to facilitate a reliable, direct visual comparison. As the incident angle decreases from 1.0° (bulk-sensitive) to 0.1° (surface-sensitive), the total scattering volume is reduced, making the near-surface GIWAXS signal inherently weaker. Standardizing the intensity scale across these different probing depths allows for a completely transparent evaluation of the crystallographic features and orientation distributions.

Crucially, this graphical correction does not alter the fundamental conclusions drawn from our structural analysis: the target film consistently exhibits an enhanced, preferential out-of-plane (100) crystallographic orientation throughout both its near-surface and bulk regions. The updated panels have been incorporated into the revised **Fig. 2** and **Fig. S15**.

As suggested, Figures S15 and S16 have been updated accordingly in the revised Supplementary Information.

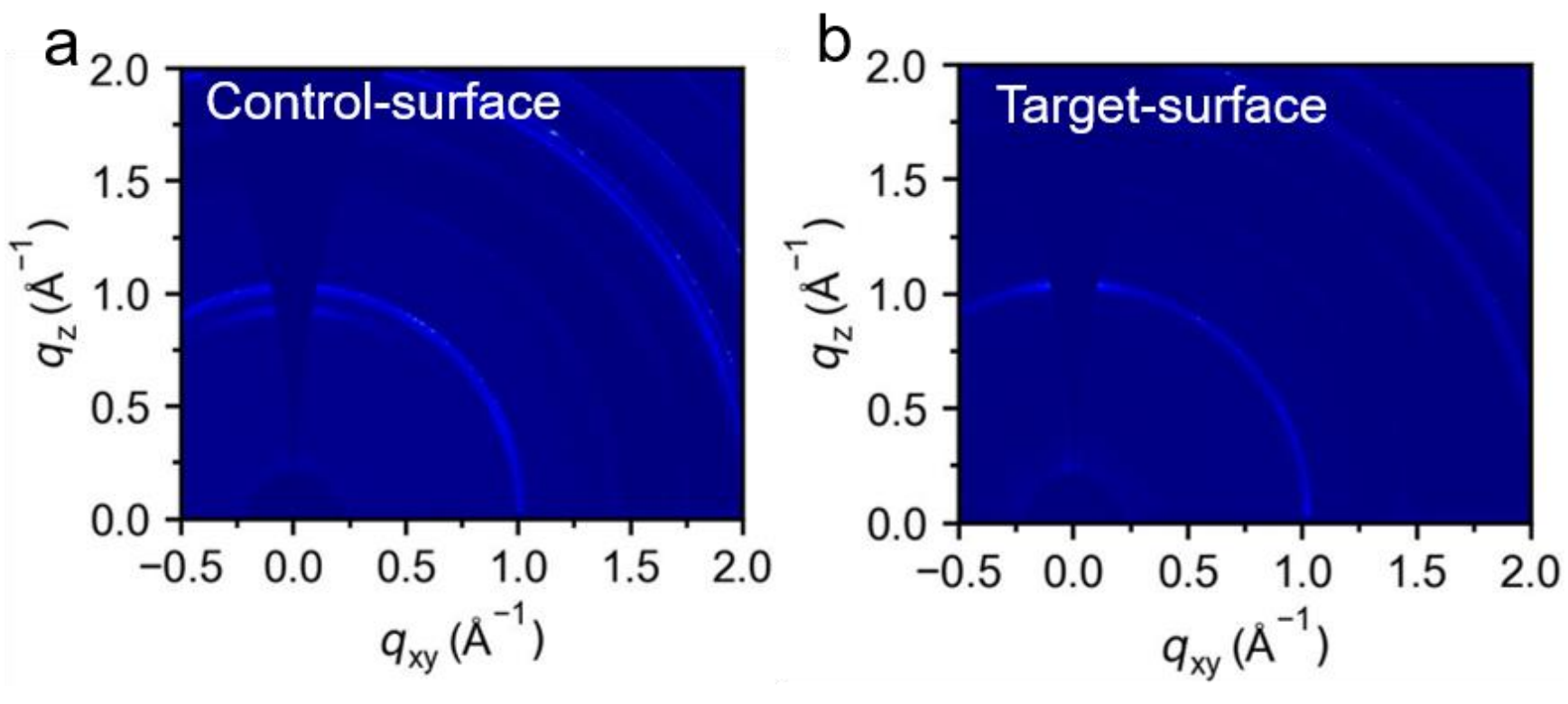

Figure S15. 2D GIWAXS patterns of the b) control and c) target perovskite films with incident angle of 0.1°, respectively.

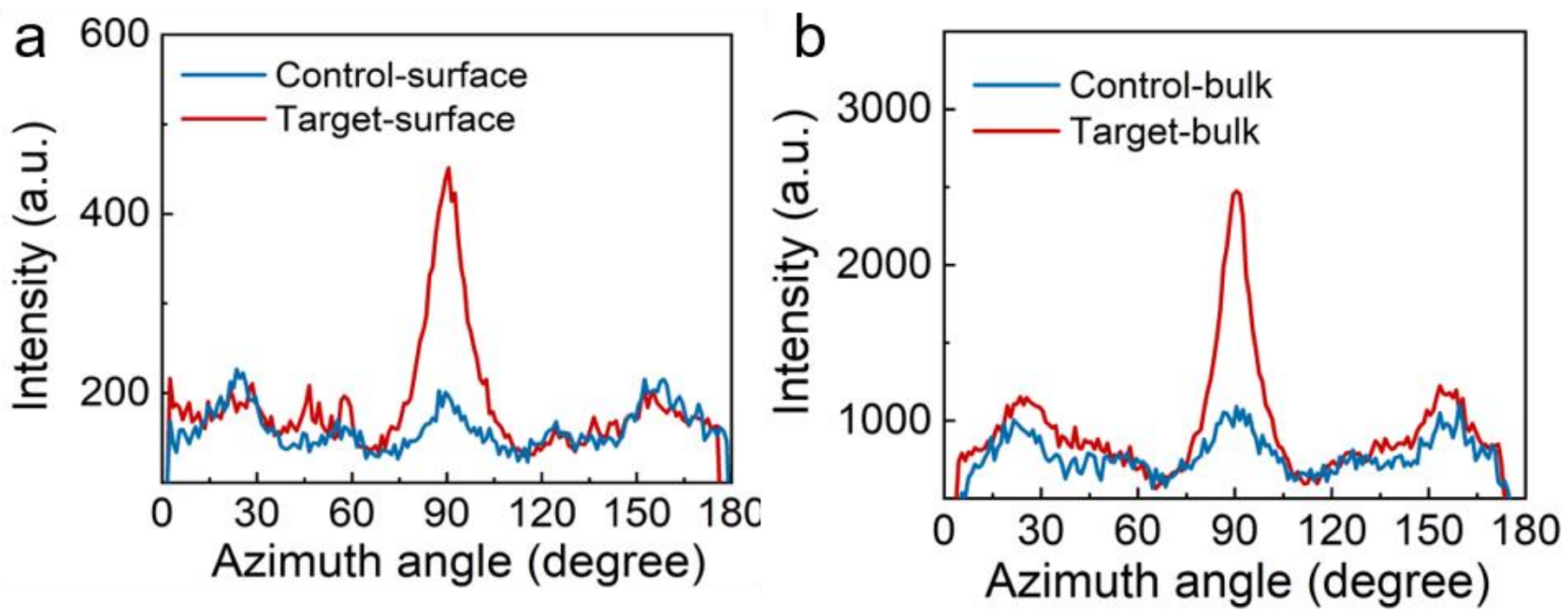

Figure S16. Integrated intensity plots azimuthally along the ring at q_{xy} = 10 nm^{-1}.

9. The reported efficiency difference between the target and control devices is excessively large, suggesting that may the efficiency of the control device may have been artificially lowered. Furthermore, the target device shows a significant improvement in V_{OC} compared to the control, yet the authors have offered no explanation for this.

Authors Response:

We thank the reviewer for this critical comment. We have addressed this concern by re-optimizing the baseline fabrication protocols and providing a detailed thermodynamic voltage-loss analysis to clarify the operational mechanism:

In the revised study, we further optimized the processing conditions for both the control and target devices. Consequently, the control devices exhibited a significantly improved average power conversion efficiency (PCE), establishing a more rigorous and representative baseline for comparison. Even against this enhanced reference matrix, the target devices consistently maintain a distinct, reproducible efficiency advantage.

To pinpoint the exact physical mechanism driving the open-circuit voltage (V_{OC}) enhancement, we performed photoluminescence quantum yield (PLQY) and light-intensity-dependent V_{OC} measurements on the re-optimized systems. The target configuration exhibited a markedly superior PLQY relative to the control across both neat films (13.21% versus 2.63%) and multi-layer device stacks (5.82% versus 1.06%).

Incorporating the FPGC ligand increased the calculated QFLS from 1.258 to 1.301 eV for the neat films, and from 1.235 to 1.280 eV for the full multi-layer architectures. Deconvolution of the non-radiative voltage drops revealed that the bulk-perovskite loss was significantly reduced from 94.0 to 52.5 meV, while the contact interfacial loss decreased from 23.2 to 21.0 meV. This confirms that the V_{OC} gains originate predominantly from suppressed bulk recombination, supplemented by a minor mitigation of interfacial recombination. Corroborating this trend, the diode ideality factor (n) extracted from the light-intensity-dependent V_{OC} curves dropped from 1.80 k_BT/q to 1.29 k_BT/q. This substantial reduction provides direct kinetic evidence of the successful suppression of trap-assisted recombination pathways.

These rigorous optoelectronic evaluations have been fully integrated into the revised manuscript (accompanied by **Fig. S35**), providing a self-contained, quantitative explanation for the enhanced V_{OC} in our target solar cells.

As suggested, the following sentences were revised on page 16, lines 7-14 in the revised manuscript: "To quantitatively elucidate the mechanism underlying the open-circuit voltage (V_{OC}) enhancement, photoluminescence quantum yield (PLQY) measurements were performed on the neat perovskite films and multi-layer device stacks under 1-sun-equivalent photoexcitation. These values were subsequently used to evaluate the quasi-Fermi level splitting (QFLS). To extract the QFLS values from the PLQY data, the dark radiative recombination current density ($J_{0,rad}$) of the device configuration must be established. The baseline flux of blackbody photons (ϕ_{BB}) was calculated according to the Planck radiation law:

$$\phi_{BB} = \frac{1}{4\pi^2\hbar^3c^2}\frac{E^2}{\exp\left(\frac{E}{k_BT}\right) - 1}$$

where $\hbar$ is the reduced Planck constant, c is the speed of light in vacuum, E represents photon energy, k_B is the Boltzmann constant, and T denotes the absolute temperature. The value of $J_{0,rad}$ was then determined by integrating the blackbody photon flux with the experimental external quantum efficiency (EQE) spectrum:

$$J_{0,rad} = q\int EQE(E)\phi_{BB}(E)dE$$

where q represents the elementary electron charge. The thermodynamic radiative limit of the quasi-Fermi level splitting ($QFLS_{rad}$) was evaluated using the following relation:

$$QFLS_{rad} = k_B T \ln\left(\frac{J_G}{J_{0,rad}}\right)$$

where J_G represents the generation current density, which is approximated by the short-circuit current density (J_{SC}). Subsequently, the actual, non-ideal QFLS value under open-circuit conditions was calculated via:

$$\text{QFLS} = QFLS_{rad} + k_B T \ln(PLQY)$$

The non-radiative voltage loss originating from the bulk perovskite film ($\Delta E_{nr,bulk}$) is defined as [5]:

$$\Delta E_{nr,bulk} = QFLS_{rad} - QFLS_{film} = -k_B T \ln\left(PLQY_{film}\right)$$

The non-radiative voltage loss associated with interfacial recombination (ΔE_{int}) across the contact boundaries is given by:

$$\Delta E_{int} = QFLS_{film} - QFLS_{stack}$$

Finally, the remaining non-radiative voltage loss occurring within the operational device layout (ΔE_{device}), often associated with non-ideality factors and resistive extraction boundaries, is defined as:

$$\Delta E_{device} = QFLS_{stack} - qV_{OC}$$

To elucidate the physical origin of the enhanced V_{OC}, we evaluated the photoluminescence quantum yield (PLQY) of the neat perovskite films and the corresponding multilayer device stacks (Figure S35a). The PLQY of the neat target film increases markedly from 2.63% (control) to 13.21%, corresponding to a substantial expansion in quasi-Fermi level splitting (QFLS) from ~ 1.258 to ~ 1.301 eV. This substantial enhancement confirms a profound suppression of bulk non-radiative recombination within the target matrix. For the complete multilayer device stacks, the PLQY similarly rises from 1.06% to 5.82%, with the corresponding QFLS increasing from ~ 1.235 to ~ 1.280 eV. Detailed energetic loss analysis reveals that the bulk non-radiative loss decreases drastically from 94.0 to 52.5 meV, whereas the interfacial

recombination loss decreases only marginally from 23.2 to 21.0 meV. These thermodynamic metrics demonstrate that the V_{OC} enhancement is primarily dominated by the mitigation of bulk defects. Consistently, the light-intensity-dependent V_{OC} ideality factor slope decreases from 1.80 to 1.29 k_BT/q, further confirming the effective suppression of trap-assisted recombination pathways (Figure S35b). These results explain the improved V_{OC} and confirm the target strategy establishes a structurally coherent perovskite framework with a minimized trap density."

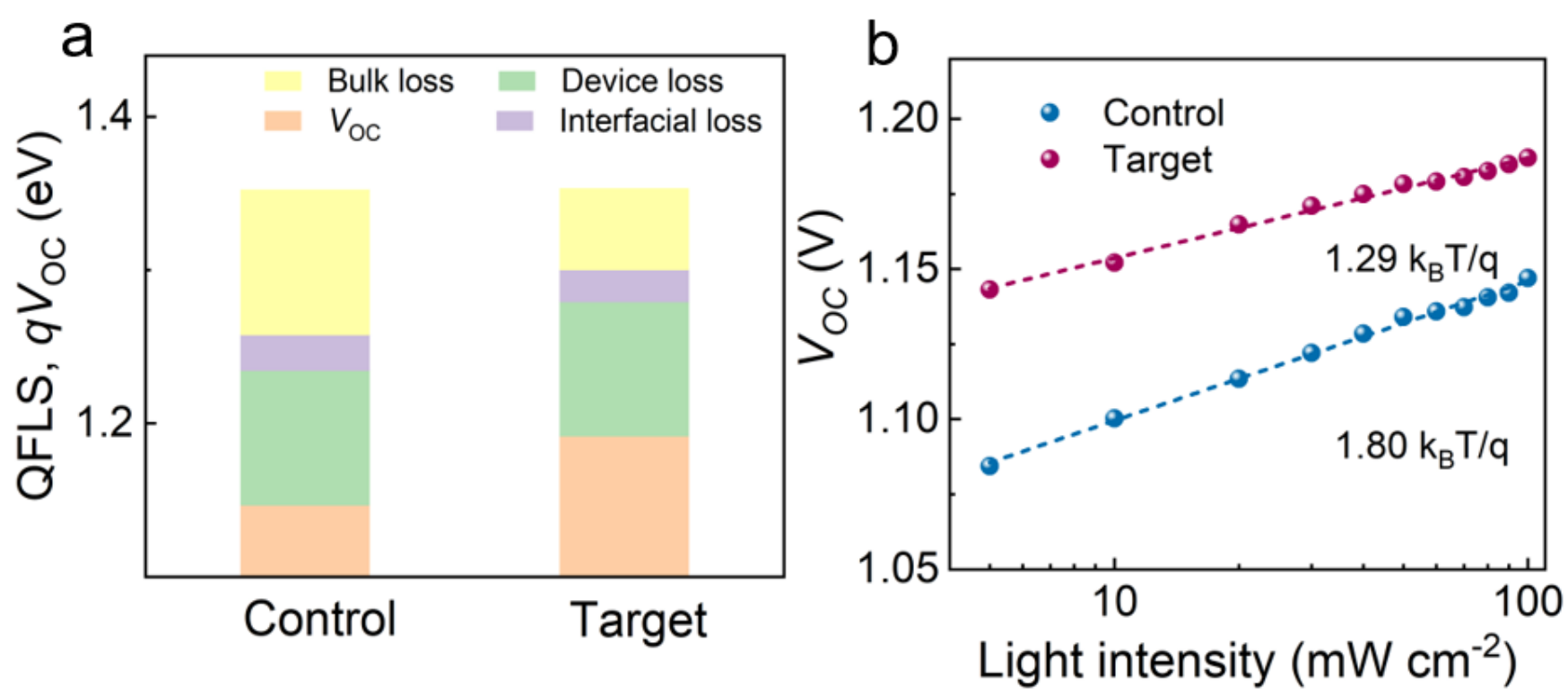

Figure S35. Mechanistic origin of the enhanced open-circuit voltage (V_{OC}) in the target device. a) Photoluminescence quantum yield (PLQY)-derived quasi-Fermi level splitting (ΔE_F or QFLS) values for the neat perovskite films and multi-layer device architectures. b) Light-intensity-dependent V_{OC} characteristics of the control and target devices.

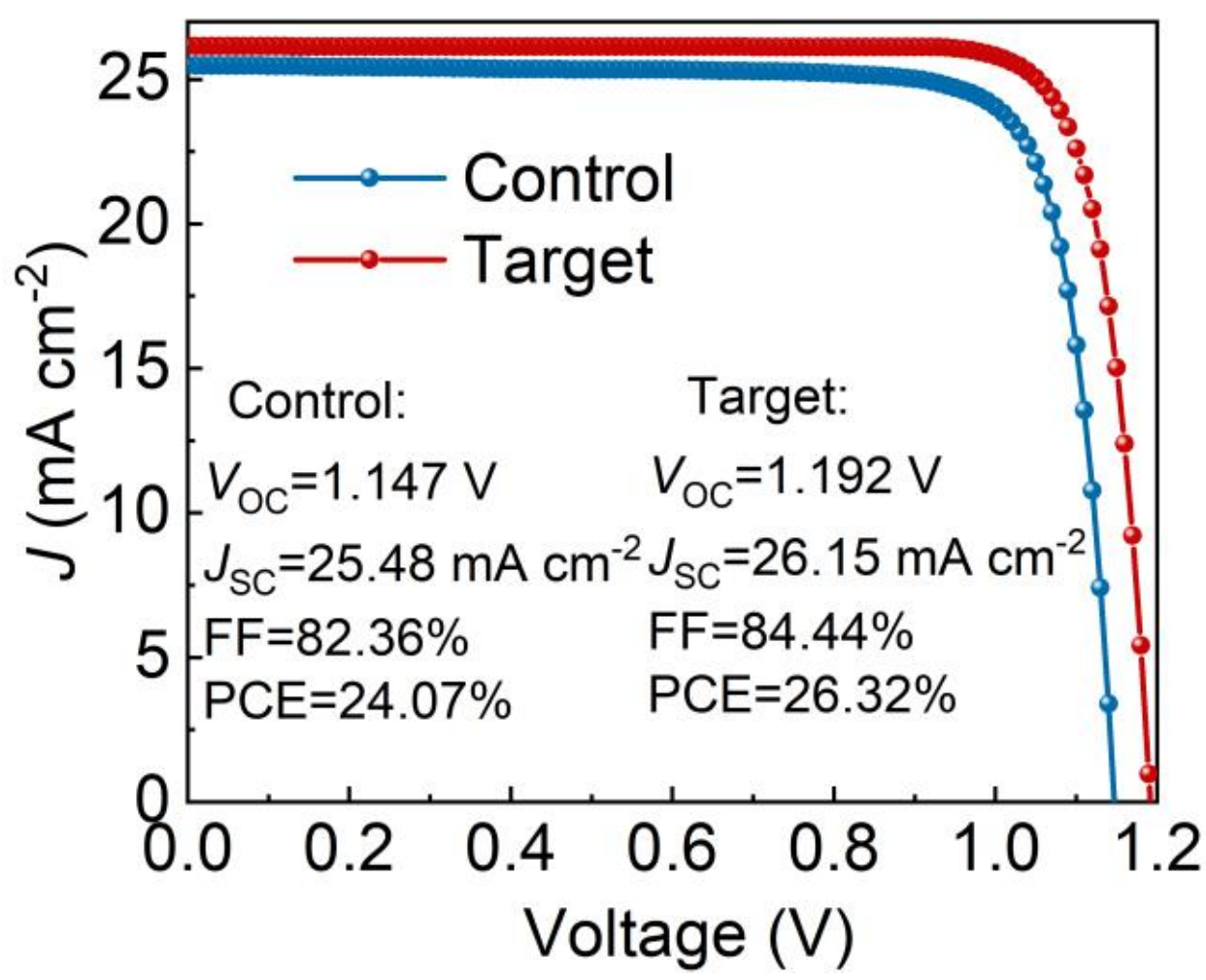

Figure 4. B) J-V curves of the control and the target champion devices (0.06 cm^2).

New references are added in the Supporting information:

16. Zhang S, *et al.* The role of bulk and interface recombination in high-efficiency low-dimensional perovskite solar cells. *Adv. Mater.* **31**, 1901090 (2019).

10. Additionally, there are several non-scientific issues: (i) in many places, the manuscript merely describes experimental phenomena without providing further conclusions or insights; (ii) many schematics depict the traditional 3D perovskite crystal structure as excessively defective.

Authors Response:

We thank the reviewer for this constructive and important comment. We have thoroughly revised the manuscript to place greater emphasis on the core conclusions and physical insights derived from each characterization method. Furthermore, we have modified the corresponding schematic illustrations to ensure a scientifically accurate, balanced comparison between the control and target films, thereby avoiding any exaggerated representation of defect densities within the control 3D perovskite matrix.

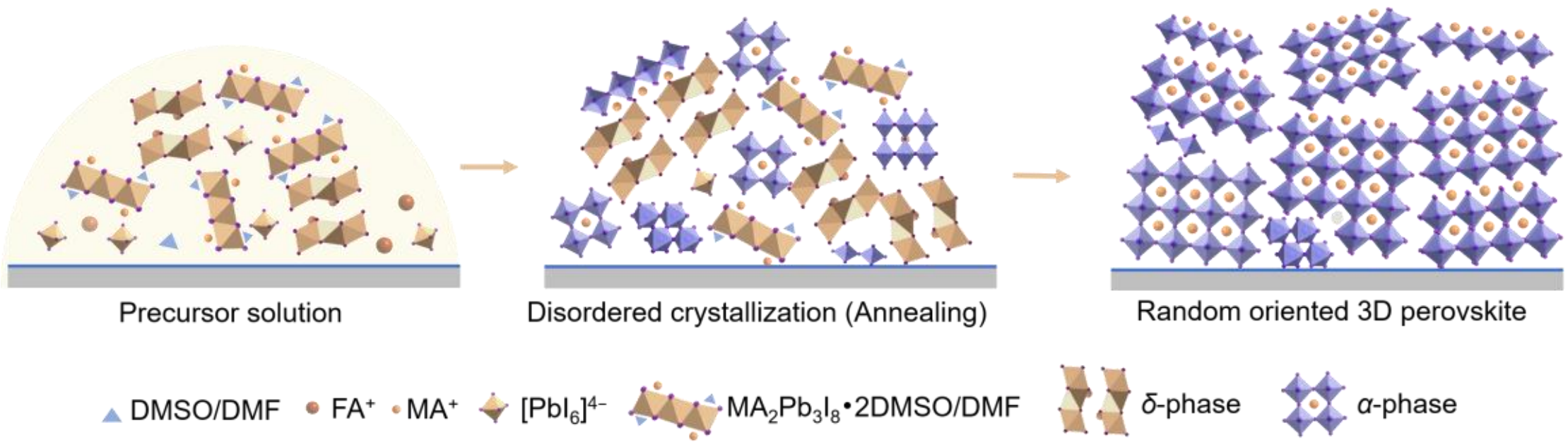

Figure S7. Schematic illustration of the possible phase evolution in the control perovskite film during annealing.

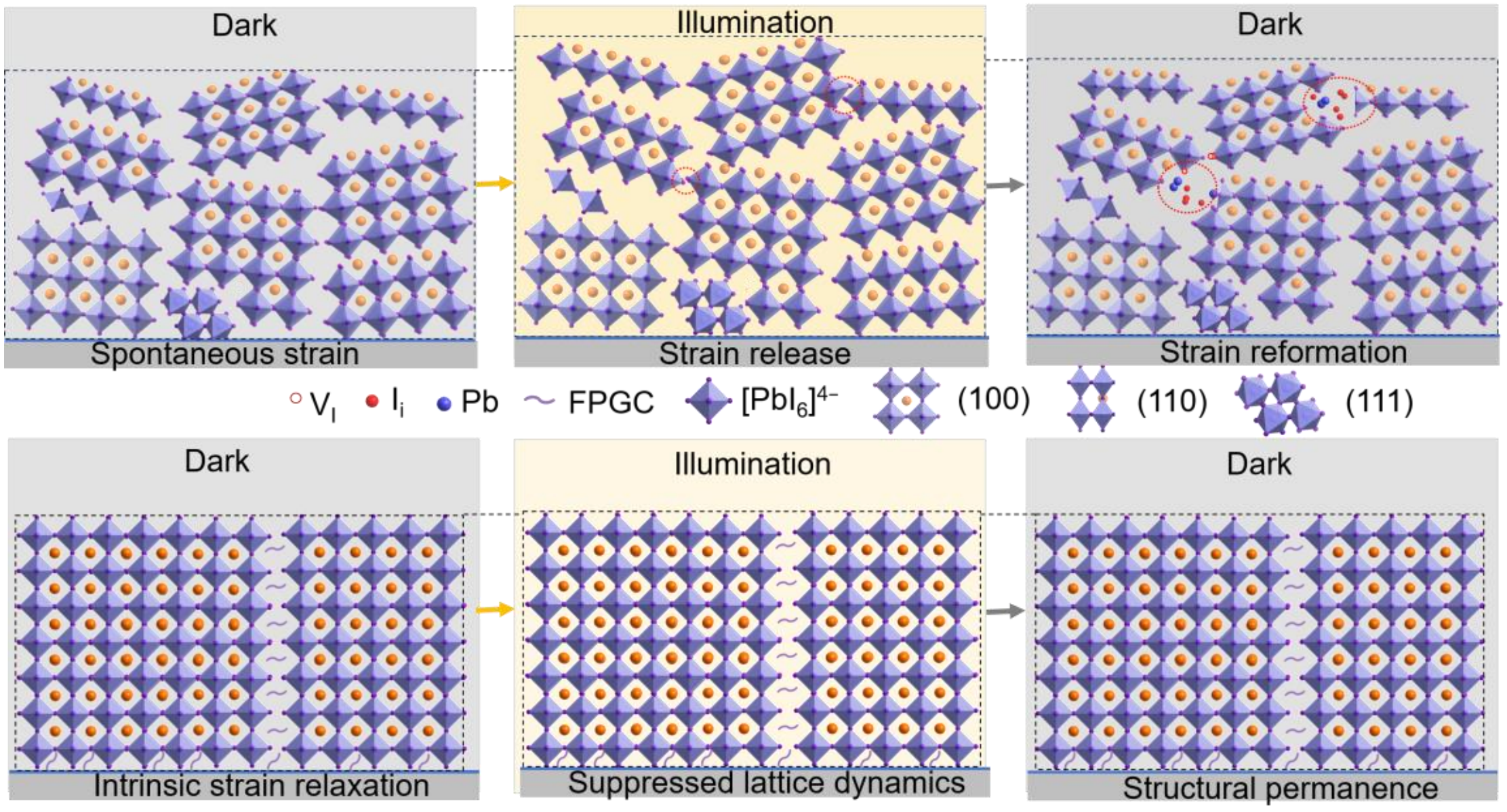

Figure 3. Schematic illustrations showing the structural lattice variation and phase evolution of the D) control and E) target perovskites under cyclic illumination. Dashed red circles highlight defect formation and structural degradation within the perovskite matrix.

Reviewer #2 (Remarks to the Author):

In this work, the authors propose a sacrificial template strategy for the regulation of the lattice orientation of perovskite films, with a view to improving device stability. Specifically, a two-dimensional perovskite 1-(p-fluorophenyl)biguanide lead iodide, with strong (00l) texture and high lattice-matched heterointerface to the three dimensional perovskite, serves as a template. These findings contribute to the fundamental understanding and technical improvement of perovskite solar energy conversion technologies. Therefore, the present manuscript is suitable for publication in Nature Communications after minor revision.

Authors Response:

We thank the reviewer for the insightful comments and recommendation.

C2.1. The authors claim that the uniform crystallization is achieved across the entire space through the sacrificial template strategy. In order to provide substantiating evidence for this claim, further characterization of the bottom interface should be performed. Such further characterization may include XRD or SEM analysis, which would serve to improve clarity.

Author response:

We thank the reviewers for this important comment. As recommended, we have integrated both buried-interface scanning electron microscopy (SEM) and X-ray diffraction (XRD) analyses to evaluate the localized crystallization behavior and structural uniformity at the film/substrate junction:

The buried-interface SEM images reveal that the target film exhibits a significantly more uniform, compact, and highly coalesced grain morphology at the bottom interface, whereas the control film displays disordered crystallization and irregular domain boundaries. Rear-side XRD profiling confirms that the target film successfully maintains the identical, preferred out-of-plane orientation trend observed from the top

surface.

These complementary depth-resolved characterizations have been incorporated in **Fig. S23**, strongly supporting the conclusion that the crystallographic orientation is systematically regulated throughout the entire film thickness.

As suggested, the following sentences were revised on pages 15 in the revised manuscript: "This structural improvement extends to the buried substrate interface, indicating uniform film growth throughout the entire thickness (Figure S23). Consistently, XRD characterization targeted at the buried interface exhibits the same preferential (100) texture observed at the top surface, confirming through-thickness crystallographic uniformity (Figure S24)."

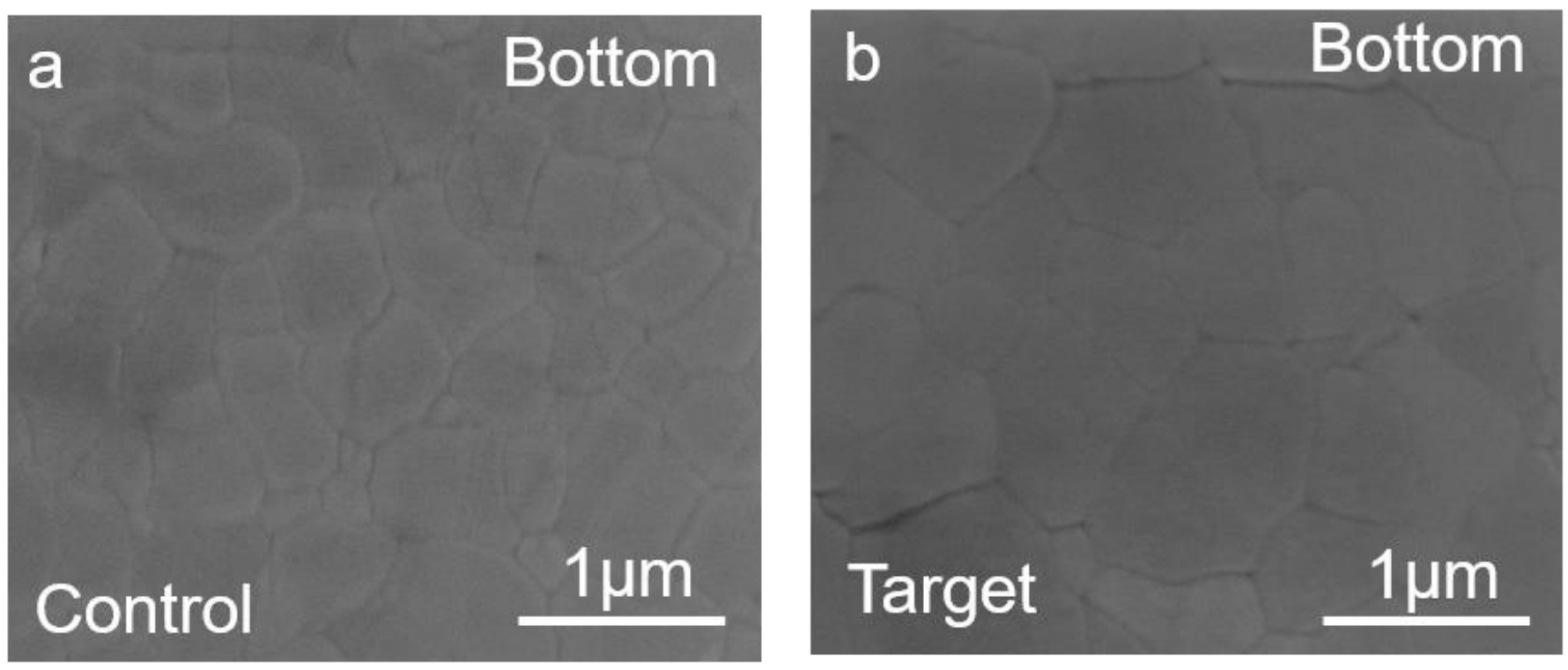

Figure S23. SEM images of bottom surface of the a) control and b) target perovskite film.

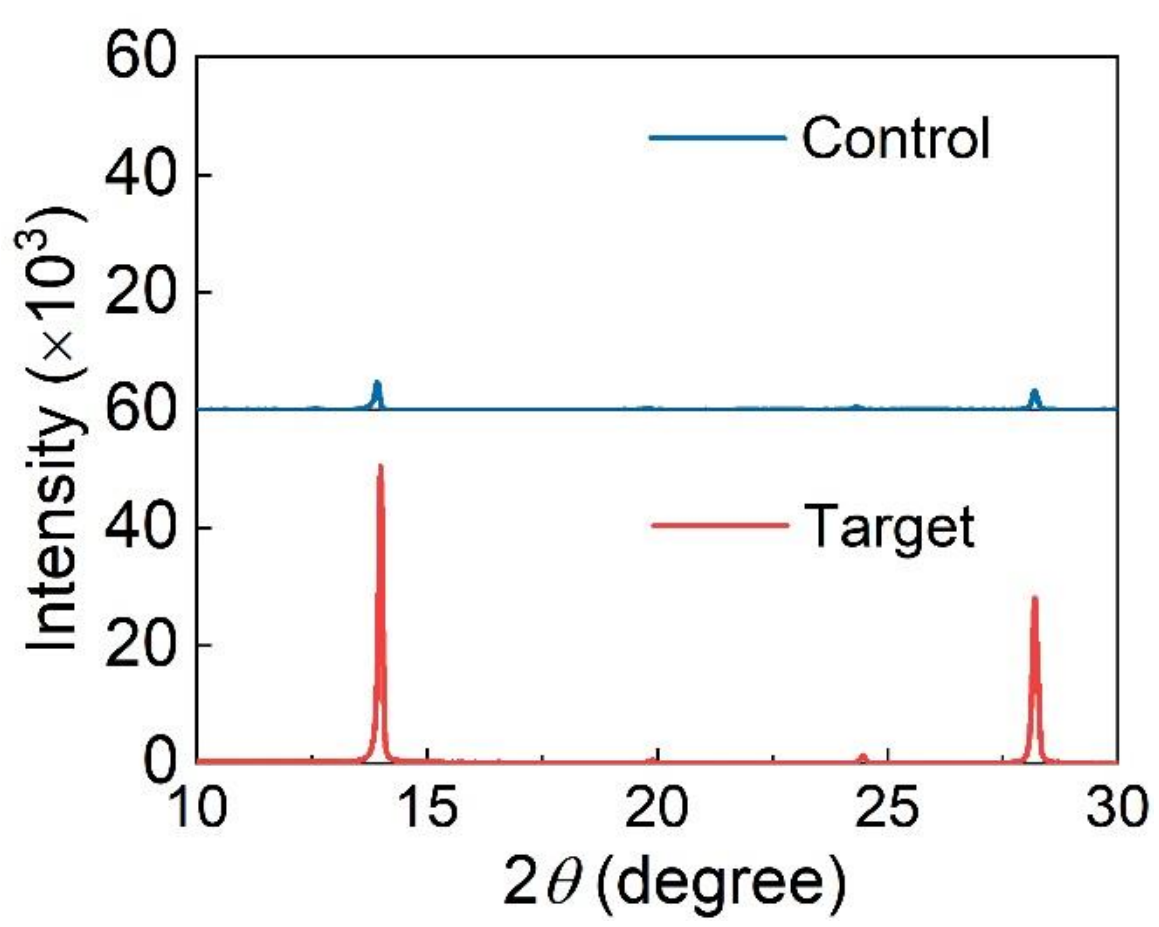

Figure S24. Comparison of the XRD patterns of the control and target films from bottom surface.

C2.2. The interaction between PbI_2 and DMF or other components in the precursor solution, amongst other factors, has been identified as a pivotal parameter in determining the quality of the film. The authors are recommended to provide information for estimating the binding energies in Figure S5. The necessary description should be given in the experimental methods.

Authors Response:

According to the suggestion, we have provided a more detailed description of the methods used to estimate the binding energy.

As suggested, following sentences were added on page 6 in the Supporting Information:

"*Density functional theory calculations.* First-principles computations were carried out using the Vienna Ab initio Simulation Package (VASP) based on density functional theory (DFT). The exchange-correlation interaction was described using the generalized gradient approximation (GGA) parameterized by the Perdew-Burke-Ernzerhof (PBE) functional. Projector-augmented wave (PAW) pseudopotentials were adopted to describe ion–electron interactions with a plane-wave cutoff energy of 500 eV. The electronic energy and ionic forces were converged to within 10^{-5} eV and 0.03 eV Å^{-1}, respectively. A vacuum spacing of 15 Å was applied perpendicular to the slab to avoid unphysical periodic interlayer interactions. For each PbI_2–ligand system (including DMF, DMSO, FA^+, and $FPGC^+$), multiple initial binding configurations featuring distinct coordination sites and molecular orientations were constructed and fully relaxed. The configuration yielding the lowest total energy was selected for binding-energy comparison (displayed in Supplementary Fig. 5).

The binding energy (ΔE_{bind}) was calculated using the following equation:

$$\Delta E_{\mathrm{bind}} = E_{\mathrm{complex}} - E_{\mathrm{PbI_2}} - E_{\mathrm{ligand}}$$

where E_{complex}, $E_{\mathrm{PbI_2}}$ and E_{ligand} represent the total energies of the optimized PbI_2-ligand complex, the isolated PbI_2 slab, and the isolated ligand, respectively. The PbI_2-DMF, PbI_2–DMSO, PbI_3^-–FA^+, and PbI_3^-–$FPGC^+$ systems were modeled as net-neutral configurations. For the charged species (FA^+, $FPGC^+$, PbI_2–FA^+, and PbI_2–$FPGC^+$), the net charge of the simulation cell was set to +1 by reducing the total electron account accordingly.

The three-dimensional charge density difference ($\Delta\rho(r)$) was calculated as:

$$\Delta\rho(r) = \rho_{\mathrm{complex}} - \rho_{\mathrm{PbI_2}} - \rho_{\mathrm{ligand}}$$

where ρ_{complex}, $\rho_{\mathrm{PbI_2}}$ and ρ_{ligand} are electronic charge densities of the optimized complex and its constituent isolated fragments, respectively, with the fragments maintaining the exact spatial coordinates of the fully relaxed complex geometry."

C2.3. In order to substantiate the enhancements in in-plane uniformity (Figure 4E), the proportions or intensity ratios of different crystal planes across the large-area film should be provided.

Authors Response:

We thank the reviewer for this helpful suggestion. In response, we have quantified the in-plane crystallographic uniformity of the large-area films by extracting the X-ray diffraction (XRD) peak-intensity ratio of the (110) and (100) planes across a nine-position grid over the 5 × 5 cm^2 substrate.

For the control film, the (110)/(100) intensity ratio fluctuates widely from 0.0567 to 0.1100, indicating a highly irregular spatial distribution and localized variations in crystallite orientation. In sharp contrast, the target film exhibits significantly suppressed and tightly distributed (110)/(100) intensity ratios, ranging narrowly from 0.0080 to 0.0140.

These quantitative datasets (now incorporated into **Fig. S36**) conclusively demonstrate

a substantially stronger, more spatially uniform out-of-plane (100) texture across the entire macro-scale substrate area, validating the enhanced macroscopic processing homogeneity mediated by the FPGC ligand.

As suggested, the following sentences were revised on page 17, lines 11-15 from the bottom in the revised manuscript: "Consistently, the extracted (110)/(100) XRD intensity ratio across the nine regions decreases sharply from a broad distribution of 0.057–0.110 for the control film to a narrow, minimized range of 0.008–0.014 for the target film (Figure S36), confirming excellent large-area homogeneity."

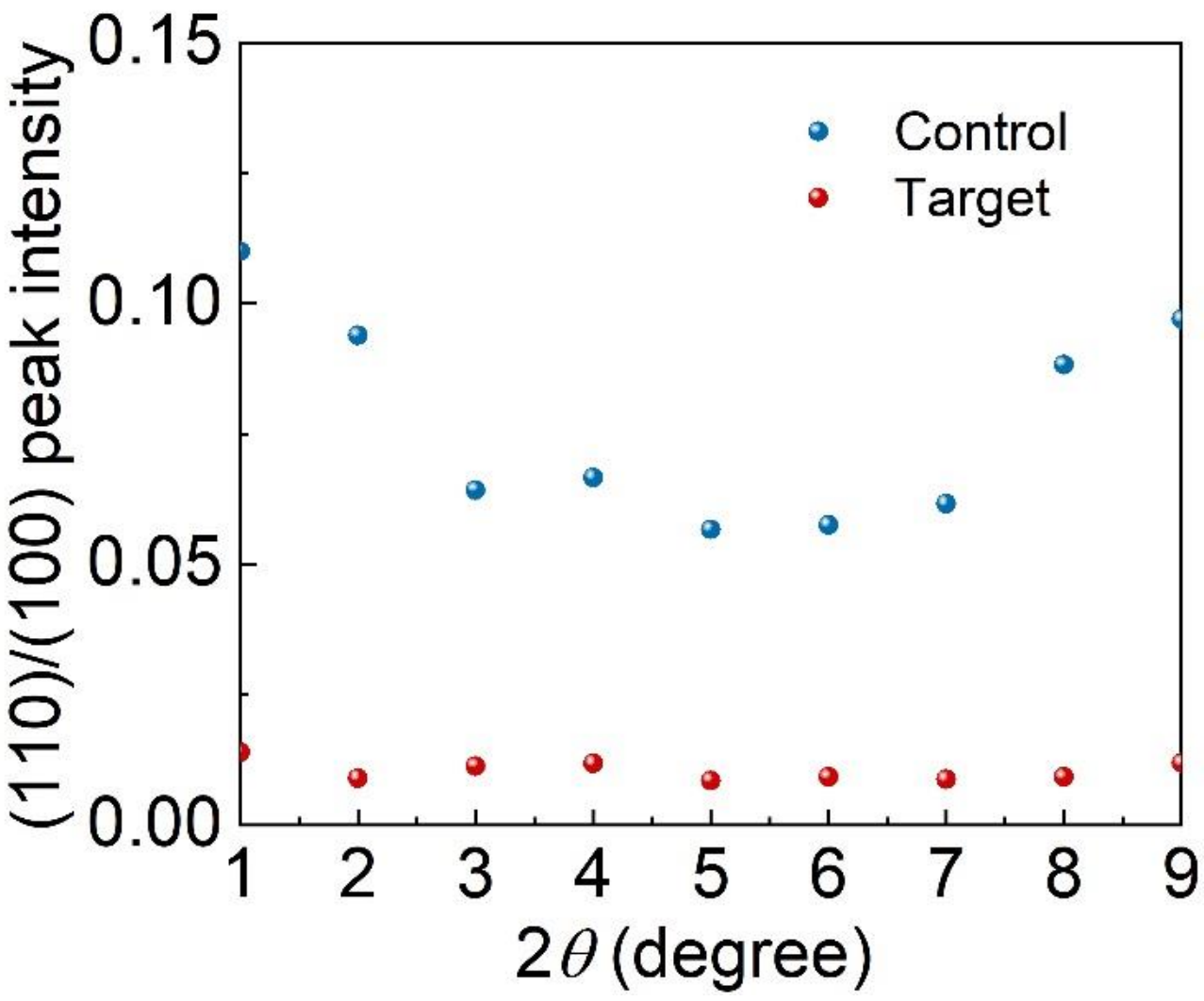

Figure S36. The peak intensity ratio of (100) and (110) facet of nine samples obtained by segmenting a 5 × 5 cm^2 control and target perovskite film.

C2.4. As shown in Figure S25, the stability test entailed the replacement of the hole transport material Spiro-OMeTAD with alternative materials. Consequently, it is imperative to elucidate the initial performance of all devices subjected to stability tests. A commentary on the phenomena observed in the stability test is highly warranted in this study.

Authors Response:

Thanks for this constructive suggestion. According to this suggestion, the initial photovoltaic performance and parameters of the P3HT device are summarized in **Fig. S41**, and these data have been incorporated into the text.

We have added the discussion accordingly in the revised manuscript on Page 18, Lines 20-21: "The corresponding initial PCEs of the control and target devices used for this thermal-cycling evaluation were 22.75% and 24.59%, respectively (Figure S41)."

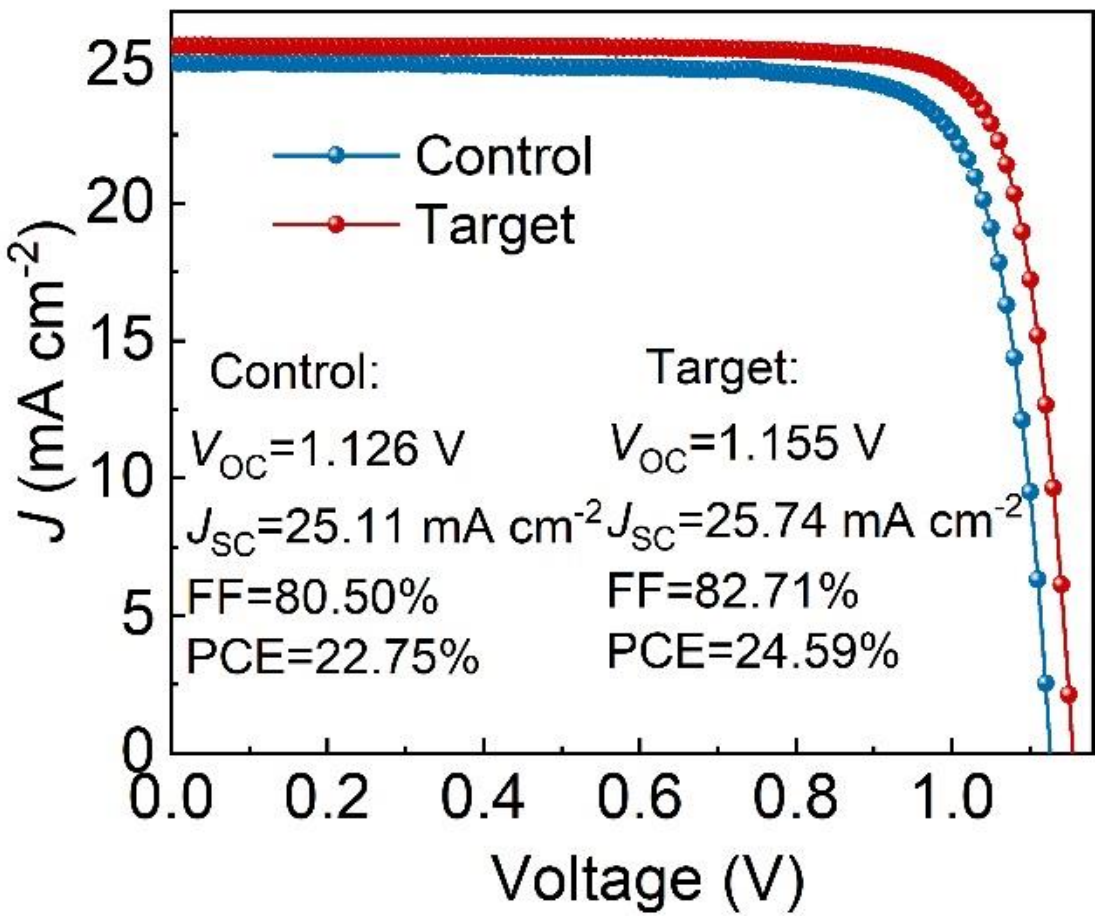

Figure S41. Initial *J-V* curve of the PSCs with P3HT as the hole transport layer.

C2.5. There are some typographical errors and inconsistencies in the use of terminology throughout the manuscript. It is recommended that a thorough proofreading is carried out in order to correct these issues and unify the terminology used across the main text and supplementary information.

Author response:

We thank the reviewers for this important recommendation. The entire manuscript and Supplementary Information have been thoroughly proofread and systematically revised to identify and rectify typographical errors. Furthermore, the scientific terminology has been standardized across all sections to ensure strict structural consistency, clarity, and precision throughout the text.

C2.6. The study concluded that the template under investigation facilitates the vertical orientation growth of the (100) crystal plane of the bulk perovskite layer. The enhanced lattice orientation that is observed throughout the entire space effectively restricts photo-induced lattice expansion and decreases the lattice distortion, thereby contributing to improved operational stability. In order to present this result, it is necessary to undertake preparatory work, including the impact of these crystal planes

and their orientations on perovskite solar cells, such as power conversion efficiency and stability. In addition, it is imperative to ascertain efficacious methodologies for the regulation of these crystal planes.

Author response:

We thank the reviewer for this insightful comment. We agree that the original manuscript lacked sufficient context regarding the fundamental relationships between crystallographic orientation, device efficiency, and long-term stability.

In response, we have thoroughly revised the **Introduction** to establish this essential theoretical background. Specifically, we have clarified that distinct perovskite facets exhibit anisotropic optoelectronic properties, meaning that crystallographic texture directly dictates charge transport and recombination dynamics. Furthermore, we emphasize that grain-to-grain misorientation introduces localized lattice mismatch, structural distortion, and residual strain, which collectively accelerate deep-level trap formation and non-radiative recombination. Within this framework, orientational heterogeneity is directly linked not only to initial photovoltaic performance losses but also to operational structural instability.

We have added the discussion accordingly in the revised manuscript on Page 3, Lines 4-17: "This durability gap originates from unconstrained crystallization kinetics during film formation, which yield polycrystalline matrices characterized by severe crystallographic heterogeneity, deep-level defects, and persistent lattice strain[7-9]. Because distinct perovskite facets exhibit disparate optoelectronic properties, uncontrolled grain orientation impairs charge transport and accelerates non-radiative recombination[10-12]. Specifically, cubic perovskite crystals with a preferred (100) orientation deliver superior carrier mobility and lower trap densities than their (110)- or (111)-oriented counterparts[12-14]. Conversely, grain-to-grain misorientation introduces localized lattice mismatch and residual tensile stress, triggering structural dislocations[15]. These localized strain fields perturb the electronic band structure by distorting Pb-X bond lengths, altering Pb-X-Pb bond angles, and diminishing orbital overlap[16].

Consequently, this pervasive strain heterogeneity creates severe transport bottlenecks and drives chronic long-term device degradation[4,17]."

New references are added in the revised manuscript:

7. Cheng Z, *et al.* Oriented nucleation and growth of halide perovskites. *Chem. Rev.* **126**, 5083-5177 (2026).
8. Luo C, *et al.* Engineering the buried interface in perovskite solar cells *via* lattice-matched electron transport layer. *Nat. Photonics* **17**, 856-864 (2023).
9. Shi P, *et al.* Oriented nucleation in formamidinium perovskite for photovoltaics. *Nature* **620**, 323-327 (2023).
10. Liang Z, *et al.* Molecular sublimation enables 2D-3D transformation of orientational $FAPbI_3$ perovskites. *Nat. Synthesis* **4**, 347-358 (2025).
11. Zhang H, *et al.* Facet engineering in perovskite solar cells: Facet properties, crystallization regulation, and device performance. *Sustainable Chemistry for Energy Materials* **3**, 100045 (2026).
12. Ma C, *et al.* Photovoltaically top-performing perovskite crystal facets. *Joule* **6**, 2626-2643 (2022).
13. Ma C, *et al.* Unveiling facet-dependent degradation and facet engineering for stable perovskite solar cells. *Science* **379**, 173-178 (2023).
14. Ma C, Grätzel M, Park N-G. Facet engineering for stable, efficient perovskite solar cells. *ACS Energy Lett.* **7**, 3120-3128 (2022).
15. Xue D-J, *et al.* Regulating strain in perovskite thin films through charge-transport layers. *Nat. Commun.* **11**, 1514 (2020).
16. Chen Y, *et al.* Strain engineering and epitaxial stabilization of halide perovskites. *Nature* **577**, 209-215 (2020).
17. Jariwala S, *et al.* Local crystal misorientation Influences non-radiative recombination in halide perovskites. *Joule* **3**, 3048-3060 (2019).

Reviewer #3 (Remarks to the Author):

In this article, Ma et al. demonstrate a highly oriented two-dimensional (2D) perovskite that acts as a sacrificial intermediate layer for the templated growth of three-dimensional (3D). The photovoltaic devices fabricated with this technique reveal power conversion efficiencies of 26.15 % for small area and 22.25 % for large area. Additionally, the device exhibits excellent day/night cycling stability under realistic conditions (ISOS-LC-1 protocol, ~50 °C in light and ~25 °C in dark). Although the work has some merit, I have major concerns that need to be addressed before this manuscript can be published in Nature Communications.

Authors Response:

We thank the reviewer for the insightful comments and recommendation.

C3.1. The authors claim that the 2D $FPGC_2PbI_4$ perovskite template directs 3D crystallization via lattice-matched, quasi-epitaxial growth (page 5, line 109). For epitaxial growth and lattice templating, the in-plane lattice parameter of 2D perovskite should be matched with the 3D perovskite. The out-of-plane (002) lattice spacing does not contribute to epitaxial templating. Therefore, the claim of lattice templating is not justified.

Authors Response:

We thank the reviewer for this important and technically precise comment. We completely agree that the terms "epitaxial growth" and "lattice templating" imply strict structural conditions that were not rigorously demonstrated.

Accordingly, we have thoroughly revised the manuscript to remove or substantially soften these expressions. Instead, we now describe the 2D $FPGC_2PbI_4$ phase more accurately as a transient, long-range-ordered crystalline intermediate that guides the oriented crystallization of the 3D perovskite during the initial stages of thermal annealing. Its mechanical role is now interpreted as an intermediate-mediated

orientational guidance effect driven by compatible out-of-plane lattice spacings, rather than a rigid epitaxial matching relationship. These corrections have been applied systematically throughout the text.

C3.2. Is there any existence of 2D crystal seeds found in the precursor solution? DLS or similar investigations could be performed to confirm that.

Authors Response:

We thank the reviewer for this valuable suggestion. Following the reviewer's advice, we performed dynamic light scattering (DLS) measurements on the precursor solutions to investigate the existence and evolution of colloidal aggregate species prior to thin-film deposition:

The DLS profiles reveal that the introduction of the FPGC ligand induces the formation of an additional population of large colloidal aggregates with hydrodynamic diameters exceeding 1 μm, a feature completely absent in the control precursor matrix. Concurrently, the size distribution of the primary, smaller colloidal species narrows substantially, concentrating within a well-defined 30–90 nm range.

These findings (now incorporated into **Fig. S5**) demonstrate that FPGC significantly restructures the precursor solution framework. The emergence of the micrometer-scale colloid population provides direct evidence of FPGC-induced seed-like aggregates, which act as nuclei to govern the subsequent crystallization pathways.

Accordingly, we have added the discussion accordingly in the revised manuscript on Page 6-7: "Dynamic light scattering (DLS) profiles reveal that the FPGC-containing precursor solution develops an additional population of large colloids (>1 μm) alongside a narrower, more concentrated distribution of smaller species in the 30-90 nm range (Figure S5). This behavior points to a profound reconstruction of the precursor colloidal structure, which is consistent with the formation of seed-like aggregates driven by a strong, preferential association between the FPGC cation and PbI_2[46, 47]."

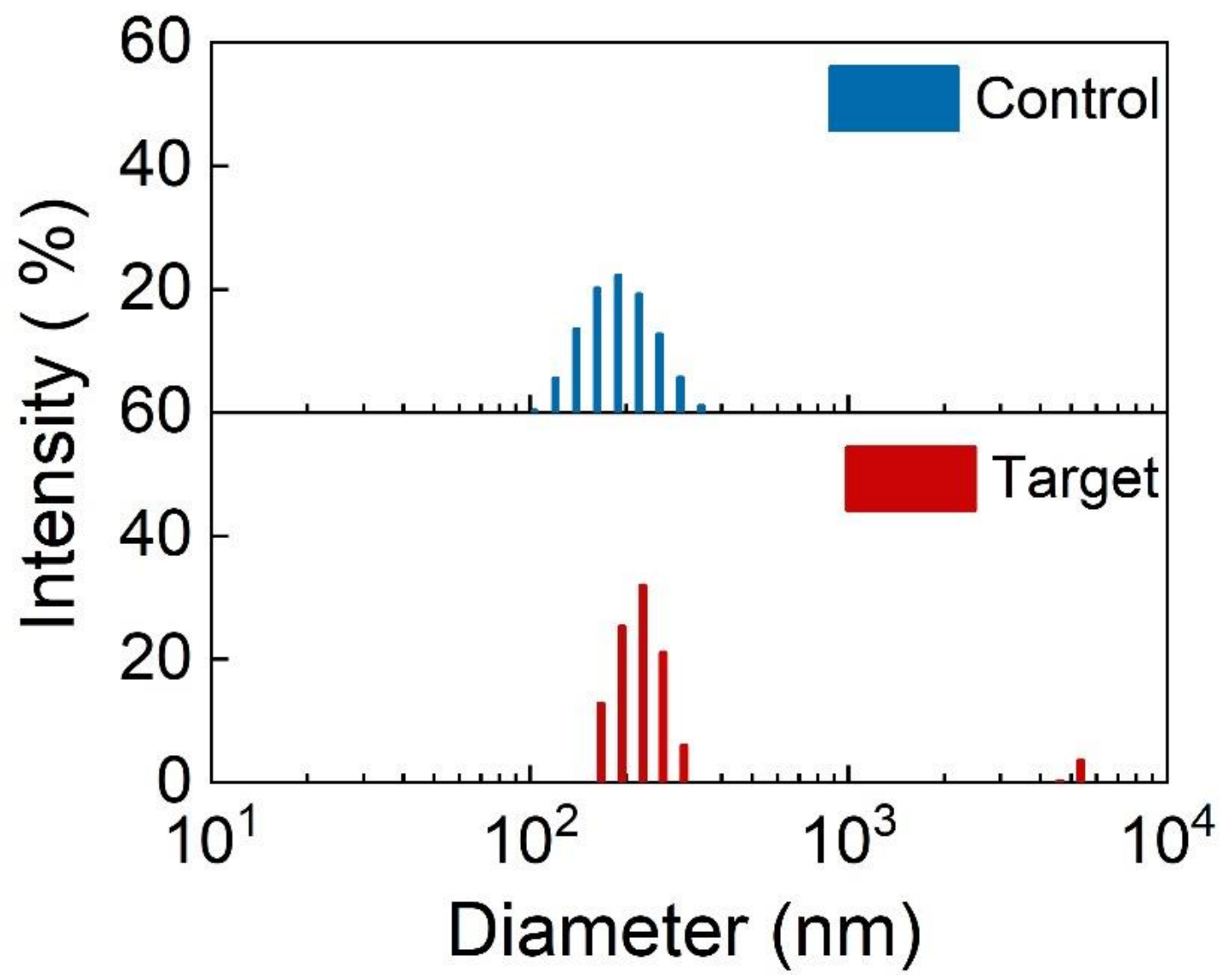

Figure S5. Dynamic light scattering spectra of control and target precursor solutions.

C3.3. For the in-situ formation of 2D, FPGC molecule has been utilized in the perovskite precursor solution. The role of "Cl" is not explained at all in the manuscript. The observed orientation effect probably originates form enhanced crystallization facilitated by the 2D component and Cl incorporation. The role of 2D and Cl should be investigated systematically.

Authors Response:

We thank the reviewer for this critical comment. We agree that distinguishing a generic chloride-assisted crystallization effect from the specific structural role of the FPGC molecule is essential for establishing the precise crystallization mechanism.

While chloride-containing additives are well known to influence phase evolution, their impact is highly sensitive to their chemical source and spatial distribution (DOI: 10.1021/acs.nanolett.5b03271, 10.1016/j.joule.2019.06.014). Indeed, poorly controlled chloride addition can lead to adverse effects, such as additive aggregation, compositional heterogeneity, and strain accumulation. To isolate the specific function of the FPGC ligand from these baseline chloride effects, we performed three distinct control experiments: 1) Yielded poor crystallinity for the α-phase perovskite with

significant residual δ-phase impurities, confirming that MACl remains necessary to lower the phase-transition barrier; 2) Fabricated by increasing the MACl molarity to match the extra chloride content introduced by FPGC. This control preserved the baseline chloride chemistry but completely failed to replicate the pronounced out-of-plane (100) texture observed in the target films; 3) Evaluated using 1-(3-fluorophenyl)biguanide hydrochloride (3-FPGC), which preserves the identical chloride-containing biguanide framework but alters the fluorine substituent position. While 3-FPGC slightly enhanced peak intensities, grazing-incidence wide-angle X-ray scattering (GIWAXS) confirmed it could not induce the highly oriented vertical framework, nor did it form a crystalline intermediate analogous to $FPGC_2PbI_4$.

Collectively, these quantitative evaluations (now summarized in **Supplementary Note 1** and **Figs. 18 and 19**) demonstrate that the observed orientation-control mechanism cannot be achieved by chloride species alone. Instead, it is uniquely dictated by the specific molecular structure of FPGC and its capacity to assemble a transient crystalline intermediate that template-guides preferentially oriented 3D grain growth.

Accordingly, we have added the discussion accordingly in the revised manuscript on Page 12, Lines 4-9: "Furthermore, control experiments using methylammonium chloride (MACl)-free, MACl-matched, and 1-(3-fluorophenyl)biguanide hydrochloride (3-FPGC)-containing precursors demonstrate that the orientation effect cannot be ascribed to chloride addition alone; instead, it is intrinsically linked to the specific molecular structure of FPGC and its unique capability to form a transient ordered intermediate (Figures S18, S19 and Note1)."

We have added the discussion accordingly in the Supporting Information on Pages 60-62: "**Supplementary Note1: Synergistic roles of MACl and the $FPGC_2PbI_4$ transient intermediate in crystallization and orientation control**

Recent studies have demonstrated that chloride-based additives are highly effective in regulating crystallization kinetics and enhancing perovskite thin-film quality [17].

Among these, methylammonium chloride (MACl) is widely utilized to lower the formation energy of the target perovskite phase, stabilize the photoactive black polymorph, and promote preferential crystallographic growth [18]. However, MACl incorporation alone is often insufficient to suppress all unfavorable intermediate pathways; furthermore, inhomogeneous chloride distribution or additive aggregation can induce severe compositional heterogeneity, defect formation, and vertical strain accumulation. To address these limitations, multi-component additive engineering strategies have been explored [19]. For instance, Ding et al. demonstrated that combining MACl with a Lewis-basic ionic liquid effectively suppresses MACl aggregation, thereby enhancing phase homogeneity, boosting crystallinity, and reducing defect density [20]. Similarly, Xiong et al. reported that the incorporation of oxalate-based alkali metal compounds can successfully homogenize the chlorine distribution within MACl-containing systems [21].

In this work, we introduced a two-dimensional (2D) perovskite intermediate into a methylammonium chloride (MACl)-containing precursor system. The resulting improvement in full-space crystallization homogeneity and the preferential out-of-plane growth along the (100) crystallographic planes arose from the synergistic effects of baseline MACl doping and FPGC incorporation. To elucidate whether these modifications originated simply from chloride-assisted crystallization or instead from the specific molecular role of FPGC, three distinct control experiments were conducted.

First, a baseline control was prepared using the standard precursor formulation without MACl or FPGC, while maintaining all other conditions identical to those established for the control films in the main text. X-ray diffraction (XRD) analysis revealed poor crystallinity for the α-phase along with residual δ-phase impurities (Supplementary Fig. 18a–c). This outcome confirms the essential role of MACl in promoting the δ-to-α phase transition, which aligns with its established impact on precursor coordination and phase-transition kinetics [17].

Second, to test whether the function of FPGC could be replicated simply by increasing the total chloride concentration, an additional-chloride control was fabricated. The MACl content was increased such that the extra chloride molarity was comparable to that introduced by the FPGC ligand (Supplementary Fig. 18d–f). Although this control preserved the chloride-assisted chemistry inherent to the original precursor formulation, it failed to reproduce the pronounced preferential out-of-plane (100) orientation observed in the FPGC-containing films, nor did it induce discernible structural variations in the final thin film. This finding indicates that a minor increase in chloride content alone is insufficient to account for the orientation-control effect.

Third, a structural analogue, 1-(3-fluorophenyl)biguanide hydrochloride (3-FPGC), was examined. This molecule preserves the chloride-containing biguanide framework of FPGC but differs strictly in the positional isomerism of the fluorine substituent on the phenyl ring. When incorporated at an identical concentration to FPGC, 3-FPGC enhanced the intensity of the (100) diffraction peak, indicating some degree of influence on bulk crystallization (Supplementary Fig. 19a–c). However, grazing-incidence wide-angle X-ray scattering (GIWAXS) profiling did not show the pronounced out-of-plane (100) diffraction features characteristic of the FPGC-modified films, demonstrating that enhanced crystallinity alone is inadequate to generate the highly oriented vertical framework.

Furthermore, when 3-FPGC was mixed with PbI_2 and spin-coated under the identical processing conditions used for the FPGC reference system (1 M, 60 °C, 10 min), no distinct diffraction peaks were detected (Supplementary Fig. 19d). This lack of signal indicates that 3-FPGC does not form a crystalline intermediate phase analogous to $FPGC_2PbI_4$. In sharp contrast, the FPGC-containing system assembled a detectable, long-range-ordered crystalline intermediate that closely correlates with the subsequent development of the strongly oriented three-dimensional (3D) perovskite matrix. Taken together, these control evaluations demonstrate that the observed orientation-control mechanism does not arise simply from baseline MACl or supplemental chloride, but is closely coupled with the specific molecular structure of FPGC and its unique capacity

to form a transient crystalline intermediate that templated preferentially oriented 3D crystal growth."

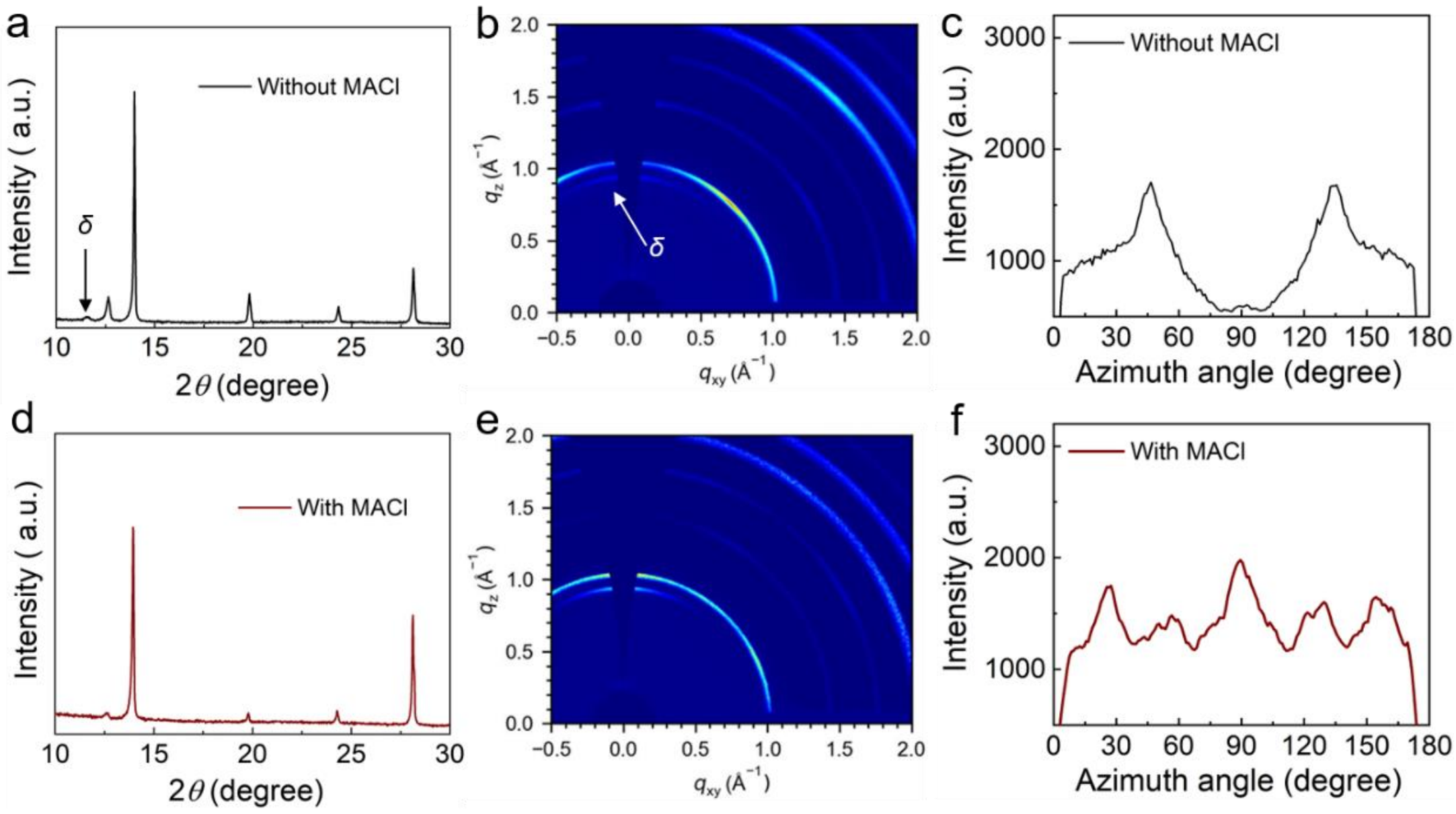

Figure S18. a) XRD patterns of perovskite films prepared without MACl. b) GIWAXS patterns of perovskite films without MACl. c) Azimuthally integrated intensity profiles along the ring at q_{xy} = 10 nm^{-1} of perovskite films prepared without MACl. d) XRD patterns of perovskite films prepared with MACl added at an amount equivalent to that introduced by FPGC. e) GIWAXS patterns of perovskite films prepared with MACl added at an amount equivalent to that introduced by FPGC. f) Integrated intensity plots azimuthally along the ring at q_{xy} = 10 nm^{-1} of perovskite films prepared with MACl added at an amount equivalent to that introduced by FPGC.

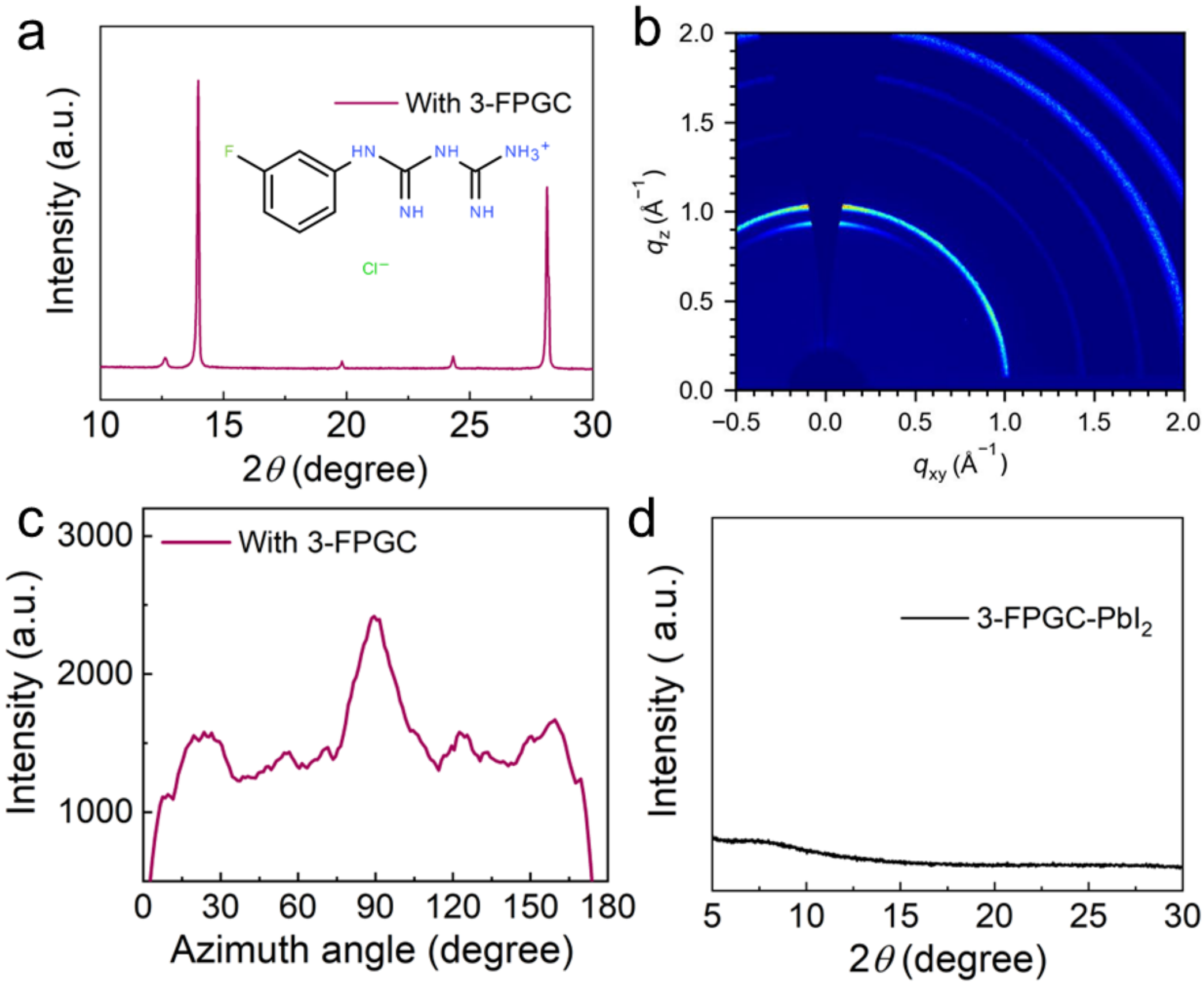

Figure S19. Structural-analogue control using 3-FPGC. a) XRD patterns of perovskite films prepared with the 3-FPGC. The inset shows the molecular structure of 3-FPGC. b) GIWAXS patterns of perovskite films prepared with the 3-FPGC. c) Integrated intensity plots azimuthally along the ring at q_{xy} = 10 nm^{-1}. d) XRD pattern of the film prepared by spin-coating the 3-FPGC with PbI_2.

New references are added in in the Supporting Information:

17. Liu X, Guo Y, Cheng Y, Lu S, Li R, Chen J. Advances in chloride additives for high-efficiency perovskite solar cells: multiple points of view. *Chem. Commun.* **59**, 13394-13405 (2023).

18. Kim M, *et al.* Methylammonium chloride induces intermediate phase stabilization for efficient perovskite solar cells. *Joule* **3**, 2179-2192 (2019).

19. Wang J, *et al.* Homogenized optoelectronic properties in perovskites: Achieving high-efficiency solar cells with common chloride additives. *J. Am. Chem. Soc.* **148**, 6229-6237 (2026).

20. Ding B, *et al.* Dopant-additive synergism enhances perovskite solar modules.

Nature **628**, 299-305 (2024).

21. Xiong Z, *et al.* Homogenized chlorine distribution for >27% power conversion efficiency in perovskite solar cells. *Science* **390**, 638-642 (2025).

C3.4. TOF-SIMS and XPS analyses demonstrate that the FPGC molecule is incorporated in the final film, while the XRD analysis demonstrates the 2D decomposes above 80 ˚C-Why? Additionally, if the molecule is readily extinguished at high temperature, that could lead to degradation of perovskite in harsh conditions.

Authors Response:

We thank the reviewer for this critical comment. We wish to clarify that the disappearance of the low-angle two-dimensional (2D) X-ray diffraction (XRD) reflections above ~80 °C does not signify that the FPGC molecule decomposes or volatilizes from the film.

To definitively verify the thermal stability of the molecule, we have performed thermogravimetric analysis (TGA) of the neat FPGC ligand, which demonstrates a high decomposition threshold of 217 °C (**Fig. S3**). Because this chemical degradation temperature is substantially higher than the processing window, the vanishing diffraction signal is instead attributed to a loss of long-range crystalline order and the structural conversion of the transient $FPGC_2PbI_4$ intermediate phase during annealing.

While the intermediate phase structurally collapses as a distinct crystalline domain, the FPGC molecules remain completely within the thin film. This phase behavior is independently confirmed by our time-of-flight secondary-ion mass spectrometry (ToF-SIMS) and X-ray photoelectron spectroscopy (XPS) results, which detect the persistent chemical signature of the FPGC species throughout the final annealed film. Therefore, the thermal disappearance of the 2D diffraction feature at ~80 °C reflects a controlled, intermediate-mediated phase conversion during three-dimensional (3D) perovskite growth rather than the thermal instability of the ligand itself, posing no intrinsic

degradation risk under device-relevant operating conditions.

As suggested, the following sentences were revised on pages 5-6in the revised manuscript: "Thermogravimetric analysis (TGA) confirms that the isolated FPGC molecule remains stable up to 217 °C (Figure S3); thus, the dissolution of the 2D XRD features above 80 °C does not stem from molecular decomposition, but rather from a loss of long-range crystalline order within the $FPGC_2PbI_4$ framework."

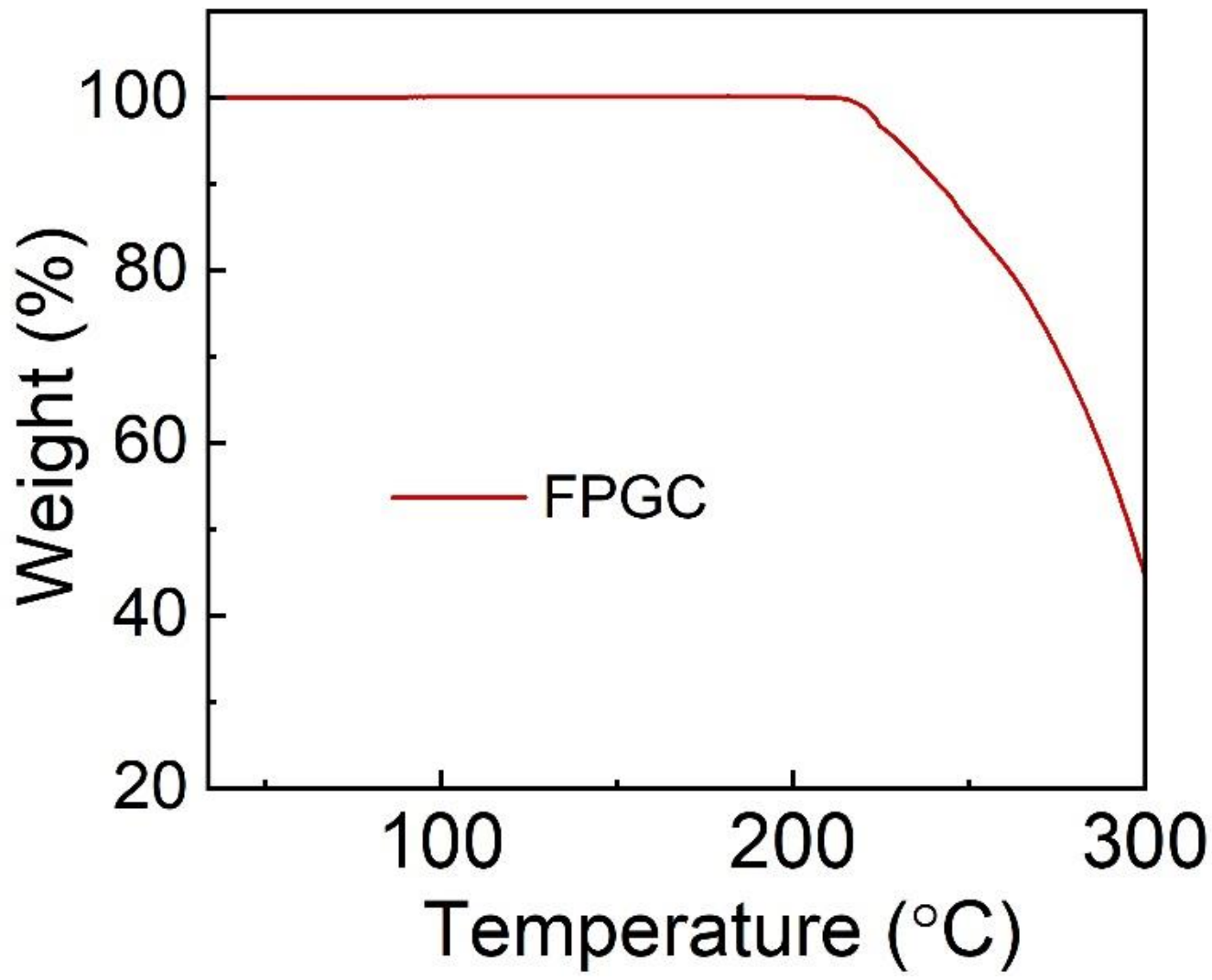

Figure S1.. Thermogravimetric analysis profile of the neat FPGC molecular powder.

C3.5. Why do GIXRD or XRD analyses reveal no low-angle peak for 2D perovskite in the final film?

Authors Response:

We thank the reviewer for this important question. The absence of low-angle diffraction peaks in the final film confirms that the $FPGC_2PbI_4$ phase is not retained as a distinct, long-range-ordered two-dimensional (2D) crystalline domain after annealing.

Instead, we interpret this phase as a transient, ordered crystalline intermediate that forms during the initial stages of wet-film crystallization and subsequently undergoes structural phase conversion into the three-dimensional (3D) perovskite matrix. This behavioral pathway aligns with recent high-impact literature demonstrating that 2D

perovskite components formed during solution processing can act strictly as transient intermediates, structurally collapsing and disappearing in the final film after successfully template-guiding oriented 3D crystallization (DOI: 10.1038/s41563-024-02073-x). We have incorporated this explicit clarification into the revised manuscript to prevent any reader misunderstanding.

As suggested, the following sentences were revised on page 10, lines 7-10 in the revised manuscript: "Crucially, the complete absence of low-angle diffraction features in the fully annealed target film confirms that the $FPGC_2PbI_4$ phase functions strictly as a transient intermediate and is not permanently retained as a distinct 2D crystalline phase."

C3.6. The cross-section FESEM in Figure 2D is not clear. Please provide high resolution FESEM image.

Authors Response:

We are very grateful to the reviewer for this helpful comment.

As suggested, Figure 2D in the revised manuscript has been updated accordingly.

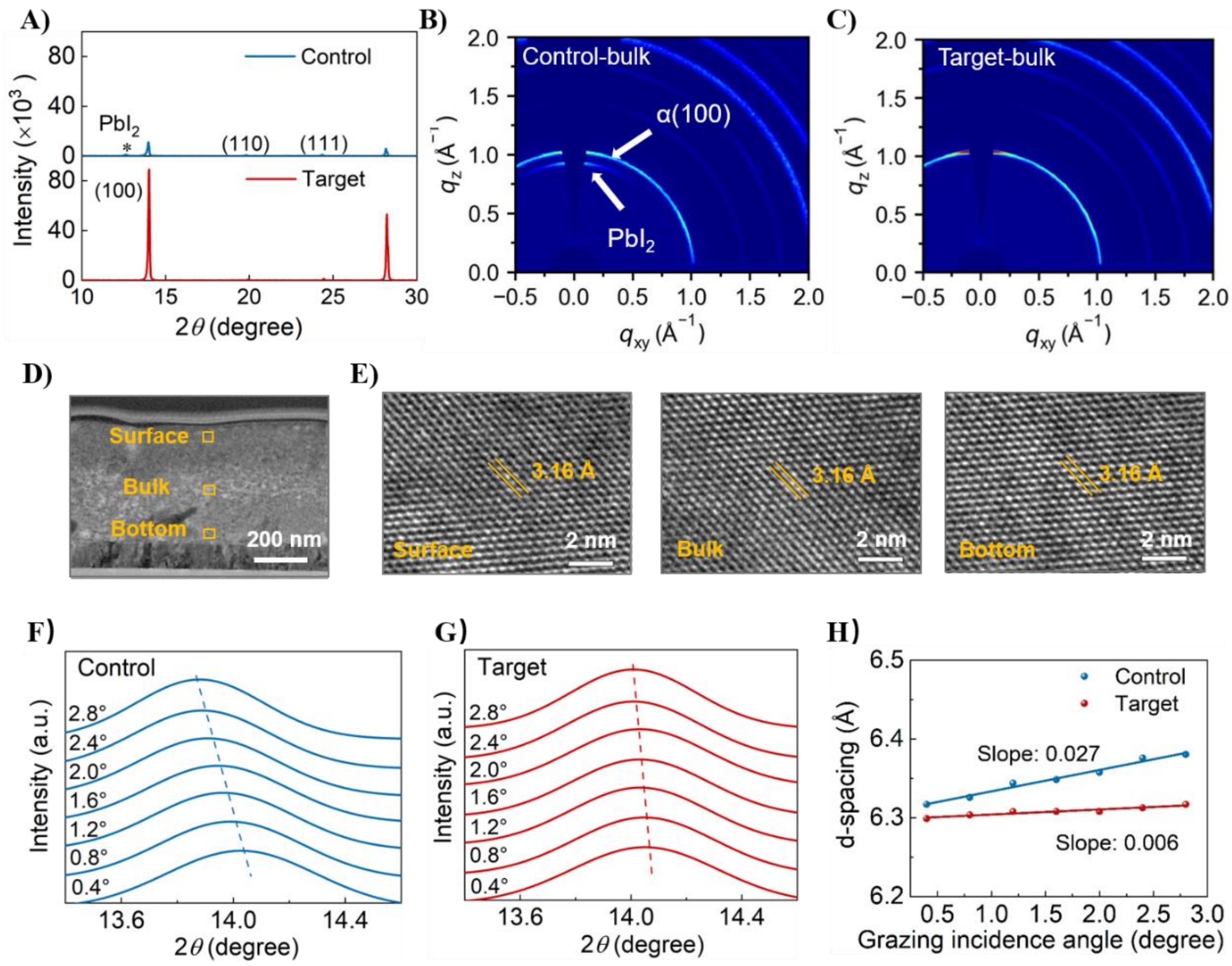

Figure 2. Crystallographic orientation and strain regulation. A) Comparison of the XRD patterns of the control and target films. Two-dimensional grazing-incidence wide-angle X-ray scattering (2D GIWAXS) patterns of the B) control and C) target films recorded at an incidence angle of 1.0°. D) Cross-sectional scanning electron microscopy (SEM) image of the target perovskite film. E) High-resolution lattice fringe images collected from the surface, bulk, and bottom regions indicated in D). Grazing-incidence X-ray diffraction (GIXRD) patterns of the F) control and G) target films at various incident angles. H) Interplanar spacing of the (100) planes derived from GIXRD measurements plotted as a function of the incidence angle.

C3.7. The authors have observed a performance recovery for both control and target devices after the dark phase of the day/night cycle. What is the reason for this observation, and why is it more pronounced for the control sample. Also, it will be interesting to co-relate the acceleration factor for the day/night cycling stability with film analyses.

Authors Response:

We thank the reviewer for this insightful comment. We have clarified the mechanistic origin of the diurnal recovery behavior and incorporated a quantitative accelerated aging framework to validate our stability findings:

1) The performance recovery observed following the dark phase is consistent with the well-established reversible diurnal behavior of perovskite solar cells (PSCs). Under dark resting conditions, the removal of photoexcitation and thermal bias allows for the relaxation of light-induced metastable states—most notably mobile ion redistribution and metastable defect annihilation (DOI: 10.1038/s41560-019-0529-5; 10.1038/ncomms11574; 10.1039/c7ee02956j; 10.1039/c6ee03352k).

In this work, both device configurations exhibit recovery due to the intrinsic, partially reversible nature of the perovskite lattice under cyclic illumination. The more pronounced recovery spike seen in the control device stems from its significantly larger transient structural perturbation during the light phase, which correlates with its severe photoinduced lattice expansion and vertical strain accumulation. This intense structural distortion generates a high density of metastable defects and severe ionic polarization, which partially relax in the dark to yield a dramatic apparent recovery. Conversely, the target device undergoes minimal structural perturbation, resulting in highly suppressed diurnal fluctuations and a more stable, less volatile operational profile.

2) As recommended, we have implemented an accelerated aging analysis under continuous diurnal cycling across a temperature series of 25, 45, 65, 80, and 95 °C. Because negligible degradation occurred at 25 °C within the testing window, the linear degradation rates extracted from the 45–95 °C regimes were fitted using an Arrhenius model. This linear regression yielded an apparent degradation activation energy (E_a) of 0.224 eV. Utilizing a standard reference operating temperature (T_{ref}) of 55 °C (328.15 K) and an accelerated aging temperature (T_{acc}) of 95 °C (368.15 K), the thermal acceleration factor (AF) was evaluated to be 2.37. Based on this kinetic modeling, the

projected T_{80} operational lifetime at 55 °C was estimated to be 78.2 cycles.

These rigorous stability datasets and their mathematical derivations have been fully integrated into the revised manuscript and Supporting Information (accompanied by **Fig. S42**).

As suggested, the following sentences were added on page 18 lines 9-16 in the revised manuscript: "The partial performance recovery observed during the dark intervals is consistent with the reversible day-night behavior widely reported for PSCs, which is generally associated with the thermal relaxation of light-induced metastable states in the dark[54-57]. In our system, the larger recovery amplitude of the control device suggests a more severe structural perturbation during illumination, which correlates with its larger photo-induced lattice expansion and greater residual strain accumulation. By contrast, the target device displays minimized day-night efficiency fluctuations, confirming that such structural perturbations are effectively suppressed."

On pages 18-19: "To evaluate the thermal dependence of day-night cycling degradation, we performed accelerated aging tests at 45, 65, 80, and 95 °C under repeated light-dark operations (Figure S42a). The degradation rates extracted across this temperature range were fitted using an Arrhenius model (Figure S42b). The fitted regression yields a degradation activation energy (E_a) of 0.224 eV (Figure S42c). Using 55 °C as the reference operational temperature and 95 °C as the accelerated ageing temperature, the corresponding acceleration factor (AF) was calculated to be 2.37[58,59]. On this basis, the projected T_{80} lifetime (defined as the time required for the efficiency to decay to 80% of its initial value) at 55 °C was estimated to be 78.2 cycles. These results indicate that the device degradation under day-night cycling is a thermally activated process and can be quantitatively accelerated under elevated temperature, providing a predictive framework for evaluating long-term operational durability under practical diurnal conditions."

We have added the discussion accordingly in the Supporting Information on Pages 55-56: “To quantify the thermal acceleration of device degradation under repeated light–dark transitions, accelerated aging tests were performed across a temperature series of 25, 45, 65, 80, and 95 °C. The normalized power conversion efficiency (PCE) decay profiles at each temperature step were fitted using a linear degradation function:

$$PCE(t) = 1 - kt$$

where k represents the characteristic degradation rate coefficient and t denotes the cumulative cycle number.

Because negligible degradation occurred at 25 °C within the operational measurement window, only the degradation rates extracted between 45 and 95 °C were utilized for the Arrhenius analysis. The apparent degradation activation energy (E_a) was determined from the temperature dependence of the degradation rate according to the Arrhenius relation:

$$E_a = -\frac{\partial In\left(k(T)\right)}{\partial\left(\frac{1}{k_B T}\right)}$$

where $k(T)$ is the temperature-dependent degradation rate, and k_B is the Boltzmann constant. Linear regression of the experimental data yielded an apparent degradation activation energy of 0.224 eV.

To accurately project device lifetime from the accelerated testing regimes to standard reference operating conditions, the thermal acceleration factor (AF) was evaluated as follows:

$$AF = \frac{k_{ace}}{k_{ref}} = exp\left[\frac{E_a}{k_B}\left(\frac{1}{T_{ref}} - \frac{1}{T_{ace}}\right)\right]$$

where T_{ace} and T_{ref} represent the absolute temperatures under the accelerated aging and reference operating conditions, respectively. In this evaluation, T_{ref} was defined as 55 °C (328.15 K) and T_{ref} was set to 95 °C (368.15 K).”

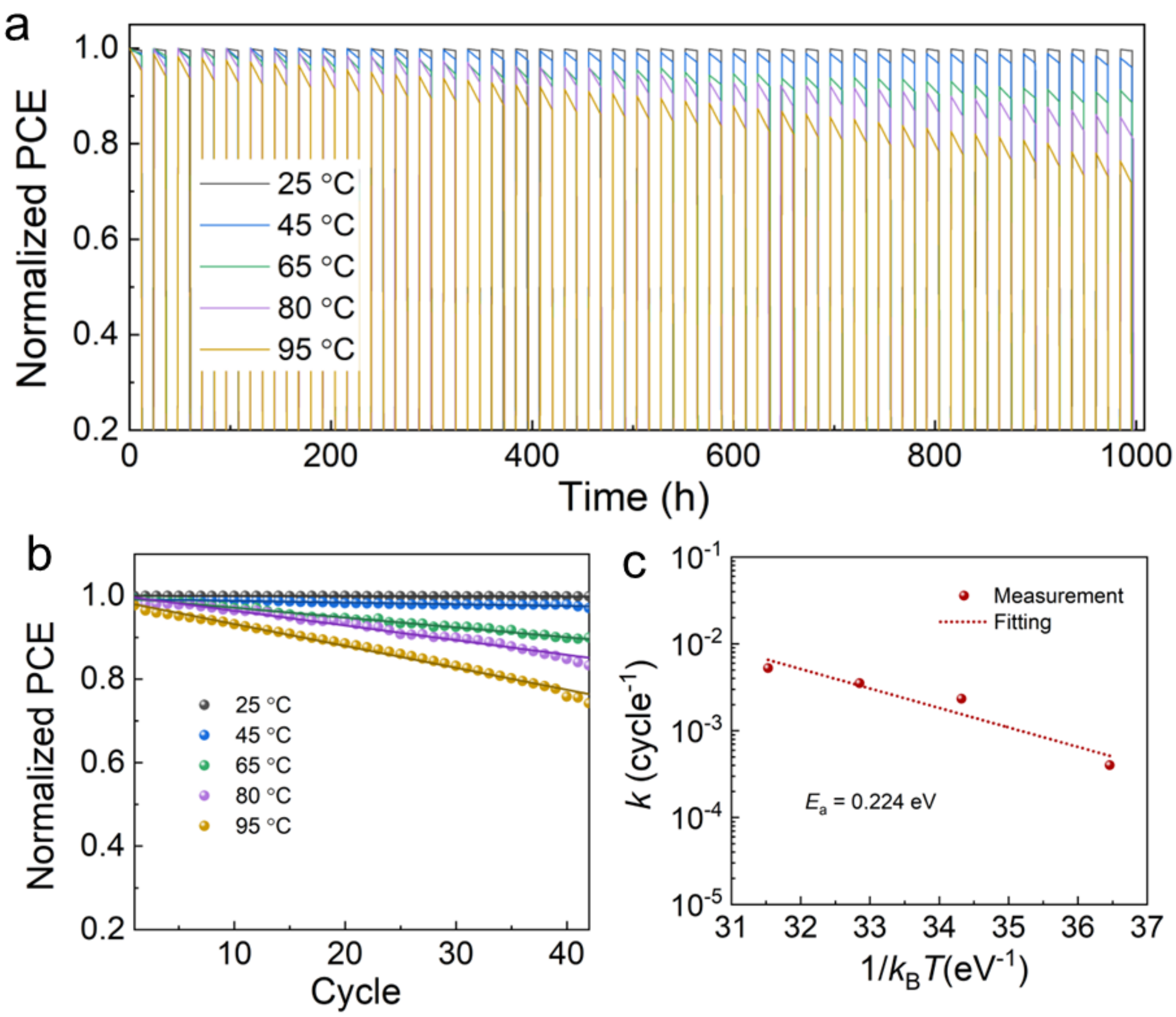

Figure S27. Accelerated ageing analysis under day/night cycling. (a) Normalized PCE evolution of the devices under repeated light-dark cycling at 25, 45, 65, 80 and 95 °C. The degradation rate increases with temperature, whereas negligible degradation is observed at 25 °C within the measured time window. (b) Normalized extracted PCE for each cycle operating under 12-hour light-dark cycling at temperatures of 25, 45, 65, 80 and 95 °C, respectively. (c) Arrhenius plot of the degradation rates extracted from the cycling data at 45-95 °C.

New references are added in the revised manuscript:

54. Khenkin MV*, et al.* Consensus statement for stability assessment and reporting for perovskite photovoltaics based on ISOS procedures. *Nat. Energy* **5**, 35-49 (2020).

55. Khenkin MV*, et al.* Reconsidering figures of merit for performance and stability

of perovskite photovoltaics. *Energy Environ. Sci.* **11**, 739-743 (2018).

56. Domanski K*, et al.* Migration of cations induces reversible performance losses over day/night cycling in perovskite solar cells. *Energy Environ. Sci.* **10**, 604-613 (2017).

57. Nie W*, et al.* Light-activated photocurrent degradation and self-healing in perovskite solar cells. *Nat. Commun.* **7**, 11574 (2016).

58. Zhao X*, et al.* Operationally stable perovskite solar modules enabled by vapor-phase fluoride treatment. *Science* **385**, 433-438 (2024).

59. Haillant O, Dumbleton D, Zielnik A. An Arrhenius approach to estimating organic photovoltaic module weathering acceleration factors. *Sol. Energy Mater. Sol. Cells* **95**, 1889-1895 (2011).

C3.8. Minor Comment: The box plot for J_{SC}, V_{OC}, and FF of the devices should be provided in the Supporting Information to better illustrate the device trend.

Authors Response:

We thank the reviewer for this valuable suggestion. As recommended, we have incorporated statistical box plots of the J_{SC}, V_{OC}, and FF for both the control and target device batches into the revised Supporting Information (**Fig. S37**). These statistical distributions provide a transparent comparison of device parameters and conclusively demonstrate the excellent reproducibility of the photovoltaic performance enhancements achieved in the target devices.

As suggested, the following sentences were revised on page 17, lines 16-17 in the revised manuscript: "The statistical distributions of the four key photovoltaic parameters collected from 30 individual devices are presented in Figure S37."

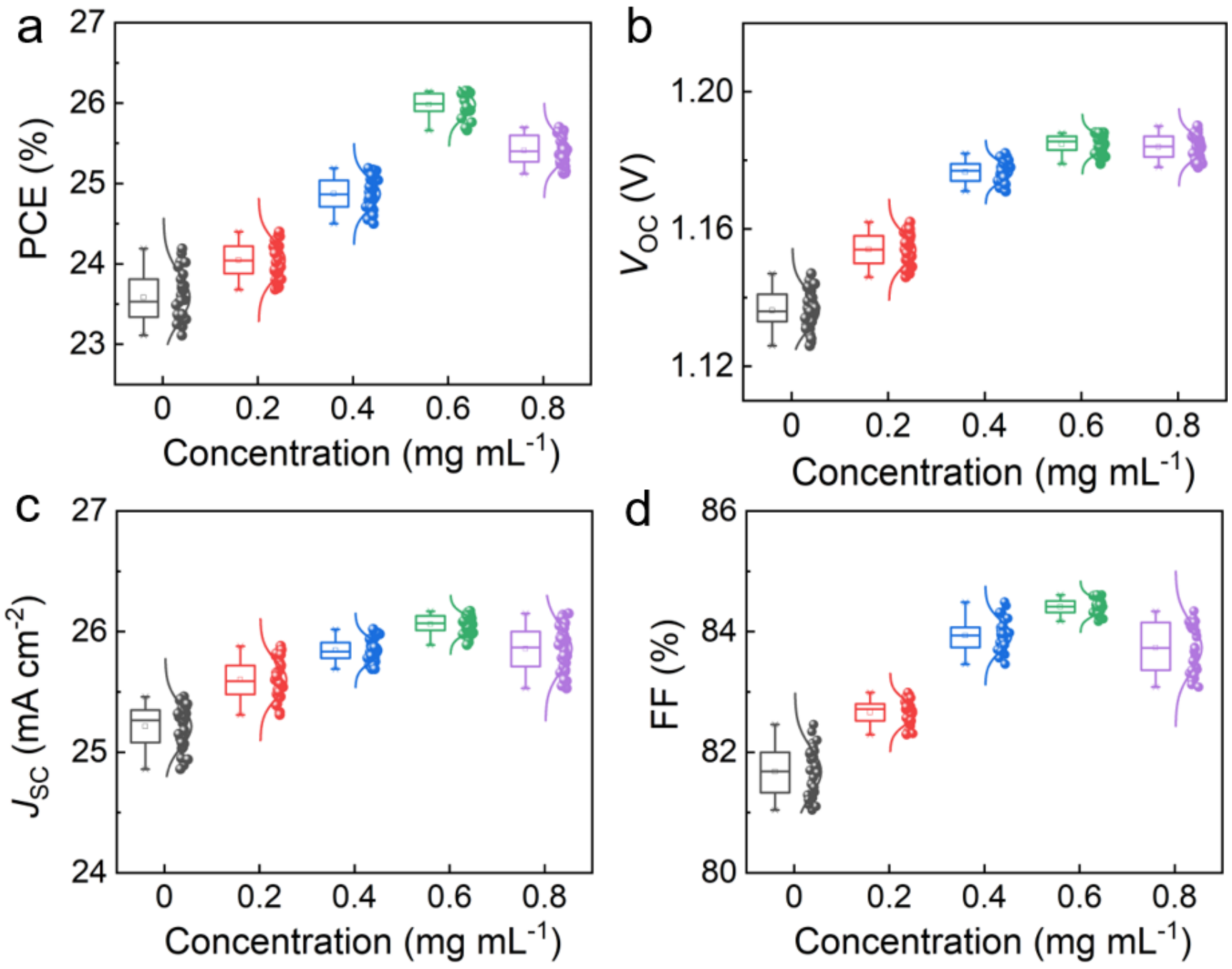

Figure S32. Photovoltaic parameters statistics of a) PCE, b) V_{OC}, c) J_{SC}, and d) FF based on 30 devices with different concentration of FPGC.

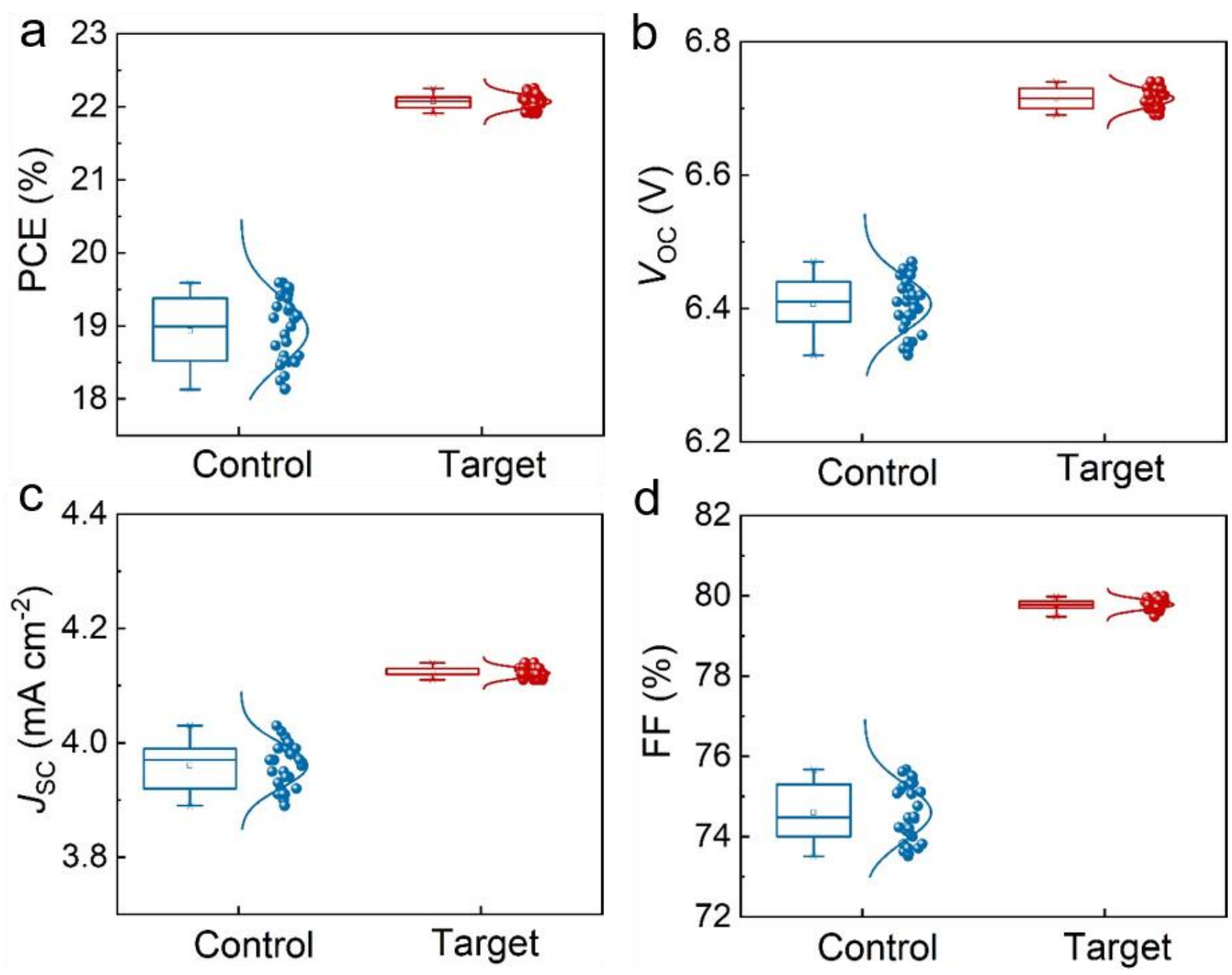

Figure S37. Photovoltaic parameters statistics of a) PCE, b) V_{OC}, c) J_{SC}, and d) FF based on 30 perovskite modules.
